# The effects of detraining on body composition and cardiometabolic health: a systematic review and meta-analysis of randomized controlled trials

**DOI:** 10.3389/fphys.2026.1891899

**Published:** 2026-07-22

**Authors:** Hao Zhang, Yiqian Zhu, Zhipeng Zhao, Dashun Tan

**Affiliations:** 1 Football School, Guangzhou Sport University, Guangzhou, China; 2 School of Physical Education and Sports Science, South China Normal University, Guangzhou, China

**Keywords:** body composition, cardiometabolic health, detraining, meta-analysis, randomized controlled trials, systematic review

## Abstract

**Background:**

Structured exercise interventions can significantly improve body composition and cardiometabolic health. However, a comprehensive synthesis of evidence regarding the effects of stopping training on body composition and cardiometabolic health outcomes remains lacking.

**Objective:**

This systematic review and meta-analysis aimed to evaluate the effect of detraining after exercise on body composition and cardiometabolic health.

**Methods:**

PubMed, Web of Science, Embase, SPORTDiscus, MEDLINE, and CINAHL were searched. Randomized controlled trials that involved an exercise and detraining phase were included. Risk of bias was assessed using Cochrane RoB2. A meta-analysis was performed to estimate effect sizes comparing pre-intervention to post-intervention and pre-intervention to post-detraining for body composition and cardiometabolic health outcomes. Subgroup analyses were stratified into short-term (≤ 2 months), medium-term (2–6 months, including 6 months), and long-term (> 6 months) intervals.

**Results:**

86 studies (n=5,670) were included. Meta-analysis indicated that after exercise withdrawal, overall reductions in Waist-to-Hip Ratio (WHR), mean arterial pressure (MAP), and fasting blood glucose (FBG) relative to baseline remained significant, and the homeostasis model assessment of insulin resistance (HOMA-IR) remained borderline significantly reduced. By detraining duration, compared with baseline, body mass, waist circumference, and maximal oxygen uptake remained significantly improved relative to baseline in the short-, medium-, and long-term subgroups; Body mass index, peak oxygen uptake, and fat mass remained significantly improved in the short- and medium-term subgroups; Systolic blood pressure remained significantly decreased in the short- and long-term subgroups; Body fat percentage remained significantly decreased in the short-term subgroup and was borderline significantly improved in the medium-term subgroup; Diastolic blood pressure, MAP, and triglycerides remained significantly improved in the short-term subgroup. High-density lipoprotein cholesterol remained significantly improved in the short-term subgroup; FBG, HOMA-IR, and fasting insulin remained significantly improved in the short-term subgroup; Fat-free mass remained significantly decreased in the medium-term subgroup; WHR, muscle mass, lean body mass, skinfold thickness, resting heart rate, and low-density lipoprotein cholesterol were no longer significant in any of the follow-up subgroups.

**Conclusions:**

Exercise-induced health benefits may not immediately return to baseline levels after detraining and may exhibit residual effects in an outcome-specific and time-dependent manner.

**Systematic Review Registration:**

https://www.crd.york.ac.uk/PROSPERO/, identifier CRD420251149494.

## 1 Introduction

Regular physical exercise is widely recognized as a cornerstone lifestyle intervention for optimizing body composition and maintaining cardiometabolic health. Through mechanisms such as autonomic regulation, improved endothelial function, and anti-atherosclerotic effects, exercise provides comprehensive multi-system benefits (Batrakoulis et al., 2022; Carson et al., 2013; Harber et al., 2017; Jensen et al., 2014; Liguori, 2020). Despite these well-documented advantages, physical inactivity remains a pervasive global health challenge, now ranking as the fourth leading risk factor for global mortality (Kohl et al., 2012; Rutters et al., 2024). This widespread lack of exercise is a primary driver of the escalating global burden of obesity and cardiometabolic diseases (CMDs), including type 2 diabetes (T2D) and cardiovascular diseases (CVDs) (Dwivedi et al., 2020; Jayedi et al., 2022; Lu et al., 2014; Mongraw-Chaffin et al., 2015).

Furthermore, even when individuals engage in structured exercise programs, real-world factors such as illness, injury, travel, and athletic off-seasons often disrupt their routines (Bosquet et al., 2013). The cessation of this training stimulus is defined as “detraining”. This phenomenon is typically characterized by the partial or complete reversal of training-induced physiological adaptations (Mujika and Padilla, 2000a). Physiologically, this rapid regression is driven by central hemodynamic reductions and peripheral maladaptation’s, including diminished muscle fiber area, capillary density, and oxidative enzyme activity. Coupled with impaired insulin sensitivity and altered substrate utilization, these multi-system decrements worsen lactate kinetics, compromising cardiovascular function and metabolic efficiency within mere days of inactivity, which ultimately manifests as detrimental shifts in macroscopic health markers such as increased adiposity, dyslipidemia, and impaired glycemic control (Barbieri et al., 2024; Hamilton et al., 2007; Mujika and Padilla, 2000a, 2000b). Evidence suggests that this regression can be swift and clinically significant. For instance, one study demonstrated that after 12 weeks of aerobic interval training, a subsequent 4-week cessation period caused peak oxygen uptake (VO_2peak_) and high-density lipoprotein levels to revert to pre-training baselines (Nikseresht et al., 2016). Similarly, another investigation found that following just 2 weeks of detraining after a 6-month intervention, 65.5% of systolic blood pressure responders and 80.6% of diastolic blood pressure responders lost their beneficial adaptations (Moker et al., 2014). Furthermore, existing evidence indicates that a 4-week detraining phase following structured exercise training is sufficient to elicit significant increases in body mass (Głyk et al., 2022).

Interestingly, despite these findings, the literature presents a complex picture, with some studies reporting contradictory outcomes. Some evidence suggests that specific exercise-induced benefits may be partially preserved, or exhibit a “residual effect”, following training cessation. For example, improvements in waist circumference achieved through combined resistance and aerobic training were preserved after a 1-month detraining phase (Nikseresht et al., 2016). Additionally, in contrast to the rapid loss of blood pressure adaptations mentioned earlier, blood pressure adaptations induced by 6 months of moderate-intensity training in older adults were largely maintained throughout a subsequent 4-month detraining period (Subías-Perié et al., 2024).

This inconsistency may be attributed to variations in study populations, initial training intensity, and, crucially, the duration of the detraining period. However, efforts to clarify these discrepancies are hindered by critical limitations in the available synthesized evidence. Although a limited number of reviews have addressed the phenomenon of detraining, significant knowledge gaps remain. Current meta-analyses predominantly focus on specific demographic groups (e.g., older adults or athletes) or isolate singular physiological parameters (e.g., muscle strength or left ventricular mass) (Grgic, 2022; Massarotto et al., 2024; Yang et al., 2021; Zheng et al., 2022, 2025). Consequently, these analyses lack a comprehensive assessment of multi-system health markers, including body composition and cardiometabolic health, across the broader population.

Given these critical shortcomings and the conflicting conclusions across individual randomized controlled trials (RCTs), there is an urgent need to systematically synthesize the global evidence and elucidate the exact impact of detraining. Accordingly, the present systematic review and meta-analysis aim to bridge this void by quantifying the persistent effects of detraining on body composition and cardiometabolic health. The primary outcomes of this study are body mass and maximal oxygen uptake (VO_2max_). Additional body composition metrics and other cardiometabolic health markers were evaluated as exploratory analyses. Importantly, to accurately isolate the true residual effects of prior exercise from natural temporal changes or regression-to-the-mean effects, the primary analytic contrast of this study relies on between-group comparisons, evaluating the differences between the exercise groups and the non-exercising control groups at different post-detraining time points. Specifically, we conducted subgroup analyses to compare the magnitude of physiological regression across varying detraining durations. By clarifying how different lengths of inactivity dictate the decay of health outcomes, this study seeks to provide evidence-based insights for developing more resilient and sustainable public health exercise guidelines.

## 2 Methods

This systematic review and meta-analysis were conducted in accordance with the guidelines outlined in the Preferred Reporting Items for Systematic Reviews and Meta-Analyses (PRISMA) statement (Page et al., 2021). The protocol is prospectively registered with PROSPERO (CRD420251149494).

### 2.1 Search strategy

A comprehensive and systematic literature search was performed across the following primary electronic databases: PubMed, Web of Science, Embase, and three databases accessed via the EBSCOhost platform, namely SPORTDiscus, MEDLINE, and CINAHL. The search spanned from database inception to December 27, 2025. The search was restricted to English-language publications. Boolean operators (“AND” and “OR”) were utilized to combine four principal conceptual domains: (1) exercise modalities (e.g., “exercise”, “physical activity”, “aerobic exercise”, “resistance exercise”, “Tai Chi”, “yoga”), (2) detraining and physical deconditioning (e.g., “detraining”, “training cessation”, “tapering”, “training interruption”), (3) body composition and cardiometabolic outcomes (e.g., “body fat percentage”, “maximal oxygen uptake”, “blood pressure”, “lipoproteins”, “C-reactive protein”, “glycated hemoglobin”), and (4) study design (restricted to randomized controlled trials). The detailed search strategy for this review is provided in Supplementary File 1.

### 2.2 Inclusion and exclusion criteria

The eligibility criteria were established following the PICOS framework, which encompasses Population, Intervention, Comparator, and Outcomes (Schardt et al., 2007). This framework provides a structured methodology for evaluating study eligibility based on explicitly defined criteria. In this systematic review, detraining was defined as complete cessation of the prescribed exercise training protocol during the follow-up period (i.e., no continued structured training). The detailed inclusion and exclusion criteria for this review are outlined in **Table 1**.

**Table 1:** Inclusion and exclusion criteria.

Category	Inclusion criteria	Exclusion criteria
Population	No restrictions were placed on age, sex, or baseline health status.	Animal studies.
Intervention	Structured exercise training followed by a defined detraining phase, defined as complete cessation of the prescribed training protocol during the follow-up period (i.e., no continued structured training), with explicitly reported protocol parameters (e.g., volume, intensity).	Multi-component interventions confound the independent effect of exercise (e.g., combined diet/pharmacotherapy) and poorly defined training or detraining protocols.
Comparator	Passive, non-exercising control groups (e.g., sedentary, waitlist, or usual care) maintain habitual routines.	Lack of a true non-exercising control (e.g., exclusive comparisons between different exercise modalities).
Outcomes	Quantitative data for ≥1 body composition or cardiometabolic outcome reported across three time points: baseline, post-intervention, and post-detraining (follow-up).	Absence of requisite post-detraining (follow-up data); insufficient statistical reporting precluding meta-analysis.
Study design	Peer-reviewed randomized controlled trials (RCTs).	Non-randomized trials, observational studies, case reports, reviews, and conference abstracts.

### 2.3 Study selection and data extraction

Initially, all bibliographic records retrieved from the six major electronic databases were imported into EndNote 21.5 reference management software for consolidation. Duplicate records were subsequently eliminated using the software’s automated deduplication function, complemented by rigorous manual, record-by-record verification.

**Figure 1:**
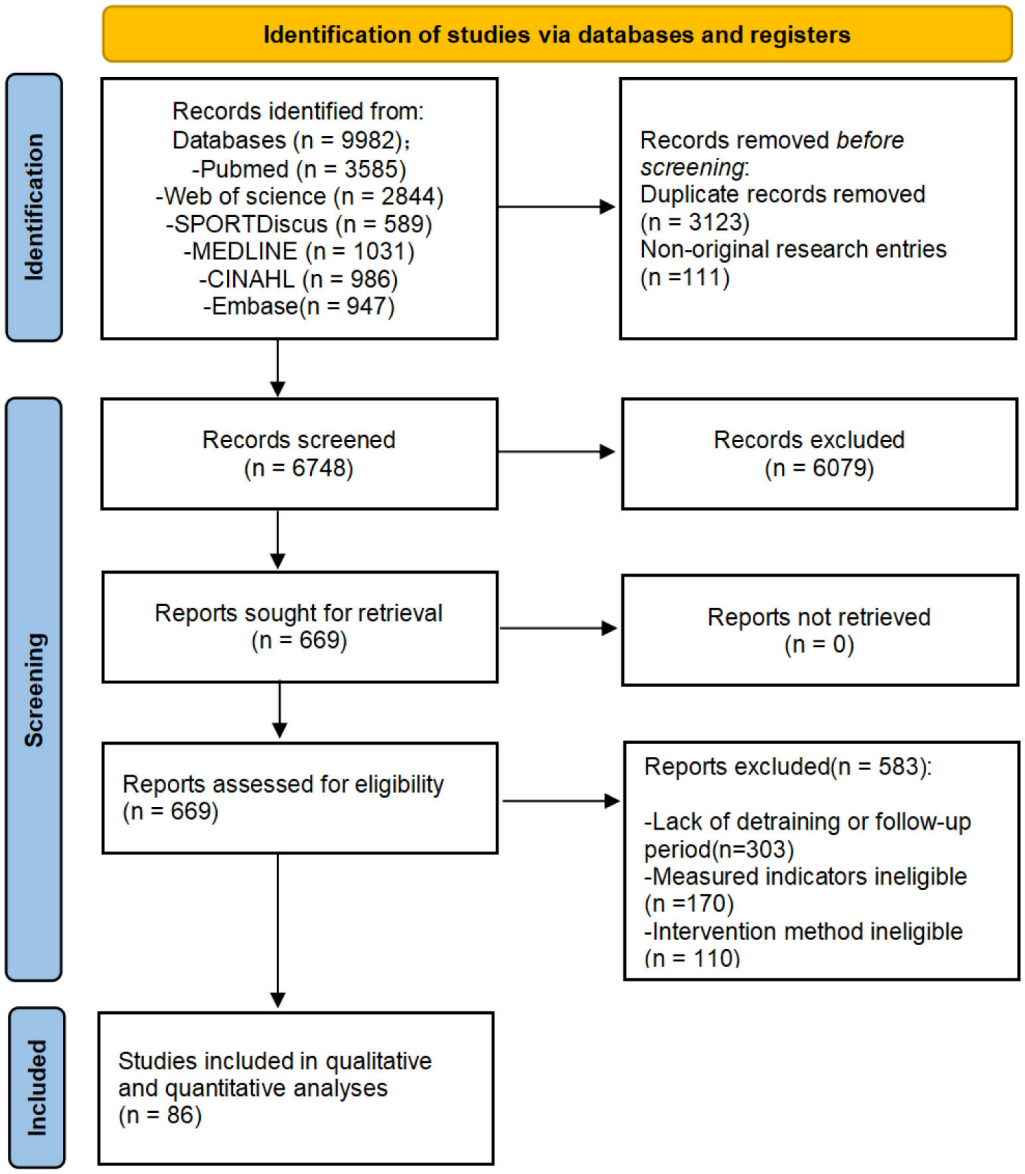
PRISMA flow-chart of literature search.

Study selection and data extraction were conducted independently by two investigators in a blinded manner, strictly adhering to a two-phase standardized protocol. In the first phase, the two investigators independently reviewed titles and abstracts based on the predefined PICOS criteria to exclude evidently irrelevant studies. In the second phase, the full texts of potentially eligible articles were retrieved for in-depth evaluation. Any discrepancies arising between the two reviewers during this process were first resolved through consensus discussion; if a consensus could not be reached, the issue was submitted to a third senior researcher with extensive systematic review experience for arbitration. The complete literature search and selection process is delineated in **Figure 1**.

Following the confirmation of the final included literature, the two investigators independently extracted key data using a standardized, pre-piloted electronic data extraction form. The extracted information primarily encompassed: (1) general study characteristics: author’s name, publication year, and study design type; (2) participant demographics: sample size, age profile, sex distribution, and baseline health status; (3) intervention protocol: specific exercise modality, duration per session, weekly training frequency, and the precisely defined duration of the detraining period; (4) outcome measures. The extracted outcome data were systematically categorized into two primary domains: body composition and cardiometabolic health. Specifically, body composition indices included body mass, body mass index (BMI), body fat percentage (BF%), fat mass (FM), waist circumference (WC), waist-to-hip ratio (WHR), fat-free mass (FFM), muscle mass (MM), lean body mass (LBM), and skinfold thickness. Cardiometabolic health indicators were further categorized into hemodynamic parameters (resting heart rate (RHR), systolic blood pressure (SBP), diastolic blood pressure (DBP), mean arterial pressure (MAP)), cardiorespiratory fitness (peak oxygen uptake (VO_2peak_), VO_2max_), lipid profiles (high-density lipoprotein cholesterol (HDL-C), low-density lipoprotein cholesterol (LDL-C), total cholesterol (TC), triglycerides (TG)), and glycemic control indices (fasting blood glucose (FBG), fasting insulin, homeostasis model assessment of insulin resistance (HOMA-IR)). To ensure the statistical rigor of the subsequent meta-analysis, a third investigator conducted a strict cross-verification and review of all extracted data for accuracy and completeness prior to quantitative synthesis.

### 2.4 Risk of bias assessment

Two independent investigators critically appraised the methodological quality of all included RCTs. This assessment strictly adhered to the guidelines outlined in the Cochrane Handbook for Systematic Reviews of Interventions, utilizing the revised Cochrane risk-of-bias tool for randomized trials (RoB 2.0). The evaluation systematically scrutinized potential sources of bias across five core domains: (1) randomization process; (2) deviations from intended interventions; (3) missing outcome data; (4) measurement of the outcome; and (5) selection of the reported result (Sterne et al., 2019).

Within each domain, investigators followed the preset algorithm of “signaling questions” to systematically deduce the risk level, precisely categorizing the risk for each domain as “low risk”, “some concerns”, or “high risk”. The overall risk of bias for each study was determined strictly according to the official algorithm outlined by Sterne et al. (2019). Specifically, a study was judged to be at high risk of bias overall if at least one domain was rated as high risk, or if there were some concerns in multiple domains that were judged to substantially lower confidence in the result. For all other combinations, the overall risk was determined by the algorithmic pathways corresponding to low risk or some concerns. The two assessors conducted the evaluation independently and were blinded to each other’s judgments. Disagreements were resolved through in-depth internal discussion or by consulting a third investigator for arbitration. The final risk of bias assessment results are visually presented via a risk-of-bias matrix (traffic-light plot) and a summary bar plot, generated using the robvis visualization tool (McGuinness and Higgins, 2021).

### 2.5 Certainty of evidence

The overall certainty of evidence for all pooled outcomes was evaluated using the GRADE (Grading of Recommendations Assessment, Development and Evaluation) framework. Given that all included studies were RCTs, the initial evidence quality for each outcome was designated as “high”. Subsequently, investigators rigorously scrutinized the evidence quality based on five potential downgrading factors: risk of bias (study limitations), inconsistency, indirectness, imprecision, and publication bias. Ultimately, the certainty of evidence for each pooled outcome was rated into one of four levels: “high”, “moderate”, “low”, or “very low”, culminating in the construction of a GRADE summary of findings table (Guyatt et al., 2011).

### 2.6 Statistical analysis

To preserve statistical independence and prevent unit-of-analysis errors during data synthesis, the following *a priori* rules were applied: (1) For multi-arm trials sharing a single control group, the sample size of the control group was divided evenly among the active intervention arms. (2) For studies reporting multiple follow-up time points, data were stratified into separate, independent subgroup analyses based on the time interval. To avoid double-counting, no overall pooled effect was calculated by combining different time points from the same participants.

Review Manager (RevMan 5.4) served as the primary software for meta-analyses and data synthesis, with statistical significance established at *p* < 0.05. For continuous outcomes, the mean difference (MD) and its 95% confidence interval (95% CI) were calculated as the pooled effect size if studies utilized consistent measurement tools. Alternatively, the standardized mean difference (SMD) and 95% CI were employed if varying measurement instruments or units were utilized. To accurately assess intervention efficacy, the change scores (post-intervention or follow-up values minus baseline values) and their corresponding standard deviations (SDs) were uniformly calculated using the formulas recommended by the Cochrane Handbook for Systematic Reviews of Interventions for between-group synthesis (Chandler et al., 2019).

Due to the expected clinical and methodological heterogeneity across studies (e.g., variations in participant characteristics, exercise modalities, and detraining protocols), we used a random-effects model for all meta-analyses. Statistical heterogeneity across the included studies was evaluated using Cochran’s Q test alongside the I^2^ statistic. Furthermore, to elucidate the impact of detraining on the residual characteristics of intervention effects relative to baseline, subgroup analyses were conducted for all outcomes based on detraining duration, which was strictly defined at the boundaries as short-term (≤ 2 months), medium-term (2–6 months, including 6 months), and long-term (> 6 months) strata. These detraining-duration boundaries were initially informed by recent detraining-related literature (Zheng et al., 2025). The exact thresholds were further adjusted according to the distribution of follow-up assessment times reported across included trials to avoid empty or extremely sparse strata and to maintain the interpretability and statistical feasibility of subgroup analyses. Concurrently, a sensitivity analysis utilizing the leave-one-out method was performed to assess the influence of individual studies on the effect size and to verify the robustness of the results. Moreover, to address the substantial discrepancy in follow-up durations between Brobakken (2025) and Anandavadivelan (2024) (4.5 years and 2 years/5 years) and the rest of the included studies, we conducted an additional sensitivity analysis entirely excluding these two trials. Finally, potential publication bias was qualitatively assessed via visual inspection of funnel plots generated in RevMan 5.4. For outcomes encompassing ≥ 10 independent studies, Egger’s regression intercept test was additionally executed using Stata 18.0 to quantitatively evaluate and perform statistical significance testing for publication bias. If publication bias was detected, the trim-and-fill method was applied using Stata 18.0 to adjust the pooled effect size.

## 3 Results

The initial literature search across major electronic databases identified 9,982 relevant records. During the full-text screening phase, 583 articles were excluded based on predefined PICOS criteria. Ultimately, 86 randomized controlled trials (RCTs) fully met the inclusion criteria and were included in this meta-analysis. The detailed flowchart is provided in **Figure 1**.

### 3.1 Characteristics of included studies

Supplementary Information 1 summarizes the characteristics of the included studies. The 86 RCTs were published between 1993 and 2025 and comprised a total of 5,670 participants. Participant ages ranged from 8.28 to 83.5 years. Study populations spanned healthy adults and various clinical cohorts (e.g., obesity, hypertension, cancer survivors, and pulmonary fibrosis). Exercise interventions lasted 2 to 24 weeks, with frequencies ranging from 1 to 5 sessions per week. Intervention modalities included aerobic exercise, resistance training, isometric handgrip training, combined training, stretching, and mind-body exercises (such as yoga and Pilates). Specifically, 86% of the interventions were fully supervised, 4% were hybrid, and 10% were unsupervised. The detraining periods ranged from 1 week to 5 years.

### 3.2 Risk of bias assessment

Assessment via the Cochrane RoB 2.0 tool indicated that 5% of studies had a low risk of bias, 85% had some concerns, and 10% were at high risk. The predominant sources of bias were the randomization process (D1) and selection of reported results (D5), with approximately 65% of studies lacking sufficient detail on allocation concealment or pre-registered protocols. Although blinding of participants and personnel (D2) was largely infeasible given the nature of exercise interventions, the potential for bias was mitigated by using objective outcome measures. Detailed assessments are provided in **Supplementary Figure 2** and **Supplementary Figure 3**.

### 3.3 Meta-analysis

Meta-analyses suggest that residual benefits of structured exercise may vary in a time-dependent and outcome-specific manner after detraining.

#### 3.3.1 Body composition

The aggregate meta-analysis indicates that exercise-induced improvements in most body composition parameters remained significantly after detraining relative to baseline, with the magnitude of residual effects varying by indicator. Exact overall and subgroup estimates, 95% CIs, and p-values for subgroup differences by follow-up duration are presented in **Supplementary Table 3**.

No significant exercise−induced changes were observed at post−intervention for the following outcomes: BF% in the long−term subgroup; FM in the long−term subgroup; FFM in the short−term subgroup; and MM, LBM, and skinfold thickness in overall and any subgroup. Consequently, the absence of residual effects during detraining for these specific variables should not be interpreted as loss of training efficacy, but rather as a lack of initial responsiveness to the exercise intervention.

Following withdrawal of the exercise intervention, the waist-to-hip ratio remained significantly decreased compared with baseline overall. Body mass and WC remained significantly decreased compared with baseline in the short-, medium-, and long-term subgroups (SI 2 **Figures 27** and **32**). Across these intervals, the mean reductions in body mass ranged from 1.12 to 2.09 kg and in WC from 1.43 to 2.17 cm. BMI and FM remained significantly decreased relative to baseline in the short- and medium-term subgroups (SI 2 **Figures 29** and **31**). However, when assessed against established clinical benchmarks, these residual effects appeared modest. The observed weight reductions may not have reached the ≥5% threshold for clinically meaningful weight loss (Nadolsky et al., 2025). For WC, only the short−term reduction (−2.17 cm) exceeded the 2−cm minimum clinically important difference (Jayedi et al., 2024). BF% remained significantly decreased relative to baseline in the short-term subgroup and showed a borderline significant decrease in the medium-term subgroup. (**Supplementary Figure 30**). The test for subgroup differences was significant for BF% (*p* = 0.04), suggesting a possible difference in the magnitude of the retained BF% effect across follow-up durations. The residual benefits for BF% may gradually attenuate as detraining duration increases. Meanwhile, FFM was significantly increased relative to baseline in the medium-term subgroup (**Supplementary Figure 35**). In contrast, WHR, MM, LBM, and skinfold thickness did not demonstrate significant changes relative to baseline in any follow-up subgroups (**Supplementary Figures 32**, **36**, **37**).

#### 3.3.2 Cardiometabolic health

The pooled analyses also indicated that several cardiometabolic adaptations were retained after exercise cessation, but the pattern varied substantially by outcome and detraining duration. Exact overall and subgroup estimates, 95% CIs, and p-values for subgroup differences by follow-up duration are presented in **Supplementary Table 3**.

No significant exercise−induced changes were observed at post−intervention for the following parameters: SBP (medium−term subgroup); DBP (medium− and long−term subgroups); TC (medium− and long−term subgroups); HDL−C (medium− and long−term subgroups); LDL−C (any subgroup); TG (medium- and long−term subgroups); FBG (medium− and long−term subgroups); fasting insulin (overall and all subgroups); and HOMA−IR (medium−term subgroup). Accordingly, the absence of residual effects during detraining for these variables should not be interpreted as a loss of training efficacy, but rather as a reflection of the absence of an initial intervention response.

Following withdrawal of the exercise intervention, significant overall changes in FBG and MAP persisted, and HOMA-IR remained borderline significantly reduced relative to baseline (*p* = 0.05). By detraining duration, compared with baseline, DBP, MAP, HDL-C, TG, FBG, HOMA-IR, and fasting insulin were significant only in the short-term subgroup (**Supplementary Figures 39**, **40**, **46**, **47**, **48**, **50**). In contrast, VO_2peak_ remained significantly increased relative to baseline in the short- and medium-term subgroups (**Supplementary Figure 41**); VO_2max_ remained significantly increased relative to baseline in all three follow-up subgroups (**Supplementary Figure 43**), with SMDs ranging from 0.29 to 0.62 across these periods. SBP remained significantly decreased relative to baseline in the short- and long-term subgroups (**Supplementary Figure 38**). Notably, the evidence for these trends is partly characterized by borderline significance for VO_2max_ in the medium-term (5 studies, SMD = 0.42, 95% CI 0.03 to 0.81, *p* = 0.04, I^2^ = 0%) and SBP in the short-term (12 studies, MD = −2.34, 95% CI −4.41 to −0.28, *p* = 0.03, I^2^ = 0%), necessitating a cautious interpretation of the residual effects over time. LDL-C and RHR did not remain significantly different from baseline in any follow-up subgroup after detraining (**Supplementary Figures 41** and **45**). Finally, a paradoxical pattern was observed for fasting insulin: although its post−intervention effect was non−significant overall (7 studies, SMD = −0.86, 95% CI −1.87 to 0.14, *p* = 0.09, I^2^ = 89%) and in the short−term subgroup (5 studies, SMD = −1.27, 95% CI −3.64 to 0.49, *p* = 0.09, I^2^ = 92%), both became significant during the detraining phase relative to baseline (Overall: 7 studies, SMD = −0.41, 95% CI −0.73 to −0.08, *p* = 0.006, I^2^ = 12%); Short-term: 5 studies, SMD = −0.51, 95% CI −0.95 to −0.08, *p* = 0.02, I^2^ = 34%).

### 3.4 Sensitivity analysis

Following the sequential exclusion of individual studies, the effect sizes and their confidence intervals for core outcome indicators did not show substantial directional changes, indicating that the core conclusions of this meta-analysis may not rely on any single extreme study and that the results may be considered reasonably robust. The sensitivity analysis plots are detailed in **Supplementary Figures 4**-**26**. In an additional sensitivity analysis excluding the very-long-term follow-ups, the statistical significance and direction of effects appear to be generally consistent with those observed in the main analysis. These findings may indicate that the core conclusions of our study might not be affected by the inclusion of a few very-long-term studies, and the robustness of our findings might be considered reasonably supported. Detailed results are shown in **Supplementary Figure 27**.

### 3.5 Publication bias

Publication bias was detected for four outcomes: Body mass (short-term subgroup), BMI (short-term subgroup), WC (long-term subgroup), and RHR (short-term subgroup). Subsequent trim-and-fill analyses were conducted to adjust the effect sizes representing the changes from baseline to follow-up. For body mass (short-term), BMI (short-term), and WC (long-term), trim-and-fill imputed several potentially missing studies, and the adjusted pooled effect estimates showed the same direction as the unadjusted estimates with a larger magnitude. This pattern suggests that publication bias, if present, may have resulted in an underestimation of the true effects. In contrast, for RHR (short-term), publication-bias adjustment materially altered statistical inference. Specifically, the unadjusted pooled estimate was non-significant (MD = 0.713; 95% CI: −0.836 to 2.263), whereas the trim-and-fill adjusted estimate became statistically significant (MD = 1.730; 95% CI: 0.325 to 3.135) after imputing six potentially missing studies. Thus, the conclusion regarding this outcome may be more sensitive to publication bias. These quantitative results are detailed in **Table 2**, and the adjusted funnel plots can be found in **Supplementary Figures 73**, **74**, **75**, and **76**.

**Table 2:** Adjusted pooled estimates using the trim-and-fill method (baseline to follow-up).

Outcomes (subgroup)	Analysis type	Observed	Imputed	MD (95% CI)
Body mass(short-term)	Original	20	/	−2.094 (−2.997, −1.190)
	Adjusted	20	9	−2.391 (−3.248, −1.533)
BMI (short-term)	Original	16	/	−0.623 (−1.034, −0.212)
	Adjusted	16	8	−1.121 (−1.611, −0.630)
WC (long-term)	Original	10	/	−1.894 (−2.748, −1.040)
	Adjusted	10	5	−2.134 (−2.939, −1.328)
RHR (short-term)	Original	14	/	0.713 (−0.836, 2.263)
	Adjusted	14	6	1.730 (0.325, 3.135)

### 3.6 Certainty of evidence

GRADE-based assessment indicated that the overall certainty of evidence for most key outcomes (e.g., body mass, BMI, SBP, DBP, and VO_2peak_) was generally rated as moderate, suggesting that the available evidence can provide some support for clinical decision-making. However, due to the lack of effective blinding inherent to exercise interventions and the resulting imprecision in long-term follow-up studies, attributable to a limited number of included RCTs and relatively small cumulative sample sizes, evidence for certain long-term residual effects was downgraded to low or very low. Accordingly, interpretations of these long-term outcomes should be made cautiously. The detailed GRADE evidence profile is provided in **Supplementary Table 4**.

## 4 Discussion

This study aimed to systematically quantify the residual effects on body composition and cardiometabolic health benefits after structured exercise interventions were discontinued. A total of 86 randomized controlled trials were included, with 5,670 participants. The findings were broadly consistent with prevailing theories regarding detraining effects and also extended current understanding in this area. Prior research has shown that detraining can partially or fully reverse exercise-induced physiological adaptations (Mujika and Padilla, 2000a, 2000b). Our meta-analysis further suggests that exercise-induced health benefits do not abruptly revert to pre-training baseline levels immediately after detraining, but rather exhibit residual effects in an outcome-specific and time-dependent manner.

### 4.1 Comparison with previous studies and mechanistic explanations

Regarding body composition, meta-analysis showed that the residual benefits of body mass and WC remained statistically significant relative to baseline after more than 6 months of detraining. However, Further analyses indicated that the residual benefits for BF% may gradually attenuate as detraining duration increases, whereas FFM remained significantly reduced in the medium-term follow-up period relative to baseline. This suggests that the residual effects on body mass and WC may persist after detraining, but may not necessarily correspond to the actual changes in overall body composition. This pattern may be explained by two key factors. First, the prolonged residual effects of body mass and WC could partly reflect continued behavior spillover effects generated during the intervention period. Although monitoring of lifestyle changes during the detraining period was relatively limited across included studies, increases in non-exercise physical activity associated energy expenditure and improvements in dietary patterns may have persisted for some time after stopping structured exercise (King et al., 2007). This observation also aligns with the biological characteristics of skeletal muscle. As a highly mechanosensitive tissue, skeletal muscle can rapidly downregulate mechanistic target of rapamycin-related protein synthesis signaling upon withdrawal of mechanical loading, leading to reduced rates of muscle fiber protein synthesis (Drummond et al., 2012; Glover et al., 2008). Previous meta-analyses have demonstrated that skeletal muscle size and strength tend to decline after resistance training cessation, particularly in older adults (Bosquet et al., 2013; Grgic, 2022). Therefore, while body mass and WC remained significantly reduced compared with baseline, this may not fully capture overall changes in body composition after detraining.

For cardiorespiratory fitness, meta-analysis showed that after more than 6 months of detraining, participants still had a significantly higher VO_2max_ than the control group. This finding differs from evidence from studies focused on athletes, where VO_2max_ has been reported to decline substantially after detraining, with the magnitude of the decline related to detraining duration (Zheng et al., 2022). Several alternative explanations may account for this apparent long-term residual effect. First, the participants included in the present review were mostly drawn from general populations or clinical settings, rather than trained athletes. Their baseline cardiorespiratory fitness may therefore have been relatively low, and exercise-induced improvements may have remained detectable for a longer period after detraining. Second, differences in study populations, including age, clinical status, baseline health condition, and prior training exposure, may have contributed to heterogeneous post-detraining responses. Third, the number of studies contributing to the long-term detraining subgroup was limited, and the corresponding sample size was relatively small. As a result, the pooled estimate may be less stable and more susceptible to the influence of individual studies. Fourth, habitual physical activity during the detraining period was not consistently monitored in the original studies; therefore, some participants may have continued informal or unsupervised physical activity, which may have attenuated the loss of VO_2max_ adaptations and contributed to the apparent persistence of benefit. Mechanistically, if this residual effect is confirmed in future studies, the long-term persistence of residual effects of VO_2max_ may relate to both central and peripheral adaptive changes induced by exercise. These adaptations include increased stroke volume, structural remodeling of the left ventricle, improved vascular endothelial function, increased capillary density, and enhanced oxidative capacity in skeletal muscle (Fiuza-Luces et al., 2018; Green et al., 2017; Hellsten and Nyberg, 2016). These relatively stable structural and functional adaptations may partly explain why VO_2max_, compared with some metabolic indicators, is more resistant to detraining-related attenuation.

Regarding blood pressure, meta-analysis evidence indicates that even after more than 6 months of detraining, SBP remains significantly reduced relative to baseline, with a mean decrease of 2.69 mmHg. From a public health perspective, even a reduction of 2 to 3 mmHg in SBP can confer significant cardiovascular risk benefits (Jung et al., 2019; Lewington et al., 2003). Nevertheless, the effect size is modest, and the clinical relevance at an individual level may vary depending on baseline risk, antihypertensive medication use, and whether lifestyle maintenance persists after structured training cessation. The prolonged residual effect on SBP after detraining may be explained by the predominance of determinants such as large-artery elasticity, central aortic pressure, vascular structural remodeling, and endothelial function. In contrast to other cardiovascular parameters, exercise-induced vascular structural adaptations may persist for a relatively longer period following detraining, thereby sustaining the SBP-lowering effect (Green et al., 2017).

In terms of glucolipid metabolism, meta-analysis showed that residual benefits for HDL−C, TG, FBG, and HOMA−IR may be confined to a short−term detraining window, which is consistent with the mechanism identified in previous studies that metabolic adaptations are highly dependent on recent muscle contractions. Specifically, skeletal muscle lipoprotein lipase activity and GLUT4 translocation to the cell membrane are both regulated by recent contractile stimuli; therefore, insulin sensitivity may decrease substantially within a few days after exercise cessation (Hamilton et al., 2007; Hansen et al., 2012). Notably, meta−analysis revealed that fasting insulin exhibited a delayed pattern of change: following the intervention, neither the overall effect nor the short-term detraining subgroup effect reached statistical significance. However, after detraining, both effects became significant, accompanied by a substantial reduction in between-study heterogeneity. Physiologically, a newly emerging improvement in fasting insulin after exercise cessation is difficult to explain, as insulin-related adaptations would generally be expected to become less pronounced during detraining. Therefore, this finding may partly reflect methodological or statistical artefacts rather than a true delayed physiological effect. Differences in the timing of the last exercise session, dietary control, medication use, and blood sampling protocols may have introduced acute exercise-related variation or measurement error, thereby obscuring the post-intervention effect. Another tentative possibility is that, after the acute physiological perturbations associated with the final exercise sessions had diminished, a more stable metabolic steady-state signal may have become more apparent during detraining (Bird and Hawley, 2017; Heath et al., 1983). However, this explanation remains speculative. In addition, the small number of contributing studies increases the possibility of chance findings, and the pooled estimate may have been influenced by one or a few study-specific effects. Residual confounding from unmonitored diet, physical activity, weight change, or medication use during the detraining period may also have contributed to the observed result. Thus, this finding should not be regarded as evidence that detraining improves fasting insulin, but rather as a preliminary and potentially unstable observation requiring confirmation in larger studies with standardized measurement and follow-up protocols.

### 4.2 Clinical and public health implications

This study offers insights for public health and clinical exercise prescription on how to address the return to baseline in training effects following exercise cessation. First, exercise-induced benefits do not immediately revert to baseline upon detraining, and improvements in core indicators such as body mass, WC, and VO_2max_ may remain elevated above baseline for a period. This finding helps correct the misconception that “stopping exercise nullifies all previous gains” and enhances the confidence of individuals who experience short−term training interruptions due to illness, travel, or life stressors to resume exercise. Secondly, exercise prescriptions should shift from simply emphasizing continuous training to a more realistic, context-specific “resilience-based prescription”, namely, establishing minimum-dose strategies to help preserve training benefits during the inevitable periods of detraining. When evaluating the efficacy of weight management during the detraining period, exclusive focus on body mass, BMI, or WC may be limited. Instead, changes in MM, FM, and functional indicators should be assessed simultaneously, with vigilance for the potential risk of occult sarcopenia during detraining (Prado et al., 2012).

### 4.3 Limitations and future directions

Several limitations should be noted. First, substantial heterogeneity was observed across enrolled populations and exercise regimens. Participants differed in age, baseline health status, and clinical conditions, and exercise interventions varied in intensity, duration, and frequency. In addition, multiple exercise modalities were pooled in the main analyses. Because different modalities may induce distinct physiological adaptations, regression after training cessation may differ; therefore, the pooled estimates should be interpreted as overall effects across heterogeneous modalities rather than modality-specific detraining patterns. Time-varying changes over the life course and disease progression may also interact with detraining effects, introducing potential confounding. Although detraining duration is a key determinant of residual effects, nested subgroup analyses stratified by both time and population or exercise modality were not feasible due to data sparsity, while subgroup analyses based only on population or modality would be heavily confounded by time. Thus, residual confounding by population and exercise modality may persist within temporal strata, warranting larger future studies. Second, multiplicity should be acknowledged. Although the main emphasis of this review was on VO_2max_ and body mass, multiple cardiometabolic and body composition outcomes were also examined across different detraining duration subgroups. Because no formal adjustment for multiple comparisons was applied, some statistically significant findings, especially those from secondary outcomes or subgroup analyses, may have occurred by chance. Therefore, these findings should be regarded as exploratory and hypothesis-generating rather than confirmatory. Third, the strength of conclusions is limited by the methodological quality of the included evidence. Despite all studies being randomized controlled trials, only a small proportion were rated as having low risk of bias, while most had some concerns or were judged to be at high risk. In addition, selective publication may have been present, inflating or distorting the observed residual effects. Thus, the observed residual effects should be interpreted with caution. Fourth, most original studies did not systematically record changes in diet, habitual physical activity, sleep, medication use, or nonexercised activity thermogenesis during the detraining period, which constrains the ability to determine the effects attributable to detraining alone. Fifth, this review included only English-language studies, which may introduce language-related selection bias and thereby limit the generalizability of the findings. Sixth, some duration-specific subgroup outcome estimates were based on a few studies, and some were informed by a single trial. Such estimates are less precise and more susceptible to study-specific characteristics; accordingly, interpretation should be particularly cautious. Seventh, as a meta-analysis based on aggregate data, the present study may be subject to the ecological fallacy when exploring heterogeneity in the decay of different outcome measures. Due to the lack of individual participant data, we are unable to precisely isolate the independent effects of baseline characteristics on detraining trajectories. Therefore, caution should be exercised when making mechanistic extrapolations regarding these residual effects. Eighth, a key methodological limitation is that our meta-analysis compared baseline to post-detraining values. Therefore, it primarily indicates whether exercise-induced improvements remain above baseline after the detraining period. However, this approach does not directly quantify the absolute magnitude of physiological loss from the post-intervention peak state, nor does it characterize the time-course of such decline. Consequently, these findings should be interpreted with caution regarding the extent and kinetics of detraining-related deterioration. Finally, meta-analyses focused specifically on detraining are still limited, and universally accepted standards for categorizing detraining duration are lacking. Therefore, our data-driven time boundaries may not be universally applicable.

Future research should involve large-scale randomized controlled trials with longer follow-up and higher methodological quality, with particular attention to detraining effects lasting beyond 6 months. Studies should also systematically monitor lifestyle changes during the detraining period and standardize the measurement of body composition and cardiometabolic outcomes. In addition, subsequent analyses should be stratified by exercise modality, age, sex, baseline health status, and disease type to identify the minimum effective post-detraining dose required to preserve residual effects on body composition and cardiometabolic benefits. Specifically, this should determine whether low-frequency or short-duration exercise (e.g., “exercise snacks”) is sufficient to attenuate the decline in metabolic function and muscle-related adaptations (Islam et al., 2022; Jones et al., 2024). Finally, the phenomenon of delayed improvement in fasting insulin warrants further mechanistic and empirical studies to confirm and elucidate its underlying basis.

## 5 Conclusion

Meta-analyses suggest that residual benefits of structured exercise on body composition and cardiometabolic health may vary in a time-dependent and outcome-specific manner after detraining. Specifically, after long-term detraining, indicators such as body mass, WC, VO_2max_, and SBP appeared to remain improved relative to baseline and relative to control groups. In contrast, measures of glucose and lipid metabolism, as well as muscle-related outcomes, appeared to show less persistent residual benefits relative to baseline.

However, these findings should be interpreted with caution because many included studies had concerns regarding risk of bias, and the certainty of the evidence varied across outcomes. We therefore explicitly note that the results should be viewed as suggestive rather than definitive conclusions, particularly for outcomes with insufficient supporting evidence, limited study numbers, or lower certainty. Accordingly, careful post-detraining monitoring of glycolipid metabolic function and changes in body composition, especially in the short term, may help identify potential risks of metabolic worsening and muscle loss, thereby supporting timely clinical management.

## Data Availability

The datasets presented in this article are not readily available because As a systematic review and meta−analysis, this study did not produce any raw data. All findings are presented within the article and its **Supplementary Materials**. Requests to access the datasets should be directed to Hao Zhang, 18927008729@163.com.
