# Supplementary material for: The effects of detraining on body composition and cardiometabolic health: a systematic review and meta-analysis of randomized controlled trials
Source: Front Physiol. 2026 Jul 22;17:1891899. doi: 10.3389/fphys.2026.1891899 (PMC13437958; doi:10.3389/fphys.2026.1891899)
Supplement: Supplementary file 1 [file DataSheet1.pdf]

Supplementary Information 1

Table of Content

Tables

Supplementary Table 1 Search Strategy pp.2-5

Supplementary Table 2 Characteristics of included studies pp.5-27

Supplementary Table 3 Summary of overall and subgroup meta-analysis results pp.28-32

Supplementary Table 4 GRADE evidence profile pp.33-44

Supplementary Table 1 Note Search strategy

Database	Search strategy
PubMed	((“Exercise”[MeSH] OR “Exercise Therapy”[MeSH] OR “Sports”[MeSH] OR exercis*[tiab] OR train*[tiab] OR “physical activity”[tiab] OR aerobic*[tiab] OR resistance*[tiab] OR strength*[tiab] OR endurance*[tiab] OR yoga[tiab] OR pilates[tiab] OR “Tai Chi”[tiab] OR Taijiquan[tiab] OR swim*[tiab] OR walk*[tiab] OR run*[tiab] OR exergame*[tiab] OR hiking[tiab] OR aquatic*[tiab] OR cycl*[tiab] OR jog*[tiab] OR sport*[tiab] OR stretch*[tiab] OR “weight lift*”[tiab] OR “Ba Duan Jin”[tiab] OR “Yi Jin Jing”[tiab] OR dance*[tiab] OR “Wu Qin Xi”[tiab] OR fitness[tiab]) AND (detrain*[tiab] OR “training cessation”[tiab] OR “training reduction”[tiab] OR “interrupted training”[tiab] OR deconditioning[tiab] OR “training interval”[tiab] OR “follow-up”[tiab]) AND (“Body Composition”[MeSH] OR “Cardiovascular Diseases”[MeSH] OR “body composition”[tiab] OR “body fat”[tiab] OR “body mass index”[tiab] OR BMI[tiab] OR “body fat percentage”[tiab] OR “waist circumference”[tiab] OR “waist-to-hip ratio”[tiab] OR “lean mass”[tiab] OR “muscle mass”[tiab] OR “visceral fat”[tiab] OR “fat-free mass”[tiab] OR “fat mass”[tiab] OR “maximal oxygen uptake”[tiab] OR VO_2max[tiab] OR cardiometabolic*[tiab] OR “cardiorespiratory fitness”[tiab] OR CRF[tiab] OR hypertens*[tiab] OR “blood pressure”[tiab] OR “systolic pressure”[tiab] OR “diastolic pressure”[tiab] OR BP[tiab] OR “heart rate”[tiab] OR HR[tiab] OR “high-density lipoprotein”[tiab] OR HDL[tiab] OR “low-density lipoprotein”[tiab] OR LDL[tiab] OR triglyceride*[tiab] OR cholesterol[tiab] OR “stroke volume”[tiab] OR “glycated hemoglobin”[tiab] OR HbA1c[tiab] OR “C-reactive protein”[tiab] OR CRP[tiab] OR IL-6[tiab] OR “tumor necrosis factor”[tiab] OR TNF*[tiab] OR “left ventricular ejection fraction”[tiab] OR LVEF[tiab] OR glucose[tiab] OR insulin[tiab]) AND (randomized controlled trial[pt] OR controlled clinical trial[pt] OR randomized[tiab]))
Embase	((‘exercise’/exp OR exercis*:ab,ti OR train*:ab,ti OR ‘physical activity’:ab,ti OR aerobic*:ab,ti OR resistance*:ab,ti OR strength*:ab,ti OR endurance*:ab,ti OR yoga:ab,ti OR pilates:ab,ti OR ‘tai chi’:ab,ti OR taijiquan:ab,ti OR swim*:ab,ti OR walk*:ab,ti OR run*:ab,ti OR

	exergame*:ab,ti OR cycl*:ab,ti OR sport*:ab,ti OR ‘weight lift*’:ab,ti OR dance*:ab,ti OR fitness:ab,ti) AND (detrain*:ab,ti OR ‘training cessation’:ab,ti OR ‘training reduction’:ab,ti OR ‘training interval’:ab,ti OR ‘interrupted training’:ab,ti OR deconditioning:ab,ti OR ‘follow-up’:ab,ti) AND (‘body composition’/exp OR ‘cardiovascular disease’/exp OR ‘body composition’:ab,ti OR ‘body fat’:ab,ti OR bmi:ab,ti OR ‘waist circumference’:ab,ti OR ‘lean mass’:ab,ti OR ‘muscle mass’:ab,ti OR ‘maximal oxygen uptake’:ab,ti OR VO_2max:ab,ti OR cardiometabolic*:ab,ti OR ‘cardiorespiratory fitness’:ab,ti OR hypertens*:ab,ti OR ‘blood pressure’:ab,ti OR bp:ab,ti OR ‘heart rate’:ab,ti OR hr:ab,ti OR ‘high density lipoprotein’:ab,ti OR hdl:ab,ti OR ldl:ab,ti OR triglyceride*:ab,ti OR cholesterol:ab,ti OR hba1c:ab,ti OR ‘c reactive protein’:ab,ti OR crp:ab,ti OR ‘il 6’:ab,ti OR ‘tumor necrosis factor’:ab,ti OR tnf*:ab,ti OR lvef:ab,ti OR glucose:ab,ti OR insulin:ab,ti) AND (‘randomized controlled trial’/exp OR ‘controlled clinical trial’/exp OR randomized:ab,ti OR randomly:ab))
Web of Science	TS = ((exercis* OR train* OR “physical activity” OR aerobic* OR resistance* OR strength* OR endurance* OR yoga OR pilates OR “Tai Chi” OR Taijiquan OR swim* OR walk* OR run* OR exergame* OR hiking OR aquatic* OR cycl* OR jog* OR sport* OR stretch* OR “weight lift*” OR “Ba Duan Jin” OR “Yi Jin Jing” OR dance* OR “Wu Qin Xi” OR fitness) AND (detrain* OR “training cessation” OR “training reduction” OR “interrupted training” OR deconditioning OR “training interval” OR “follow-up”) AND (“body composition” OR “body fat” OR “body mass index” OR BMI OR “waist circumference” OR “waist-to-hip ratio” OR “lean mass” OR “muscle mass” OR “visceral fat” OR “fat-free mass” OR “fat mass” OR “maximal oxygen uptake” OR VO_2max OR cardiometabolic OR “cardiorespiratory fitness” OR hypertens* OR “blood pressure” OR BP OR “heart rate” OR HR OR “high-density lipoprotein” OR HDL OR LDL OR triglyceride* OR cholesterol OR “stroke volume” OR “glycated hemoglobin” OR HbA1c OR “C-reactive protein” OR CRP OR IL-6 OR “tumor necrosis factor” OR TNF* OR LVEF OR glucose OR insulin) AND (randomized OR randomised OR randomly OR “controlled trial” OR RCT))

EBSCO（SPORTDiscus, MEDLINE,CINAHL）	((TI (exercis* OR train* OR “physical activity” OR aerobic* OR resistance* OR strength* OR endurance* OR yoga OR “Tai Chi” OR swim* OR walk* OR run* OR cycl* OR sport* OR fitness) OR AB (exercis* OR train* OR “physical activity” OR aerobic* OR resistance* OR strength* OR endurance* OR yoga OR “Tai Chi” OR swim* OR walk* OR run* OR cycl* OR sport* OR fitness)) AND (TI (detrain* OR “training cessation” OR “interrupted training” OR deconditioning OR “follow-up”) OR AB (detrain* OR “training cessation” OR “interrupted training” OR deconditioning OR “follow-up”)) AND (TI (“body composition” OR “body mass index” OR BMI OR “body fat” OR “lean mass” OR VO_2max OR “blood pressure” OR BP OR cardiometabolic OR cholesterol OR HbA1c OR CRP OR glucose OR insulin) OR AB (“body composition” OR “body mass index” OR BMI OR “body fat” OR “lean mass” OR VO_2max OR “blood pressure” OR BP OR cardiometabolic OR cholesterol OR HbA1c OR CRP OR glucose OR insulin)) AND (TI (randomized OR randomly OR trial OR RCT) OR AB (randomized OR randomly OR trial OR RCT)))

Supplementary Table 2 Characteristics of included studies

Study	N (female%)	Population& Age(mean(SD))	Intervention	Exercise frequency & duration	Session length	Comparator	Exercise cessation	Outcomes
Anandavadivelan 2024	206 (100)	Women with early breast cancer; RT-HIIT:52.7 (10.3) AT-HIIT:54.4 (10.3) Usual care:52.6 (10.2)	RT-HIIT; AT-HIIT	2d/wk for 16wk	NR	Usual care	2 years 5 years	Body composition: Body mass (calibrated electric scales); Cardiometabolic health: VO_2peak (submaximal cycle test)
Batrakoulis 2018	35 (100)	Overweight/obese females; Overall:36.4 (4.4)	CINT	3d/wk for 20wk	NR	No intervention	20 weeks	Body composition: Body mass, BMI, Body fat, Fat mass, Fat-free mass (DXA) Waist circumference, WHR; Cardiometabolic health: VO_2max (graded test)
Bertz 2012	33 (100)	Prepregnancy women ; EG:32.2 (4.6) CG:33.2 (3.7)	60-70% HRmax Brisk walk	4d/wk for 12wk	30-45 min/session	Usual care	9 months	Body composition: Body mass, BMI, Fat mass, Muscle mass (DXA)
Bisconti 2020	39 (48.7)	Healthy adults; Overall:23 (2)	PSBil; PSMono	5d/wk for 12wk	PSBil: 40 min/session PSMono:20 min/session	No intervention	6 weeks	Cardiometabolic health: Systolic/Diastolic blood pressure; Resting heart rate (applanation tonometry)
Bjerre 2019	214 (0)	Men with prostate cancer; Overall:68.4 (6.2)	Football training	5d/wk for 24wk	60 min/session	Usual care (PA recommendations)	6 months	Body composition: Fat mass, Lean body mass (DXA)

Study	N (female%)	Population& Age(mean(SD))	Intervention	Exercise frequency & duration	Session length	Comparator	Exercise cessation	Outcomes
Brekke 2014	35 (100)	Prepregnancy women ; Overall:33 (4)	60-70% HR_{max} walks	4d/wk for 12wk	45 min/session	No intervention	9 months	Body composition: Waist circumference (tape measure); Cardiometabolic health: Systolic/Diastolic blood pressure (auscultatory method); Total-C, LDL-C, HDL-C, Triglycerides (laboratory analysis); Fasting glucose/insulin (laboratory analysis)
Brobakken 2025	48 (41)	Schizophrenia spectrum disorders; Overall:35 (11)	High-intensity Aerobic interval training	2d/wk for 52wk	NR	Usual care	4.5 years	Body composition: Body mass, BMI, Waist circumference; Cardiometabolic health: Systolic/Diastolic blood pressure (ProBP 2400, Welch Allyn) Total-C, LDL-C, HDL-C, Triglycerides, Fasting glucose (ADVIA Chemistry XPT system)
Broderick 2013	43 (86)	72.1% Breast cancer, others (colon, lymphoma, etc.); Overall:51.8 (9.2)	Supervised aerobic training + home exercise program;	2d/wk for 8wk	NR	Usual care	4weeks	Body composition: BMI, waist circumference Cardiometabolic health: VO_{2max}(sub-maximal incremental exercise test)
Brooker 2023	100 (76)	Inactive adults with overweight or obesity AMEx:41 (12) PMEx:38 (11) CG:38 (10)	Self-paced aerobic exercise (treadmill-based)	250 min for 12wk	NR	No intervention	3 months, 6 months	Body composition: Body mass(Load Cell Digitizer); Body fat, Fat mass, Fat-free mass(DXA); Cardiometabolic health: VO_2peak (indirect calorimetry)

Study	N (female%)	Population& Age(mean(SD))	Intervention	Exercise frequency & duration	Session length	Comparator	Exercise cessation	Outcomes
Cantarero-Villanueva 2016	40 (35)	Colon cancer survivors; EG:57.5 (8) CG:62.3 (8)	Core stabilization exercises	3d/wk for 8wk	90 min/session	Usual care (lifestyle recommendations)	6 months	Body composition: Body mass, BMI, Body fat, Muscle mass(bioelectrical impedance); Waist circumference(plastic tape measure)
Carei 2010	53 (92.6)	Anorexia nervosa, bulimia nervosa, or eating disorder not otherwise specified patients; Overall:16.52 (2.35)	Individualized Viniyoga	2d/wk for 8wk	60 min/session	Usual care (standard care)	4 weeks	Body composition: BMI
Carter 2013	28 (86.7)	Moderate disability from Multiple Sclerosis; EG:39.5 (6.5) Usual care:40.9 (8.7)	AT+Tailored functional body conditioning exercises	3d/wk for 10wk	NR	Usual care (offered exercise advice	14 weeks	Body composition: BMI, WHR
Carvalho 2009	57 (100)	Healthy older adults; Overall:69(3.5)	Low-intensity MT	2d/wk for 32wk	NR	No intervention	12weeks	Body composition: BMI
Chee Huei 2017	119 (71)	Employees in Putrajaya with metabolic syndrome; Aerobic group: 33.63 (9.84) CG:35.18 (9.19)	Moderate-intensity aerobic exercise	1h/wk for 16wk	NR	No intervention	8 weeks	Body composition: Waist circumference (inelastic tape); Cardiometabolic health: Systolic/Diastolic Blood Pressure (automated monitor); Triglycerides, HDL-C, Fasting glucose (analyzed at Gribbles Pathology)

Study	N (female%)	Population& Age(mean(SD))	Intervention	Exercise frequency & duration	Session length	Comparator	Exercise cessation	Outcomes
Chen 2018	33 (100)	Females with sarcopenia; KT:66.7 (5.3) CG:68.3 (2.8)	KT	2d/wk for 8wk	60 min/session	No intervention	4 weeks	Body composition: Body mass, VFA (InBody 720 analyzer)
Ciccolo 2011	25 (52)	Females smokers Overall:36.5 (12)	Full-body RT exercises	2d/wk for 12wk	60 min/session	View health videos	3 months	Body composition: Body mass
Donyaei 2024	34 (100)	Females with type 2 diabetes and moderate depression; Overall:60.6 (6.3)	Combined aerobic-resistance training.	3d/wk for 12wk	NR	No intervention	8 weeks	Body composition: Body fat; Cardiometabolic health: Fasting glucose (colorimetric enzymatic method) Fasting insulin (ELISA) HOMA-IR
Elliott 2002	15 (100)	Healthy, sedentary, postmenopausal females Overall:53 (3)	Low-intensity RT	3d/wk for 8wk	NR	No intervention	8 weeks	Body composition: Body mass (weighing scales); BMI, WHR; Body fat (tape measure); Cardiometabolic health: Systolic/Diastolic blood pressure, Mean arterial pressure (automatic sphygmomanometer); Total-C, LDL-C, HDL-C, Triglycerides (Monarch Chemistry System)
Fatouros 2004a	19 (0)	Sedentary healthy older adults; EG:71.5 (6.5) CG:72.8 (4.8)	Endurance training	3d/wk for 16wk	12–42 min/session	No intervention	4 months	Body composition: Body mass, Skinfolds; Cardiometabolic health: VO_{2peak} (treadmill test)

Study	N (female%)	Population& Age(mean(SD))	Intervention	Exercise frequency & duration	Session length	Comparator	Exercise cessation	Outcomes
Fatouros 2005b	36 (0)	Sedentary older men; High-intensity: 70.8 (2.8) Moderate-intensity: 69.7 (3.8) Low-intensity: 71.1 (3.6) CG:69.5 (5.1)	High-intensity RT; Moderate-intensity RT; Low-intensity RT	3d/wk for 24wk	NR	No intervention	24 weeks	Body composition: Body mass, BMI, WHR; Skinfold (Harpenden caliper) Cardiometabolic health: VO_{2max} (Modified Bruce protocol, Oxycon Champion system) Fasting glucose (Glucose oxidase method); HOMA-IR
Fernandez-del-Valle 2014	36 (100)	Females with restricting-type anorexia nervosa； EG:12.61 (0.59) CG:13 (0.6)	High-intensity resistance training	3d/wk for 8wk	NR	Usual care (psychological therapy)	4 weeks	Body composition: Body mass, BMI
Frost 2020	99 (54.5)	Cognitively normal, no regular high-intensity exercise people 69.1 (5.2)	High-Intensity exercise; Moderate-Intensity exercise	2d/wk for 6wk	NR	Information session on lifestyle	12 months	Cardiometabolic health: VO_2peak (Highest 15s mean VO_2)
Furzer 2016	37 (NR)	Haematological cancer patients; 48.9 (13.1)	Tailored aerobic + resistance training	3d/wk for 12wk	NR	Usual care	12 weeks	Body composition: Lean mass, Body fat (DXA)

Study	N (female%)	Population& Age(mean(SD))	Intervention	Exercise frequency & duration	Session length	Comparator	Exercise cessation	Outcomes
Gjellesvik 2020	70 (42)	3 months to 5 years post-first stroke patients; Overall:57.6 (9.3)	High-intensity interval training	3d/wk for 8wk	38 min/session	Usual care (standard care)	10 months	Cardiometabolic health: VO_2peak (Graded exercise treadmill test); Systolic/Diastolic blood pressure (Automatic device); Total-C, LDL-C, HDL-C, Triglycerides (12-h fasting blood test)
Gram 2010	68 (45.6)	Type 2 diabetes patients; NW:62 (10) EP:59 (10) CG:61 (10)	Outdoor moderate-intensity walk; Indoor exercise	1st 2 mo: 2d/wk; 2nd 2 mo: 1d/wk for 4 mo	45 min/session	Usual care (standard exercise information)	8 months	Body composition: Body mass (Tanita BW800A scale),BMI;Waist circumference (tape measure); Cardiometabolic health: Systolic/Diastolic blood pressure (Omron sphygmomanometer); Total-C, LDL-C, HDL-C (standard methods)
Haganes 2025	66 (100)	BMI≥27 kg/m² females; Overall:36.9 (6)	HIIT	3d/wk for 7wk	NR	No intervention	97 weeks	Body composition: Body mass, Fat mass, Muscle mass (bioelectrical impedance InBody720); Cardiometabolic health: Systolic/Diastolic blood pressure Total-C, LDL-C, HDL-C, Triglycerides, Fasting glucose (Siemens Atellica CH930); Resting heart rate (Philips IntelliVue MP50); VO_2peak (indirect calorimetry MetaMax II)

Study	N (female%)	Population& Age(mean(SD))	Intervention	Exercise frequency & duration	Session length	Comparator	Exercise cessation	Outcomes
Hamdorf 1993	66 (100)	Healthy older adults;<br>EG:64.8 (2.5)<br>CG:64.4 (2.5)	Progressive walking program	2d/wk<br>for 36wk	NR	No intervention	6 months	Body composition:<br>Body mass (wheelchair scale);<br>Cardiometabolic health:<br>Resting heart rate (Electrocardiogram)
Hansen 2024	17 (41.1)	Wheelchair-dependent individuals with Spinal Cord Injury;<br>EG:50 (4)<br>CG:50 (12)	UBROW exercise	3d/wk<br>for 12wk	30 min/session	No intervention	6 months	Body composition:<br>Body mass (wheelchair scale);<br>Waist circumference (non-elastic tape);<br>Cardiometabolic health:<br>Systolic/Diastolic blood pressure (Random zero sphygmomanometer)<br>Total-C, LDL-C, HDL-C, Triglycerides, Fasting glucose (Cobas 8000);<br>Fasting insulin (Cobas 600e)
Hao 2019	113 (46)	Rural 9-12-year-old overweight/obese children<br>Overall: NR	Rope skipping	5d/wk<br>for 8wk	30 min/session	No intervention	10 months	Body composition:<br>BMI
Herring 2017	24 (91.7)	Post-bariatric surgery 12-24 months females;<br>Overall:48.4 (8.9)	Moderate-intensity aerobic training	3d/wk<br>for 12wk	60 min /session	Usual care	12 weeks	Body composition:<br>Body mass, BMI, Body fat, Fat mass (Tanita BC-418-MA scale);<br>Waist circumference<br>Cardiometabolic health:<br>Systolic/Diastolic blood pressure (Omron M7 monitor);<br>Resting heart rate (Contec pulse oximeter)

Study	N (female%)	Population& Age(mean(SD))	Intervention	Exercise frequency & duration	Session length	Comparator	Exercise cessation	Outcomes
Islam 2019	68 (77.5)	Healthy young adults; Overall:21 (3)	VICT; Tabata exercise	4d/wk for 4wk	VICT: 30 min /session; Tabata: 4 min/session	No intervention	2 months	Cardiometabolic health: VO_{2peak} (treadmill ramp test, 30s average)
Kagioglou 2021	22 (52)	Precapillary pulmonary hypertension; Overall:53.9 (13.8)	Combined aerobic + strength training	3d/wk for 6wk	NR	No intervention	3 weeks	Cardiometabolic health: VO_{2peak} (Cardiopulmonary exercise test)
Kemmler 2020	43 (0)	community-dwelling with osteosarcopenia; HIT-RT:79.2 (4.2) CG:77.8 (3.6)	HIT-RT	2d/wk for 18wk	NR	No intervention	6 months	Body composition: BMI
Kim. H 2015	66 (100)	≥75 y frail Japanese women EG:81.1 (2.8) CG:80.3 (3.3)	Strengthening + balance/gait training.	2d/wk for 12wk	60 min/session	Usual care (placebo)	4 weeks	Body composition: Muscle mass (DXA)
Kim 2011	127 (100)	community-dwelling elderly Japanese women with stress, urge, and mixed urinary incontinence; EG:76.1 (4.3) CG:75.7 (4.4)	PFM exercise	2d/wk for 12wk	NR	Usual care (education classes)	7 months	Body composition: Body mass, BMI, Waist circumference (tape measure)
Kindred 2019	46 (56.5)	Non-Hispanic white (97.8%); 82.6% received chemotherapy; 30.4% stage 3 cancer; Overall:57.28 (9.73)	Home-based moderate-intensity physical activity	2-5d/wk for 12wk	10-30 min/session	Usual care (weekly phone calls)	3 months 6 months	Body composition: Fat mass (Bioelectrical impedance analysis); Cardiometabolic health: VO_{2}peak (Treadmill walk test)

Study	N (female%)	Population& Age(mean(SD))	Intervention	Exercise frequency & duration	Session length	Comparator	Exercise cessation	Outcomes
Knols 2011	131 (41.2)	Hematopoietic stem-cell transplantation; Overall:46.7 (12.8)	PE program	2d/wk for 12wk	NR	Usual care	12 weeks	Body composition: Body mass, Fat mass, Lean body mass (DXA)
Kranen 2023	19 (0)	Healthy adolescents; Overall:13.3 (0.5)	High-intensity HIIT running	3d/wk for 4wk	NR	No intervention	2 weeks	Body composition: BMI, Fat mass, Fat-free mass, Body fat (BodPod); Cardiometabolic health: Total-C, LDL-C, HDL-C, Triglycerides (CardioChek PA); Fasting glucose (YSI 2900D Biochemistry Analyzer) Fasting insulin (Enzyme immunoassay)
Krawcyk 2023	71 (19)	Lacunar stroke; Overall:63.9 (8.8)	Home-based HIIT	5d/wk for 12wk	15 min/session	Usual care (medication)	6 months 12 months	Body composition: BMI; Cardiometabolic health: Systolic/Diastolic blood pressure (automatic monitor)
Lee 2021	27 (100)	Postmenopausal with osteosarcopenic adiposity; EG:70.13 (4.41) CG:71.82 (5.23)	Progressive elastic band resistance	3d/wk for 12wk	50 min/session	Usual care (general education classes)	6 months	Body composition: Body fat (DXA)
Li 2015	206 (82.5)	Healthy freshmen or sophomores; Overall:20.78 (1.1)	Baduanjin exercise	5d/wk for 12wk	60 min/d	No intervention	12 weeks	Cardiometabolic health: Systolic/Diastolic blood pressure (sphygmomanometer); Resting heart rate (heart rate monitor)

Study	N (female%)	Population& Age(mean(SD))	Intervention	Exercise frequency & duration	Session length	Comparator	Exercise cessation	Outcomes
Liu 2023	20 (100)	Eumenorrheic, untrained females; HIIT:23.0 (1.2) CG:22.4 (0.7)	HIIT	3d/wk for 4wk	NR	No intervention	2 weeks	Body composition: Body mass, Body fat, BMI (bioelectrical impedance analysis Inbody720); Cardiometabolic health: VO_2max (ramp-incremental test, metabolic cart)
Lo 2011	30 (0)	Healthy college-aged men; Overall:20.4 (1.36)	Resistance training	3d/wk for 24wk	NR	No intervention	24 weeks	Body composition: Body mass, Body fat, BMI Fat mass, Lean body mass (DXA); WHR (Flexible inelastic tape)
Lovell 2011	24 (0)	Healthy men aged EG:70.2 (0.8) CG:73.7 (1)	Lower limb strength training	3d/wk for 16wk	NR	No intervention	4 weeks	Body composition: Body mass, Body fat, Lean body mass (DXA); Cardiometabolic health: VO_2max (Incremental exercise test)
Malandish 2020	26 (100)	Healthy sedentary women, ⩾1 year postmenopausal; EG:53.36 (3.98) CG:53 (3.26)	Moderate-intensity aerobic training	3d/wk for 12wk	1h/session	No intervention	5 months	Cardiometabolic health: Resting heart rate (electrocardiogram)
Martins 2010	45 (60)	hypertension (47%), diabetes mellitus (16%), angina pectoris (15%); ST:73.2 (6.5) AT:76.2 (7.4) CG:81.2 (7.9)	Strength training; Aerobic training	3d/wk for 16wk	45 min/session	No intervention	16 weeks	Body composition: Body mass (Seca 770 scale); BMI, Waist circumference(Tape); Cardiometabolic health: Total-C, LDL-C, HDL-C, Triglycerides (Randox direct enzymatic assays);

Study	N (female%)	Population& Age(mean(SD))	Intervention	Exercise frequency & duration	Session length	Comparator	Exercise cessation	Outcomes
Miyachi 2004	28 (0)	Healthy man; Overall:22 (1)	Resistance training	3d/wk for 16wk	45 min/session	No intervention	4 months	Cardiometabolic health: Systolic/Diastolic blood pressure (Semiautomated device); Resting heart rate
Moghadasi 2012	16 (0)	Sedentary overweight/obese middle-aged men; Overall:41.18 (6.1)	High-intensity endurance training	4d/wk for 2wk	45 min/session	No intervention	1 week	Body composition: Body mass, Fat mass, Body fat, Lean body mass (bioelectrical impedance analysis); WHR, BMI; Cardiometabolic health: VO_2max (modified Bruce protocol); Fasting glucose (enzymatic colorimetric method)； Fasting insulin (radioimmunoassay)； HOMA-IR (homeostasis model assessment)
Mundbjerg 2018	60 (70)	30% with T2D Eligible for Roux-en-Y gastric bypass; Overall:42.3 (9.1)	Combined endurance + resistance training	2d/wk for 26wk	40 min/session	Usual care (physical activity information)	12 months	Body composition: Body mass, BMI
Nemoto 2025	60 (55)	Treated hypertensive patients ; Overall:66.9 (10.5)	Low-intensity IHG training	⩾3d/wk for 12wk	12 min/session	No intervention	12 weeks	Body composition: Body fat (InBody 770); Cardiometabolic health: Systolic/Diastolic blood pressure, Resting heart rate (HEM-8713 device); Total-C, LDL-C, HDL-C

Study	N (female%)	Population& Age(mean(SD))	Intervention	Exercise frequency & duration	Session length	Comparator	Exercise cessation	Outcomes
Ng 2015	98 (44)	community-dwelling prefrail/frail older adults;<br>EG:70.3 (5.25)<br>CG:70.1 (5.02)	First 12 wk: group strength/balance training; next 12 wk: home-based exercises	2d/wk for 24wk	90 min/session	Usual care (placebo)	6 months	Body composition:<br>BMI
Nikseresht 2016	33 (0)	Middle-aged obese men, sedentary,<br>NRT:40.4 (5.2)<br>AIT:39.6 (3.7)<br>CG:38.9 (4.1)	Nonlinear resistance training;<br>Treadmill interval training	3d/wk for 12wk	NRT:55 min/session<br>AIT:16 min/session	No intervention	4 weeks	Body composition:<br>Body mass (Seca 700 scale);<br>Body fat (Lange skinfold caliper, Jackson-Pollock equation);<br>Waist circumference (tape measure); WHR;<br>Cardiometabolic health:<br>VO_2peak (Modified Bruce treadmill protocol)<br>Total-C, LDL-C, HDL-C, Triglycerides, Fasting glucose (AutoChemistry Analyzer)
Niño 2017	29 (0)	Healthy, physically active adults;<br>Overall:21.8 (2.3)	ST;AT;Combind AT+ST	3d/wk for 6wk	60 min/session	No intervention	3 weeks	Cardiometabolic health:<br>VO_{2max} (Maximal test)<br>Resting heart rate(ECG)
Nogueira 2017	311 (45)	Apparently healthy prepubertal and peripubertal children;<br>Overall:10 (0.6)	High-intensity aerobic and impact exercises	3d/wk for 9wk	10 min/session	No intervention	12 months	Body composition:<br>Body mass (digital scales); BMI;<br>Waist circumference (Anthropometric tape measure);<br>Cardiometabolic health:<br>Resting heart rate (Stethoscope, radial pulse); VO_2max (20-m shuttle run test)

Study	N (female%)	Population& Age(mean(SD))	Intervention	Exercise frequency & duration	Session length	Comparator	Exercise cessation	Outcomes
Nordén 2024	60 (57)	Patients with inflammatory joint disease; Overall:58 (8.15)	HIIT + self-guided moderate exercise	3d/wk for 12wk	NR	Usual care	3 months	Body composition: BMI, Fat-free mass; Cardiometabolic health: VO_2peak (cardiopulmonary exercise test); Systolic/Diastolic blood pressure (Ambulatory blood pressure monitor); Total-C, LDL-C, HDL-C, Triglycerides (bioelectrical impedance analysis); Resting heart rate
Nuri 2016	30 (0)	Colorectal cancer patients; Overall:51.56 (11.28)	Aerobic training	3d/wk for 8wk	45 min/session	No intervention	1 week	Body composition: Body mass, BMI, Body fat (bioelectrical impedance analysis); Cardiometabolic health: VO_2peak (rockport walking test); Fasting glucose (photometric method); Fasting insulin (ELISA kit); HOMA-IR (homeostasis model assessment)
O'Connor 2017	60 (41)	Kidney transplant recipients; Overall:51.8 (12.5)	Moderate-intensity aerobic training + resistance training	3d/wk for 12wk	NR	Usual care (exercise encouragement)	9 months	Body composition: Body mass (calibrated stadiometer); Cardiometabolic health: VO_2peak (recumbent cycling incremental); Systolic/Diastolic blood pressure (automated sphygmomanometer)

Study	N (female%)	Population& Age(mean(SD))	Intervention	Exercise frequency & duration	Session length	Comparator	Exercise cessation	Outcomes
Øiestad 2023	162 (50.9)	Symptomatic knee osteoarthritis; Strength exercise: 57.6 (6.6) Aerobic exercise : 57.3 (7.1) UC:57.8 (7.4)	Strength exercise; Aerobic exercise	2-3d/wk for 12wk	NR	No intervention	8 months	Cardiometabolic health: VO_2max (Monark 839E cycle ergometer)
Okamoto 2007	33 (80)	Healthy people without regular physical exercise; 18.8 (0.1)	BRT;ART	2d/wk for 8wk	NR	No intervention	4 weeks	Body composition: Body mass, BMI, Body fat; Cardiometabolic health: Systolic/Diastolic blood pressure, Mean arterial pressure (Automatic oscillometric device); Resting heart rate (Polar RS100)
Oliveira 2019	29 (0)	Healthy adolescents EG:13.3 (0.5) CG:13.2 (0.5)	HIIE	3d/wk for 4wk	NR	No intervention	2 weeks	Body composition: Body mass, Body fat; Cardiometabolic health: Systolic/Diastolic blood pressure (Finometer PRO); Resting heart rate (3-lead ECG)
Opdenacker 2011	120 (48)	Sedentary but healthy older adults; EG:70 (4.3) CG:67.9 (5.3)	Endurance + strength + posture/balance training	3d/wk for 11wk	60-90 min/session	No intervention	12 months	Body composition: BMI, Body fat (bioelectrical impedance analysis); Waist circumference (metal tape) Cardiometabolic health: VO_2peak (Cycle ergometer + breath-by-breath gas analysis) Systolic/Diastolic blood pressure (Automatic manometer）

Study	N (female%)	Population& Age(mean(SD))	Intervention	Exercise frequency & duration	Session length	Comparator	Exercise cessation	Outcomes
Prasertsri 2019	50 (78)	Sedentary older adults with prehypertension; EG:67.24 (5.29) CG:67.32 (6.89)	Low-intensity ASE	3d/wk for 12wk	30 min/session	No intervention	1 month	Cardiometabolic health: Systolic/Diastolic blood pressure, Mean Arterial Pressure (Digital automatic BP monitor); Resting heart rate (digital automatic BP monitor); Total-C, LDL-C, HDL-C, Triglycerides (Accelerator selective detergent/Enzymatic method); Fasting glucose (Enzymatic method)
Rosado 2022	56 (75)	Community-dwelling older adults at risk of falling; EG1:74.3 (5.4) EG2:74.7 (5.5) CG:75.9 (5.7)	Psychomotor intervention; Psychomotor intervention + whole-body vibration training	3d/wk for 24wk	75 min/session	No intervention	12 weeks	Body composition: Body mass, Fat mass, Lean body mass(DXA)
Rosety-Rodriguez 2014	34 (100)	Premenopausal obese woman with Down syndrome; EG:24.7 (3.6) CG:25.1 (3.9)	Aerobic training	3d/wk for 10wk	45-65 min/session	No intervention	1 month, 3 months, 6 months	Body composition: Fat mass (bioelectrical impedance analysis); Waist circumference; Cardiometabolic health: VO_2max (treadmill incremental test)

Study	N (female%)	Population& Age(mean(SD))	Intervention	Exercise frequency & duration	Session length	Comparator	Exercise cessation	Outcomes
Rydwik 2008	46 (0)	Overweight/obese<br>EG:83.5 (3.7)<br>CG:83.1 (4.5）	Aerobic + strength + functional training	2d/wk for 12wk	60 min/session	Usual care (lifestyle advice)	6 months	Body composition:<br>Body mass, Fat-free mass
Sosa 2012a	22 (45)	Children with cystic fibrosis;<br>EG:11 (3)<br>CG:10 (2)	Aerobic + strength exercises	3d/wk for 8wk	NR	Usual care (standard therapy, physiotherapy)	4 weeks	Body composition:<br>Body mass (Balance scale); BMI; Fat-free mass, Body fat (Harpenden caliper)
Sosa 2014b	20 (40)	Paediatric outpatients with cystic fibrosis;<br>EG:11 (1)<br>CG:10 (1)	In-hospital Combined training: IMT + AT	3d/wk for 8wk	NR	Usual care (standard therapy, physiotherapy)	4 weeks	Body composition:<br>Body mass (Balance scale); BMI; Body fat, Fat mass, Fat-free mass (Harpenden caliper);<br>Cardiometabolic health:<br>VO_2peak (Finger pulse oximeter for SpO2)
Schranz 2013	56 (0)	Overweight/obese males;<br>EG:11 (1)<br>CG:10 (1)	Resistance training	3d/wk for 6mo	75 min/session	No intervention	6 months	Body composition:<br>Body mass, BMI (scale);<br>Skinfolds (Harpenden calipers);<br>Body fat, Lean body mass (DXA)

Study	N (female%)	Population& Age(mean(SD))	Intervention	Exercise frequency & duration	Session length	Comparator	Exercise cessation	Outcomes
Shokri 2023	30 (100)	Girls with CPP<br>EG:8.42 (0.75)<br>CG:8.28 (0.64)	Aerobic exercise	3d/wk for 12wk	50 min/session	Usual care	4 weeks	Body composition:<br>Body mass (Beurer GS20); BMI, Waist circumference, Fat mass (Dezenberg formula)
Shokri 2021	30 (100)	Girls with CPP<br>EG:8.42 (0.75)<br>CG:8.28 (0.64)	Combined AT +RT	3d/wk for 12wk	60 min/session	Usual care	4 weeks	Body composition:<br>Body mass (digital scale); BMI; Body fat(Cortés-Castell formula)<br>Cardiometabolic health:<br>VO_2peak (6-Minute Walk Test); Systolic/Diastolic blood pressure (barometer); Total-C, LDL-C, HDL-C, Triglycerides (Enzymatic procedures);
Sloan 2018a	119 (53)	Healthy sedentary young adults;<br>Overall:31 (6)	Aerobic Training	4d/wk for 12wk	40-55 min/session	Waitlist	4 weeks	Body composition:<br>Fat-free mass (Tanita BF-350 monitor);<br>Cardiometabolic health:<br>VO_2max (CPET on Ergoline 800S)
Sloan 2021b	119 (53)	Healthy sedentary young adults;<br>Overall:31 (6)	Aerobic Training	4d/wk for 12wk	40-55 min/session	Waitlist	4 weeks	Cardiometabolic health:<br>Resting heart rate (ECG);<br>Systolic/Diastolic blood pressure (Nexfin blood pressure cuff)

Study	N (female%)	Population& Age(mean(SD))	Intervention	Exercise frequency & duration	Session length	Comparator	Exercise cessation	Outcomes
Stebbings 2013	21 (48)	Healthy adults;<br>EG:19 (3)<br>CG:23 (2.4)	Resistance Training	3d/wk<br>for 8wk	NR	No intervention	2 weeks,<br>4 weeks	Cardiometabolic health:<br>Resting heart rate (Doppler ultrasound waveforms average)
Stener-Victorin 2012	45 (100)	Women with PCOS;<br>18-38 y<br>Overall: NR	Aerobic exercise	≥3d/wk<br>for 8wk	NR	No intervention	16 weeks	Body composition:<br>Body mass;<br>Cardiometabolic health:<br>Resting heart rate (Simultaneous recording);<br>Systolic/Diastolic blood pressure (Omron M5-1 monitor)
Stergioulas 2006	38 (0)	Healthy sedentary boys;<br>EG:12 (0.7)<br>CG:12.3 (0.8)	Bicycle ergometer exercise	4d/wk<br>for 8wk	60 min/session	No intervention	4 weeks	Body composition:<br>Body mass;<br>Cardiometabolic health:<br>Total-C, HDL-C, Triglycerides (Technicon R-XT autoanalyzer, enzymatic technique);
Teixeira 2023	39 (0)	Males with treated arterial hypertension;<br>EG:48 (8)<br>CG:51 (6)	Recreational Futsal	2-3d/wk<br>for 3 mo	60 min/session	Usual care	1 month	Body composition:<br>Body mass (bioelectrical impedance scale)<br>Body fat, BMI;<br>Cardiometabolic health:<br>VO_2peak (Cycle ergometer);<br>Systolic/Diastolic blood pressure<br>Mean arterial pressure (Automated monitor);<br>Resting heart rate;<br>Total-C, HDL-C, LDL-C, Triglycerides (Variant II analyzer)

Study	N (female%)	Population& Age(mean(SD))	Intervention	Exercise frequency & duration	Session length	Comparator	Exercise cessation	Outcomes
Theodorou 2016	56 (0)	Elderly individuals diagnosed with coronary artery disease; AT:61 (7) RT:62 (8) Combined:64 (6) CG:64 (8)	Aerobic;Resistance; Combined exercise	3d/wk for 8wk	NR	No intervention	1 month, 2 months, 3 months	Body composition: Body mass, BMI; Body fat (Harpenden caliper for 7-site skinfold thickness); Cardiometabolic health: Total-C, HDL-C, LDL-C, Triglycerides (Cobas Integra Plus 400 chemistry analyser);
Thompson 2010	41 (0)	Sedentary middle-aged men; EG:54 (5) CG:52 (4）	Progressive exercise	1d/wk for 24wk	NR	No intervention	2 weeks	Body composition: Body mass,BMI(Standard measurement); Cardiometabolic health: VO_2max (treadmill progressive incline test); Systolic/Diastolic blood pressure Total-C, HDL-C, Triglycerides Fasting glucose, Fasting insulin ,HOMA-IR(Commercial kits)
Vainshelboim 2016	34 (34)	Idiopathic Pulmonary Fibrosis; Overall:68 (8)	Aerobic, resistance, flexibility, deep breathing exercises + home-based walking	2d/wk for 24wk	60 min/session	Usual care (medication)	8 months	Body composition: Body mass; Body fat (Lange skin-fold caliper); Waist circumference (Standard fiber glass centimeter)
Waller 2017	87 (100)	Post-menopausal women with mild knee OA AT:63.8 (2.4) CG:63.9 (2.4)	High-intensity aquatic resistance training	3d/wk for 16wk	60 min/session	No intervention	12 weeks	Body composition: Body mass, BMI, Lean body mass, Fat mass(DXA)

Study	N (female%)	Population& Age(mean(SD))	Intervention	Exercise frequency & duration	Session length	Comparator	Exercise cessation	Outcomes
Wang 2022	30 (73)	Overweight/obese children; Overall:14.17 (0.45)	Aerobic games + resistance training	2d/wk for 12wk	60 min/session	No intervention	12 weeks	Body composition: Body mass, BMI, Body fat (TANITA scale/SECA height gauge); Waist circumference, WHR (Flexible metre ribbon); Cardiometabolic health: Systolic/Diastolic blood pressure (Omron monitor)
Winn 2019	616 (45.8)	Healthy and asthmatic adolescents; Overall:13 (1.1)	School-based HIIT	3d/wk for 12mo	30 min/session	No intervention	3 months	Body composition: Body mass, BMI
Zech 2012	70 (70)	Prefrail older adults; Overall:77.6 (6.1)	Low-intensity RT; High-intensity RT	2d/wk for 12wk	NR	No intervention	12 weeks, 24 weeks	Body composition: Body mass, BMI
Zhang 2017	220 (71)	Obese adults with NAFLD; VME:53.2(7.1) ME:54.4(7.4) CG:54(6.8)	Vigorous exercise + moderate exercise; Moderate exercise	NR for 12 mo	NR	Health education	12 months	Body composition: Body mass,Body fat(Body fat analyzer),Waist circumference (Tape measure) Cardiometabolic health: Systolic/Diastolic blood pressure (Blood pressure monitor Visceral)
Zhao 2024	60 (100)	Overweight/obese (BMI≥24 kg/m²), stage 0-III breast cancer survivors, completed adjuvant therapy ≥1 month; EG:47.23 (6.03) CG:48.37 (6.40)	Home-based exercise (aerobic+resistance training).	NR for 12wek	NR	Exercise information	3 months	Body composition: Body mass (weighing machine);BMI;Waist circumference (Tape measure); Body fat, Fat-free mass (Fat caliper + Fat Calculator Software)

Study	N (female%)	Population& Age(mean(SD))	Intervention	Exercise frequency & duration	Session length	Comparator	Exercise cessation	Outcomes
Zheng 2019	170 (64)	High risk of ischaemic stroke, no regular exercise for ⩾ 1 year,<br>54.1% (Baduanjin) and 50.6% (Control) with hypertension;<br>EG:59.75 (6.34)<br>CG:60.53 (6.29)	Baduanjin training	5d/wk for 12wk	60 min/session	No intervention	12 weeks	Body composition:<br>BMI; Waist circumference (Metric ruler);WHR;<br>Cardiometabolic health:<br>Systolic/Diastolic blood pressure (manual measurement);<br>Resting heart rate;<br>Total-C, HDL-C, LDL-C, Triglycerides (Automatic analyser);<br>Fasting glucose (Automatic analyser)

AMEx	Morning exercise
ART	Running after resistance training group
ASE	Arm swing exercise
AT	Aerobic training
AT-HIIT	Moderate-Intensity Aerobic Training combined with High-Intensity Interval Training
BMI	Body Mass Index
BRT	Running before resistance training group
CG	Control group
CINT	Circuit-integrated neuromuscular training
CPP	Central Precocious Puberty
d	Days
DXA	Dual-energy X-ray absorptiometry
ECG	Electrocardiogram
EG	Exercise group
ELISA	Enzyme-linked immunosorbent assay
EP	Exercise on Prescription
HDL-C	High-Density Lipoprotein Cholesterol
HIIE	High-intensity interval exercise
HIT-RT	High-intensity resistance training
HOMA-IR	Homeostatic Model Assessment of Insulin Resistance
HR_{max}	Maximal heart rate
IHG	Lower intensity isometric handgrip
IMT	Inspiratory muscle training
KT	Kettlebell training
LDL-C	Low-Density Lipoprotein Cholesterol
mo	Months
NAFLD	Nonalcoholic fatty liver disease
NRT	Nonlinear resistance training
NW	Nordic walking
OA	Osteoarthritis
PFM	Pelvic floor muscle
PMEx	Evening exercise
PSBil	Bilateral passive static stretching
PSMono	Monolateral passive static stretching

RF	Recreational Futsal
RT	Resistance Training
RT-HIIT	Resistance Training combined with High-Intensity Interval Training
SED	Sedentary group
Total-C	Total cholesterol
UBROW	Upper-body rowing exercise
VICT	Vigorous-intensity continuous training
VME	Vigorous-moderate exercise
VO_2max	Max Oxygen Uptake
VO_2peak	Peak Oxygen Uptake
WHR	Waist-hip ratio

Supplementary Table 3 Summary of overall and subgroup meta-analysis results

Follow-up duration	No. of RCTs	Pre to post ES [95% CI]	*p*	Sample size	I^2	Pre to follow-up ES [95% CI]	*p*	Sample size	I^2	*P* (bet)
**Body mass (kg) (MD)**										
≤ 2months	20	-2.97 [-3.78 to -2.16]	< 0.001	536	0	-2.09 [-3.00 to -1.19]	< 0.001	526	0	0.17
2-6months	28	-1.46 [-1.86 to -1.05]	< 0.001	1347	0	-1.14 [-1.66 to -0.62]	< 0.001	1238	0	
> 6months	16	-1.58 [-2.18 to -0.98]	< 0.001	1408	0	-1.12 [-1.83 to -0.40]	0.002	1157	0	
**BMI (MD)**										
≤ 2months	16	-0.66 [-1.21 to -0.12]	0.02	480	0.28	-0.62 [-1.03 to -0.21]	0.003	469	0.12	0.38
2-6months	25	-0.50 [-0.68 to -0.32]	< 0.001	1385	0	-0.42 [-0.63 to -0.21]	< 0.001	1351	0	
> 6months	12	-0.53 [-1.02 to -0.03]	0.04	999	0.32	-0.20 [-0.64 to 0.23]	0.37	869	0	
**Body fat (%) (MD)**										
≤ 2months	16	-2.12 [-2.91 to -1.34]	< 0.001	389	0	-1.30 [-2.03 to -0.56]	< 0.001	384	0	0.04
2-6months	16	-0.85 [-1.23 to -0.47]	< 0.001	645	0.12	-0.56 [-1.13 to 0.01]	0.05	565	0.16	
> 6months	4	-1.05 [-2.65 to 0.54]	0.20	362	0.89	-0.13 [-0.67 to 0.42]	0.65	332	0	
**Fat mass (kg) (MD)**										
≤ 2months	6	-1.47 [-2.71 to -0.24]	0.02	140	0	-1.49 [-2.27 to -0.71]	< 0.001	135	0	0.06
2-6months	10	-1.36 [-2.22 to -0.50]	0.002	645	0.67	-0.73 [-1.30 to -0.17]	0.01	581	0.15	
> 6months	4	-0.59 [-2.03 to 0.86]	0.43	203	0	0.61 [-1.05 to 2.28]	0.47	172	0	
**Waist circumference (cm) (MD)**										
≤ 2months	6	-3.05 [-4.53 to -1.57]	< 0.001	250	0.16	-2.17 [-3.55 to -0.79]	0.002	241	0	0.65
2-6months	13	-2.50 [-3.82 to -1.18]	< 0.001	523	0.75	-1.43 [-2.40 to -0.46]	0.004	499	0.41	
> 6months	10	-1.74 [-2.53 to -0.95]	< 0.001	928	0	-1.89 [-2.75 to -1.04]	< 0.001	815	0	

**Waist-to-Hip Ratio (MD)**										
≤ 2months	4	-0.03 [-0.06 to -0.01]	0.02	65	0	-0.02 [-0.05 to 0.01]	0.12	65	0	0.50
2-6months	9	-0.01 [-0.01 to -0.00]	0.04	340	0	-0.01 [-0.02 to 0.00]	0.08	338	0	
Overall	13	-0.02 [-0.03 to -0.00]	0.004	405	0.15	-0.01 [-0.02 to -0.00]	0.02	403	0	
**Skinfold (cm) (MD)**										
2-6months	5	-4.44 [-10.06 to 1.17]	0.12	117	0	-1.74 [-7.80 to 4.31]	0.57	103	0	/
**Fat-free mass (kg) (MD)**										
≤ 2months	4	1.34 [-1.54 to 4.21]	0.36	162	0	1.52 [-1.16 to 4.20]	0.27	152	0	0.09
2-6months	7	-0.65 [-1.09 to -0.21]	0.004	367	0	-0.83 [-1.36 to -0.30]	0.002	297	0	
**Muscle mass (kg) (MD)**										
≤ 2months	1	0.06 [-0.60 to 0.73]	0.85	35	0	0.47 [-5.18 to 6.12]	0.87	35	/	0.12
2-6months	1	0.39 [-0.23 to 1.02]	0.22	40	0	0.90 [-0.71 to 2.51]	0.27	31	/	
> 6months	2	-0.03 [-0.45 to 0.39]	0.89	86	0	-1.06 [-2.04 to 0.08]	0.03	51	0	
Overall	4	0.09 [-0.22 to 0.40]	0.55	161	0	-0.45 [-1.67 to 0.76]	0.62	117	0.37	
**Lean body mass (kg) (MD)**										
≤ 2months	2	0.03 [-3.99 to 4.05]	0.99	40	0	1.19 [-2.90 to 5.27]	0.57	40	0	0.80
2-6months	8	0.23 [-0.33 to 0.80]	0.42	482	0.30	-0.18 [-0.59 to 0.23]	0.38	450	0	
> 6months	1	0.00 [-1.78 to 1.78]	1.00	84	0	0.00 [-1.86 to 1.86]	1.00	76	0	
Overall	11	0.06 [-0.31 to 0.43]	0.75	606	0.01	-0.16 [-0.55 to 0.24]	0.43	566	0	
**Resting heart rate (beats/min) (MD)**										
≤ 2months	14	-3.86 [-7.19 to -0.52]	0.02	373	0.71	0.71 [-0.84 to 2.26]	0.37	365	0	0.13
2-6months	10	-2.33 [-4.32 to -0.34]	0.02	691	0.39	-1.46 [-2.96 to 0.05]	0.06	691	0	
> 6months	2	-4.96[-6.00 to -3.92]	< 0.001	366	0	-4.10 [-18.47 to 10.28]	0.58	261	0.93	
**Systolic blood pressure (mmHg) (MD)**										
≤ 2months	12	-3.11 [-5.40 to -0.82]	0.008	471	0.21	-2.34 [-4.41 to -0.28]	0.03	459	0	0.91

2-6months	11	-2.78 [-6.38 to 0.81]	0.13	715	0.67	-1.89 [-4.98 to 1.20]	0.23	715	0.54	
> 6months	12	-3.42 [-6.04 to -0.80]	0.01	686	0.45	-2.69 [-4.73 to -0.66]	0.009	606	0	
**Diastolic blood pressure (mmHg) (MD)**										
≤ 2months	12	-2.71 [-4.27 to -1.23]	< 0.001	471	0.08	-1.59 [-3.17 to -0.01]	0.05	459	0.31	
2-6months	11	-1.92 [-4.16 to 0.32]	0.09	715	0.54	-0.87 [-3.29 to 1.56]	0.48	715	0.62	0.81
> 6months	12	-0.85 [-2.66 to 0.95]	0.35	686	0.40	-1.93 [-4.12 to 0.26]	0.08	606	0.48	
**Mean arterial pressure (mmHg) (MD)**										
≤ 2months	7	-3.35 [-6.18 to -0.52]	0.02	153	0.17	-3.23 [-5.70 to -0.77]	0.01	152	0	/
**VO_2peak (ml/kg/min) (SMD)**										
≤ 2months	9	0.68 [0.33 to 1.03]	< 0.001	242	0.36	0.49 [0.13 to 0.81]	0.009	229	0.37	
2-6months	6	1.04 [0.65 to 1.44]	< 0.001	306	0.52	0.37 [0.10 to 0.64]	0.007	248	0	0.11
> 6months	11	0.58 [0.14 to 0.75]	< 0.001	827	0.21	0.13 [-0.06 to 0.32]	0.12	642	0.40	
**VO_2max (ml/kg/min) (SMD)**										
≤ 2months	10	0.73 [0.48 to 0.97]	< 0.001	297	0	0.60 [0.36 to 0.84]	< 0.001	296	0	
2-6months	5	0.67 [0.23 to 1.12]	0.003	124	0.12	0.42 [0.03 to 0.81]	0.04	124	0	0.17
> 6months	3	0.42 [0.23 to 0.61]	< 0.001	448	0	0.29 [0.07 to 0.50]	0.009	366	0	
**HDL-C (mmol/L) (SMD)**										
≤ 2months	10	0.59 [0.14 to 1.03]	0.009	397	0.73	0.39 [0.04 to 0.74]	0.03	386	0.57	
2-6months	7	0.10 [-0.10 to 0.30]	0.33	400	0	0.17 [-0.04 to 0.37]	0.12	378	0	0.18
> 6months	6	-0.00 [-0.26 to 0.25]	0.99	246	0	-0.05 [-0.37 to 0.26]	0.73	194	0.15	
**LDL-C (mmol/L) (SMD)**										
≤ 2months	8	-0.57 [-1.25 to 0.11]	0.10	233	0.81	-0.51 [-1.17 to 0.14]	0.12	229	0.80	
2-6months	7	-0.15 [-0.41 to 0.12]	0.27	400	0.27	-0.06 [-0.26 to 0.15]	0.60	378	0	0.41
> 6months	6	-0.07 [-0.33 to 0.18]	0.59	246	0	-0.05 [-0.34 to 0.24]	0.75	194	0	
**Total cholesterol (mmol/L) (SMD)**										

≤ 2months	10	-0.52 [-1.01 to -0.02]	0.04	312	0.75	-0.25 [-0.73 to 0.24]	0.32	308	0.74	0.58
2-6months	7	-0.12 [-0.32 to 0.08]	0.24	400	0	-0.14 [-0.35 to 0.07]	0.18	378	0	
> 6months	6	-0.08 [-0.34 to 0.18]	0.54	246	0	0.01 [-0.27 to 0.30]	0.92	194	0	
**Triglycerides (mmol/L) (SMD)**										
≤ 2months	11	-0.42 [-0.82 to -0.01]	0.04	435	0.72	-0.28 [-0.52 to -0.04]	0.02	427	0.24	0.04
2-6months	6	0.08 [-0.14 to 0.29]	0.50	344	0	0.08 [-0.15 to 0.31]	0.50	296	0	
> 6months	4	-0.04 [-0.34 to 0.25]	0.78	179	0	0.18 [-0.17 to 0.53]	0.31	126	0.	
**Fasting Glucose (mmol/L) (SMD)**										
≤ 2months	10	-0.63[-1.11 to -0.16]	0.009	374	0.75	-0.50 [-0.89 to -0.11]	0.01	368	0.63	0.13
2-6months	5	-0.04 [-0.31 to 0.23]	0.75	233	0	-0.01 [-0.29 to 0.28]	0.96	211	0	
> 6months	3	-0.11 [-0.47 to 0.25]	0.54	118	0	-0.24 [-1.11 to 0.63]	0.59	78	0.72	
Overall	18	-0.39 [-0.68 to -0.10]	0.009	725	0.65	-0.31 [-0.57 to -0.05]	0.02	657	0.52	
**Fasting Insulin (µIU/mL) (SMD)**										
≤ 2months	5	-1.27 [-3.64 to 0.49]	0.09	140	0.92	-0.51 [-0.95 to -0.08]	0.02	140	0.34	0.65
2-6months	1	-0.08 [-1.14 to 0.98]	0.89	14	0	-0.12 [-1.18 to 0.94]	0.83	14	0	
> 6months	1	-0.07 [-0.64 to 0.77]	0.85	31	0	-0.18 [-0.93 to 0.56]	0.63	28	0	
Overall	7	-0.86 [-1.87 to 0.14]	0.09	185	0.89	-0.41 [-0.73 to -0.08]	0.006	182	0.12	
**HOMA-IR (SMD)**										
≤ 2months	4	-1.60 [-3.17 to -0.02]	0.05	121	0.92	-0.77 [-1.47 to -0.07]	0.03	121	0.68	0.14
2-6months	3	-0.19 [-0.91 to 0.54]	0.61	49	0	-0.01 [-0.73 to 0.71]	0.98	49	0	
Overall	7	-1.00 [-1.98 to -0.01]	0.05	170	0.85	-0.50 [-1.00 to -0.01]	0.05	170	0.48	

Footnote: All effect sizes reported in this table represent between-group comparisons (Intervention vs. Control). The labels "Pre to post" and "Pre to follow-up" denote the time intervals over which the between-group differences in change scores were evaluated. The sample size reflects the total number of participants (intervention + control) included in each specific meta-analytical comparison."

BMI:Body Mass Index

ES:Effect size
VO_2max:Max Oxygen Uptake
VO_2peak:Peak Oxygen Uptake
HDL-C:High-Density Lipoprotein Cholesterol
LDL-C:Low-Density Lipoprotein Cholesterol
HOMA-IR:Homeostatic Model Assessment of Insulin Resistance

Supplementary Table 4 GRADE evidence profile

Quality assessment							No of patients		Effect		Quality	Importance
No of studies	Design	Risk of bias	Inconsistency	Indirectness	Imprecision	Other considerations	Exercise and detraining	Control	Relative (95% CI)	Absolute		
**Body mass - ≤ 2months (Better indicated by lower values)**												
20	randomised trials	serious[1]	no serious inconsistency	no serious indirectness	no serious imprecision	none	294	232	-	MD 2.09 lower (3.00 to 1.19 lower)	⊕⊕⊕O MODERATE	CRITICAL
**Body mass - 2-6months (Better indicated by lower values)**												
28	randomised trials	serious[1]	no serious inconsistency	no serious indirectness	no serious imprecision	none	665	573	-	MD 1.14 lower (1.66 to 0.62 lower)	⊕⊕⊕O MODERATE	CRITICAL
**Body mass - > 6months (Better indicated by lower values)**												
16	randomised trials	serious[1]	no serious inconsistency	no serious indirectness	no serious imprecision	none	716	441	-	MD 1.12 lower (1.83 to 0.40 lower)	⊕⊕⊕O MODERATE	CRITICAL

**BMI - ≤ 2months (Better indicated by lower values)**												
16	randomised trials	serious[1]	no serious inconsistency	no serious indirectness	no serious imprecision	none	257	212	-	MD 0.62 lower (1.03 to 0.21 lower)	⊕⊕⊕O MODERATE	CRITICAL
**BMI - 2-6months (Better indicated by lower values)**												
25	randomised trials	serious[1]	no serious inconsistency	no serious indirectness	no serious imprecision	none	679	672	-	MD 0.42 lower (0.63 to 0.21 lower)	⊕⊕⊕O MODERATE	CRITICAL
**BMI - > 6months (Better indicated by lower values)**												
12	randomised trials	serious[1]	no serious inconsistency	no serious indirectness	no serious imprecision	none	485	384	-	MD 0.2 lower (0.64 lower to 0.23 higher)	⊕⊕⊕O MODERATE	CRITICAL
**Body fat - ≤ 2months (Better indicated by lower values)**												
16	randomised trials	serious[1]	no serious inconsistency	no serious indirectness	no serious imprecision	none	219	165	-	MD 1.30 lower (2.03 to 0.56 lower)	⊕⊕⊕O MODERATE	
**Body fat - 2-6months (Better indicated by lower values)**												
16	randomised trials	serious[1]	no serious inconsistency	no serious indirectness	no serious imprecision	none	343	222	-	MD 0.56 lower (1.13 lower to 0.01 higher)	⊕⊕⊕O MODERATE	CRITICAL
**Body fat - > 6months (Better indicated by lower values)**												

4	randomised trials	serious[1]	no serious inconsistency	no serious indirectness	serious	none	71	64	-	MD 0.13 lower (0.67 lower to 0.42 higher)	⊕⊕OO LOW	CRITICAL
**Fat mass - ≤ 2months (Better indicated by lower values)**												
6	randomised trials	serious[1]	no serious inconsistency	no serious indirectness	no serious imprecision	none	71	64	-	MD 1.49 lower (2.27 to 0.71 lower)	⊕⊕⊕O MODERATE	CRITICAL
**Fat mass - 2-6months (Better indicated by lower values)**												
10	randomised trials	serious[1]	no serious inconsistency	no serious indirectness	no serious imprecision	none	324	257	-	MD 0.73 lower (1.30 to 0.17 lower)	⊕⊕⊕O MODERATE	CRITICAL
**Fat mass - > 6months (Better indicated by lower values)**												
4	randomised trials	very serious[3]	no serious inconsistency	no serious indirectness	serious[4]	none	87	85	-	MD 0.61 higher (1.05 lower to 2.28 higher)	⊕OOO VERY LOW	IMPORTANT
**Muscle mass (Better indicated by lower values)**												
4	randomised trials	very serious[3]	serious[4]	no serious indirectness	serious[4]	none	60	57	-	MD 0.45 lower (1.67 lower to 0.76 higher)	⊕OOO VERY LOW	IMPORTANT
**Muscle mass - ≤ 2months (Better indicated by lower values)**												
1	randomised	very	very serious[5]	no serious	very serious[5]	none	15	16	-	MD 0.90 higher (0.71	⊕OOO	CRITICAL

	trials	serious[1]		indirectness						lower to 2.51 higher)	VERY LOW	
**Muscle mass - 2-6months (Better indicated by lower values)**												
1	randomised trials	very serious[3]	very serious[6]	no serious indirectness	very serious[6]	none	15	16	-	MD 0.9 higher (0.71 lower to 2.51 higher)	⊕OOO VERY LOW	NOT IMPORTANT
**Muscle mass - > 6months (Better indicated by lower values)**												
2	randomised trials	very serious[3]	serious[6]	no serious indirectness	very serious[6]	none	27	24	-	MD 1.06 lower (2.04 to 0.08 lower)	⊕OOO VERY LOW	NOT IMPORTANT
**Fat-free mass - ≤ 2months (Better indicated by lower values)**												
4	randomised trials	very serious[3]	no serious inconsistency	no serious indirectness	serious[4]	none	70	82	-	MD 1.52 higher (1.16 lower to 4.2 higher)	⊕OOO VERY LOW	IMPORTANT
**Fat-free mass - 2-6months (Better indicated by lower values)**												
7	randomised trials	serious[1]	no serious inconsistency	no serious indirectness	no serious imprecision	none	183	114	-	MD 0.83 lower (1.36 to 0.30 lower)	⊕⊕⊕O MODERATE	CRITICAL
**Lean body mass (Better indicated by lower values)**												
11	randomised trials	serious[1]	no serious inconsistency	no serious indirectness	no serious imprecision	none	299	267	-	MD 0.16 lower (0.55 lower to 0.24 higher)	⊕⊕⊕O MODERATE	CRITICAL

**Lean body mass - ≤ 2months (Better indicated by lower values)**												
2	randomised trials	very serious[3]	no serious inconsistency	no serious indirectness	very serious[6]	none	20	20	-	MD 1.19 higher (2.9 lower to 5.27 higher)	⊕OOO VERY LOW	NOT IMPORTANT
**Lean body mass - 2-6months (Better indicated by lower values)**												
8	randomised trials	serious[1]	no serious inconsistency	no serious indirectness	no serious imprecision	none	239	211	-	MD 0.18 lower (0.59 lower to 0.23 higher)	⊕⊕⊕O MODERATE	IMPORTANT
**Lean body mass - >6months (Better indicated by lower values)**												
1	randomised trials	very serious[3]	very serious[5]	no serious indirectness	very serious[6]	none	40	36	-	MD 0 higher (1.86 lower to 1.86 higher)	⊕OOO VERY LOW	NOT IMPORTANT
**Waist circumference - ≤ 2months (Better indicated by lower values)**												
6	randomised trials	serious[1]	no serious inconsistency	no serious indirectness	no serious imprecision	none	102	139	-	MD 2.17 lower (3.55 to 0.79 lower)	⊕⊕⊕O MODERATE	CRITICAL
**Waist circumference - 2-6months (Better indicated by lower values)**												
13	randomised trials	serious[1]	no serious inconsistency	no serious indirectness	no serious imprecision	reporting bias	275	224	-	MD 1.43 lower (2.40 to 0.46 lower)	⊕⊕OO LOW	CRITICAL
**Waist circumference - >6months (Better indicated by lower values)**												

10	randomised trials	serious[1]	no serious inconsistency	no serious indirectness	no serious imprecision	none	486	329	-	MD 1.89 lower (2.75 to 1.04 lower)	⊕⊕⊕O MODERATE	CRITICAL
**Waist-to-Hip Ratio (Better indicated by lower values)**												
13	randomised trials	serious[1]	no serious inconsistency	no serious indirectness	no serious imprecision	reporting bias[2]	224	179	-	MD 0.01 lower (0.02 lower to 0 lower)	⊕⊕OO LOW	CRITICAL
**Waist-to-Hip Ratio - ≤ 2months (Better indicated by lower values)**												
4	randomised trials	very serious[3]	no serious inconsistency	no serious indirectness	serious[4]	none	38	27	-	MD 0.02 lower (0.05 lower to 0.01 higher)	⊕OOO VERY LOW	CRITICAL
**Waist-to-Hip Ratio - 2-6months (Better indicated by lower values)**												
9	randomised trials	serious[1]	no serious inconsistency	no serious indirectness	no serious imprecision	none	186	152	-	MD 0.01 lower (0.01 lower to 0 higher)	⊕⊕⊕O MODERATE	IMPORTANT
**Skinfold (Better indicated by lower values)**												
5	randomised trials	very serious[3]	no serious inconsistency	no serious indirectness	no serious imprecision	none	67	36	-	MD 1.74 lower (7.80 lower to 3.31 higher)	⊕⊕OO LOW	IMPORTANT
**Systolic blood pressure - ≤ 2months (Better indicated by lower values)**												
12	randomised	serious[1]	no serious	no serious	no serious	none	206	253	-	MD 2.34 lower (4.41 to	⊕⊕⊕O	CRITICAL

	trials		inconsistency	indirectness	imprecision					0.28 lower)	MODERATE	
**Systolic blood pressure - 2-6months (Better indicated by lower values)**												
11	randomised trials	serious[1]	serious[7]	no serious indirectness	no serious imprecision	none	365	350	-	MD 1.89 lower (4.98 lower to 1.21 higher)	⊕⊕OO LOW	IMPORTANT
**Systolic blood pressure - >6months (Better indicated by lower values)**												
12	randomised trials	serious[1]	no serious inconsistency	no serious indirectness	no serious imprecision	none	348	258	-	MD 2.69 lower (4.73 to 0.66 lower)	⊕⊕⊕O MODERATE	IMPORTANT
**Diastolic blood pressure - ≤ 2months (Better indicated by lower values)**												
12	randomised trials	serious[1]	no serious inconsistency	no serious indirectness	no serious imprecision	none	206	273	-	MD 1.59 lower (3.17 to 0.01 lower)	⊕⊕⊕O MODERATE	CRITICAL
**Diastolic blood pressure - 2-6months (Better indicated by lower values)**												
11	randomised trials	serious[1]	serious[7]	no serious indirectness	no serious imprecision	none	365	350	-	MD 0.87 lower (3.29 lower to 1.56 higher)	⊕⊕OO LOW	IMPORTANT
**Diastolic blood pressure - >6months (Better indicated by lower values)**												
12	randomised trials	serious[1]	serious[7]	no serious indirectness	no serious imprecision	none	348	258	-	MD 1.93 lower (4.12 lower to 0.26 higher)	⊕⊕OO LOW	CRITICAL

**Mean arterial pressure (Better indicated by lower values)**												
7	randomised trials	serious[1]	no serious inconsistency	no serious indirectness	no serious imprecision	none	86	66	-	MD 3.23 lower (5.70 to 0.77 lower)	⊕⊕⊕O MODERATE	CRITICAL
**Mean arterial pressure - ≤ 2months (Better indicated by lower values)**												
7	randomised trials	serious[1]	no serious inconsistency	no serious indirectness	no serious imprecision	none	86	66	-	MD 3.23 lower (5.70 to 0.77 lower)	⊕⊕⊕O MODERATE	CRITICAL
**Resting heart rate - ≤ 2months (Better indicated by lower values)**												
14	randomised trials	serious[1]	no serious inconsistency	no serious indirectness	no serious imprecision	none	197	168	-	MD 0.71 higher (0.84 lower to 2.26 higher)	⊕⊕⊕O MODERATE	CRITICAL
**Resting heart rate - 2-6months (Better indicated by lower values)**												
10	randomised trials	serious[1]	no serious inconsistency	no serious indirectness	no serious imprecision	none	350	341	-	MD 1.46 lower (2.96 lower to 0.05 higher)	⊕⊕⊕O MODERATE	CRITICAL
**Resting heart rate - >6months (Better indicated by lower values)**												
2	randomised trials	very serious[3]	serious[7]	no serious indirectness	very serious[6]	none	167	94	-	MD 4.1 lower (18.47 lower to 10.28 higher)	⊕OOO VERY LOW	NOT IMPORTANT
**VO2peak - ≤ 2months (Better indicated by higher values)**												

9	randomised trials	serious[1]	no serious inconsistency	no serious indirectness	no serious imprecision	none	142	87	-	SMD 0.49 higher (0.12 to 0.85 higher)	⊕⊕⊕O MODERATE	CRITICAL
**VO2peak - 2-6months (Better indicated by higher values)**												
6	randomised trials	serious[1]	no serious inconsistency	no serious indirectness	no serious imprecision	none	153	95	-	SMD 0.37 higher (0.10 to 0.64 higher)	⊕⊕⊕O MODERATE	CRITICAL
**VO2peak - >6months (Better indicated by higher values)**												
11	randomised trials	serious[1]	no serious inconsistency	no serious indirectness	no serious imprecision	none	395	247	-	SMD 0.13 higher (0.03 lower to 0.39 higher)	⊕⊕⊕O MODERATE	CRITICAL
**VO2max - ≤ 2months (Better indicated by higher values)**												
10	randomised trials	serious[1]	no serious inconsistency	no serious indirectness	no serious imprecision	none	153	143	-	SMD 0.60 higher (0.39 to 0.85 higher)	⊕⊕⊕O MODERATE	CRITICAL
**VO2max - 2-6months (Better indicated by higher values)**												
5	randomised trials	serious[1]	no serious inconsistency	no serious indirectness	no serious imprecision	none	76	48	-	SMD 0.42 higher (0.03 to 0.66 higher)	⊕⊕⊕O MODERATE	CRITICAL
**VO2max - >6months (Better indicated by higher values)**												
3	randomised	serious[1]	no serious	no serious	serious[4]	none	239	127	-	SMD 0.29 higher (0.07	⊕⊕OO	IMPORTANT

	trials		inconsistency	indirectness						to 0.5 higher)	LOW	
**Total cholesterol - ≤ 2months (Better indicated by lower values)**												
10	randomised trials	serious[1]	serious[7]	no serious indirectness	no serious imprecision	none	175	133	-	SMD 0.25 lower (0.73 lower to 0.24 higher)	⊕⊕OO LOW	CRITICAL
**Total cholesterol - 2-6months (Better indicated by lower values)**												
7	randomised trials	serious[1]	no serious inconsistency	no serious indirectness	no serious imprecision	none	217	161	-	SMD 0.14 lower (0.35 lower to 0.07 higher)	⊕⊕⊕O MODERATE	IMPORTANT
**Total cholesterol - >6months (Better indicated by lower values)**												
6	randomised trials	serious[1]	no serious inconsistency	no serious indirectness	no serious imprecision	none	109	85	-	SMD 0.01 higher (0.27 lower to 0.3 higher)	⊕⊕⊕O MODERATE	IMPORTANT
**HDL-C - ≤ 2months (Better indicated by higher values)**												
10	randomised trials	serious[1]	serious[7]	no serious indirectness	no serious imprecision	none	188	198	-	SMD 0.39 higher (0.04 to 0.74 higher)	⊕⊕OO LOW	CRITICAL
**HDL-C - 2-6months (Better indicated by higher values)**												
7	randomised trials	serious[1]	no serious inconsistency	no serious indirectness	no serious imprecision	none	217	161	-	SMD 0.17 higher (0.04 lower to 0.37 higher)	⊕⊕⊕O MODERATE	IMPORTANT

**HDL-C - >6months (Better indicated by higher values)**												
6	randomised trials	serious[1]	no serious inconsistency	no serious indirectness	no serious imprecision	none	109	85	-	SMD 0.05 lower (0.37 lower to 0.26 higher)	⊕⊕⊕O MODERATE	IMPORTANT
**LDL-C - ≤ 2months (Better indicated by lower values)**												
8	randomised trials	serious[1]	serious[7]	no serious indirectness	no serious imprecision	none	133	96	-	SMD 0.51 lower (1.17 lower to 0.14 higher)	⊕⊕OO LOW	CRITICAL
**LDL-C - 2-6months (Better indicated by lower values)**												
7	randomised trials	serious[1]	no serious inconsistency	no serious indirectness	no serious imprecision	none	217	161	-	SMD 0.06 lower (0.26 lower to 0.15 higher)	⊕⊕⊕O MODERATE	IMPORTANT
**LDL-C - >6months (Better indicated by lower values)**												
6	randomised trials	serious[1]	no serious inconsistency	no serious indirectness	no serious imprecision	none	109	85	-	SMD 0.05 lower (0.34 lower to 0.24 higher)	⊕⊕⊕O MODERATE	IMPORTANT
**Triglycerides - ≤ 2months (Better indicated by lower values)**												
11	randomised trials	serious[1]	no serious inconsistency	no serious indirectness	no serious imprecision	none	209	218	-	SMD 0.28 lower (0.52 to 0.04 lower)	⊕⊕⊕O MODERATE	CRITICAL
**Triglycerides - 2-6months (Better indicated by lower values)**												

6	randomised trials	serious[1]	no serious inconsistency	no serious indirectness	no serious imprecision	none	162	134	-	SMD 0.08 higher (0.15 lower to 0.31 higher)	⊕⊕⊕O MODERATE	IMPORTANT
**Triglycerides - >6months (Better indicated by lower values)**												
4	randomised trials	very serious[3]	no serious inconsistency	no serious indirectness	serious[4]	none	63	63	-	SMD 0.18 higher (0.17 lower to 0.53 higher)	⊕OOO VERY LOW	IMPORTANT
**Fasting glucose (Better indicated by lower values)**												
18	randomised trials	serious[1]	no serious inconsistency	no serious indirectness	no serious imprecision	none	332	325	-	SMD 0.31 lower (0.57 to 0.05 lower)	⊕⊕⊕O MODERATE	CRITICAL
**Fasting glucose - ≤ 2months (Better indicated by lower values)**												
10	randomised trials	serious[1]	serious[7]	no serious indirectness	no serious imprecision	none	164	204	-	SMD 0.50 lower (0.89 to 0.11 lower)	⊕⊕OO LOW	CRITICAL
**Fasting glucose - 2-6months (Better indicated by lower values)**												
5	randomised trials	very serious[3]	no serious inconsistency	no serious indirectness	no serious imprecision	none	127	84	-	SMD 0.01 higher (0.29 lower to 0.28 higher)	⊕⊕OO LOW	IMPORTANT
**Fasting glucose - >6months (Better indicated by lower values)**												
3	randomised	very	serious[7]	no serious	serious[4]	none	41	37	-	SMD 0.24 lower (1.11	⊕OOO	IMPORTANT

	trials	serious[3]		indirectness						lower to 0.63 higher)	VERY LOW	
**HOMA-IR (Better indicated by lower values)**												
7	randomised trials	serious[1]	serious[7]	no serious indirectness	no serious imprecision	none	101	69	-	SMD 0.50 lower (1 lower to 0.01 higher)	⊕⊕OO LOW	CRITICAL
**HOMA-IR - ≤ 2months (Better indicated by lower values)**												
4	randomised trials	serious[1]	serious[7]	no serious indirectness	serious[4]	none	61	60	-	SMD 0.77 lower (1.47 to 0.07 lower)	⊕OOO VERY LOW	CRITICAL
**HOMA-IR - 2-6months (Better indicated by lower values)**												
3	randomised trials	very serious[3]	no serious inconsistency	no serious indirectness	serious[4]	none	40	9	-	SMD 0.01 lower (0.73 lower to 0.41 higher)	⊕OOO VERY LOW	IMPORTANT
**Fasting insulin (Better indicated by lower values)**												
7	randomised trials	serious[1]	no serious inconsistency	no serious indirectness	no serious imprecision	none	94	88	-	SMD 0.41 lower (0.73 to 0.08 lower)	⊕⊕⊕O MODERATE	CRITICAL
**Fasting insulin - ≤ 2months (Better indicated by lower values)**												
5	randomised trials	serious[1]	no serious inconsistency	no serious indirectness	no serious imprecision	none	71	69	-	SMD 0.51 lower (0.95 to 0.08 lower)	⊕⊕⊕O MODERATE	

**Fasting insulin - 2-6months (Better indicated by lower values)**												
1	randomised trials	very serious[3]	very serious[5]	no serious indirectness	very serious[5]	none	8	6	-	SMD 0.12 lower (1.18 lower to 0.94 higher)	⊕OOO VERY LOW	
**Fasting insulin - >6months (Better indicated by lower values)**												
1	randomised trials	very serious[3]	very serious[5]	no serious indirectness	very serious[5]	none	15	13	-	SMD 0.18 lower (0.93 lower to 0.56 higher)	⊕OOO VERY LOW	

[1] >30% of the included studies lacked allocation concealment.

[2] Egger test p<0.05

[3] 100% of the included studies lacked allocation concealment.

[4] No.of study < 5

[5] No.of study=1

[6] No.of study < 3

[7] I^2 > 50%
