# Supplementary material for: The effects of detraining on body composition and cardiometabolic health: a systematic review and meta-analysis of randomized controlled trials
Source: Front Physiol. 2026 Jul 22;17:1891899. doi: 10.3389/fphys.2026.1891899 (PMC13437958; doi:10.3389/fphys.2026.1891899)

Supplementary Information 2

Table of Content

Figure

Supplementary Figure 1	PRISMA selection flow diagram	pp.4
Supplementary Figure 2	Risk of bias assessment for each included randomized study	pp.5
Supplementary Figure 3	Summary of risk of bias assessment for all included randomized studies	pp.6
Supplementary Figure 4	Leave-one-out sensitivity analysis plot of Body mass	pp.7
Supplementary Figure 5	Leave-one-out sensitivity analysis plot of BMI	pp.8
Supplementary Figure 6	Leave-one-out sensitivity analysis plot of Body fat percentage	pp.9
Supplementary Figure 7	Leave-one-out sensitivity analysis plot of Fat mass	pp.10
Supplementary Figure 8	Leave-one-out sensitivity analysis for Muscle mass	pp.11
Supplementary Figure 9	Leave-one-out sensitivity analysis for Lean body mass	pp.11
Supplementary Figure 10	Leave-one-out sensitivity analysis for Fat-free mass	pp.12
Supplementary Figure 11	Leave-one-out sensitivity analysis for Waist circumference	pp.13
Supplementary Figure 12	Leave-one-out sensitivity analysis for Waist-to-Hip Ratio	pp.14
Supplementary Figure 13	Leave-one-out sensitivity analysis for Skinfold	pp.14
Supplementary Figure 14	Leave-one-out sensitivity analysis for Systolic blood pressure	pp.15
Supplementary Figure 15	Leave-one-out sensitivity analysis for Diastolic blood pressure	pp.16
Supplementary Figure 16	Leave-one-out sensitivity analysis for Mean arterial pressure	pp.17
Supplementary Figure 17	Leave-one-out sensitivity analysis for Resting heart rate	pp.18
Supplementary Figure 18	Leave-one-out sensitivity analysis for VO_{2peak}	pp.19
Supplementary Figure 19	Leave-one-out sensitivity analysis for VO_{2max}	pp.20
Supplementary Figure 20	Leave-one-out sensitivity analysis for Total cholesterol	pp.21
Supplementary Figure 21	Leave-one-out sensitivity analysis for HDL-C	pp.22
Supplementary Figure 22	Leave-one-out sensitivity analysis for LDL-C	pp.23
Supplementary Figure 24	Leave-one-out sensitivity analysis for Fasting blood glucose	pp.25
Supplementary Figure 25	Leave-one-out sensitivity analysis for Fasting insulin	pp.25
Supplementary Figure 26	Leave-one-out sensitivity analysis for HOMA-IR	pp.26
Supplementary Figure 27	Sensitivity analysis excluding very-long-term trials	pp.26
Supplementary Figure 28	Forest plot of subanalyses of Body mass	pp.27
Supplementary Figure 29	Forest plot of subanalyses of BMI	pp.28
Supplementary Figure 30	Forest plot of subanalyses of Body fat percentage	pp.29

Supplementary Figure 31 Forest plot of subanalyses of Fat mass pp.30
Supplementary Figure 32 Forest plot of subanalyses of Muscle mass pp.30
Supplementary Figure 33 Forest plot of subanalyses of Waist circumference pp.31
Supplementary Figure 34 Forest plot of subanalyses of Waist-to-Hip Ratio pp.31
Supplementary Figure 35 Forest plot of subanalyses of Fat-free mass pp.32
Supplementary Figure 36 Forest plot of subanalyses of Lean body mass pp.32
Supplementary Figure 37 Forest plot of subanalyses of Skinfold pp.32
Supplementary Figure 38 Forest plot of subanalyses of Systolic blood pressure pp.33
Supplementary Figure 39 Forest plot of subanalyses of Diastolic blood pressure pp.34
Supplementary Figure 40 Forest plot of subanalyses of Mean arterial pressure pp.34
Supplementary Figure 41 Forest plot of subanalyses of resting heart rate pp.35
Supplementary Figure 42 Forest plot of subanalyses of VO_{2peak} pp.36
Supplementary Figure 43 Forest plot of subanalyses of VO_{2max} pp.37
Supplementary Figure 44 Forest plot of subanalyses of Total cholesterol pp.38
Supplementary Figure 45 Forest plot of subanalyses of LDL-C pp.39
Supplementary Figure 46 Forest plot of subanalyses of HDL-C pp.40
Supplementary Figure 47 Forest plot of subanalyses of Triglycerides pp.41
Supplementary Figure 48 Forest plot of subanalyses of HOMA-IR pp.41
Supplementary Figure 49 Forest plot of subanalyses of Fasting blood glucose pp.42
Supplementary Figure 50 Forest plot of subanalyses of Fasting insulin pp.42
Supplementary Figure 51 Funnel plot of study bias on Body mass pp.43
Supplementary Figure 52 Funnel plot of study bias on BMI pp.43
Supplementary Figure 53 Funnel plot of study bias on Body fat percentage pp.44
Supplementary Figure 54 Funnel plot of study bias on Fat mass pp.44
Supplementary Figure 55 Funnel plot of study bias on Muscle mass pp.45
Supplementary Figure 56 Funnel plot of study bias on Waist circumference pp.45
Supplementary Figure 57 Funnel plot of study bias on Waist-to-Hip Ratio pp.46
Supplementary Figure 58 Funnel plot of study bias on Fat-free mass pp.46
Supplementary Figure 59 Funnel plot of study bias on Lean body mass pp.47
Supplementary Figure 60 Funnel plot of study bias on Skinfold pp.47
Supplementary Figure 61 Funnel plot of study bias on Systolic blood pressure pp.48
Supplementary Figure 62 Funnel plot of study bias on Diastolic blood pressure pp.48
Supplementary Figure 63 Funnel plot of study bias on Mean arterial pressure pp.49

Supplementary Figure 64	Funnel plot of study bias on Resting heart rate	pp.49
Supplementary Figure 65	Funnel plot of study bias on VO_{2peak}	pp.50
Supplementary Figure 66	Funnel plot of study bias on VO_{2max}	pp.50
Supplementary Figure 67	Funnel plot of study bias on Total cholesterol	pp.51
Supplementary Figure 68	Funnel plot of study bias on HDL-C	pp.51
Supplementary Figure 69	Funnel plot of study bias on LDL-C	pp.52
Supplementary Figure 70	Funnel plot of study bias on Triglycerides	pp.52
Supplementary Figure 71	Funnel plot of study bias on HOMA-IR	pp.53
Supplementary Figure 72	Funnel plot of study bias on Fasting blood glucose	pp.53
Supplementary Figure 73	Funnel plot of study bias on Fasting insulin	pp.54
Supplementary Figure 74	Trim-and-fill funnel plot for Body mass in short-term subgroup	pp.54
Supplementary Figure 75	Trim-and-fill funnel plot for BMI in short-term subgroup	pp.55
Supplementary Figure 76	Trim-and-fill funnel plot for Waist circumference in long-term subgroup	pp.55
Supplementary Figure 77	Trim-and-fill funnel plot for Resting heart rate in short-term subgroup	pp.56

Supplementary Figure 1 PRISMA selection flow diagram

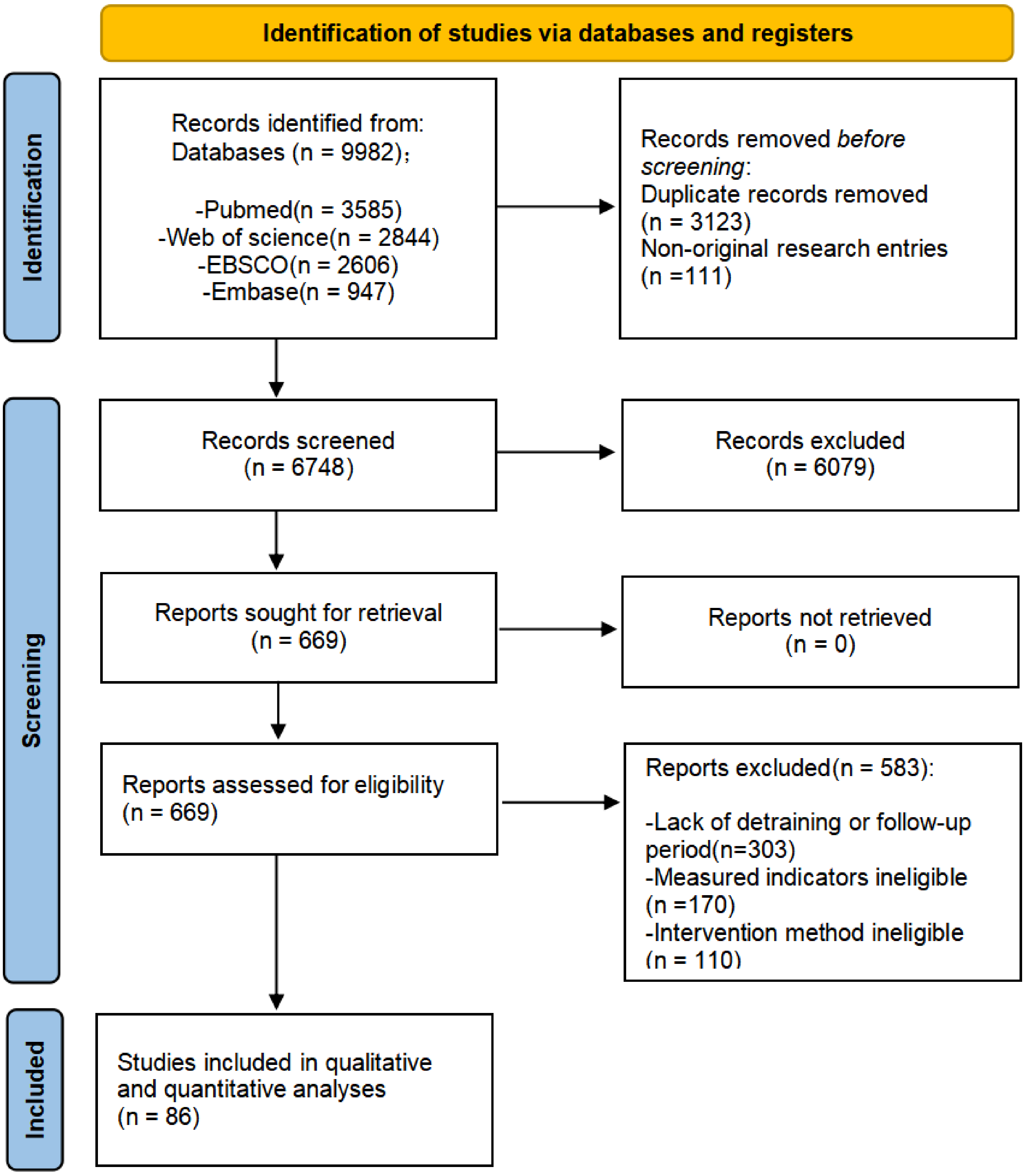

Supplementary Figure 2 Risk of bias assessment for each included randomized Study

Study	D1	D2	D3	D4	D5	Overall
Anandavadivelan 2024	-	-	+	+	-	-
Batrakoulis 2018	+	+	+	+	+	+
Bertz 2012	-	-	+	+	-	-
Bisconti 2020	-	-	+	+	-	-
Bjerre 2019	-	+	+	+	+	-
Brekke 2014	-	-	+	+	+	-
Brobakken 2024	-	-	+	+	-	-
Broderick 2013	-	-	+	+	-	-
Brooker 2023	-	+	+	+	+	-
Cantarero-Villanueva 2016	-	-	+	+	+	-
Carei 2009	+	+	+	+	-	-
Carter 2013	-	-	+	+	-	-
Carvalho 2009	-	+	+	+	-	-
Chee Huei 2017	-	+	+	+	-	-
Chen 2018	-	+	+	+	-	-
Ciccolo 2011	+	+	+	+	-	-
Donyaei 2024	-	+	+	+	-	-
Elliott 2002	-	+	+	+	-	-
Fatouros 2004	-	+	+	+	-	-
Fatouros 2005	-	-	-	+	-	-
Fernandez-del-Valle 2014	-	X	X	X	-	X
Frost 2020	X	X	+	+	+	X
Furzer 2016	-	-	+	+	-	-
Gjellesvik 2020	-	-	+	+	+	-
Gram 2010	-	-	+	+	+	-
Haganes 2025	-	-	+	+	+	-
Hamdorf 1993	-	X	+	+	-	X
Hansen 2024	-	X	X	+	+	X
Hao 2019	-	X	X	+	-	X
Herring 2017	-	+	+	+	-	-
Islam 2020	-	-	+	+	-	-
Kagioglou 2021	X	X	+	+	-	X
Kemmler 2020	+	+	+	+	-	-
Kim 2011	-	-	+	+	-	-
Kim 2015	-	+	+	+	+	-
Kindred 2019	-	-	+	+	-	-
Knols 2011	-	-	+	+	-	-
Kranen 2023	-	-	+	+	-	-
Krawcyk 2022	-	+	+	+	-	-
Lee 2021	+	+	+	+	+	+
Li 2015	+	-	+	+	-	-
Liu 2023	-	-	+	+	-	-
Lo 2011	-	-	+	+	-	-
Lovell 2011	-	+	+	+	-	-
Malandish 2020	+	X	+	+	-	X
Martins 2010	X	-	+	+	-	X
Miyachi 2004	-	+	+	+	-	-
Moghadasi 2012	-	-	+	+	-	-
Mundbjerg 2018	+	-	+	+	+	-
Nemoto 2024	-	-	+	+	-	-
Ng 2015	-	-	+	+	-	-
Nikseresht 2016	-	+	+	+	-	-
Niño 2017	-	+	+	+	-	-
Nogueira 2017	-	-	+	+	-	-
Nordén 2024	+	-	+	+	+	-
Nuri 2016	-	+	+	+	-	-
O'Connor 2017	-	-	+	+	-	-
Øiestad 2023	-	-	+	+	+	-
Okamoto 2007	-	+	+	+	-	-
Oliveira 2021	-	+	+	+	-	-
Opdenacker 2011	-	+	+	+	-	-
Prasertsri 2019	-	+	+	+	-	-
Rosado 2022	-	+	+	+	-	-
Rosety-Rodriguez 2014	-	+	+	+	+	-
Rydwik 2007	X	-	+	+	-	X
Schranz 2014	-	-	+	+	-	-
Shokri 2023	-	-	+	+	-	-
Shorki 2021	-	+	+	+	+	-
Sloan 2018	-	-	+	+	+	-
Sloan 2021	-	+	+	+	-	-
Sosa 2012	-	+	+	+	-	-
Sosa 2014	+	+	+	+	-	-
Stebbings 2013	-	-	+	+	-	-
Stener-Victorin 2012	-	+	+	+	+	-
Stergioulas 2006	-	-	+	+	-	-
Teixeira 2023	-	+	+	+	+	-
Theodorou 2016	-	+	+	+	-	-
Thompson 2010	+	+	+	+	+	+
Vainshelboim 2016	-	+	+	+	+	-
Waller 2017	-	+	+	+	-	-
Wang 2022	+	+	+	+	+	+
Winn 2021	-	+	+	+	-	-
Zech 2012	+	-	+	+	+	-
Zhang 2017	-	+	+	+	+	-
Zhao 2024	-	+	+	+	-	-
Zheng 2019	-	+	+	+	+	-

Risk of bias domains

Domains:
D1: Bias arising from the randomization process.
D2: Bias due to deviations from intended intervention.
D3: Bias due to missing outcome data.
D4: Bias in measurement of the outcome.
D5: Bias in selection of the reported result.

Judgement
X High
- Some concerns
+ Low

Supplementary Figure 3 Summary of risk of bias assessment for all included randomized studies

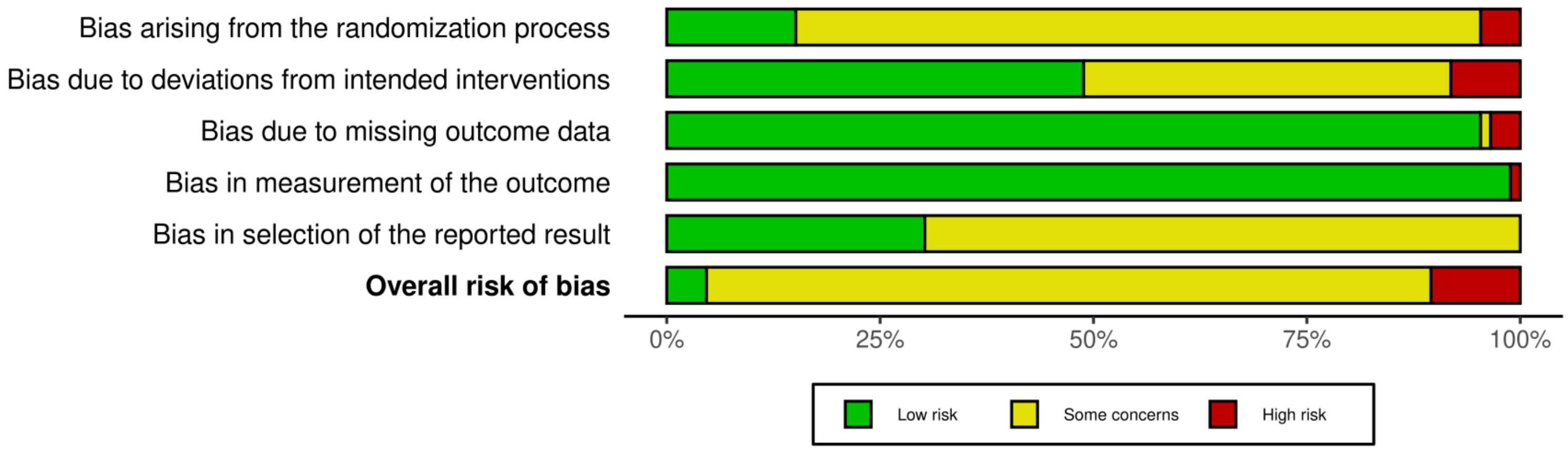

Supplementary Figure 4 Leave-one-out sensitivity analysis for Body mass

Short-term subgroup

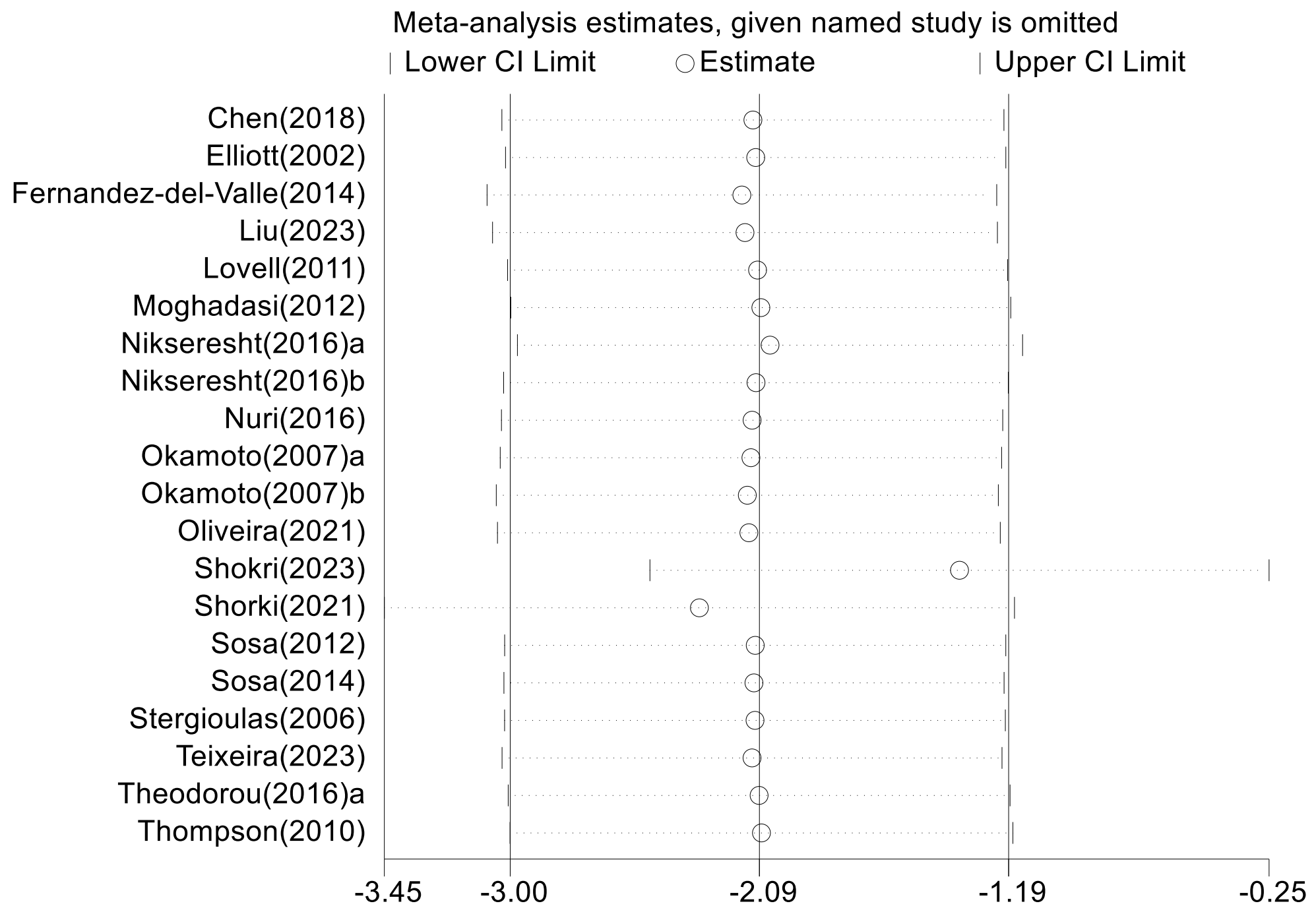

medium-term subgroup

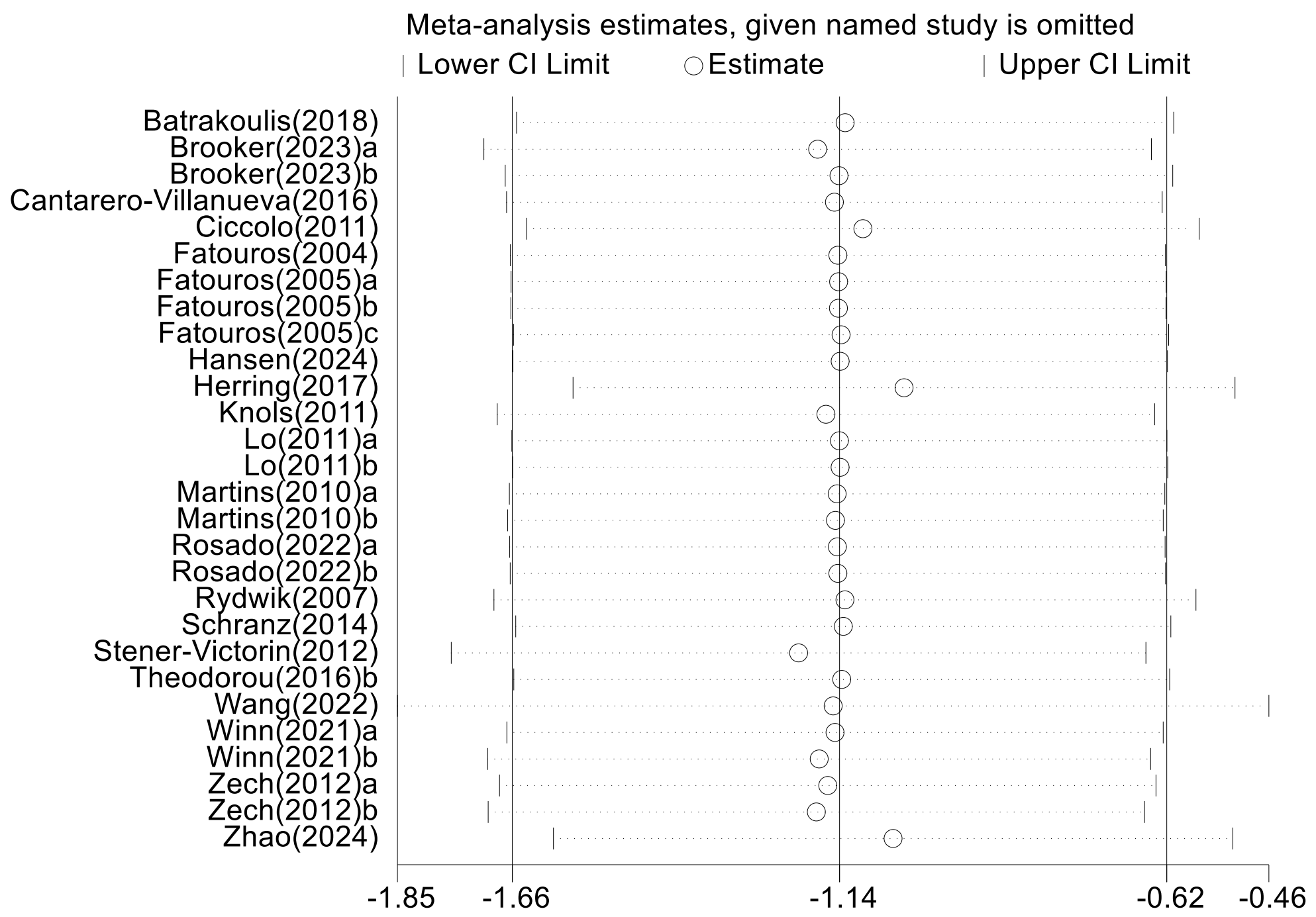

Long-term subgroup

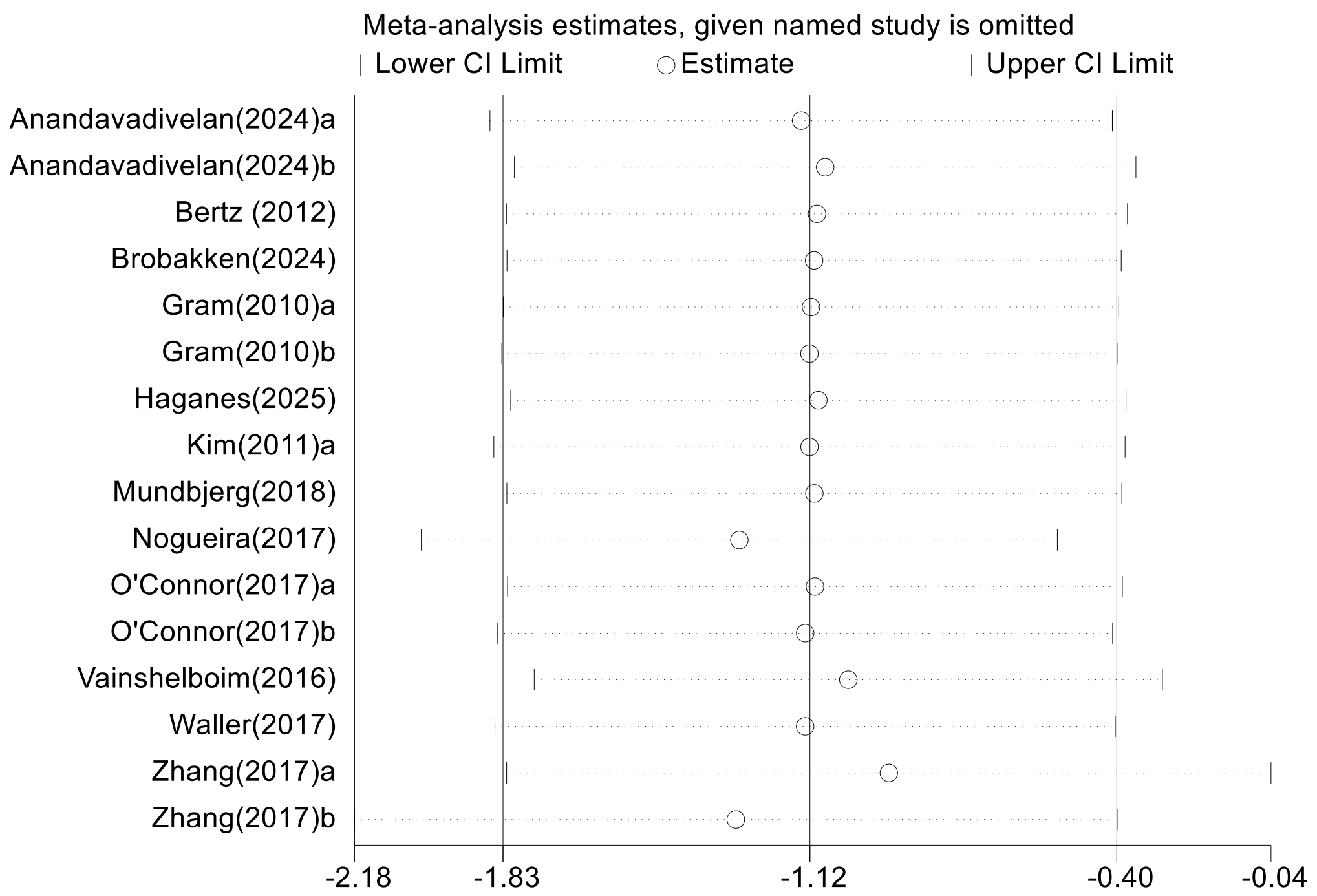

Supplementary Figure 5 Leave-one-out sensitivity analysis for BMI

Short-term subgroup

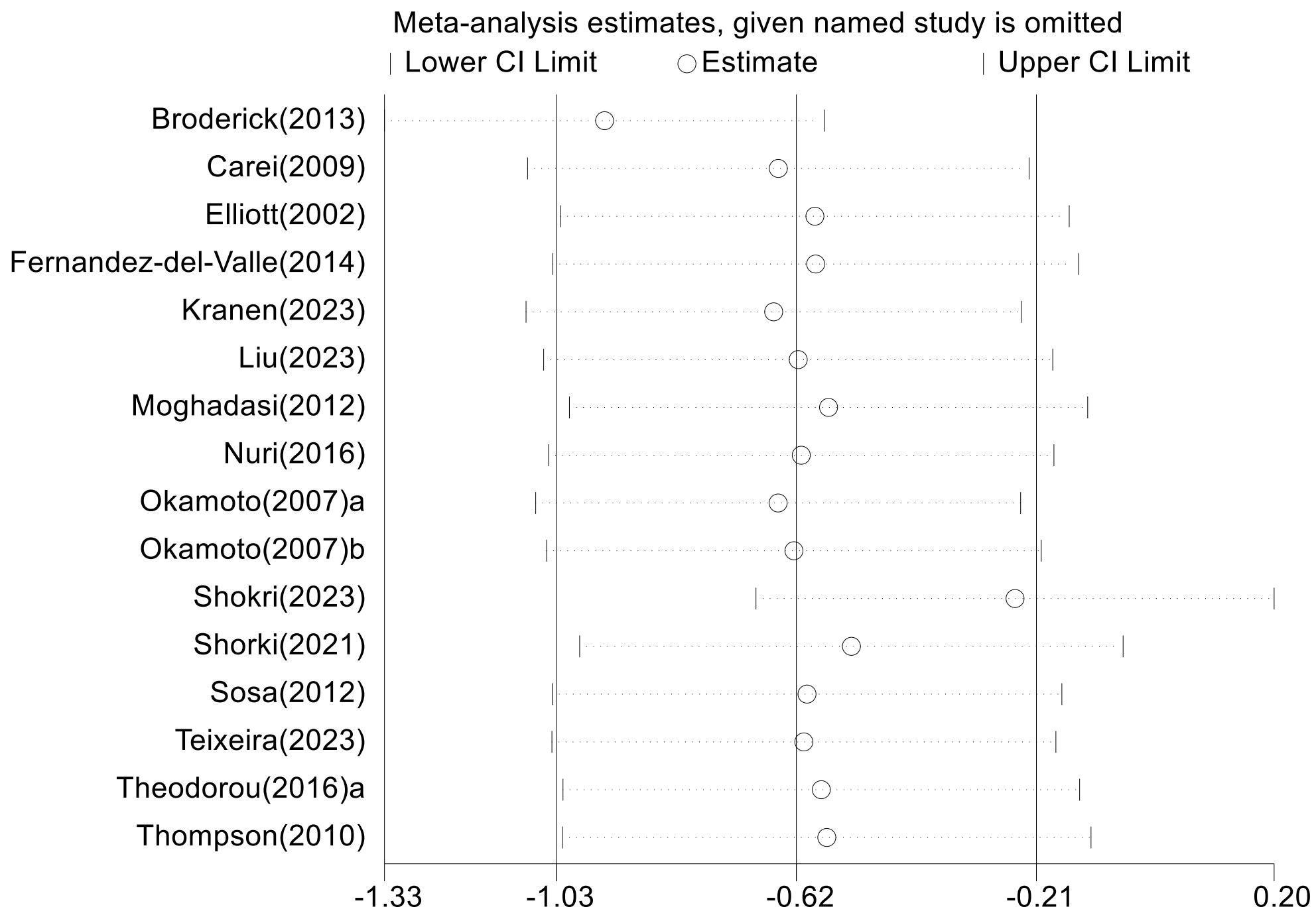

medium-term subgroup

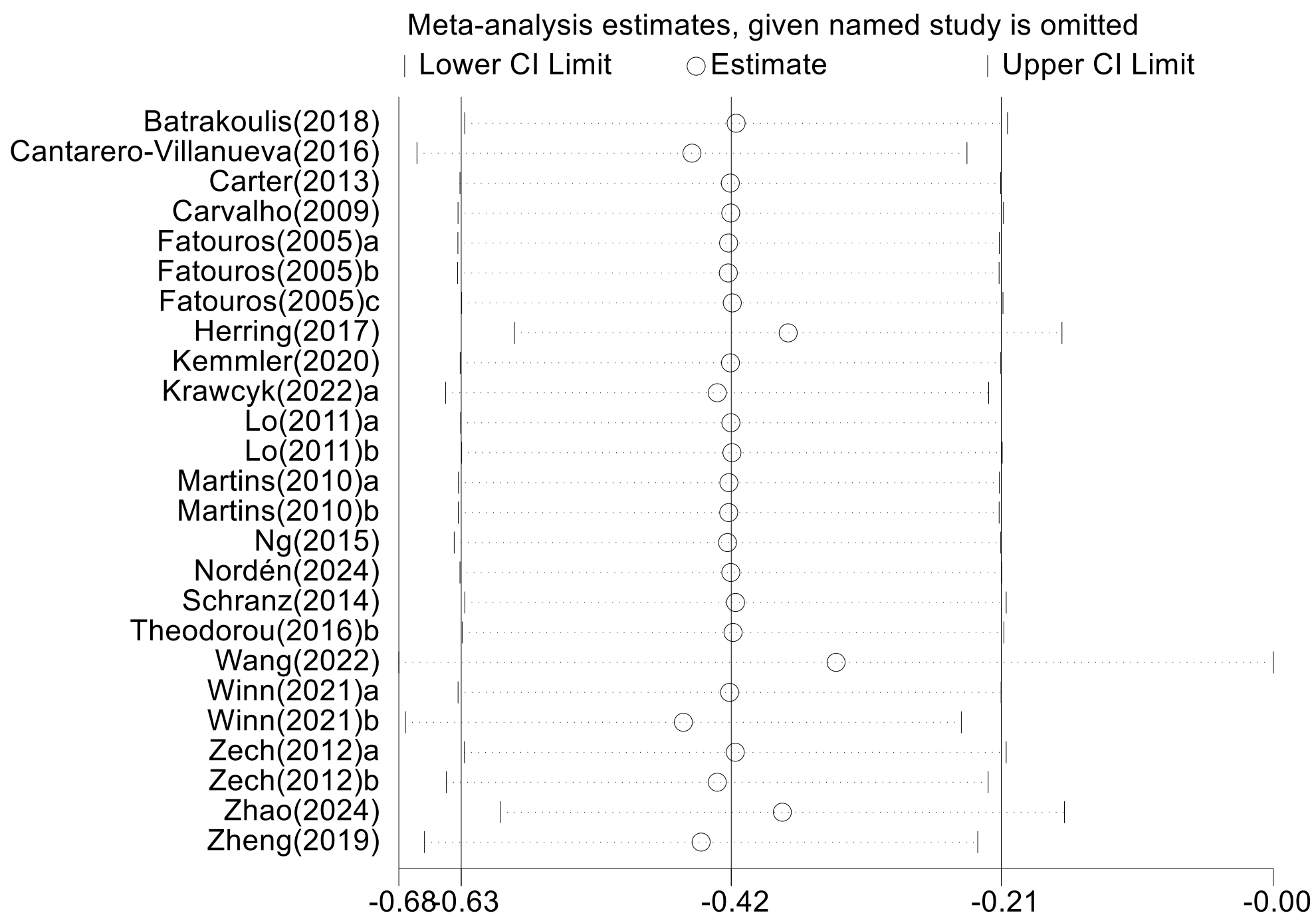

Long-term subgroup

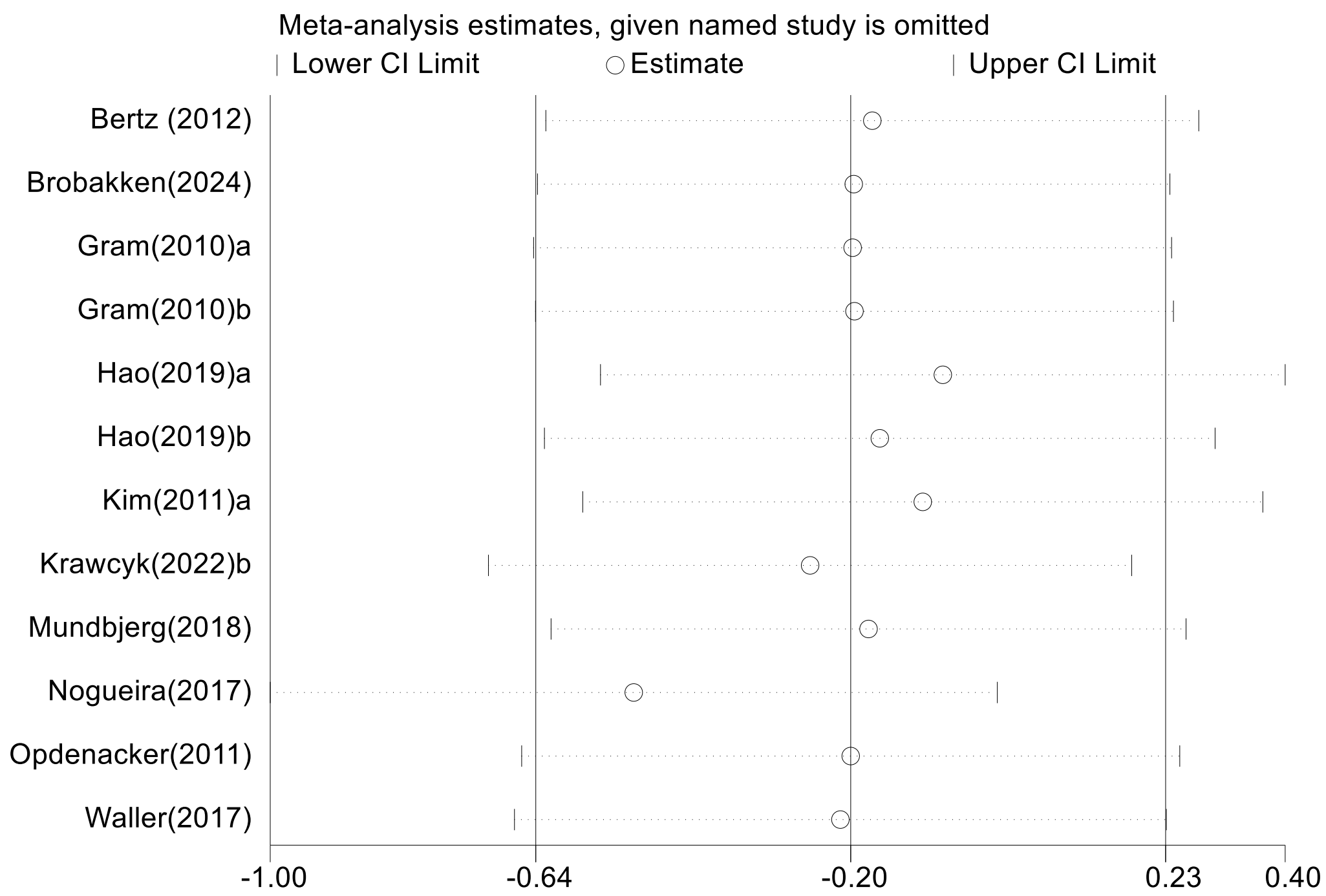

Supplementary Figure 6 Leave-one-out sensitivity analysis for Body fat percentage

Short-term subgroup

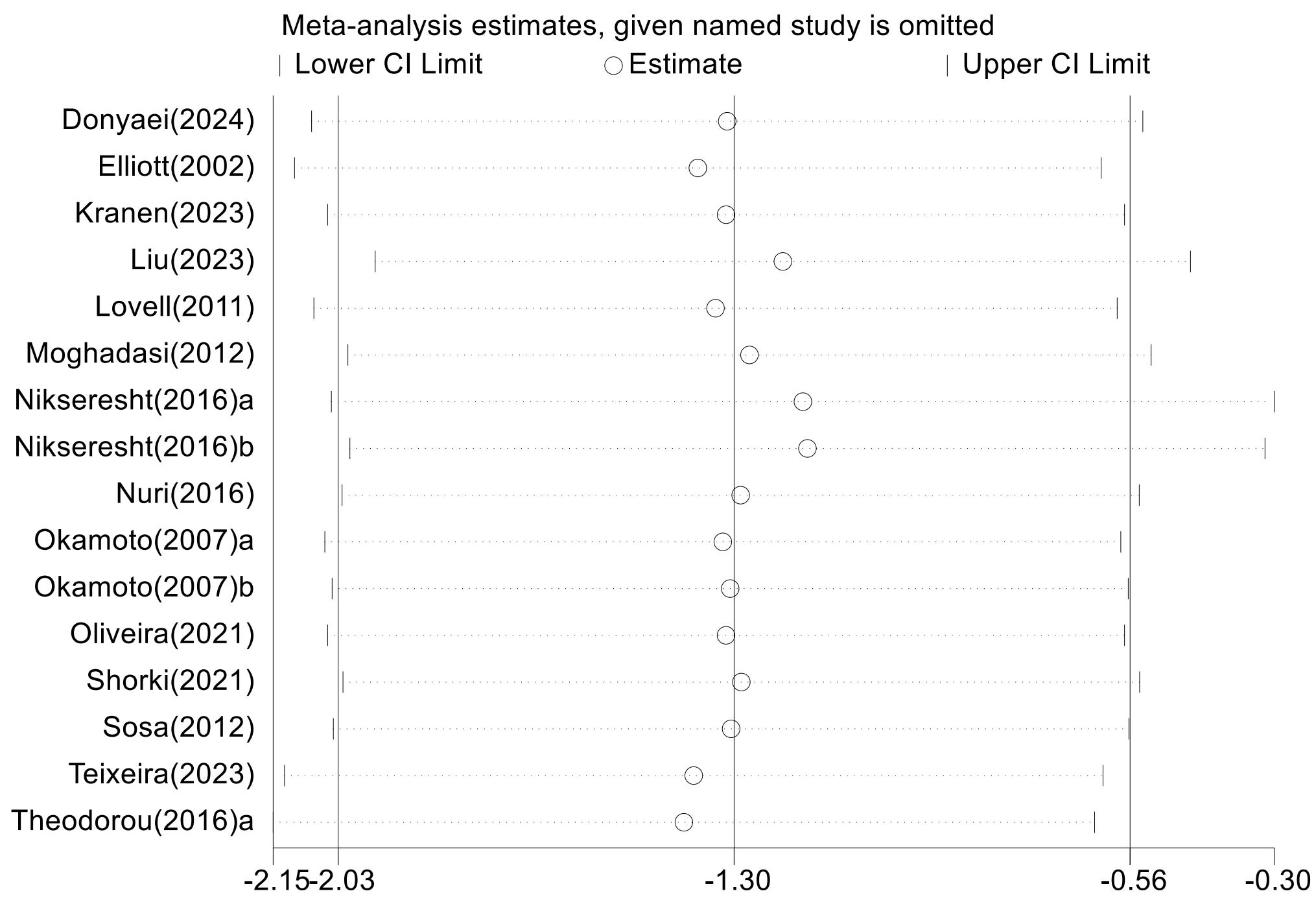

medium-term subgroup

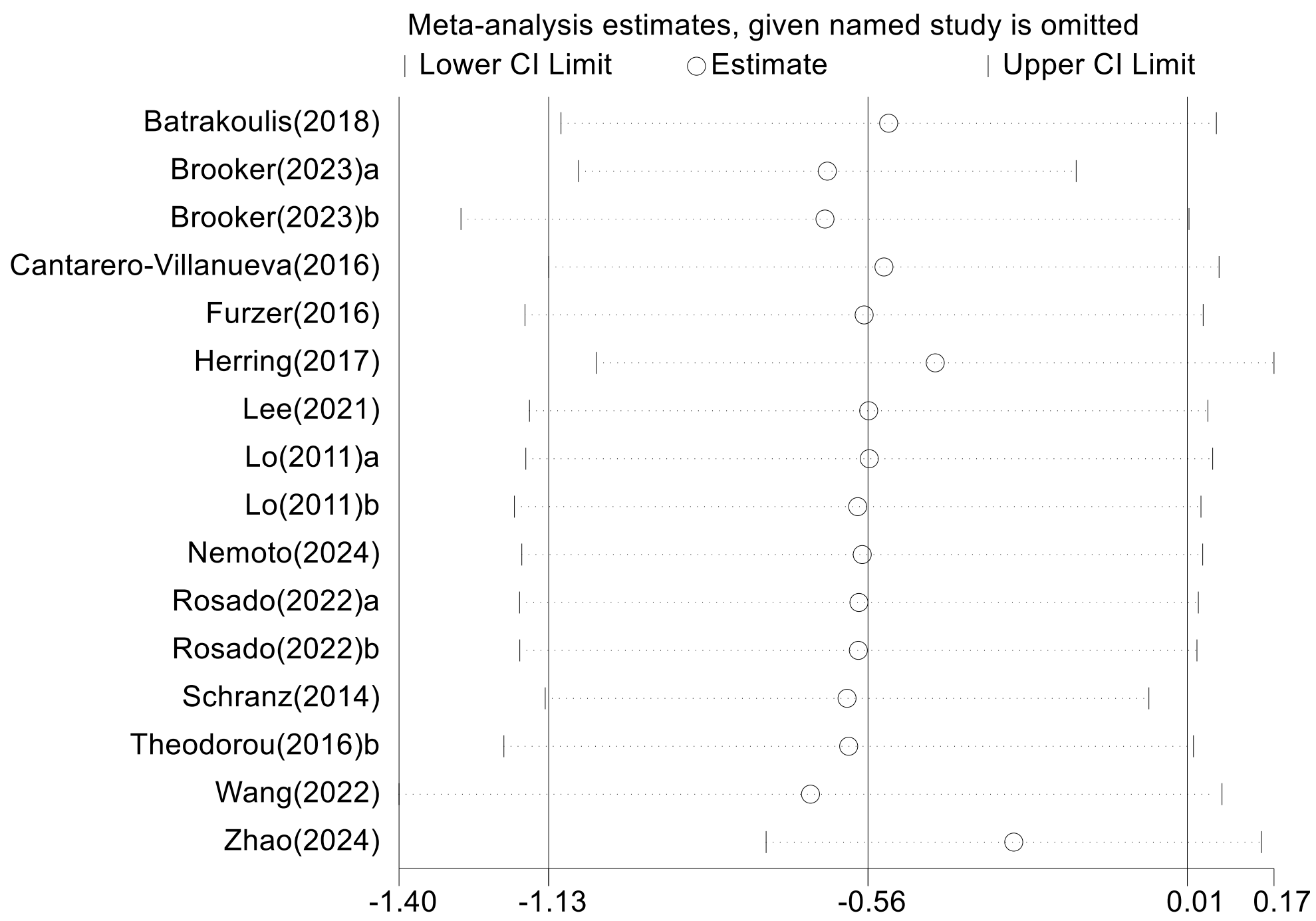

Long-term subgroup

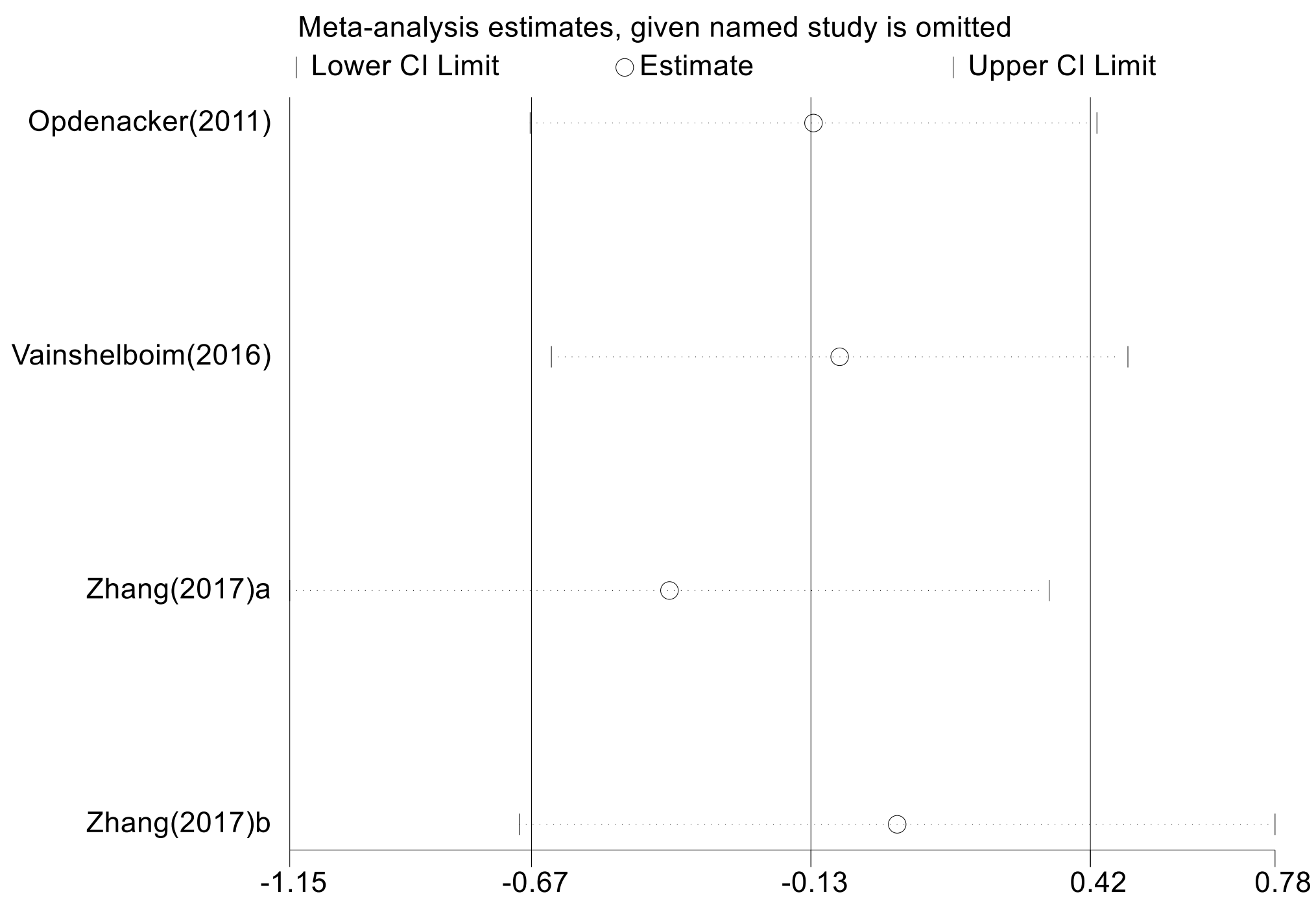

Supplementary Figure 7 Leave-one-out sensitivity analysis for Fat mass

Short-term subgroup

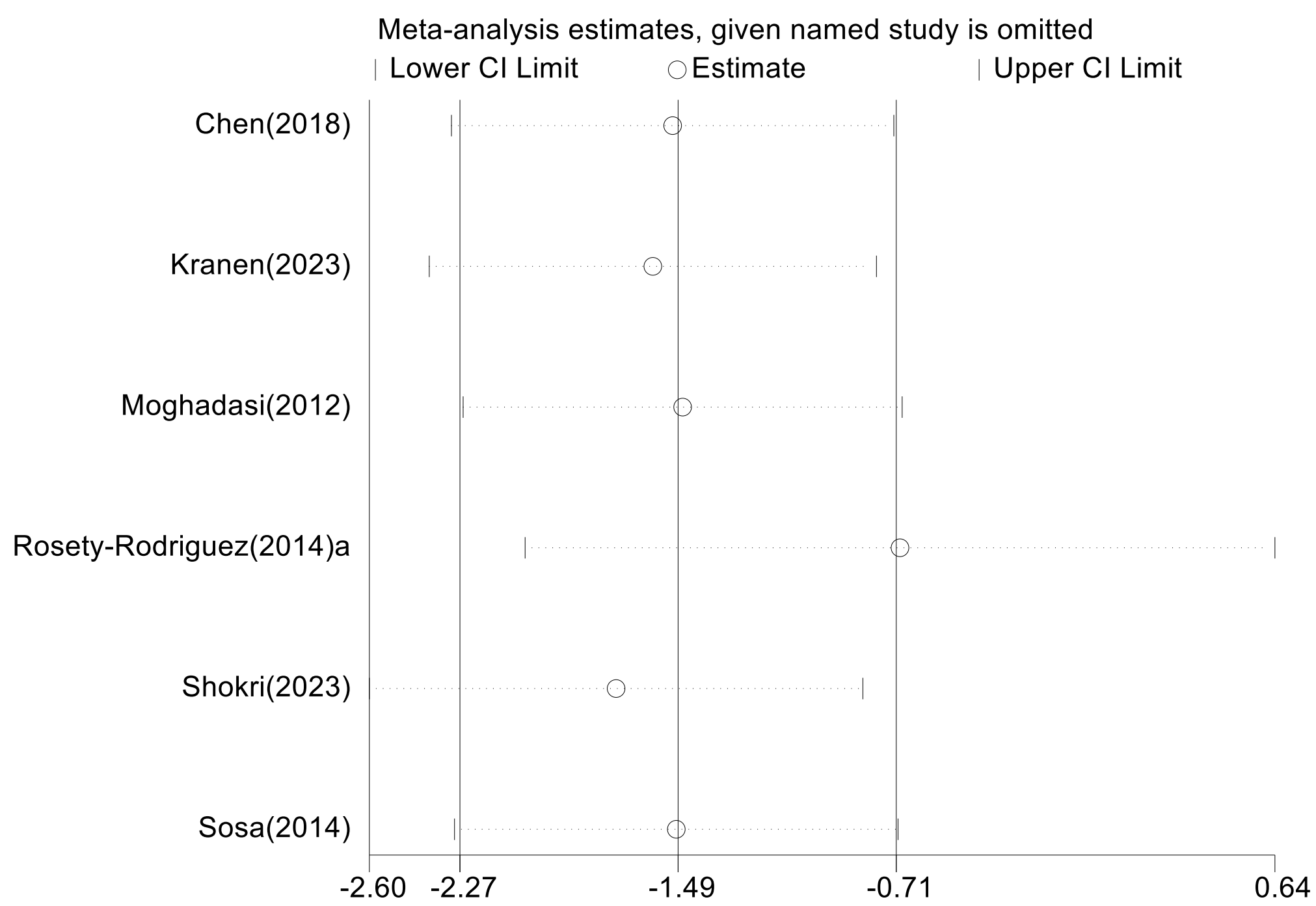

medium-term subgroup

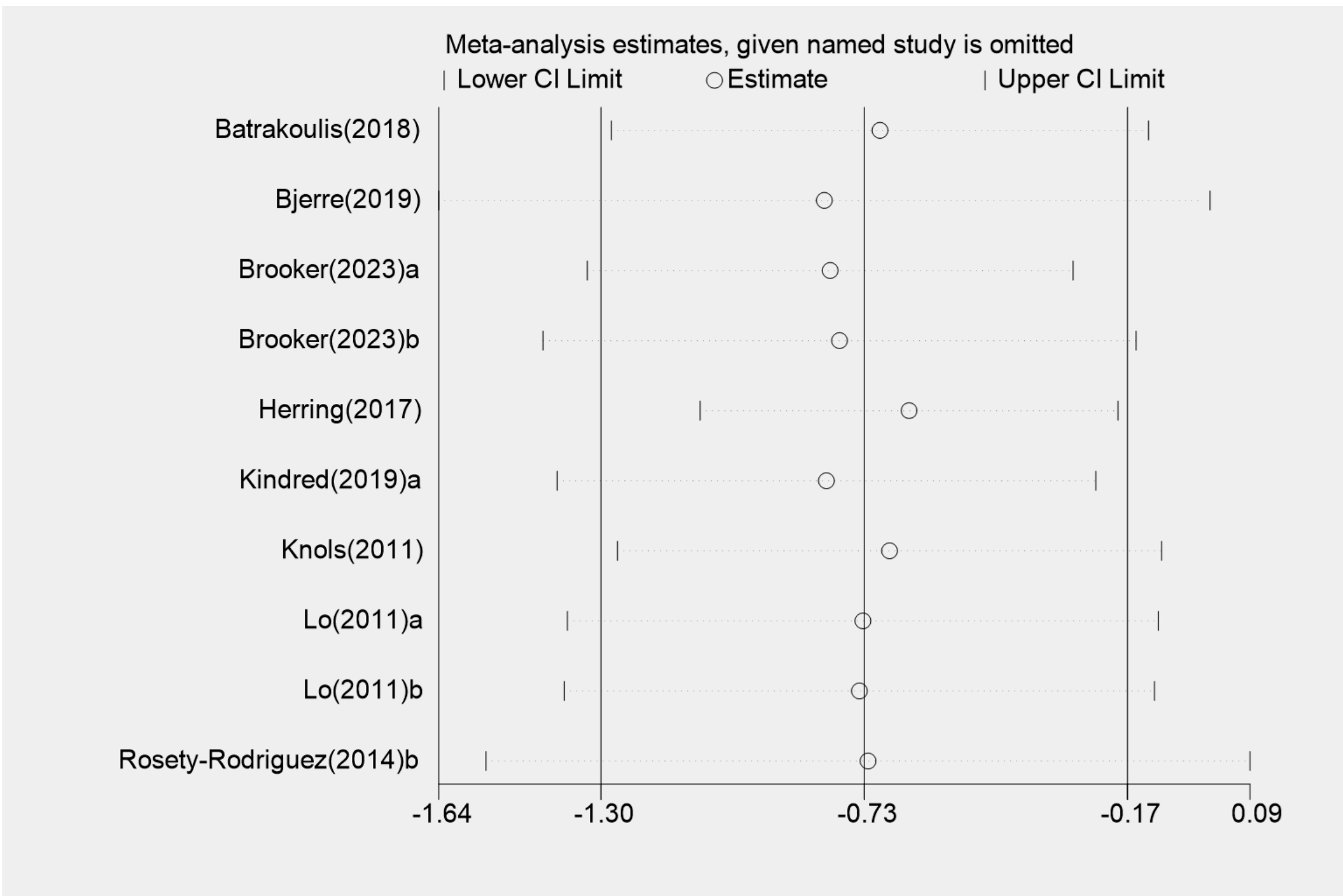

Long-term subgroup

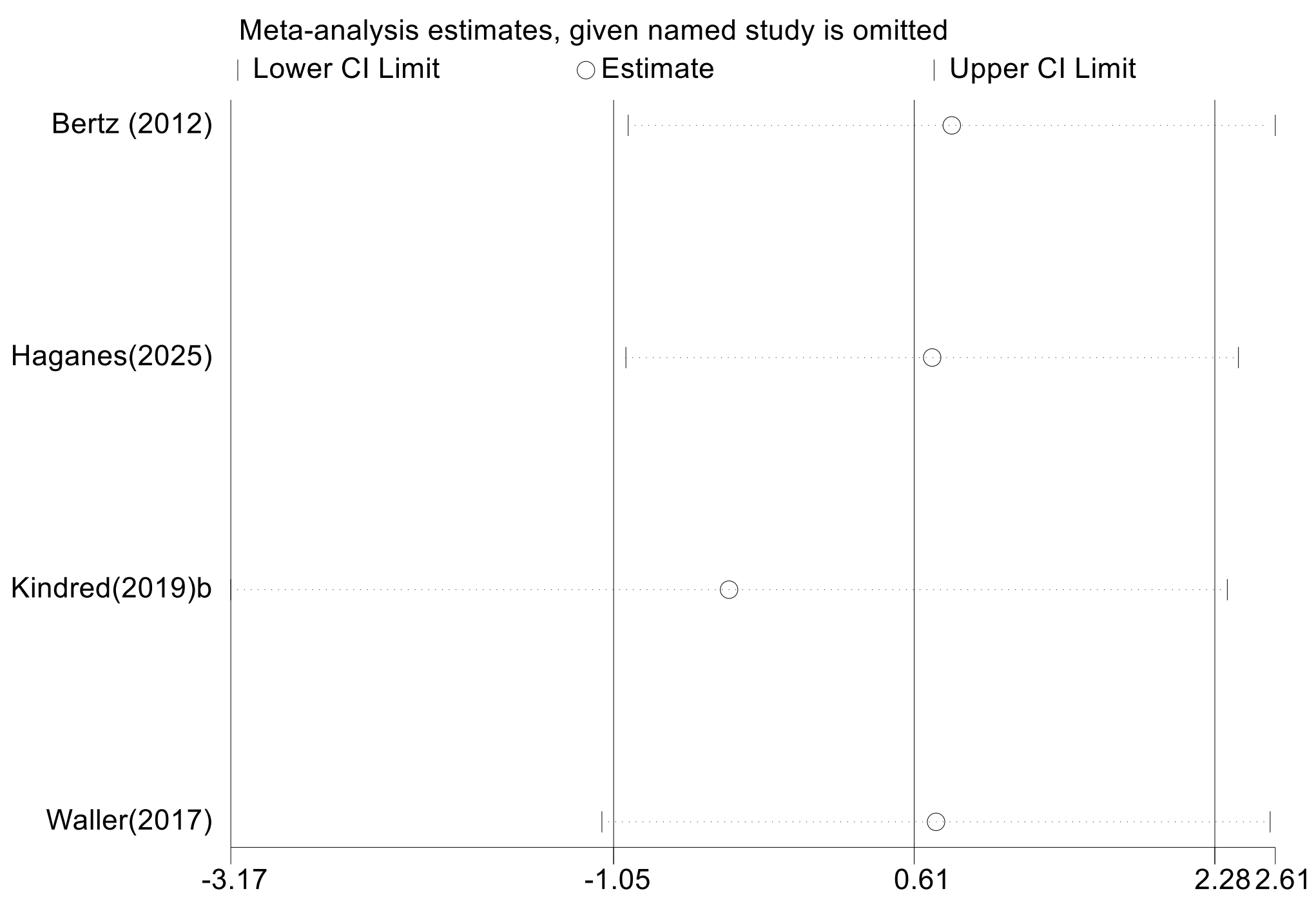

Supplementary Figure 8 Leave-one-out sensitivity analysis for Muscle mass

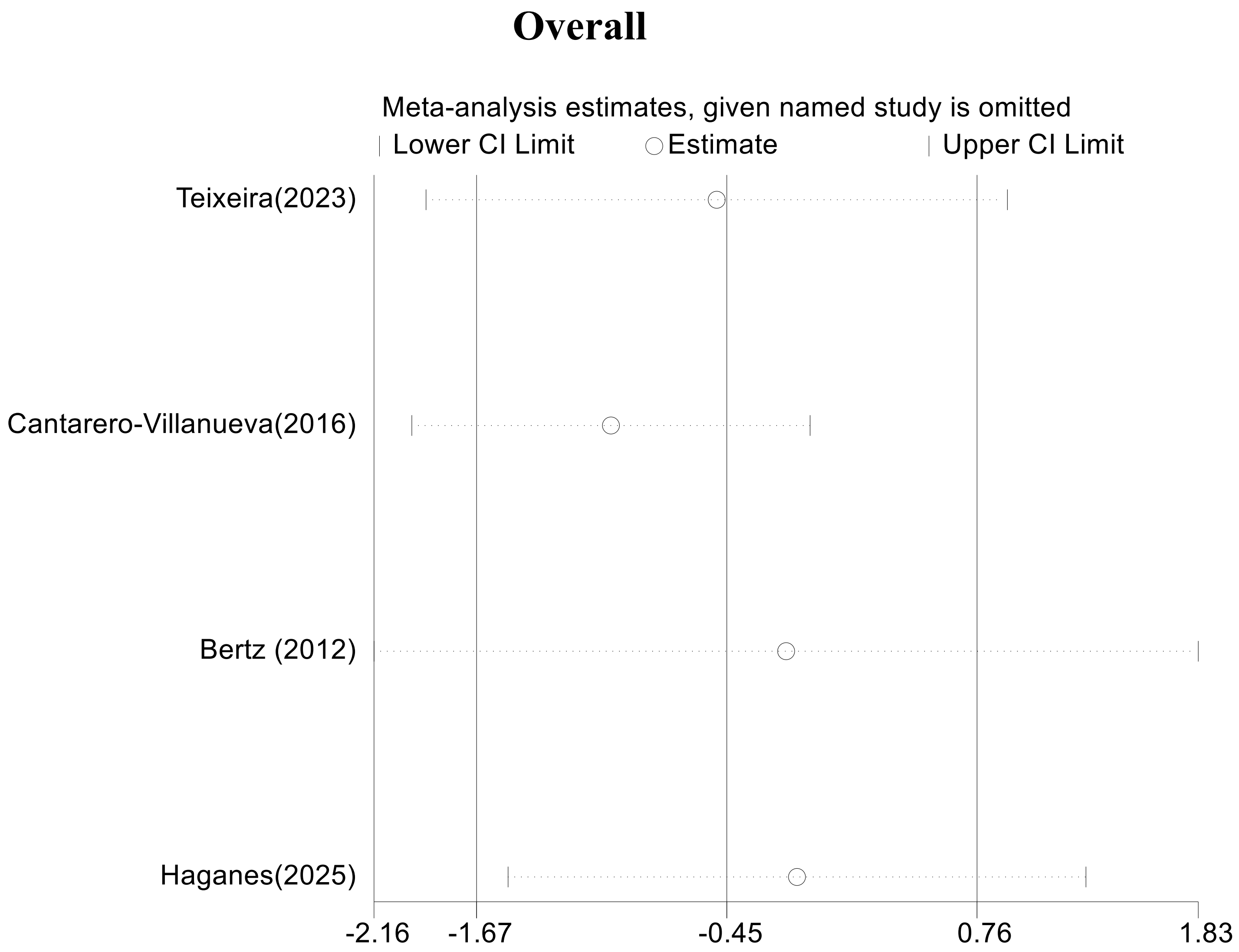

Supplementary Figure 9 Leave-one-out sensitivity analysis for Lean body mass

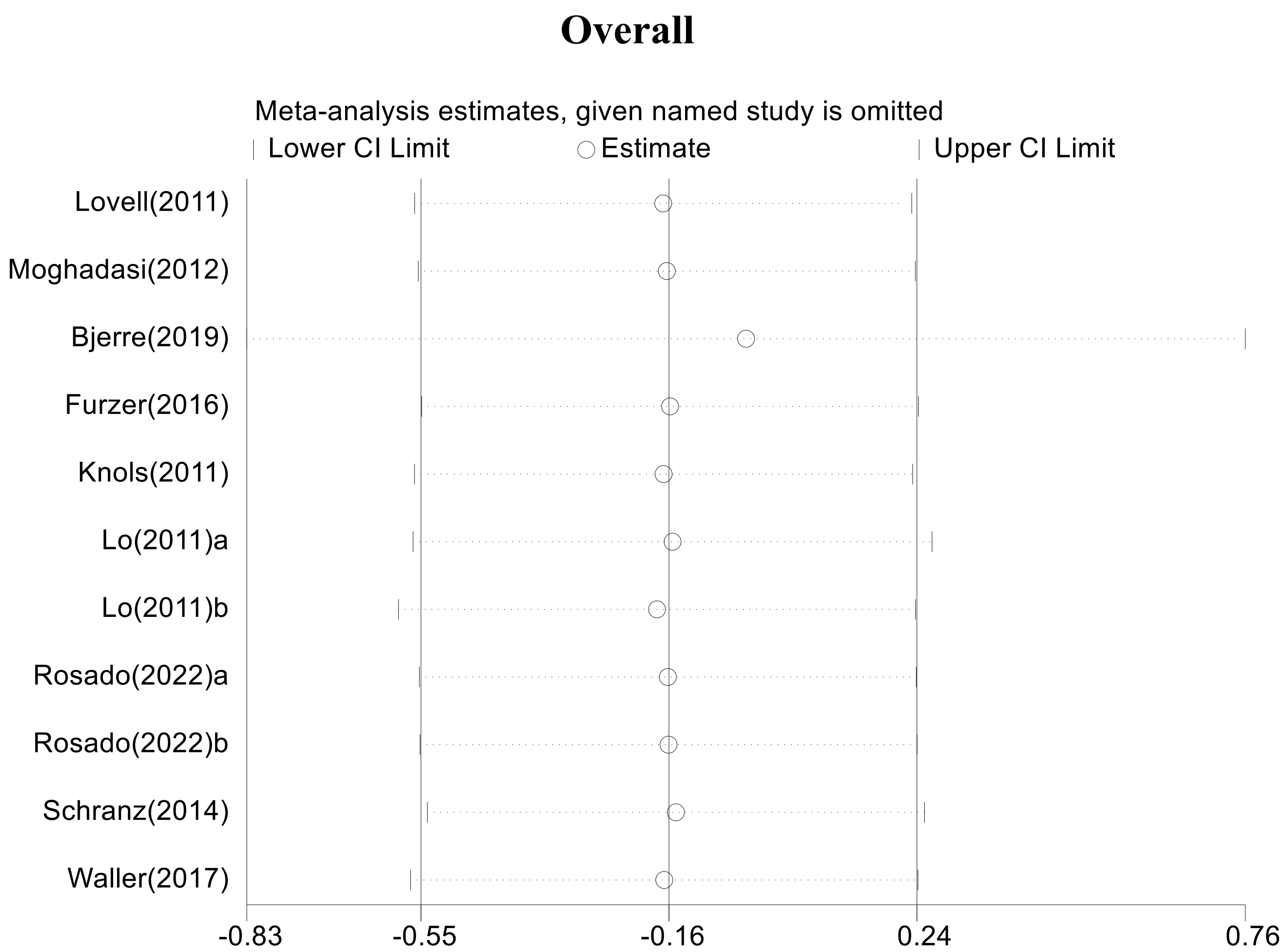

Supplementary Figure 10 Leave-one-out sensitivity analysis for Fat-free mass

Short-term subgroup

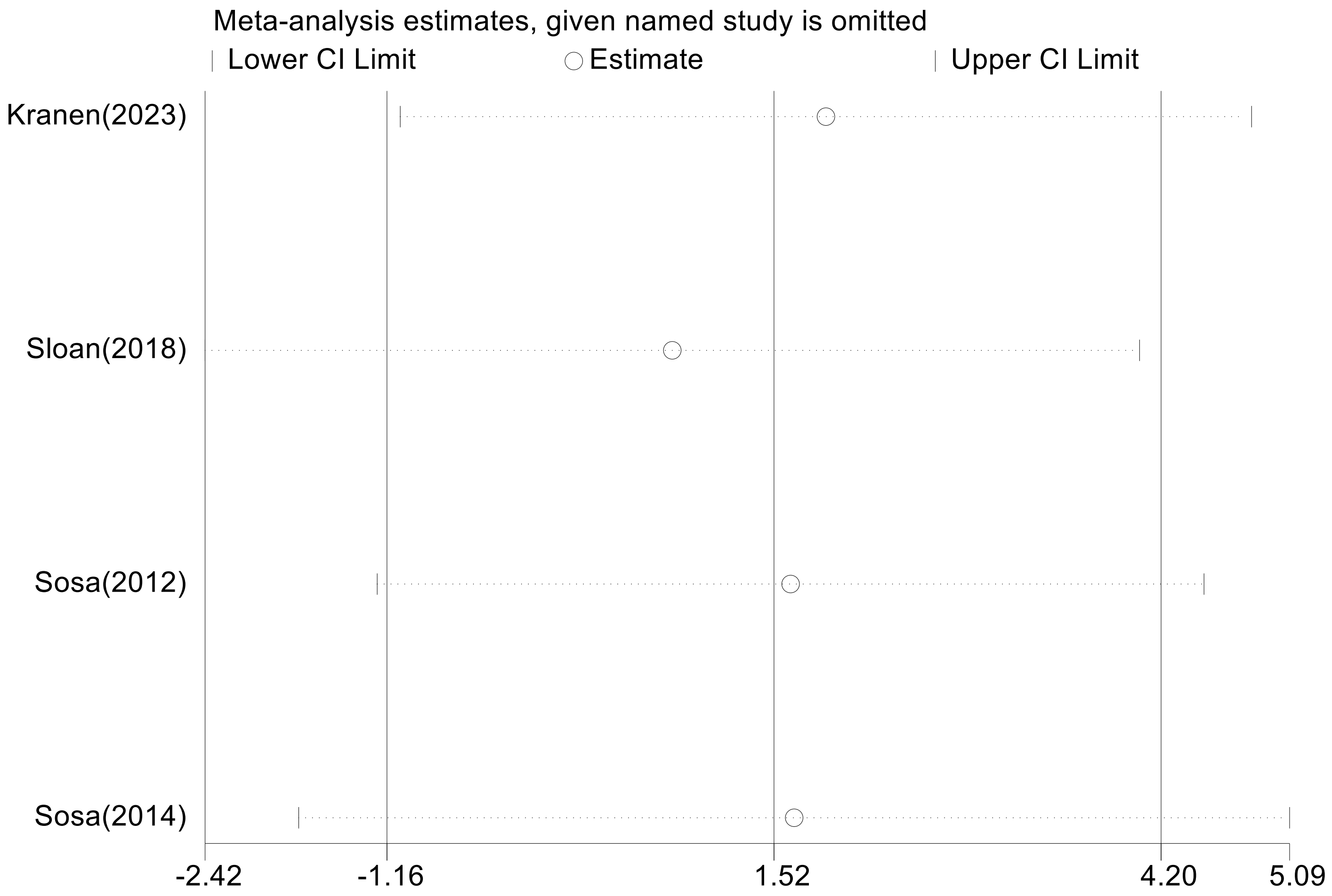

medium-term subgroup

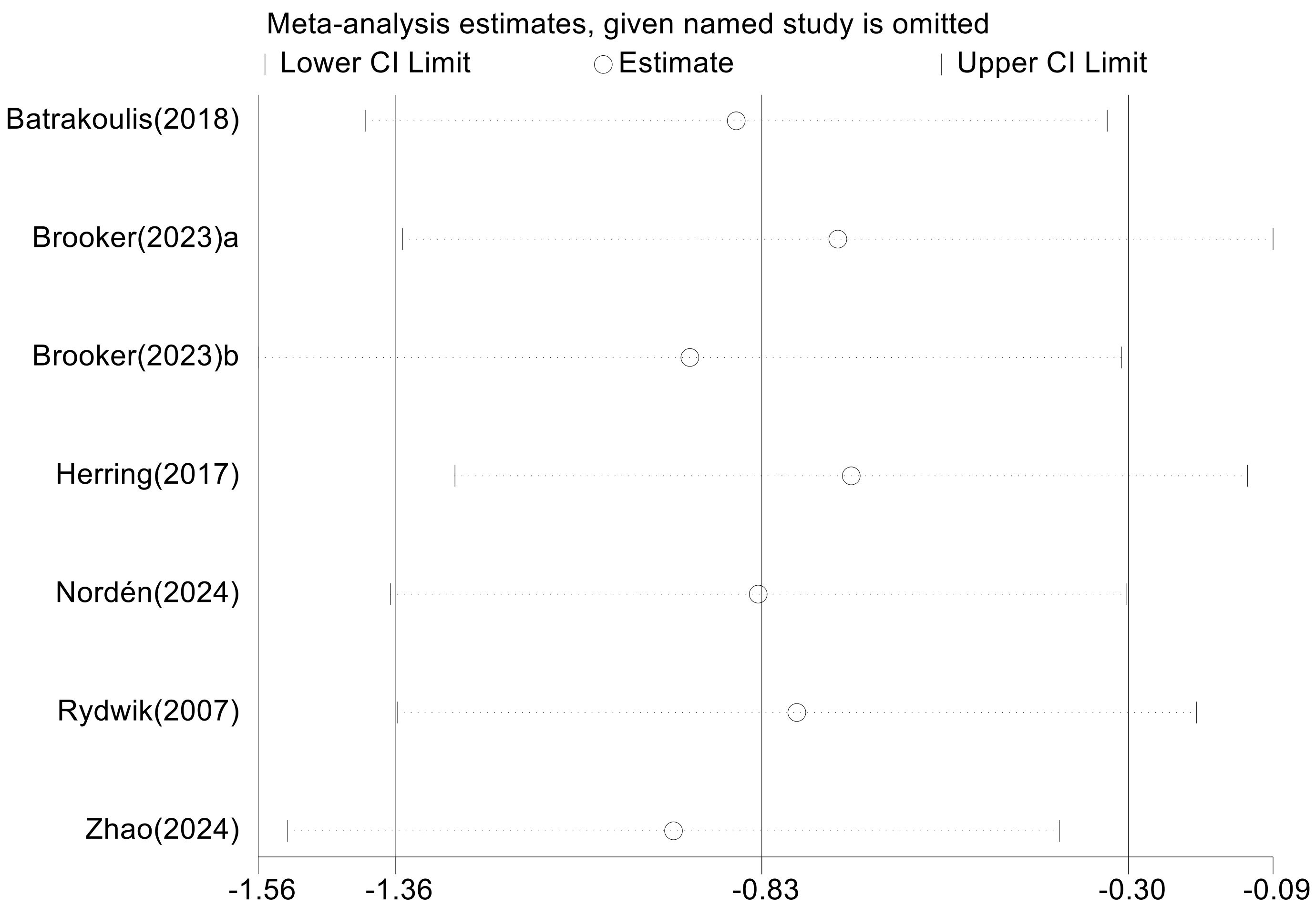

Supplementary Figure 11 Leave-one-out sensitivity analysis for Waist circumference

Short-term subgroup

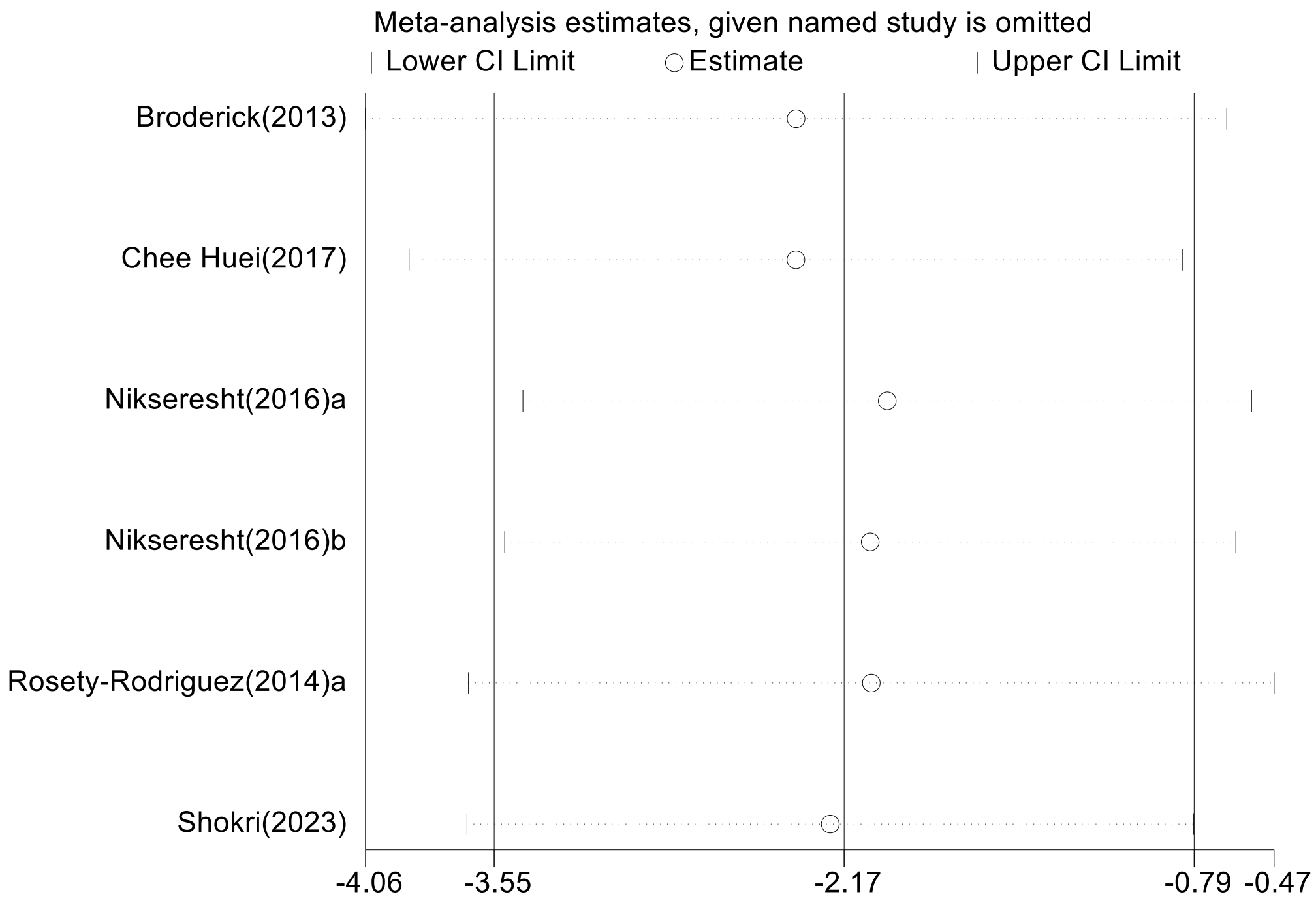

medium-term subgroup

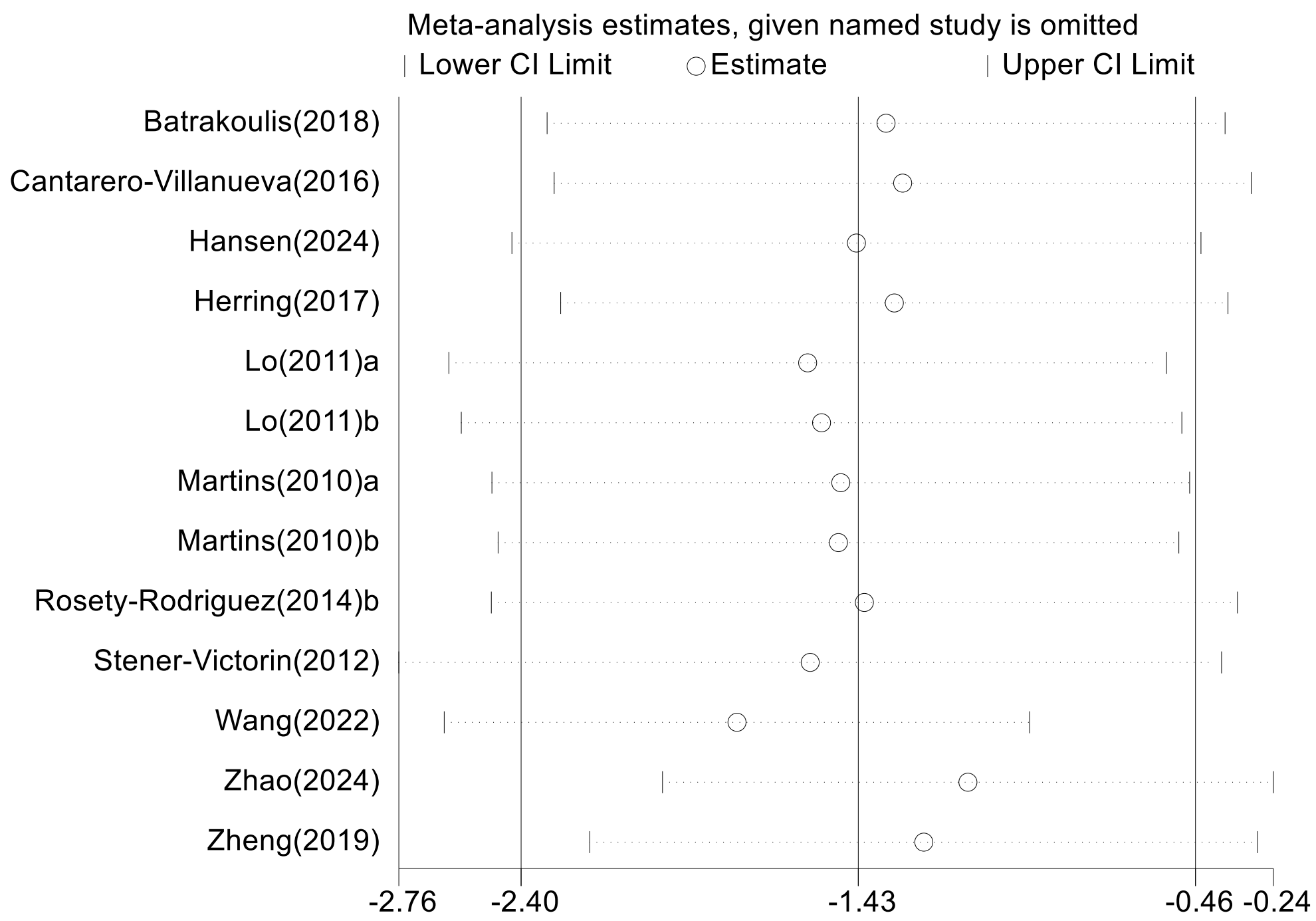

Long-term subgroup

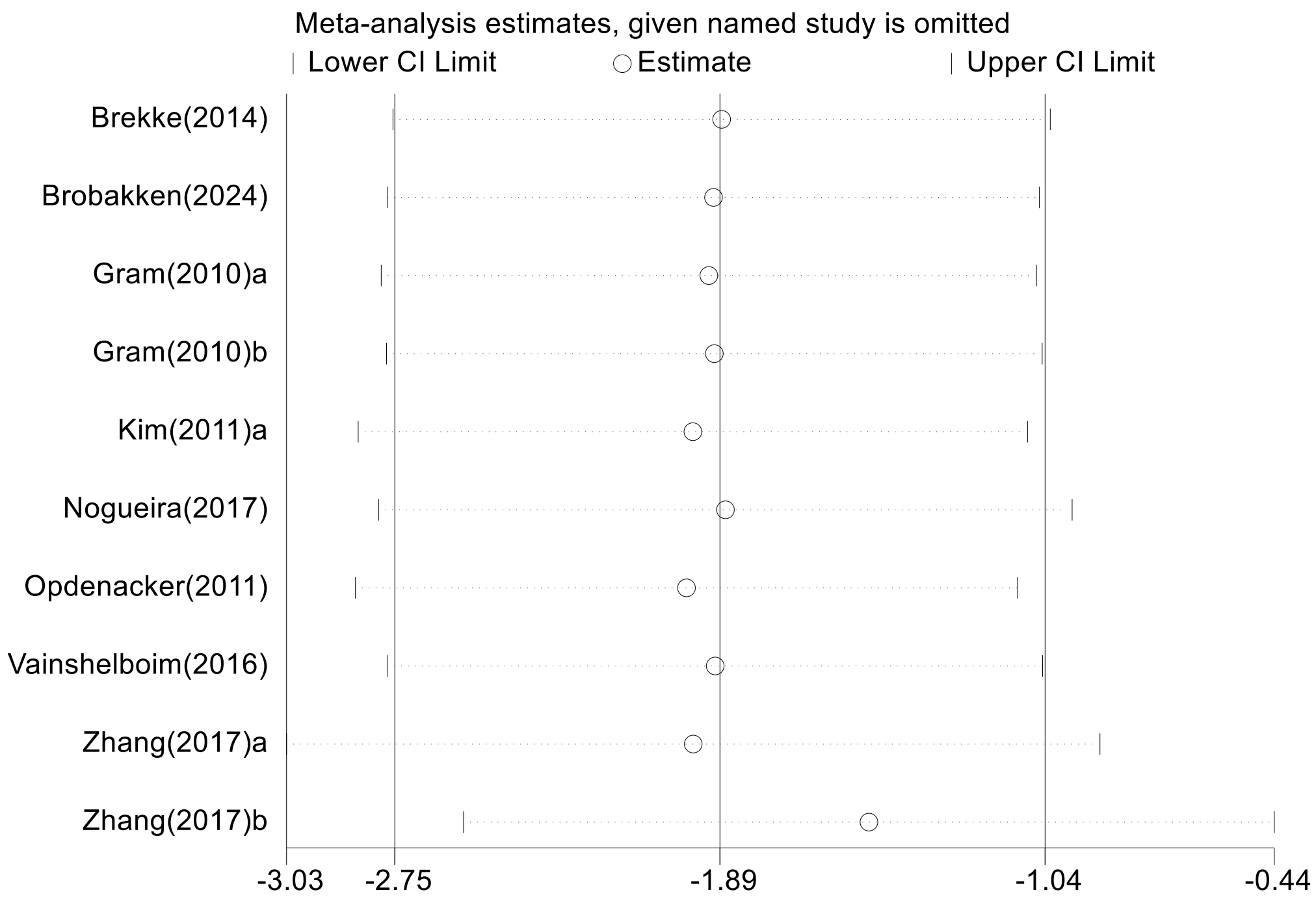

Supplementary Figure 12 Leave-one-out sensitivity analysis for Waist-to-Hip Ratio

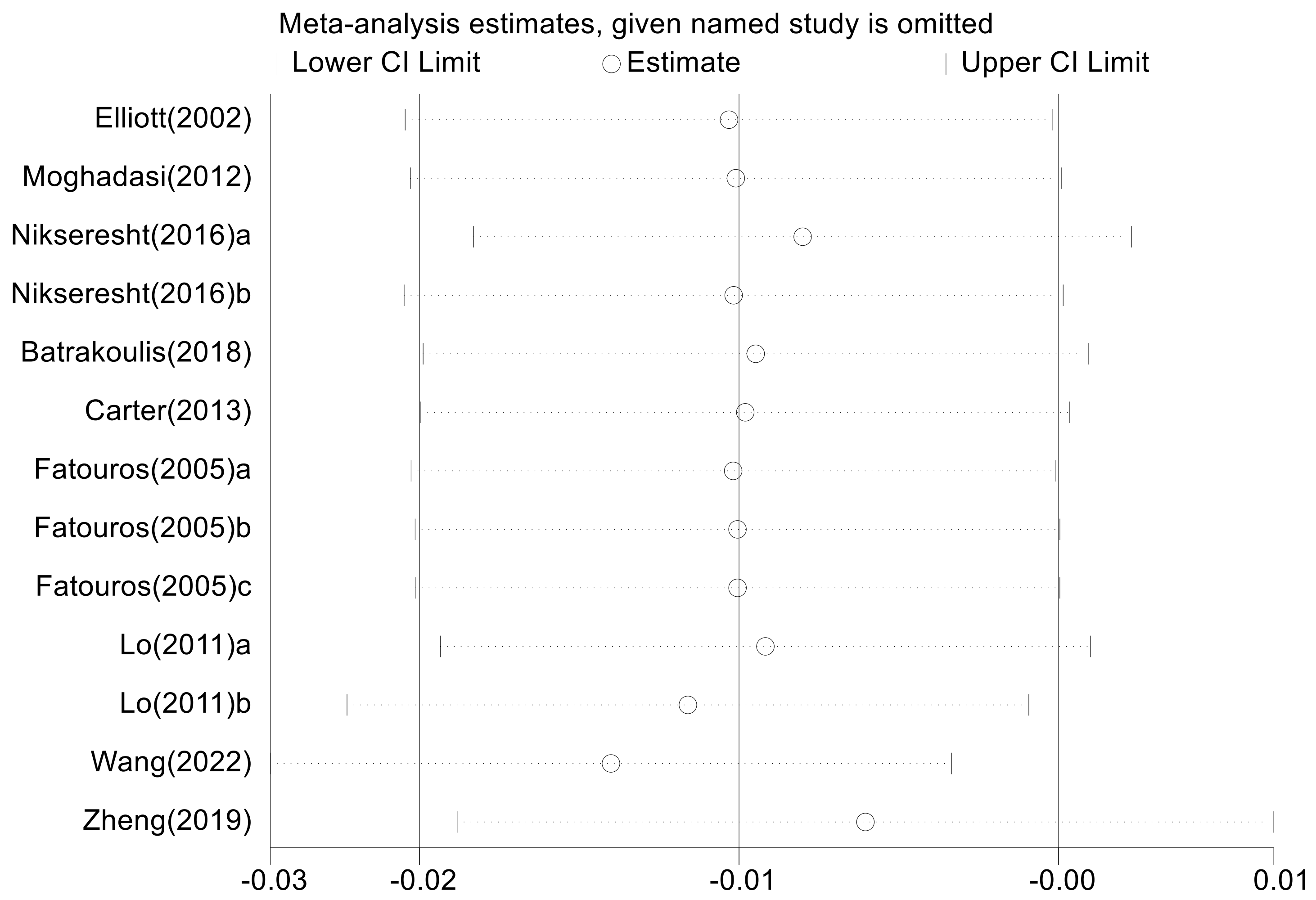

Supplementary Figure 13 Leave-one-out sensitivity analysis for Skinfold

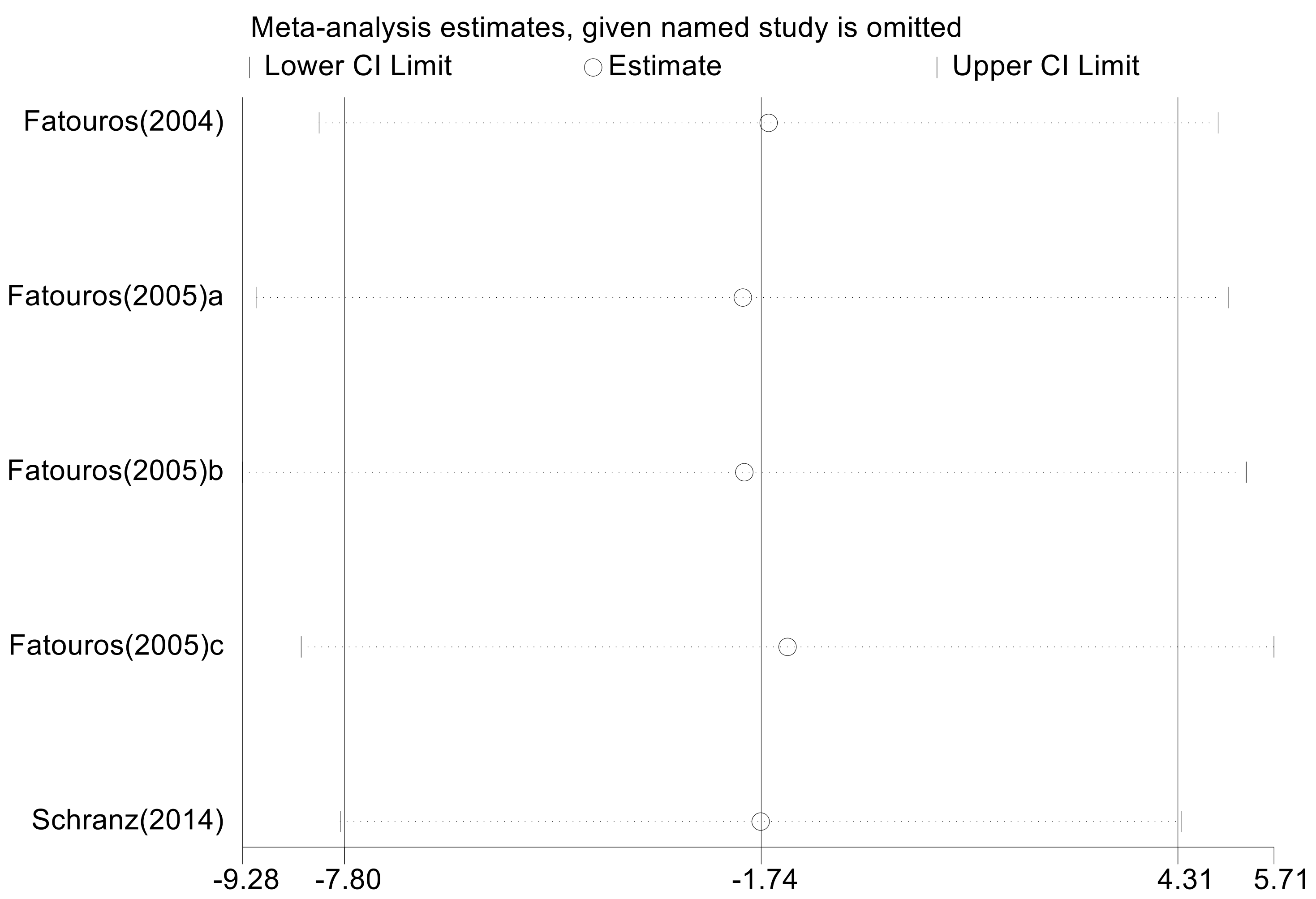

Supplementary Figure 14 Leave-one-out sensitivity analysis for Systolic blood pressure

Short-term subgroup

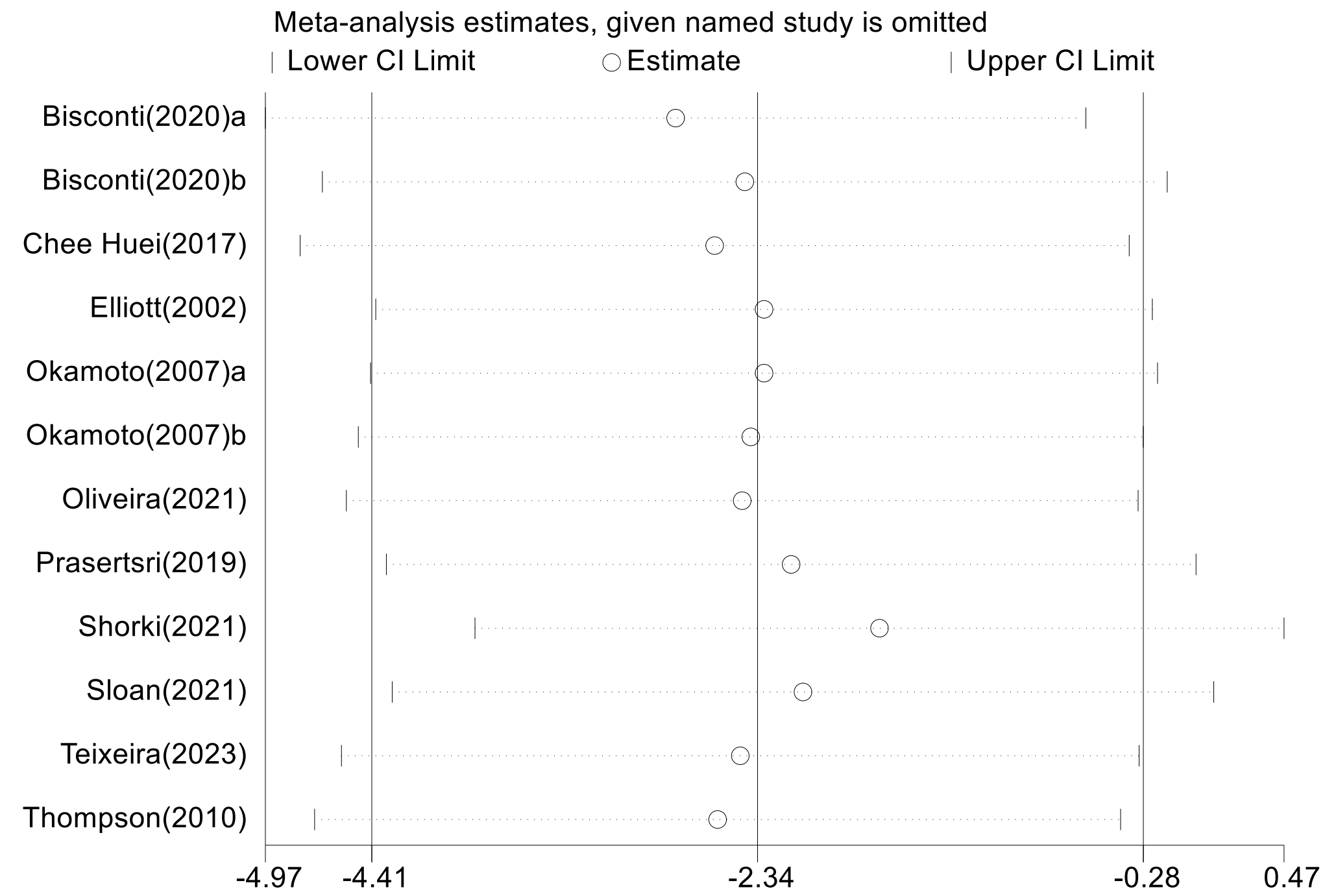

medium-term subgroup

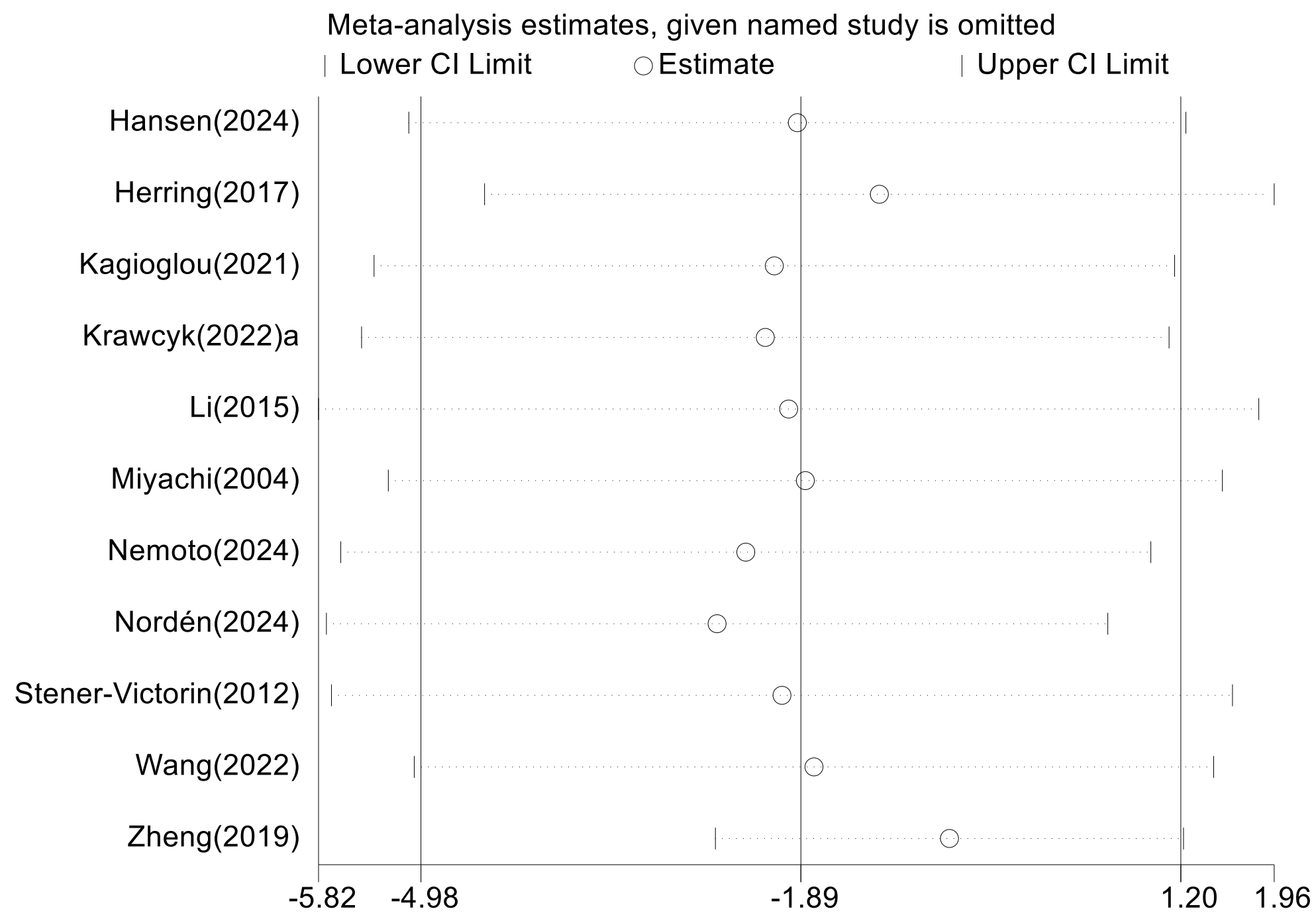

Long-term subgroup

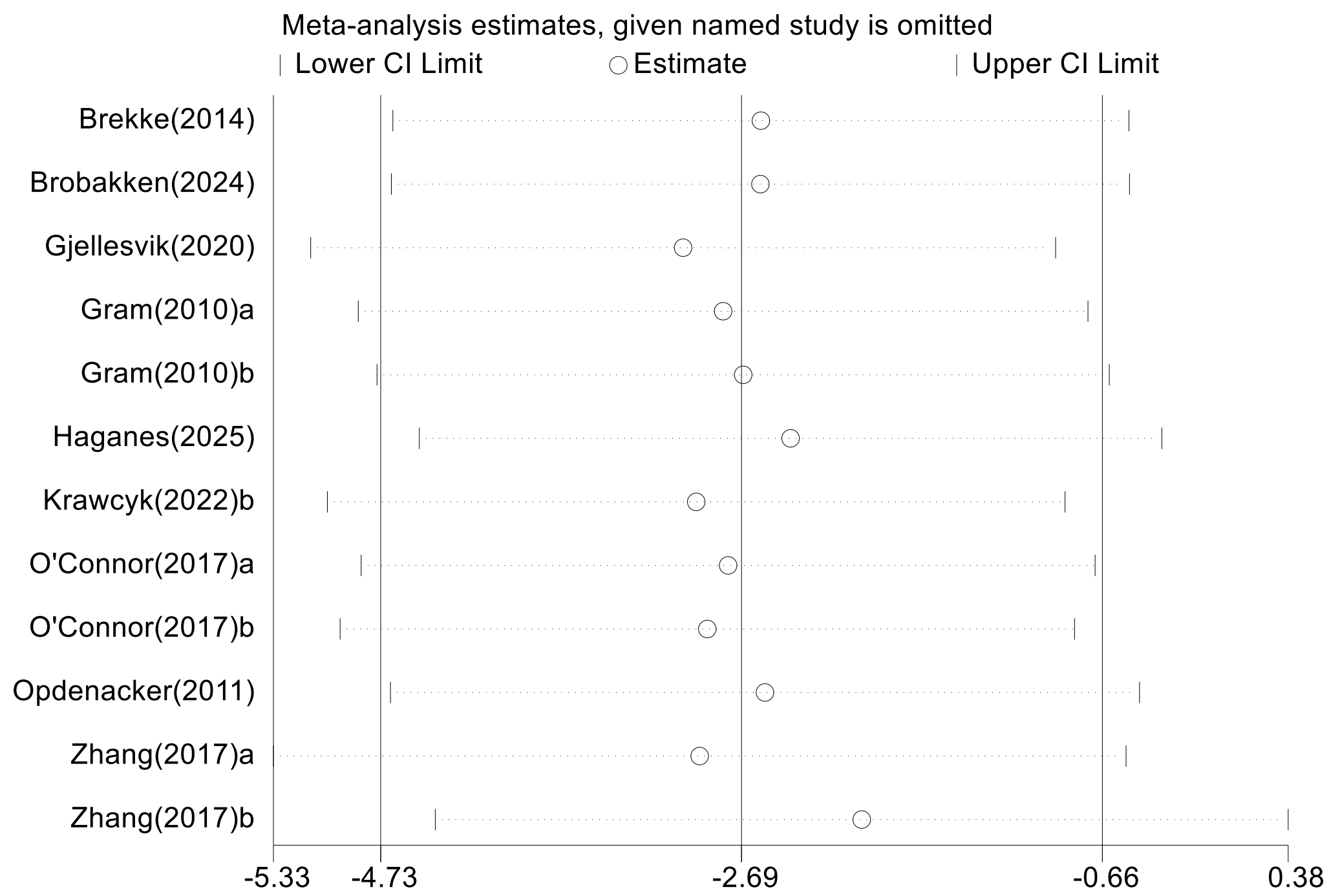

Supplementary Figure 15 Leave-one-out sensitivity analysis for Diastolic blood pressure

Short-term subgroup

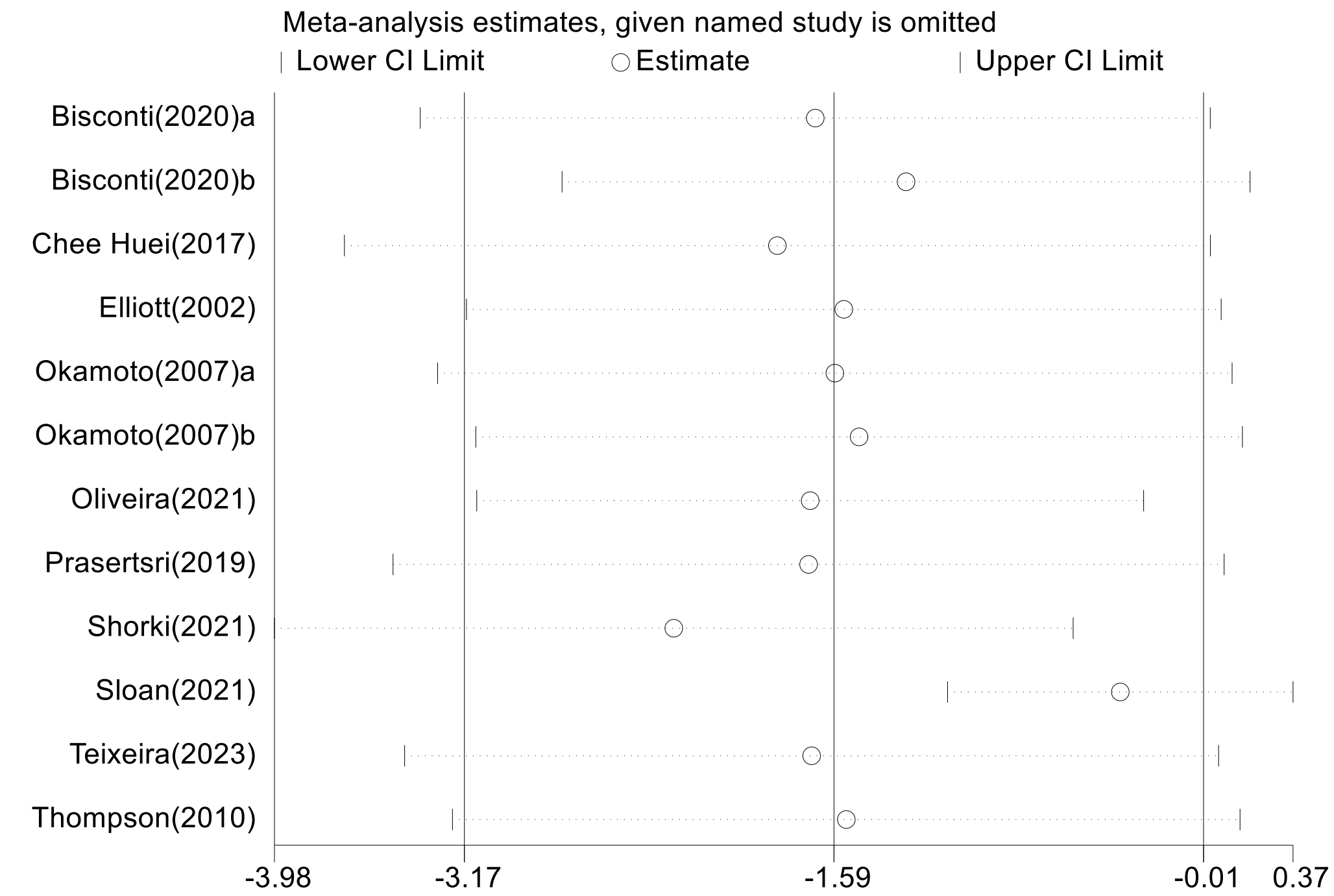

medium-term subgroup

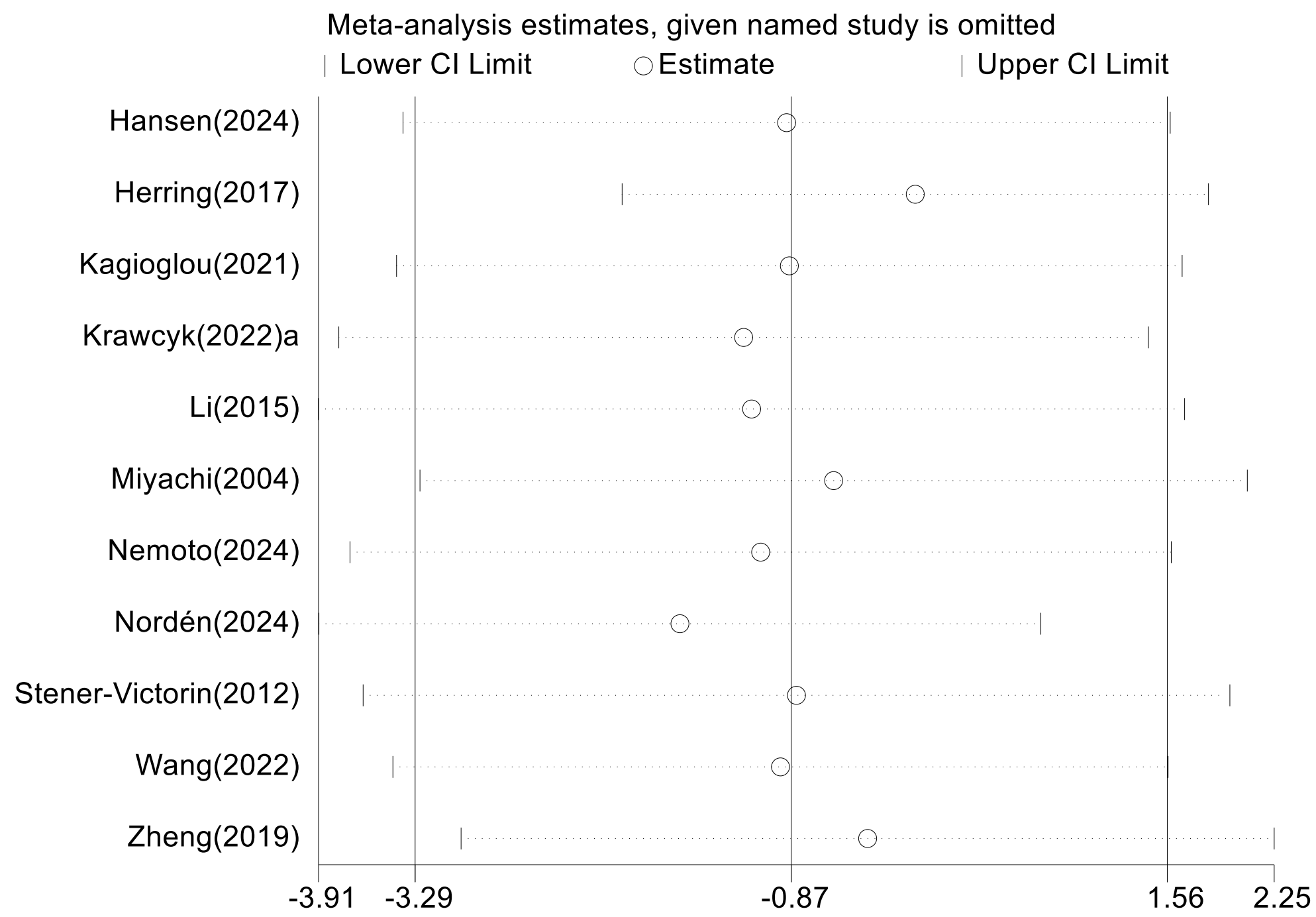

Long-term subgroup

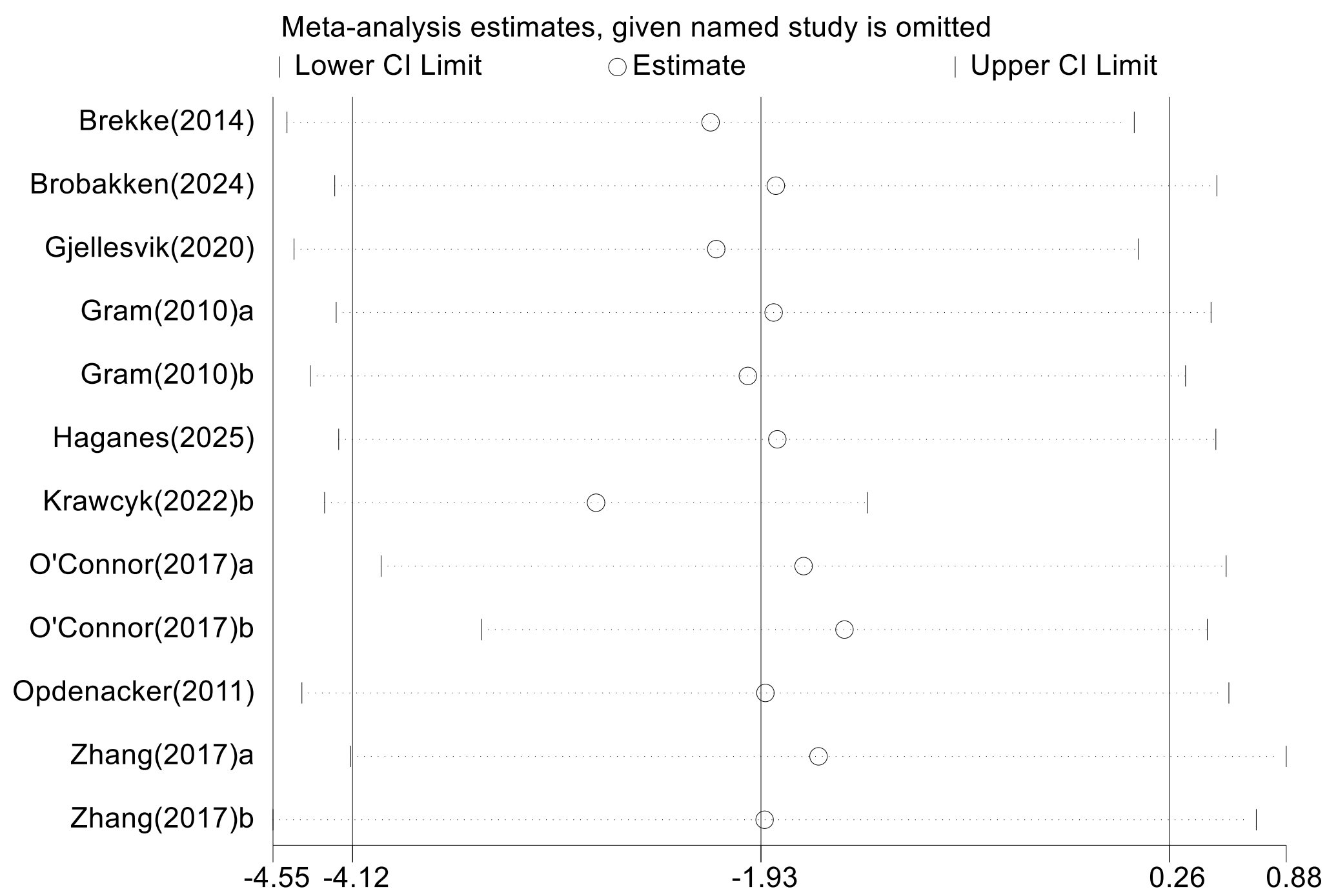

Supplementary Figure 16 Leave-one-out sensitivity analysis for Mean arterial pressure

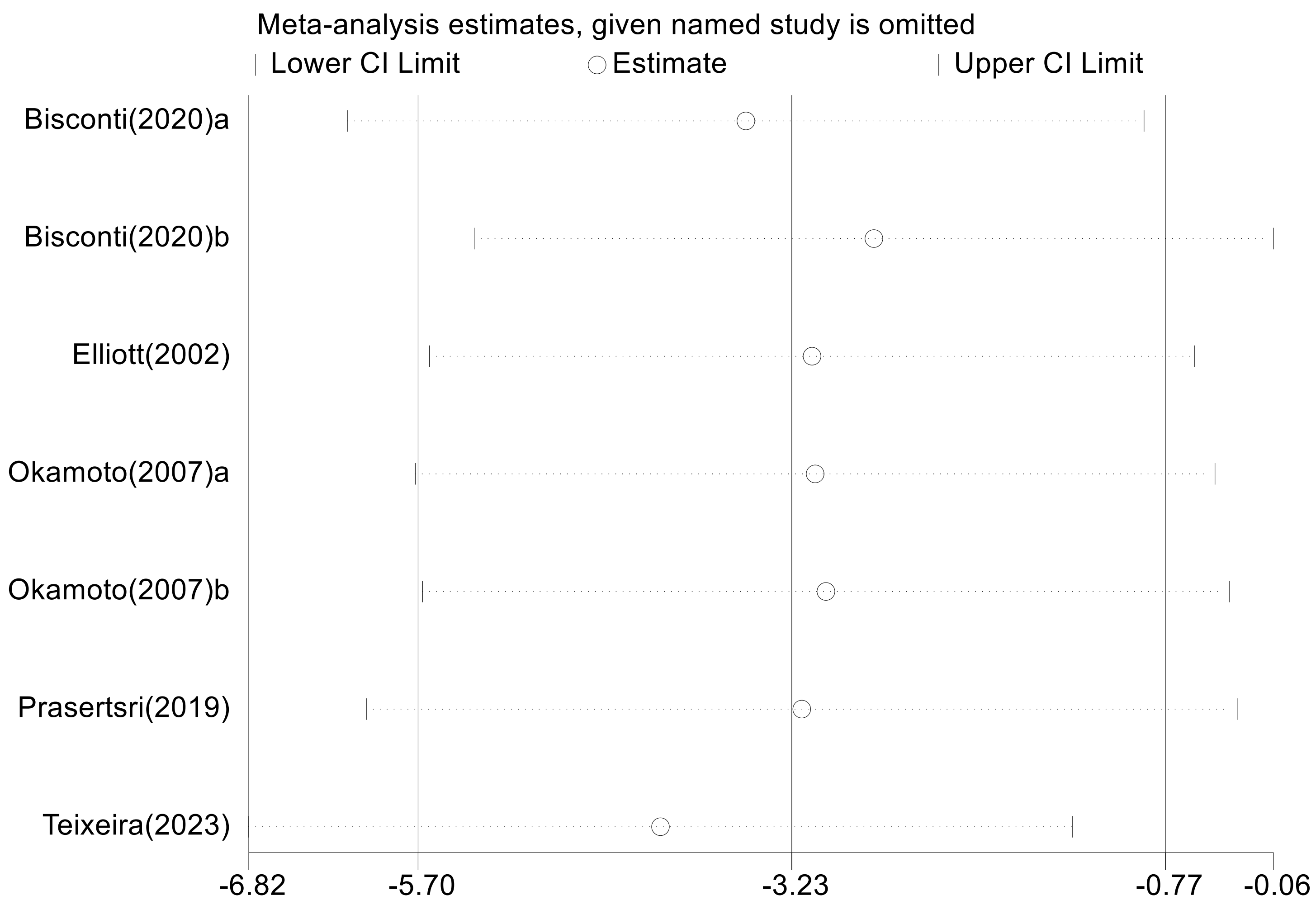

Supplementary Figure 17 Leave-one-out sensitivity analysis for Resting heart rate

Short-term subgroup

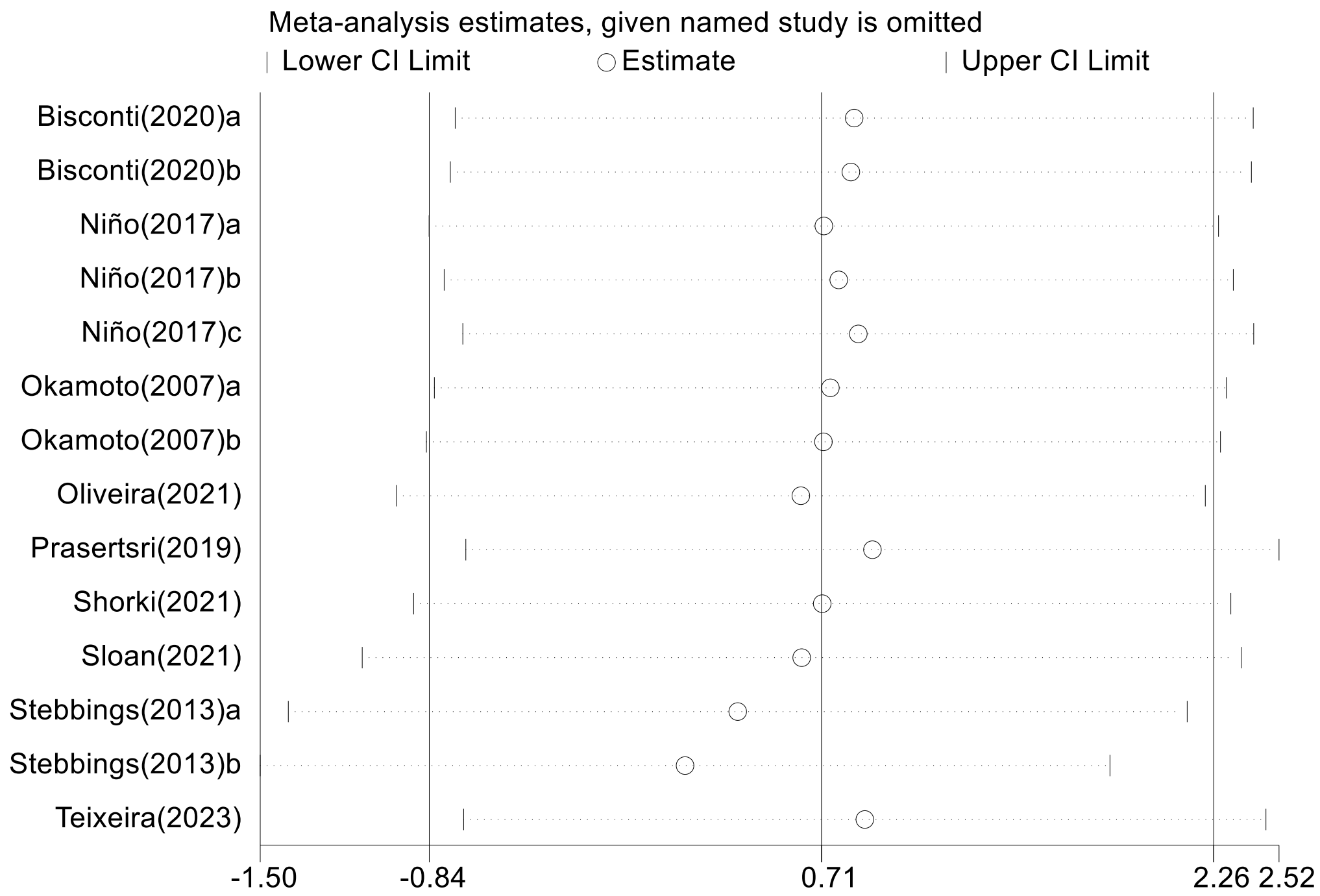

medium-term subgroup

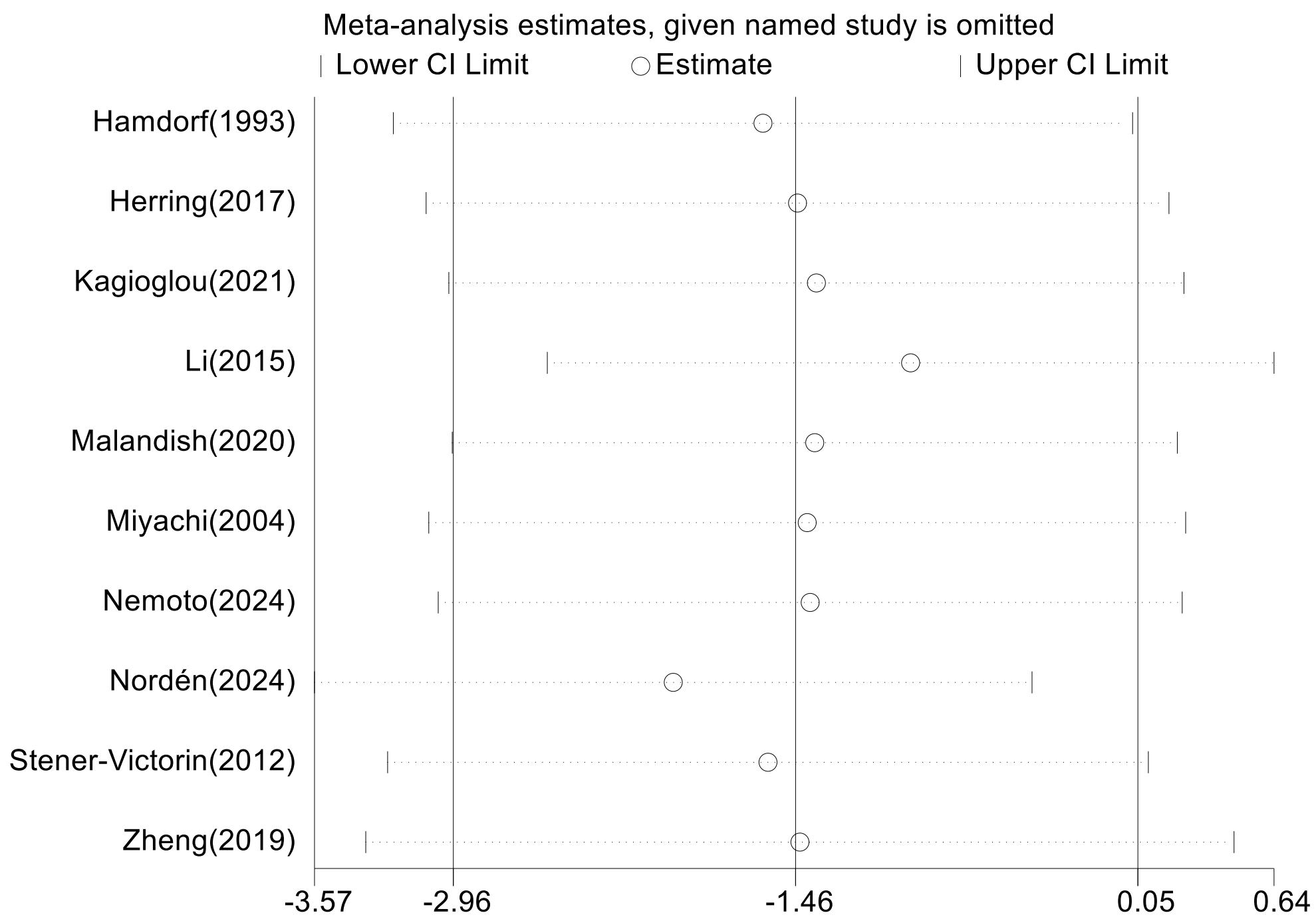

Supplementary Figure 18 Leave-one-out sensitivity analysis for VO_{2peak}

Short-term subgroup

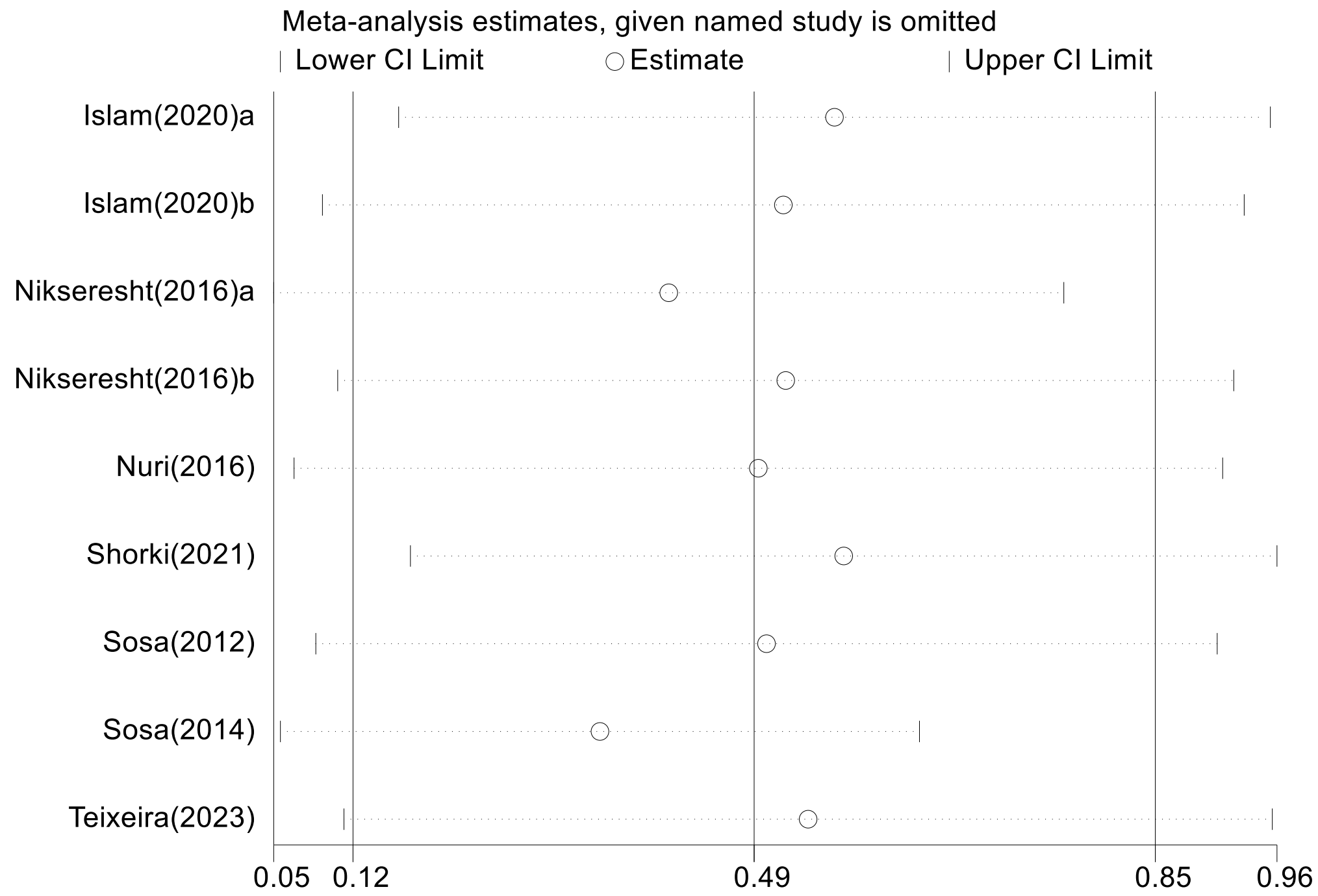

medium-term subgroup

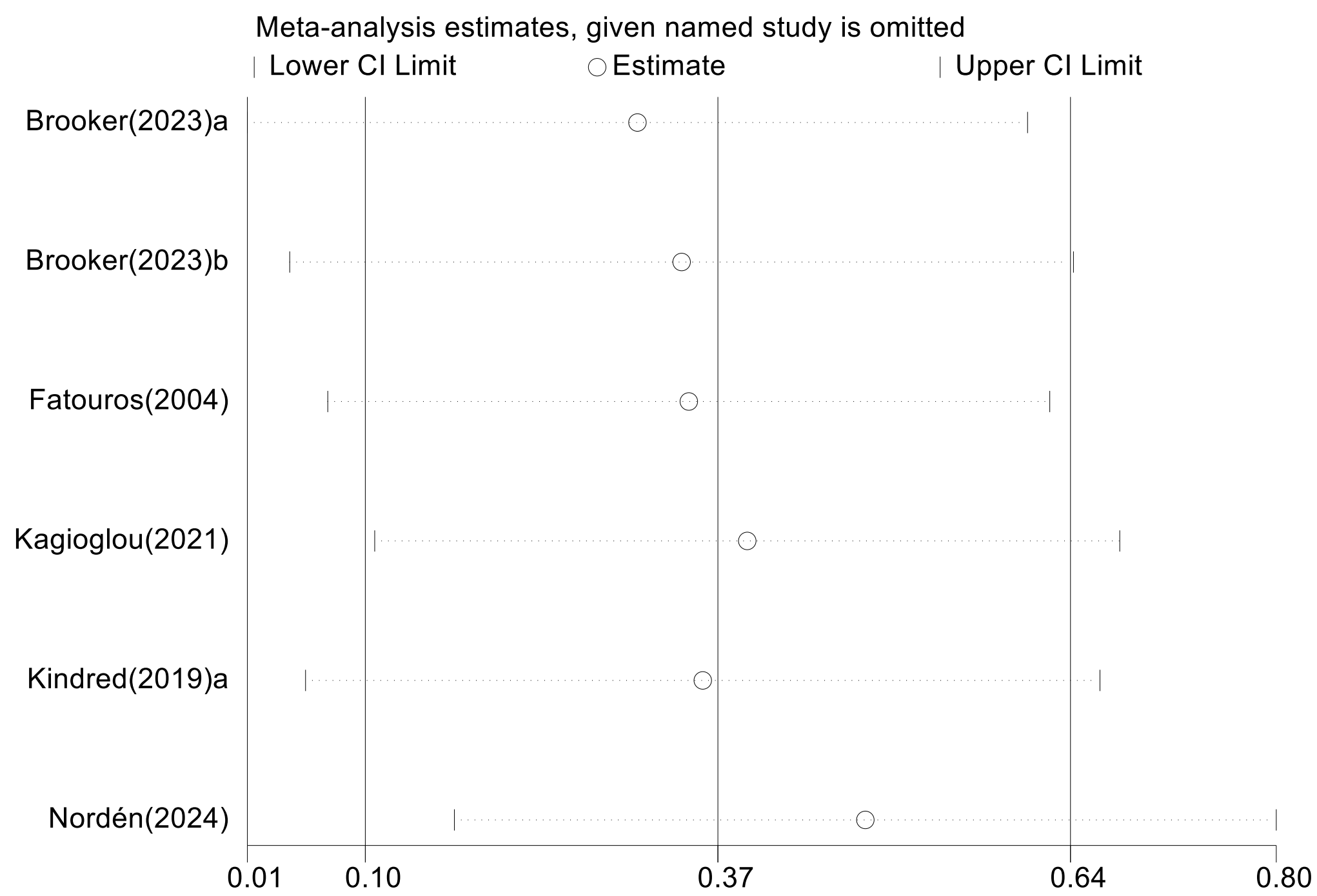

Long-term subgroup

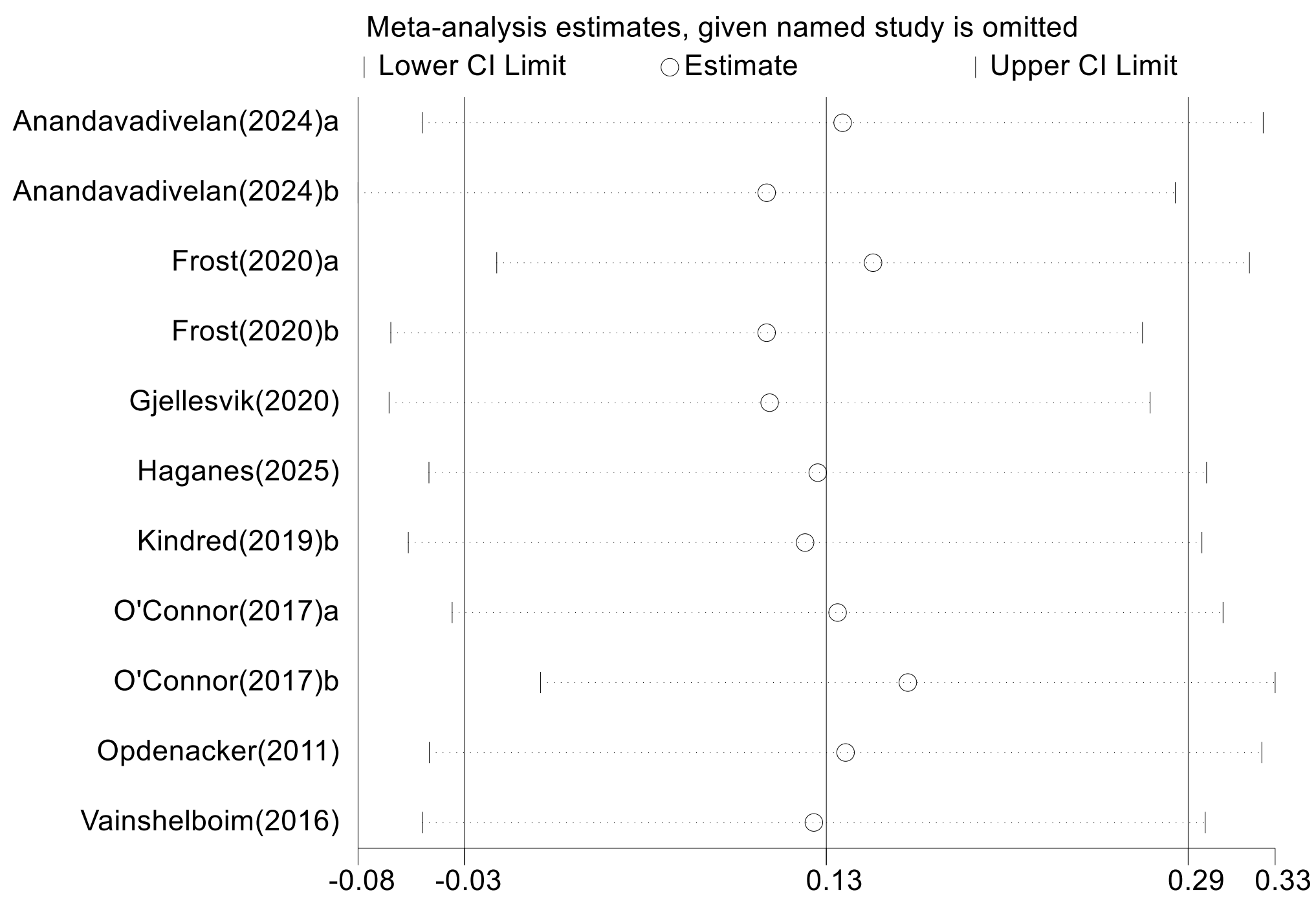

Supplementary Figure 19 Leave-one-out sensitivity analysis for VO_{2max}

Short-term subgroup

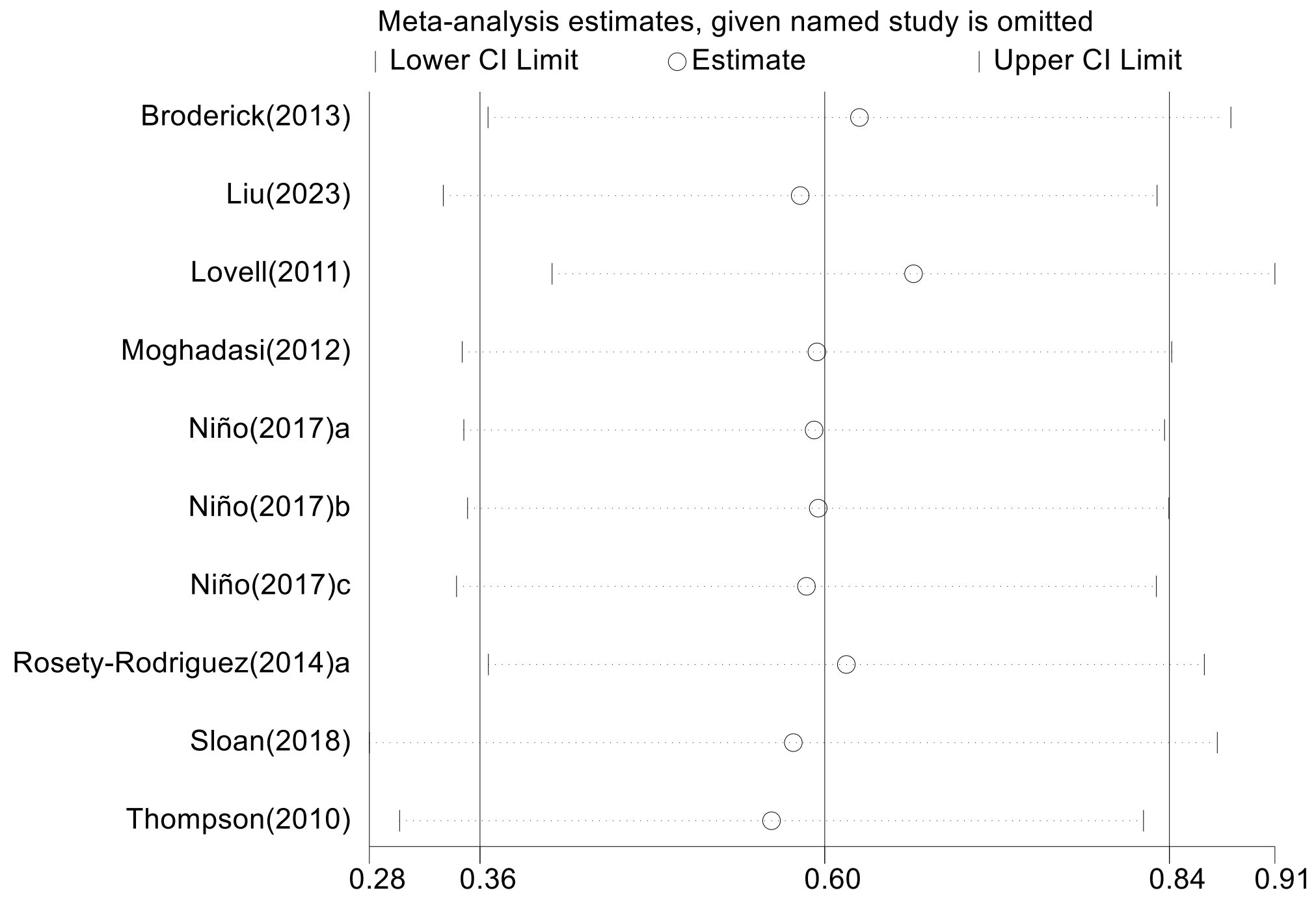

medium-term subgroup

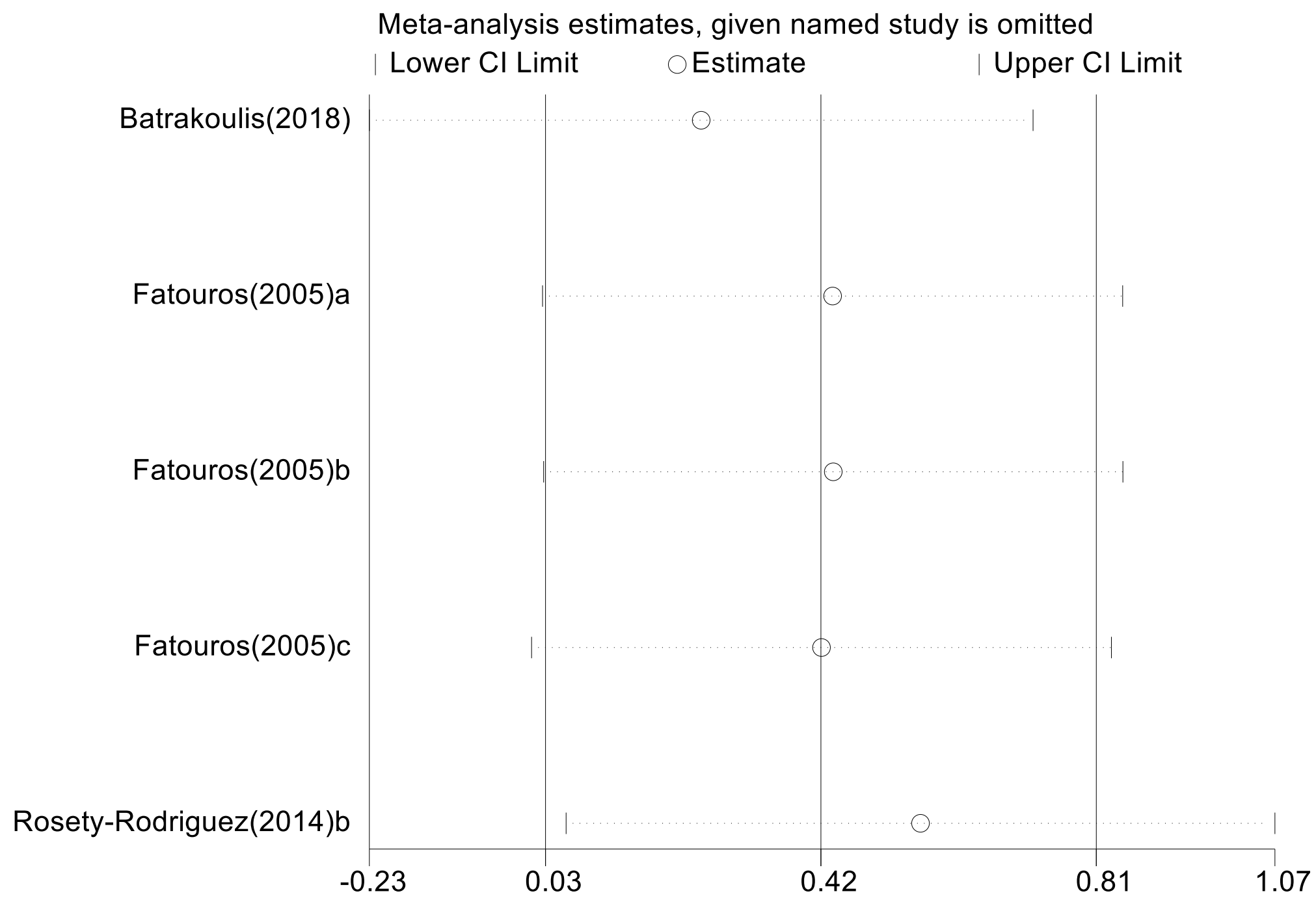

Long-term subgroup

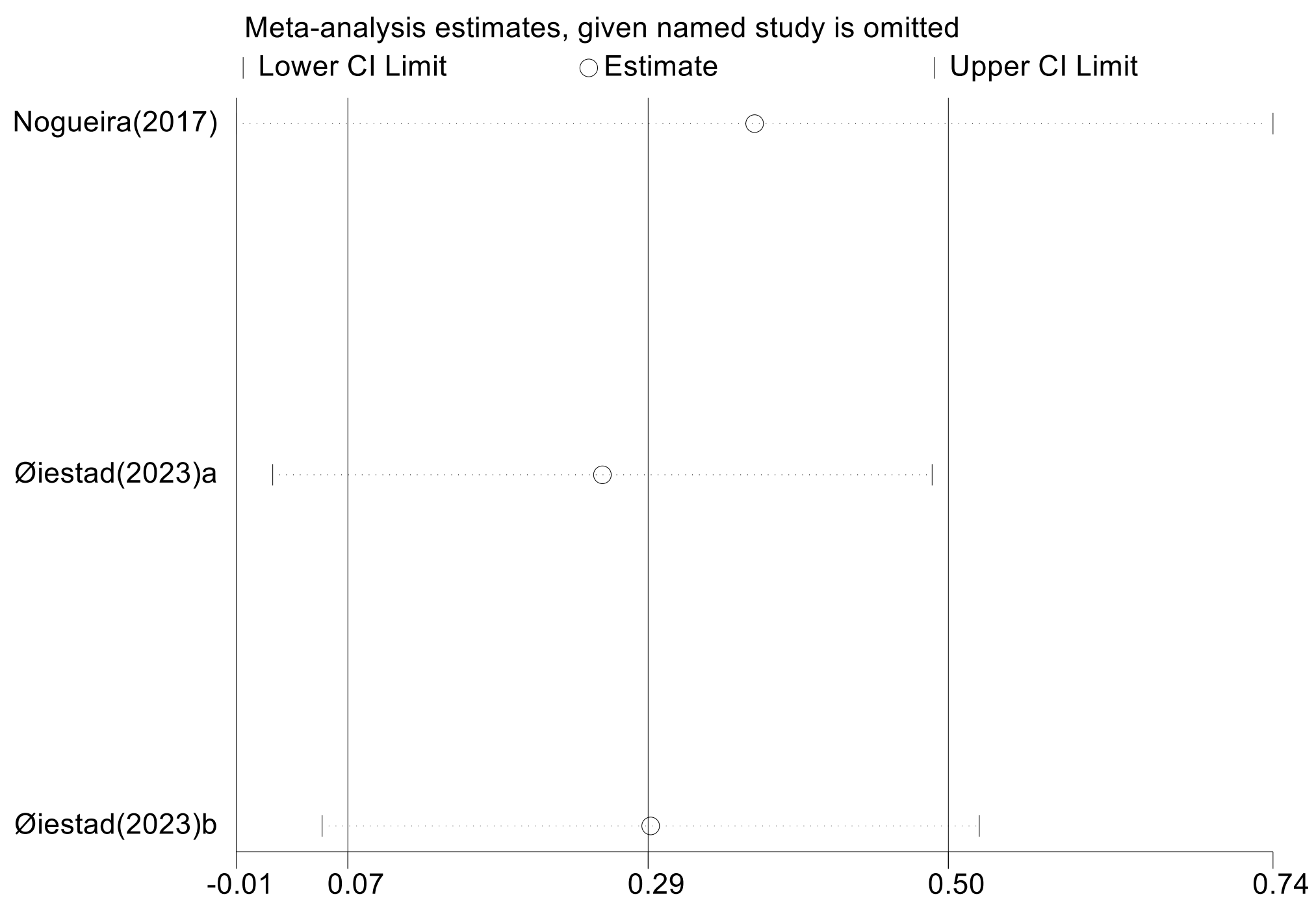

Supplementary Figure 20 Leave-one-out sensitivity analysis for Total cholesterol

Short-term subgroup

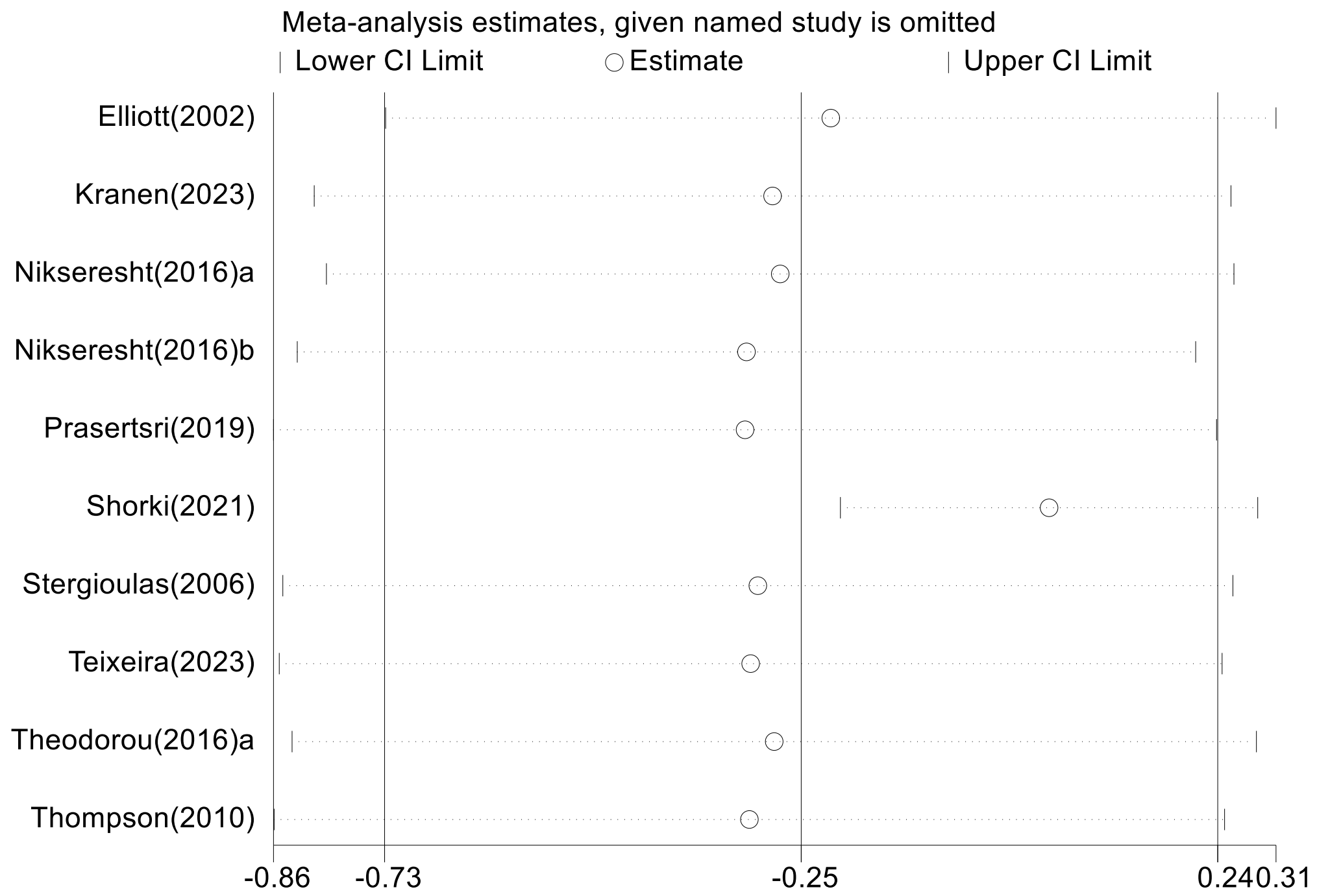

medium-term subgroup

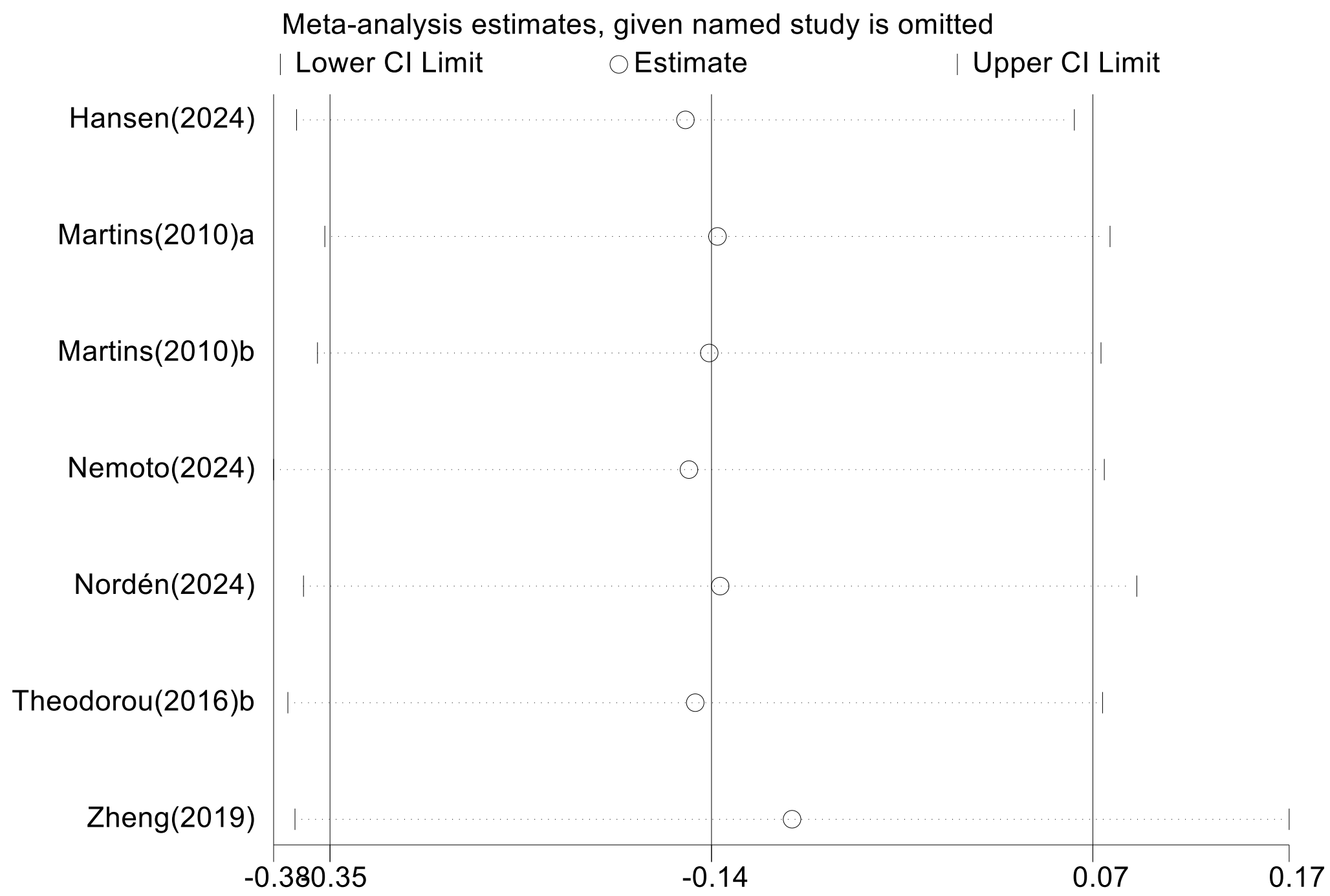

Long-term subgroup

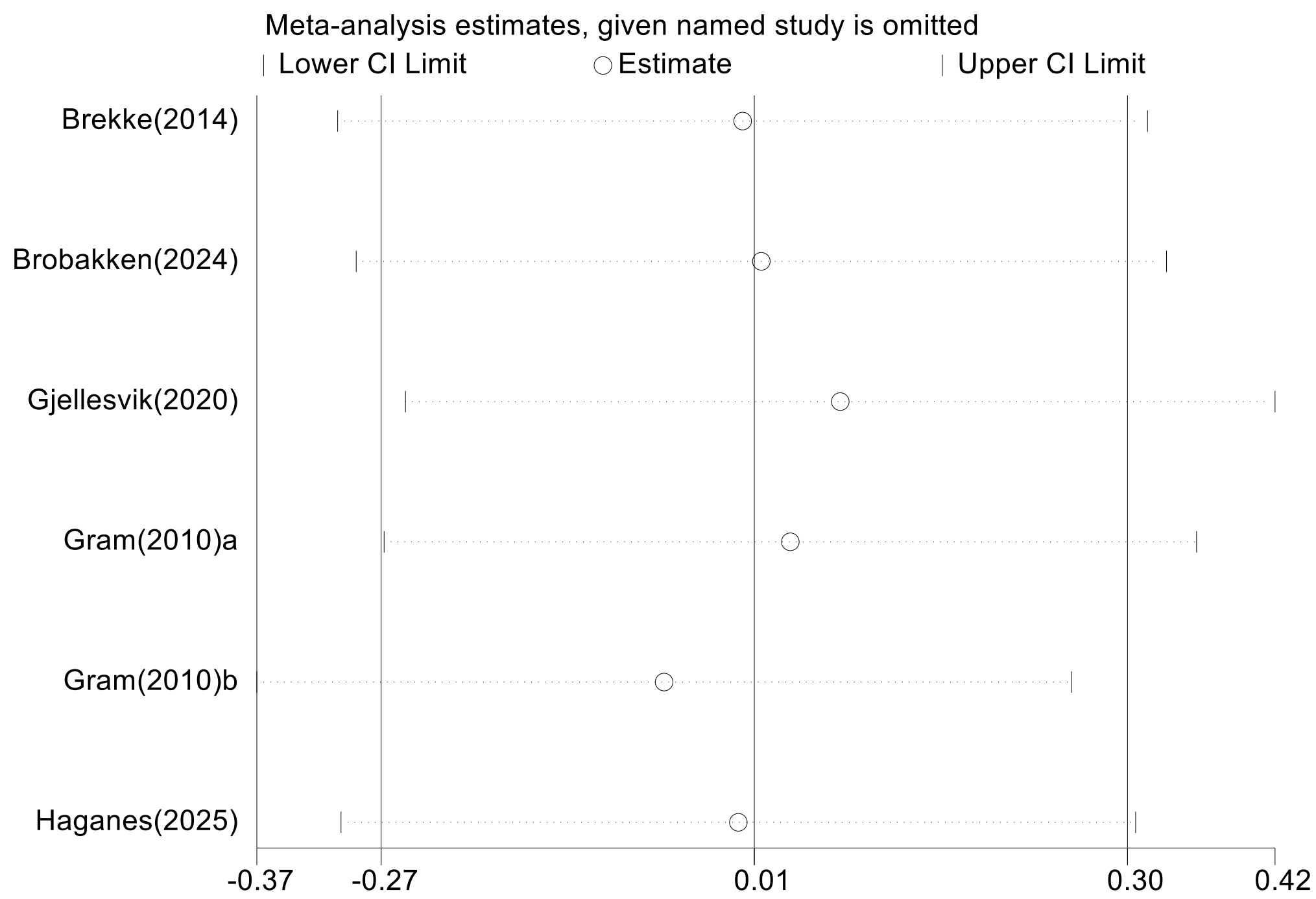

Supplementary Figure 21 Leave-one-out sensitivity analysis for HDL-C

Short-term subgroup

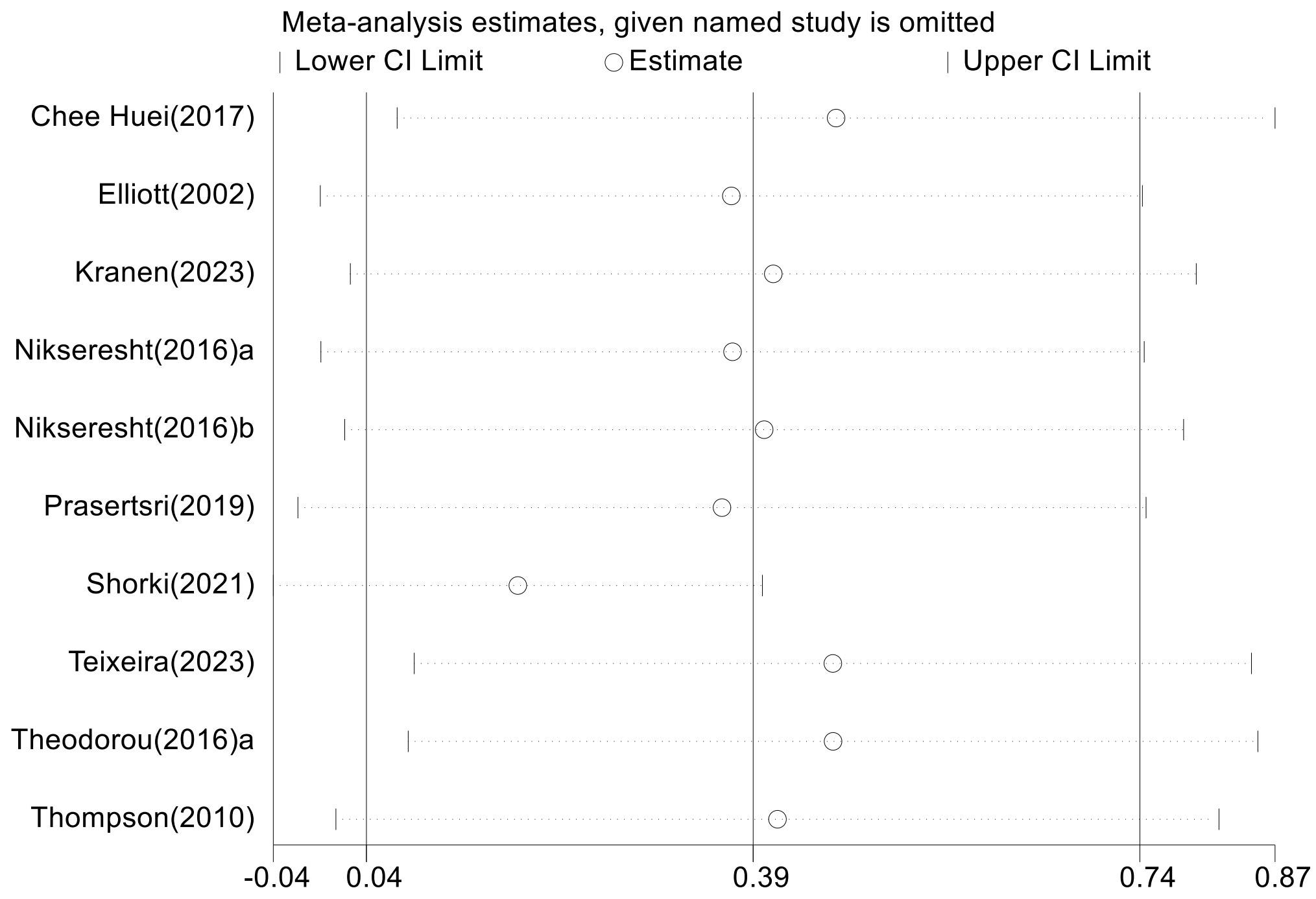

medium-term subgroup

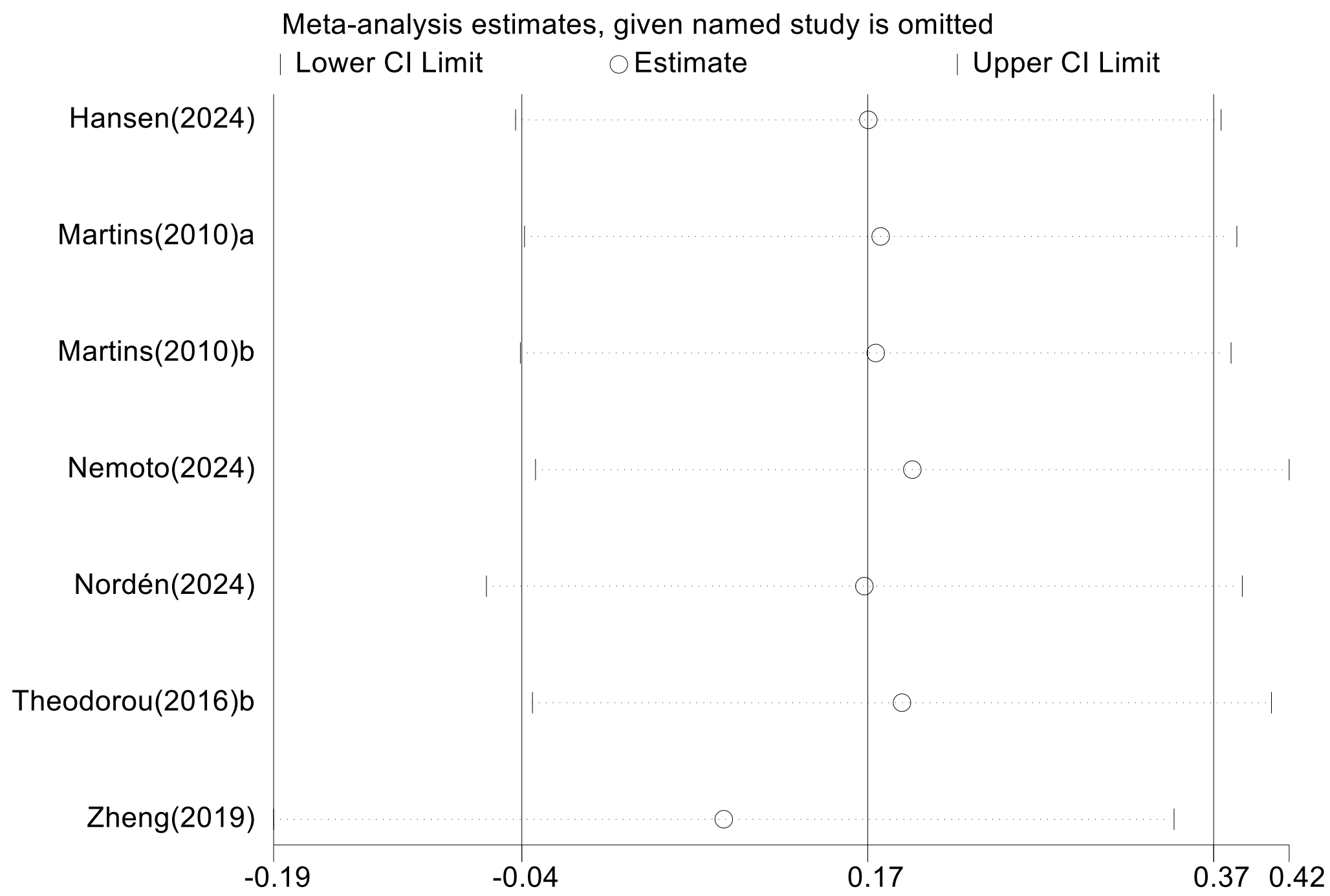

Long-term subgroup

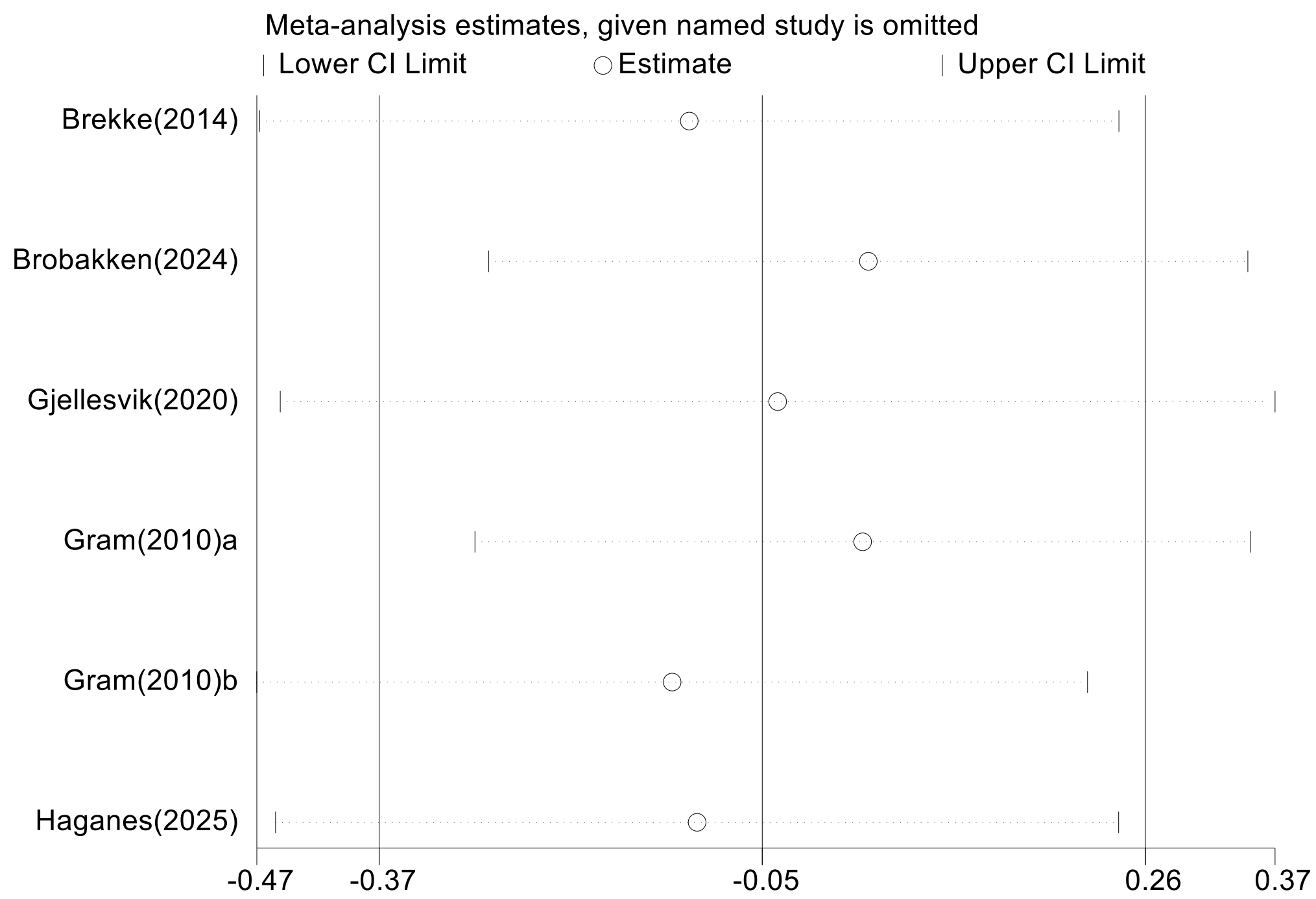

Supplementary Figure 22 Leave-one-out sensitivity analysis for LDL-C

Short-term subgroup

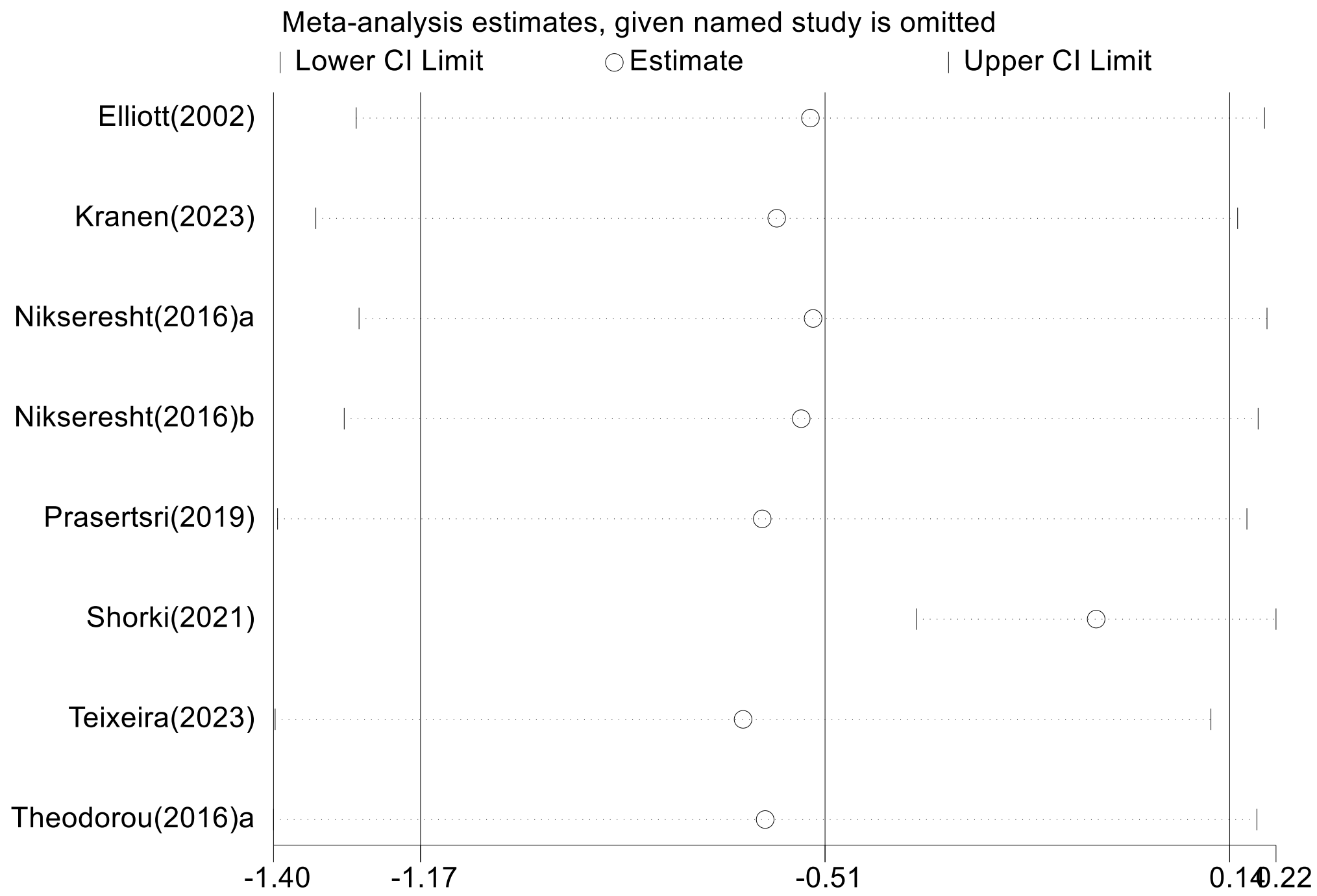

medium-term subgroup

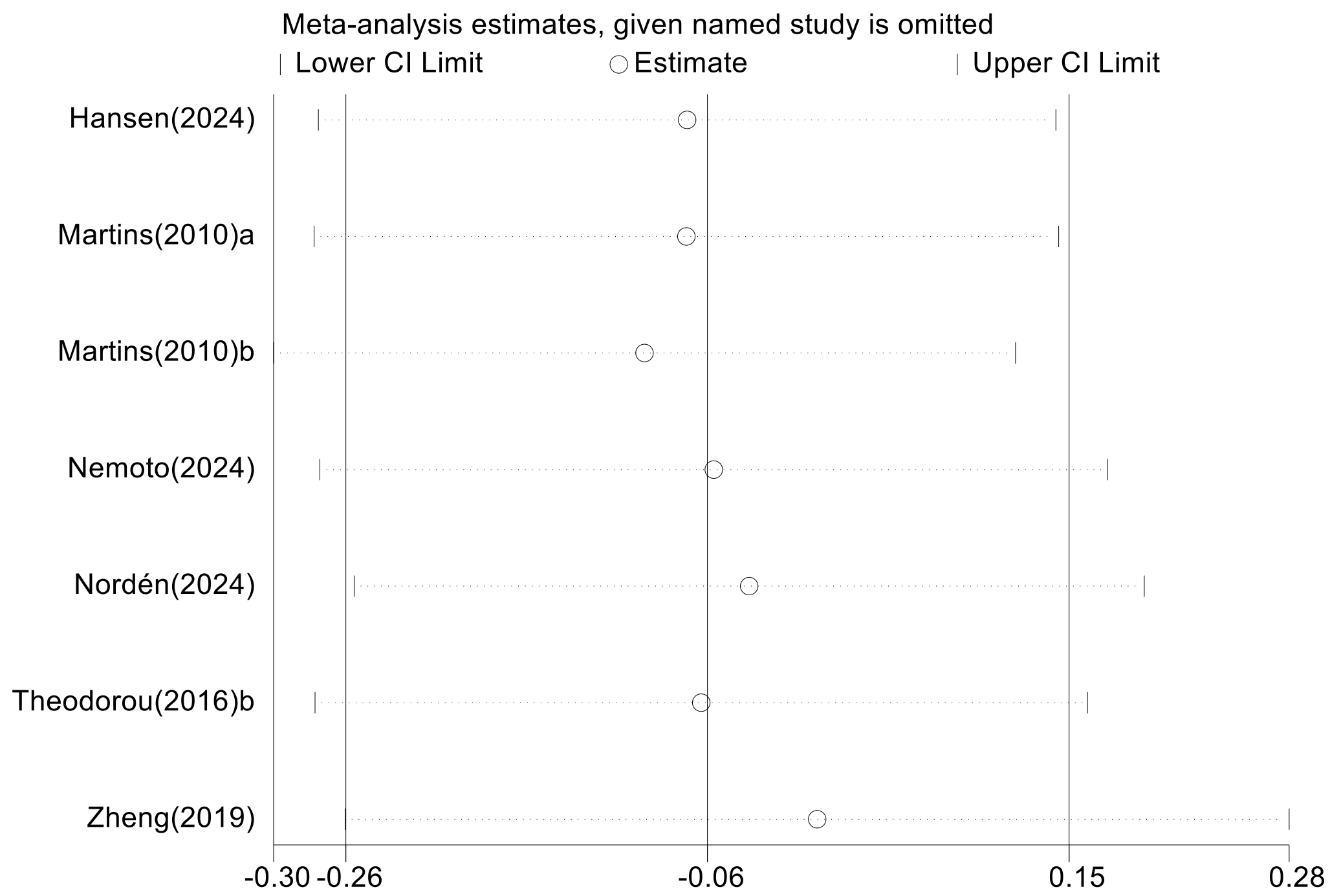

Long-term subgroup

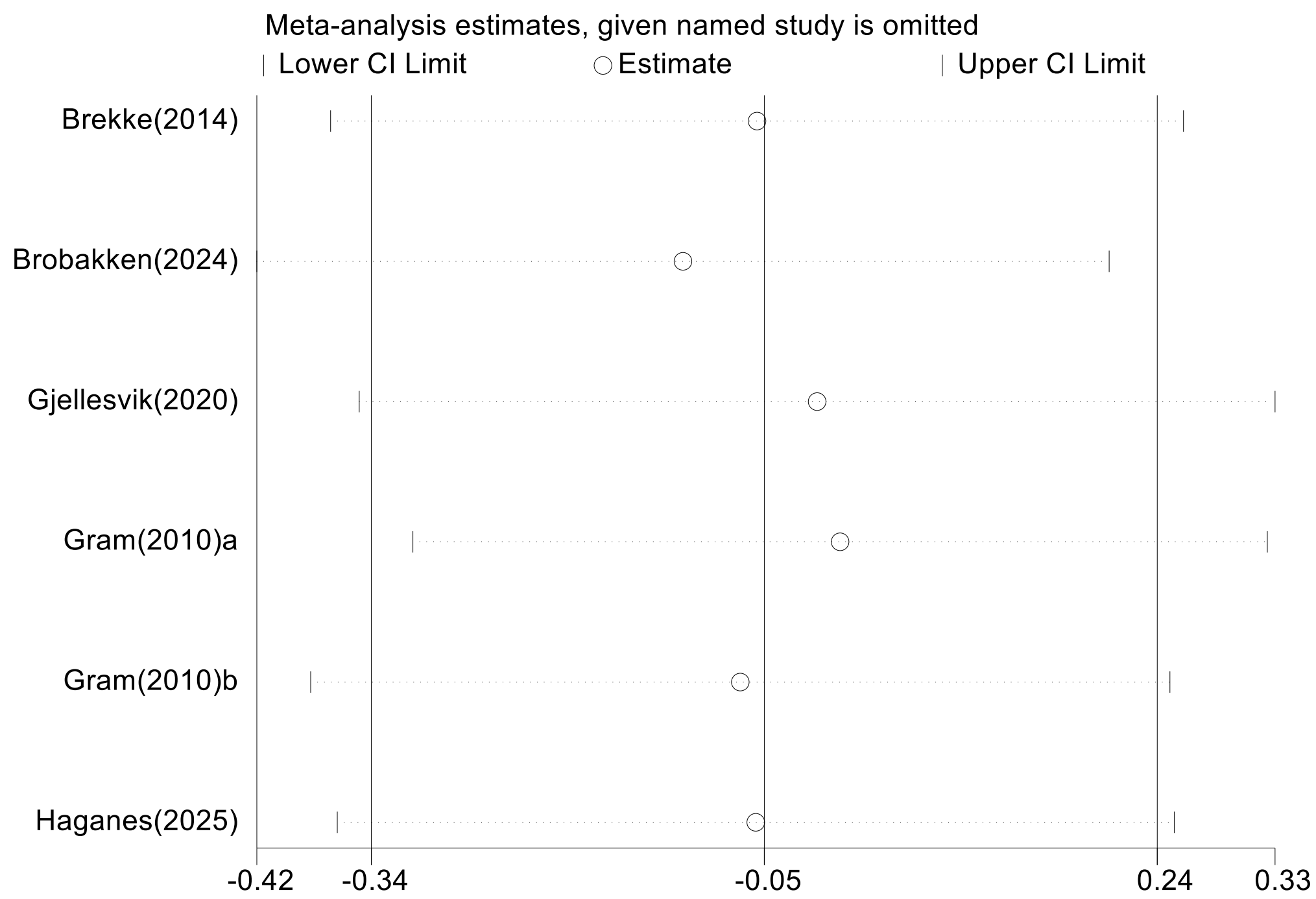

Supplementary Figure 23 Leave-one-out sensitivity analysis for Triglycerides

Short-term subgroup

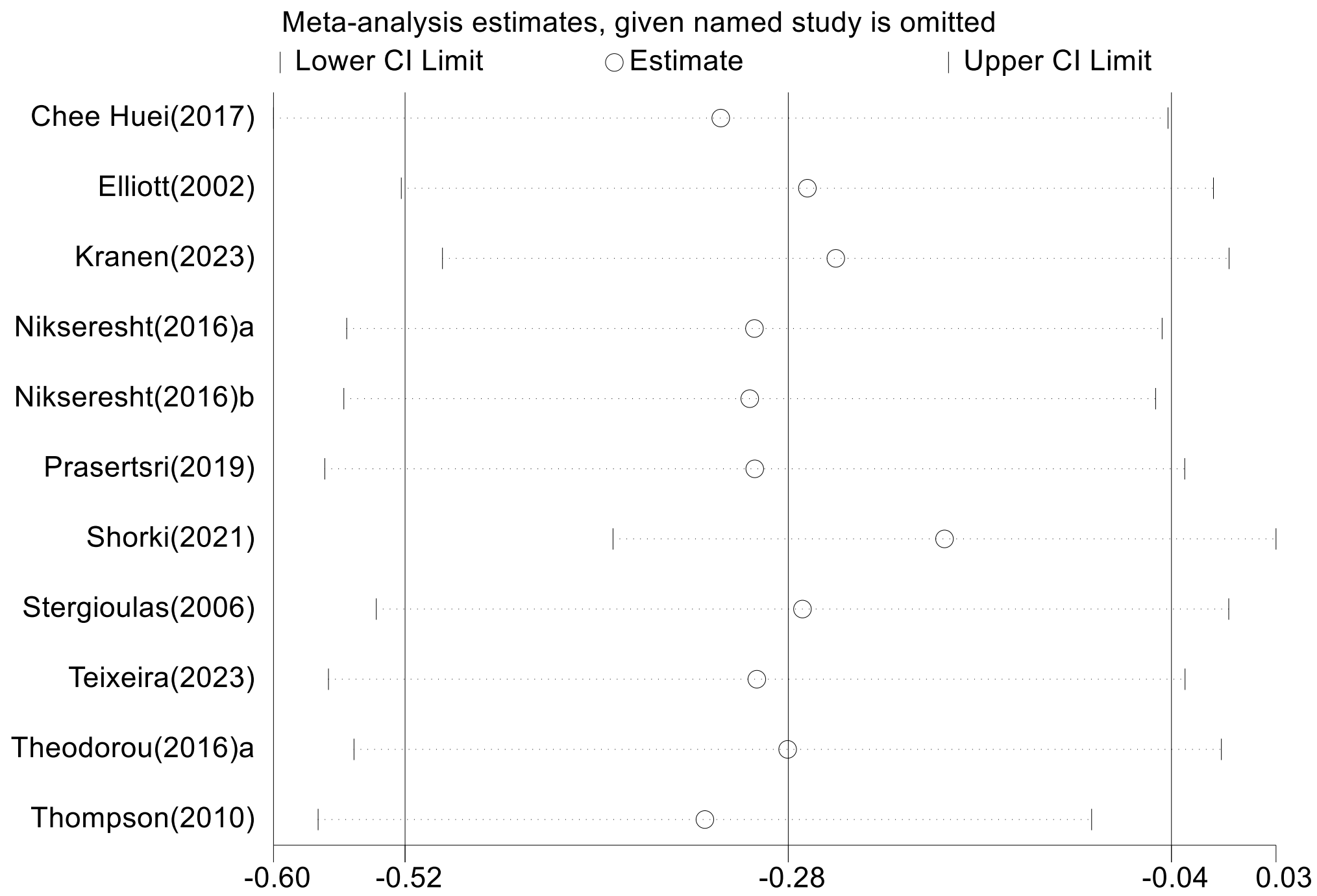

medium-term subgroup

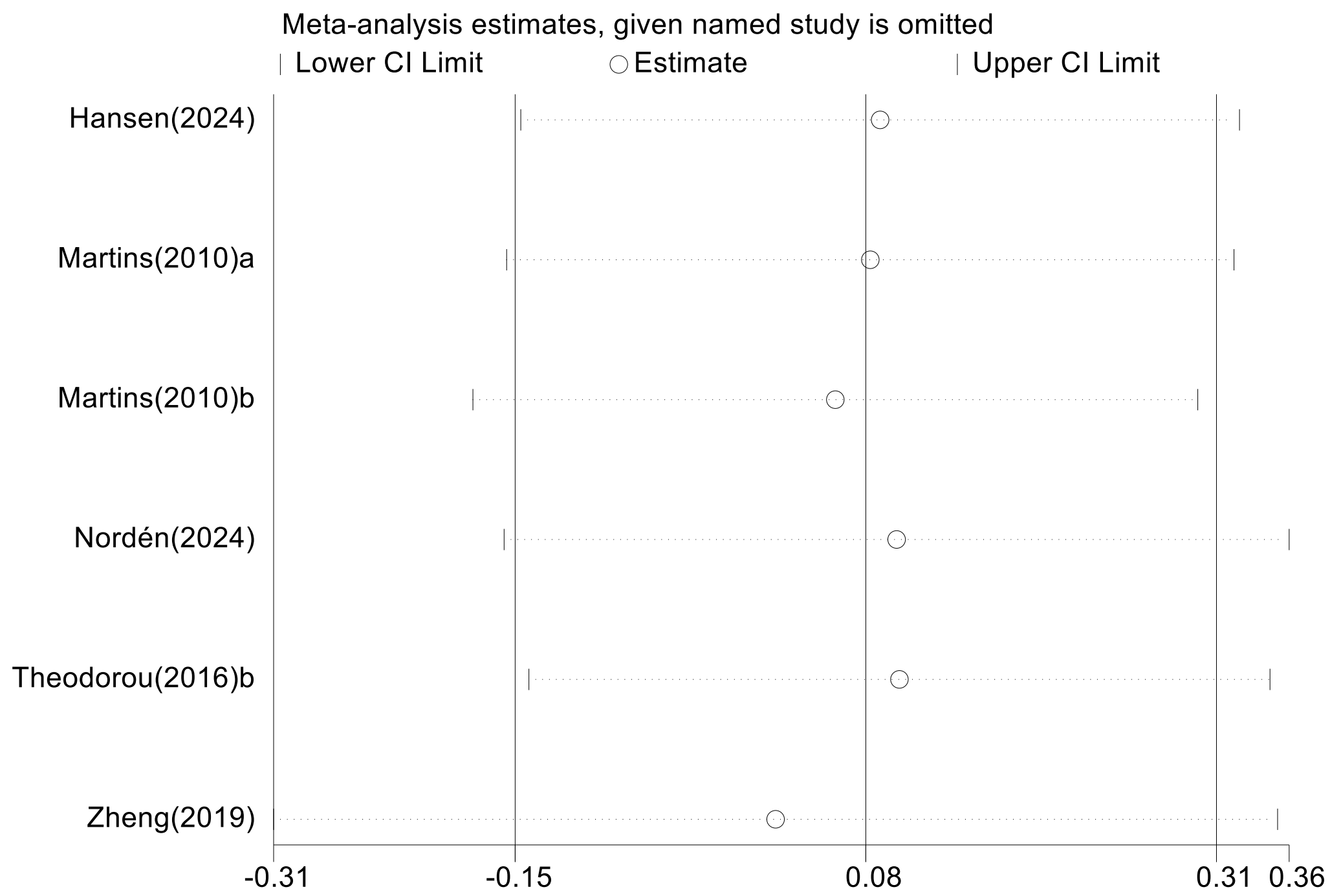

Long-term subgroup

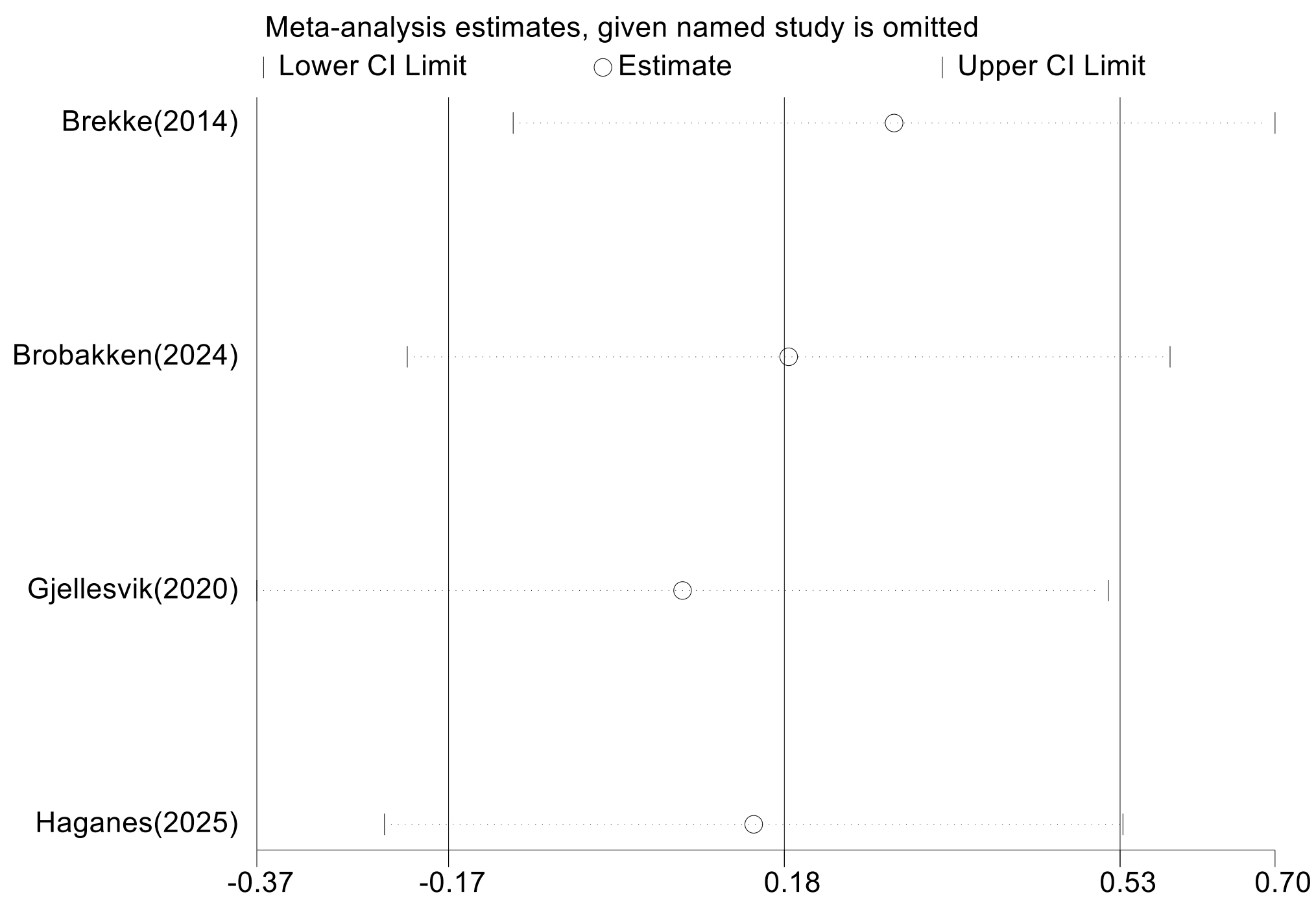

Supplementary Figure 24 Leave-one-out sensitivity analysis for Fasting blood glucose

Overall

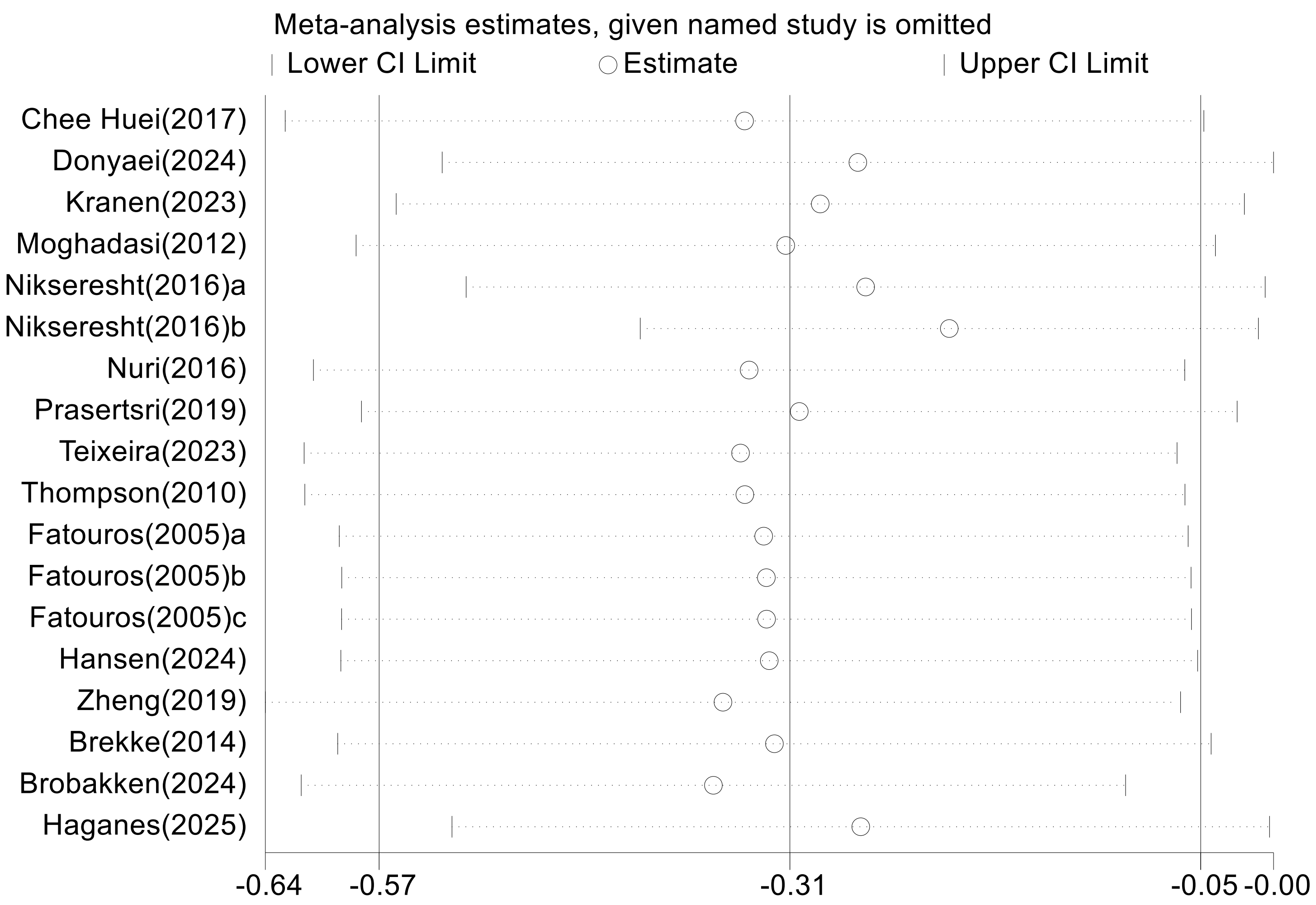

Supplementary Figure 25 Leave-one-out sensitivity analysis for Fasting insulin

Overall

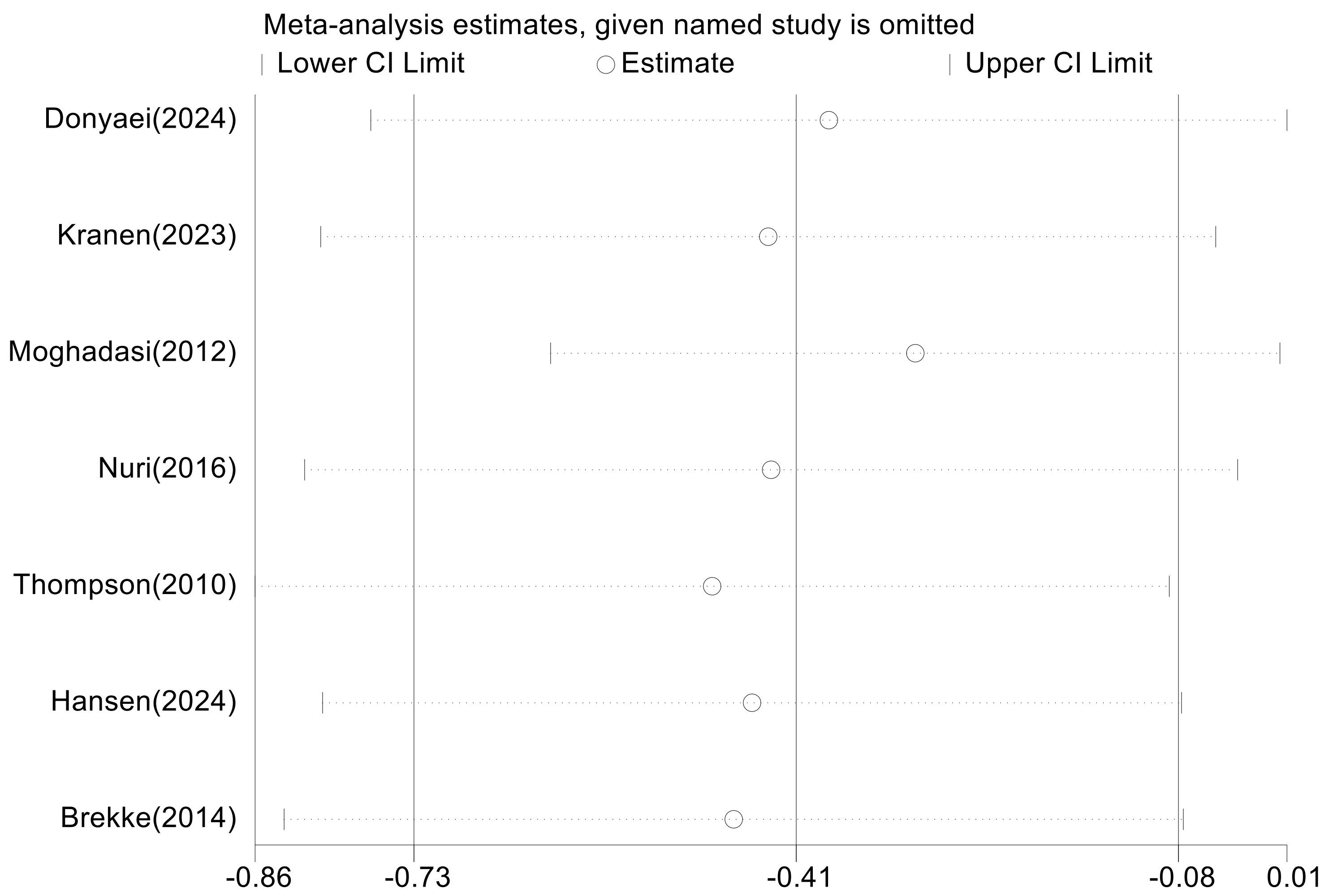

Supplementary Figure 26 Leave-one-out sensitivity analysis for HOMA-IR

Overall

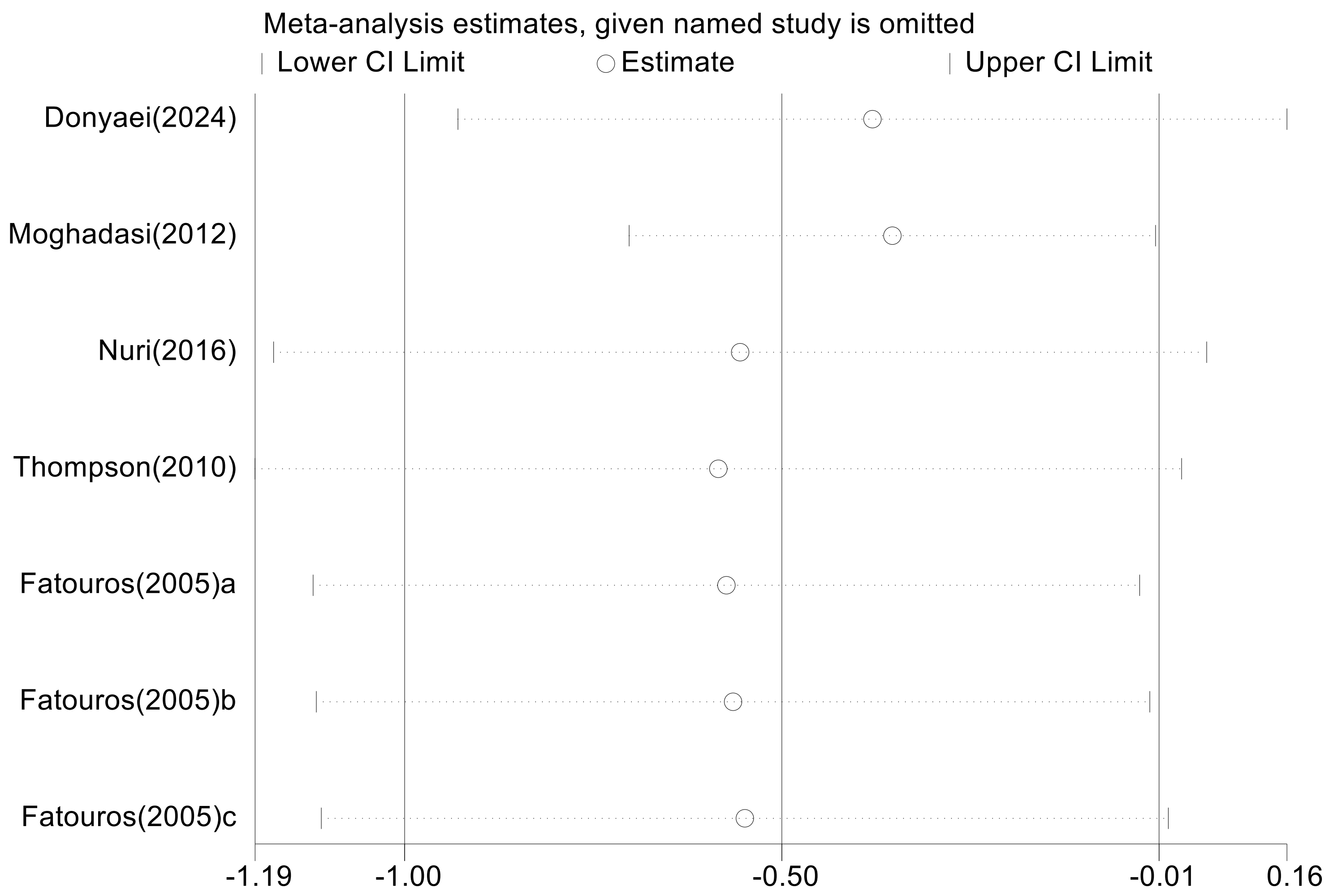

Supplementary Figure 27 Sensitivity analysis excluding very-long-term trials

Outcome	Main analysis: Effect size (95% CI, *p*)	Excluding : Effect size (95% CI, *p*)
Body mass (long-term)	1.12 (−1.83 to −0.40, *p* = 0.002)	−1.09 (−1.82 to −0.36, *p* = 0.004)
BMI (long-term)	−0.20 (−0.64 to 0.23, *p* = 0.37)	−0.20 (−0.63 to 0.24, *p* = 0.38)
WC (long-term)	−2.69 (−4.73 to −0.66, *p* < 0.001)	−2.59 (−4.67 to −0.51, *p* < 0.001)
SBP (long-term)	−1.93 (−4.12 to 0.26, *p* = 0.08)	−1.85 (−4.22 to 0.51, *p* = 0.12)
DBP (long-term)	−1.12 (−1.83 to −0.40, *p* = 0.08)	−1.09 (−1.82 to −0.36, *p* = 0.12)
VO_{2peak} (long-term)	0.13 (−0.03 to 0.29, *p* = 0.12)	0.10 (−0.12 to 0.33, *p* = 0.36)
TC (long-term)	0.01 (−0.27 to 0.30, *p* = 0.92)	0.02 (−0.29 to 0.33, *p* = 0.90)
HDL-C (long-term)	−0.05 (−0.37 to 0.26, *p* = 0.73)	0.03 (−0.28 to 0.35, *p* = 0.84)
LDL-C (long-term)	−0.05 (−0.34 to 0.24, *p* = 0.75)	−0.11 (−0.42 to 0.21, *p* = 0.50)
TG (long-term)	0.18 (−0.17 to 0.53, *p* = 0.31)	0.19 (−0.21 to 0.59, *p* = 0.36)
FBG (overall)	−0.31 (−0.57 to −0.05, p = 0.02)	−0.36 (−0.62 to −0.10, *p* = 0.007)

Supplementary Figure 28 Forest plot of subanalyses of Body mass

Study or Subgroup	Experimental Mean	Experimental SD	Experimental Total	Control Mean	Control SD	Control Total	Weight	Mean Difference IV, Random, 95% CI
**3.1.1 ≤ 2months**								
Chen(2018)	-0.38	11.97	17	0.1	10	16	1.4%	-0.48 [-7.99, 7.03]
Elliott(2002)	-0.41	12.87	8	-0.13	7.49	7	0.7%	-0.28 [-10.78, 10.22]
Fernandez-del-Valle(2014)	1.63	6.97	18	2.33	6.25	18	4.4%	-0.70 [-5.02, 3.62]
Liu(2023)	-0.55	6.62	11	-0.46	6.27	9	2.5%	-0.09 [-5.75, 5.57]
Lovell(2011)	-1	14.38	12	0	14.38	12	0.6%	-1.00 [-12.51, 10.51]
Moghadasi(2012)	-2.68	8.65	8	0.2	14	8	0.6%	-2.88 [-14.28, 8.52]
Nikseresht(2016)a	-2.5	5.65	10	1	5.36	6	2.7%	-3.50 [-9.04, 2.04]
Nikseresht(2016)b	-0.6	6.78	12	1	5.36	6	2.5%	-1.60 [-7.35, 4.15]
Nuri(2016)	-0.9	11.34	15	-1.12	12.34	15	1.1%	0.22 [-8.26, 8.70]
Okamoto(2007)a	0.9	10.45	11	0.4	6.99	6	1.2%	0.50 [-7.83, 8.83]
Okamoto(2007)b	1.3	8.5	11	0.4	6.99	6	1.4%	0.90 [-6.62, 8.42]
Oliveira(2021)	0.8	6.15	10	0.7	8.8	9	1.7%	0.10 [-6.80, 7.00]
Shokri(2023)	-2.93	1.83	15	0.5	2.39	15	35.2%	-3.43 [-4.95, -1.91]
Shorki(2021)	-1.57	2.18	15	0.16	1.94	15	37.4%	-1.73 [-3.21, -0.25]
Sosa(2012)	1.5	11.2	10	2.2	7.58	8	1.1%	-0.70 [-9.40, 8.00]
Sosa(2014)	1.9	9.8	10	1.2	12.04	7	0.7%	0.70 [-10.09, 11.49]
Stergioulas(2006)	-0.76	13.11	21	-0.4	15.93	17	0.9%	-0.36 [-9.78, 9.06]
Teixeira(2023)	-1.15	15.16	18	-3.6	19.94	17	0.6%	2.45 [-9.34, 14.24]
Theodorou(2016)a	-2.21	9.71	41	-0.2	14.14	15	1.4%	-2.01 [-9.76, 5.74]
Thompson(2010)	-1.8	12.03	21	0.7	10.05	20	1.8%	-2.50 [-9.27, 4.27]
**Subtotal (95% CI)**			**294**			**232**	**100.0%**	**-2.09 [-3.00, -1.19]**
Heterogeneity: Tau² = 0.00; Chi² = 7.42, df = 19 (P = 0.99); I² = 0%								
Test for overall effect: Z = 4.54 (P < 0.00001)								
**3.1.2 2-6months**								
Batrakoulis(2018)	-1.5	8.46	14	0.7	8.36	21	0.8%	-2.20 [-7.89, 3.49]
Brooker(2023)a	-1.63	3.28	42	-1.35	4.5	13	3.9%	-0.28 [-2.92, 2.36]
Brooker(2023)b	-2.47	3.41	46	-1.35	4.5	13	3.9%	-1.12 [-3.76, 1.52]
Cantarero-Villanueva(2016)	2.9	13.72	15	1.7	11.06	16	0.3%	1.20 [-7.61, 10.01]
Ciccolo(2011)	-0.7	1.89	7	1.1	2.2	6	5.3%	-1.80 [-4.05, 0.45]
Fatouros(2004)	-0.6	9.97	6	-0.9	10.51	5	0.2%	0.30 [-11.89, 12.49]
Fatouros(2005)a	0.3	10.4	14	0.7	9.1	3	0.2%	-0.40 [-12.05, 11.25]
Fatouros(2005)b	0.4	8.56	12	0.7	9.1	3	0.2%	-0.30 [-11.68, 11.08]
Fatouros(2005)c	-1.6	9.4	14	0.7	9.1	3	0.2%	-2.30 [-13.71, 9.11]
Hansen(2024)	-5.37	15.71	8	-0.66	37.04	6	0.0%	-4.71 [-36.28, 26.86]
Herring(2017)	-2.7	5.4	12	2.9	2.9	12	2.2%	-5.60 [-9.07, -2.13]
Knols(2011)	-0.2	14.41	51	-1.4	14.02	54	0.9%	1.20 [-4.24, 6.64]
Lo(2011)a	0.2	8.26	10	1.2	11.63	5	0.2%	-1.00 [-12.41, 10.41]
Lo(2011)b	-0.4	8.82	10	1.2	11.63	5	0.2%	-1.60 [-13.17, 9.97]
Martins(2010)a	-0.9	11.66	18	-1	14.02	13	0.3%	0.10 [-9.23, 9.43]
Martins(2010)b	-0.2	9.1	14	-1	14.02	13	0.3%	0.80 [-8.19, 9.79]
Rosado(2022)a	0.3	9.41	16	0.3	11.9	8	0.3%	0.00 [-9.45, 9.45]
Rosado(2022)b	0.1	10.82	16	0.3	11.9	8	0.3%	-0.20 [-10.00, 9.60]
Rydwik(2007)	-0.7	2.39	19	0.5	1.82	14	13.1%	-1.20 [-2.64, 0.24]
Schranz(2014)	3.3	16.26	21	7.7	24	22	0.2%	-4.40 [-16.60, 7.80]
Stener-Victorin(2012)	0.07	3.43	30	0.7	1.87	15	11.2%	-0.63 [-2.18, 0.92]
Theodorou(2016)b	-1.81	9.87	41	0.1	14.14	15	0.4%	-1.91 [-9.68, 5.86]
Wang(2022)	-1	1.34	15	0.13	0.79	15	43.6%	-1.13 [-1.92, -0.34]
Winn(2021)a	4	16.39	28	3.9	13.62	71	0.6%	0.10 [-6.75, 6.95]
Winn(2021)b	3.9	13.42	95	3.8	11.82	160	2.5%	0.10 [-3.16, 3.36]
Zech(2012)a	1.35	10.56	36	-0.51	13.16	21	0.6%	1.86 [-4.74, 8.46]
Zech(2012)b	4.69	11.39	33	-0.51	13.16	21	0.6%	5.20 [-1.64, 12.04]
Zhao(2024)	-2.56	2.67	22	-0.32	2.8	12	7.2%	-2.24 [-4.18, -0.30]
**Subtotal (95% CI)**			**665**			**573**	**100.0%**	**-1.14 [-1.66, -0.62]**
Heterogeneity: Tau² = 0.00; Chi² = 15.48, df = 27 (P = 0.96); I² = 0%								
Test for overall effect: Z = 4.31 (P < 0.0001)								
**3.1.4 > 6months**								
Anandavadivelan(2024)a	1.43	10.51	93	1.82	11.51	36	2.7%	-0.39 [-4.71, 3.93]
Anandavadivelan(2024)b	-0.69	12.25	90	1.82	11.51	36	2.5%	-2.51 [-7.04, 2.02]
Bertz (2012)	-2.7	5.9	15	-0.9	6.6	13	2.3%	-1.80 [-6.47, 2.87]
Brobakken(2024)	1.3	25.42	14	8.8	23.83	14	0.2%	-7.50 [-25.75, 10.75]
Gram(2010)a	-1.8	12.97	21	-0.2	13.68	11	0.5%	-1.60 [-11.40, 8.20]
Gram(2010)b	-1.1	15.26	24	-0.2	13.68	11	0.5%	-0.90 [-11.03, 9.23]
Haganes(2025)	-6.2	13.2	12	-1.1	11.15	10	0.5%	-5.10 [-15.28, 5.08]
Kim(2011)a	-1.1	8.9	59	0	8.15	61	5.5%	-1.10 [-4.16, 1.96]
Mundbjerg(2018)	-8.2	18.63	22	-4.4	19.5	20	0.4%	-3.80 [-15.36, 7.76]
Nogueira(2017)	8.4	10.38	155	7.3	10.22	85	6.9%	1.10 [-1.62, 3.82]
O'Connor(2017)a	-3.8	15.75	12	0.9	14.06	10	0.3%	-4.70 [-17.16, 7.76]
O'Connor(2017)b	2.9	13.33	10	0.9	14.06	10	0.4%	2.00 [-10.01, 14.01]
Vainshelboim(2016)	-2	4	14	1	5	14	4.5%	-3.00 [-6.35, 0.35]
Waller(2017)	-0.6	10.45	40	0	11.35	36	2.1%	-0.60 [-5.52, 4.32]
Zhang(2017)a	-2.89	2.96	69	-1.44	3.02	37	35.5%	-1.45 [-2.65, -0.25]
Zhang(2017)b	-2.24	2.93	66	-1.44	3.02	37	35.2%	-0.80 [-2.00, 0.40]
**Subtotal (95% CI)**			**716**			**441**	**100.0%**	**-1.12 [-1.83, -0.40]**
Heterogeneity: Tau² = 0.00; Chi² = 6.78, df = 15 (P = 0.96); I² = 0%								
Test for overall effect: Z = 3.07 (P = 0.002)								

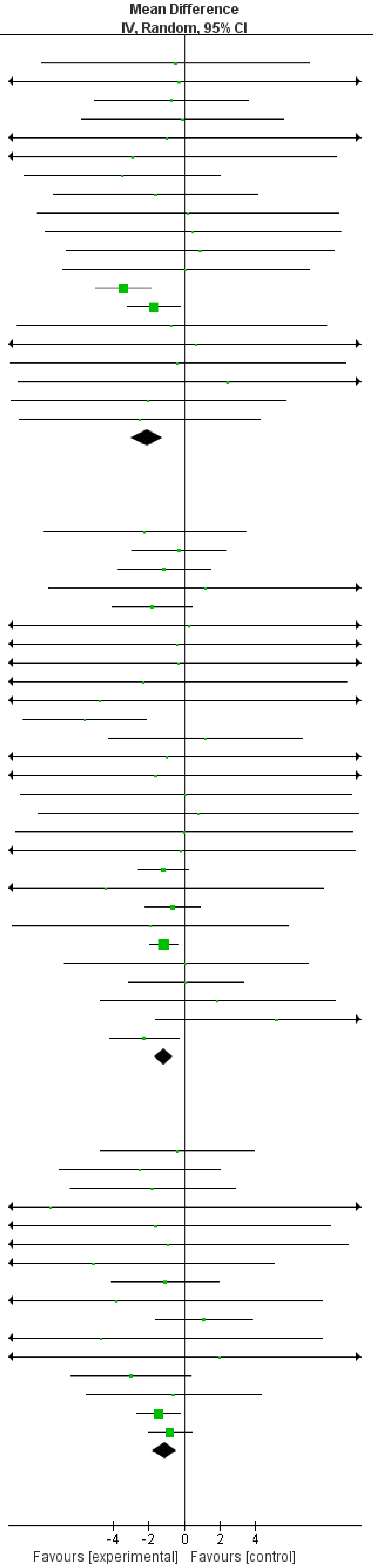

Test for subgroup differences: Chi² = 3.57, df = 2 (P = 0.17), I² = 43.9%

Supplementary Figure 29 Forest plot of subanalyses of BMI

Study or Subgroup	exercise Mean	exercise SD	exercise Total	Control Mean	Control SD	Control Total	Weight	Mean Difference IV, Random, 95% CI
**3.2.1 ≤ 2months**								
Broderick(2013)	0.7	1.28	20	0.7	1.21	18	18.1%	0.00 [-0.79, 0.79]
Carei(2009)	0.5	3.31	24	0.5	2.29	26	6.0%	0.00 [-1.59, 1.59]
Elliott(2002)	-0.15	5.91	8	-0.05	2.49	7	0.8%	-0.10 [-4.59, 4.39]
Fernandez-del-Valle(2014)	0.33	2.5	18	0.8	2.5	18	5.7%	-0.47 [-2.10, 1.16]
Kranen(2023)	-0.1	0.85	10	-0.2	2.45	9	5.4%	0.10 [-1.59, 1.79]
Liu(2023)	-0.24	2.34	11	-0.16	2.19	9	4.0%	-0.08 [-2.07, 1.91]
Moghadasi(2012)	-0.96	2.21	8	0.05	2.45	8	3.1%	-1.01 [-3.30, 1.28]
Nuri(2016)	-0.33	3.84	15	-0.4	3.32	15	2.4%	0.07 [-2.50, 2.64]
Okamoto(2007)a	0.3	2.17	11	-0.3	2.84	6	2.4%	0.60 [-2.01, 3.21]
Okamoto(2007)b	0.3	4.02	11	-0.3	2.84	6	1.5%	0.60 [-2.69, 3.89]
Shokri(2023)	-1.29	0.69	15	0.24	0.79	15	28.7%	-1.53 [-2.06, -1.00]
Shorki(2021)	-0.98	1.57	15	0.01	1.72	15	10.0%	-0.99 [-2.17, 0.19]
Sosa(2012)	0.1	2.93	10	0.2	2.6	8	2.5%	-0.10 [-2.66, 2.46]
Teixeira(2023)	-0.2	4.85	19	-0.4	4.8	17	1.6%	0.20 [-2.96, 3.36]
Theodorou(2016)a	-0.81	3.57	41	-0.15	4.66	15	2.4%	-0.66 [-3.26, 1.94]
Thompson(2010)	-0.7	2.76	21	0	2.7	20	5.5%	-0.70 [-2.37, 0.97]
**Subtotal (95% CI)**			**257**			**212**	**100.0%**	**-0.62 [-1.03, -0.21]**
Heterogeneity: Tau² = 0.08; Chi² = 17.06, df = 15 (P = 0.32); I² = 12%								
Test for overall effect: Z = 2.97 (P = 0.003)								
**3.2.2 2-6months**								
Batrakoulis(2018)	-0.5	3.27	14	0.3	2.86	21	1.0%	-0.80 [-2.90, 1.30]
Cantarero-Villanueva(2016)	0.9	1.53	15	0.5	1.6	16	3.6%	0.40 [-0.70, 1.50]
Carter(2013)	0.3	5.7	12	0.4	5.26	12	0.2%	-0.10 [-4.49, 4.29]
Carvalho(2009)	0.2	3.06	32	0.6	2.65	25	2.0%	-0.40 [-1.88, 1.08]
Fatouros(2005)a	0.1	3.55	14	0.1	2.05	3	0.5%	0.00 [-2.97, 2.97]
Fatouros(2005)b	0.1	2.91	12	0.1	2.05	3	0.5%	0.00 [-2.84, 2.84]
Fatouros(2005)c	-0.5	4.1	14	0.1	2.05	3	0.4%	-0.60 [-3.76, 2.56]
Herring(2017)	-1	2	12	1	1	12	2.7%	-2.00 [-3.27, -0.73]
Kemmler(2020)	-0.2	2.91	21	-0.1	11.17	22	0.2%	-0.10 [-4.93, 4.73]
Krawcyk(2022)a	0.2	4.5	31	-0.4	3.65	32	1.1%	0.60 [-1.43, 2.63]
Lo(2011)a	0	2.17	10	0.3	4.84	5	0.2%	-0.30 [-4.75, 4.15]
Lo(2011)b	-0.38	3.2	10	0.3	4.84	5	0.2%	-0.68 [-5.36, 4.00]
Martins(2010)a	-0.3	4.75	18	-0.4	3.72	7	0.3%	0.10 [-3.42, 3.62]
Martins(2010)b	-0.1	5.5	14	-0.4	3.72	7	0.3%	0.30 [-3.69, 4.29]
Ng(2015)	-0.1	3.13	46	0.2	3.47	46	2.4%	-0.30 [-1.65, 1.05]
Nordén(2024)	-0.71	4.69	28	-0.35	5.88	30	0.6%	-0.36 [-3.09, 2.37]
Schranz(2014)	0.1	4.16	21	1.2	5.77	22	0.5%	-1.10 [-4.10, 1.90]
Theodorou(2016)b	-0.65	3.63	41	0	4.84	15	0.6%	-0.65 [-3.34, 2.04]
Wang(2022)	-0.43	0.42	15	0.04	0.31	15	61.9%	-0.47 [-0.73, -0.21]
Winn(2021)a	0.1	4.65	28	0.4	4.4	71	1.1%	-0.30 [-2.30, 1.70]
Winn(2021)b	0.6	3.4	95	0.4	3.56	161	5.6%	0.20 [-0.68, 1.08]
Zech(2012)a	-0.56	4.38	36	0.35	5.15	21	0.6%	-0.91 [-3.54, 1.72]
Zech(2012)b	1.75	4.61	33	0.35	5.15	21	0.6%	1.40 [-1.31, 4.11]
Zhao(2024)	-0.96	1.04	22	-0.11	1.01	12	8.4%	-0.85 [-1.57, -0.13]
Zheng(2019)	-0.33	3.28	85	-0.38	3.09	85	4.7%	0.05 [-0.91, 1.01]
**Subtotal (95% CI)**			**679**			**672**	**100.0%**	**-0.42 [-0.63, -0.21]**
Heterogeneity: Tau² = 0.00; Chi² = 16.15, df = 24 (P = 0.88); I² = 0%								
Test for overall effect: Z = 3.96 (P < 0.0001)								
**3.2.3 > 6months**								
Bertz (2012)	-0.9	2	15	-0.3	2.4	13	6.9%	-0.60 [-2.25, 1.05]
Brobakken(2024)	0.7	6.98	14	1.4	5.94	14	0.8%	-0.70 [-5.50, 4.10]
Gram(2010)a	-0.5	3.93	21	-0.2	3.63	11	2.5%	-0.30 [-3.03, 2.43]
Gram(2010)b	-0.6	4.26	24	-0.2	3.63	11	2.5%	-0.40 [-3.14, 2.34]
Hao(2019)a	-0.7	2.15	25	0.2	1.91	27	15.4%	-0.90 [-2.01, 0.21]
Hao(2019)b	-0.7	2.75	32	-0.2	2.29	29	11.8%	-0.50 [-1.77, 0.77]
Kim(2011)a	-0.5	3.26	59	0.3	3.18	61	14.2%	-0.80 [-1.95, 0.35]
Krawcyk(2022)b	0.4	4.73	29	-0.7	3.6	31	4.1%	1.10 [-1.04, 3.24]
Mundbjerg(2018)	-3.2	6.01	22	-1.5	5.3	20	1.6%	-1.70 [-5.12, 1.72]
Nogueira(2017)	1.2	3.36	155	0.5	3.25	85	25.0%	0.70 [-0.17, 1.57]
Opdenacker(2011)	0.1	3.87	49	0.3	3.59	46	8.4%	-0.20 [-1.70, 1.30]
Waller(2017)	-0.2	3.9	40	-0.2	3.6	36	6.6%	0.00 [-1.69, 1.69]
**Subtotal (95% CI)**			**485**			**384**	**100.0%**	**-0.20 [-0.64, 0.23]**
Heterogeneity: Tau² = 0.00; Chi² = 9.40, df = 11 (P = 0.58); I² = 0%								
Test for overall effect: Z = 0.90 (P = 0.37)								

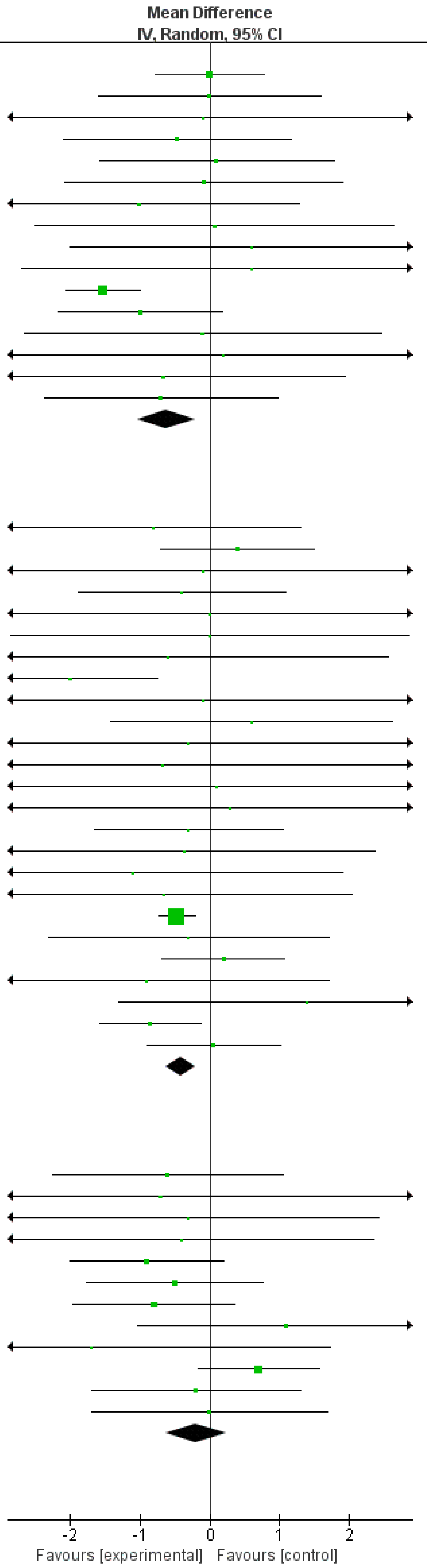

Test for subgroup differences: Chi² = 1.92, df = 2 (P = 0.38), I² = 0%

Supplementary Figure 30 Forest plot of subanalyses of Body fat percentage

Study or Subgroup	Experimental Mean	Experimental SD	Experimental Total	Control Mean	Control SD	Control Total	Weight	Mean Difference IV, Random, 95% CI
**3.3.1 ≤ 2months**								
Donyaei(2024)	-0.97	0.91	17	0.2	4.99	17	9.2%	-1.17 [-3.58, 1.24]
Elliott(2002)	1	2.65	8	0.5	4.58	7	3.6%	0.50 [-3.36, 4.36]
Kranen(2023)	-0.4	6.9	10	-0.3	7.71	9	1.2%	-0.10 [-6.71, 6.51]
Liu(2023)	-3.3	3.67	11	-0.5	3.35	9	5.6%	-2.80 [-5.88, 0.28]
Lovell(2011)	-0.1	3.46	12	0	6.93	12	2.8%	-0.10 [-4.48, 4.28]
Moghadasi(2012)	-2.27	3.1	8	0.01	5.5	8	2.8%	-2.28 [-6.65, 2.09]
Nikseresht(2016)a	-1.7	1.37	10	-0.1	1.31	6	29.5%	-1.60 [-2.95, -0.25]
Nikseresht(2016)b	-1.8	1.8	12	-0.1	1.31	6	25.1%	-1.70 [-3.16, -0.24]
Nuri(2016)	-2.3	8.92	15	-0.1	8.89	15	1.3%	-2.20 [-8.57, 4.17]
Okamoto(2007)a	0.7	7.3	11	-0.1	7.47	6	1.0%	0.80 [-6.57, 8.17]
Okamoto(2007)b	-0.7	6.8	11	-0.1	7.47	6	1.0%	-0.60 [-7.80, 6.60]
Oliveira(2021)	-0.4	6.89	10	-0.3	7.71	9	1.2%	-0.10 [-6.70, 6.50]
Shorki(2021)	-1.78	9.18	15	0.63	9.64	15	1.2%	-2.41 [-9.15, 4.33]
Sosa(2012)	-0.7	9.06	10	0	7.47	8	0.9%	-0.70 [-8.34, 6.94]
Teixeira(2023)	-0.3	4.45	18	-0.1	4.3	17	6.4%	-0.20 [-3.10, 2.70]
Theodorou(2016)a	-0.62	2.36	41	-0.55	5.26	15	7.1%	-0.07 [-2.83, 2.69]
**Subtotal (95% CI)**			**219**			**165**	**100.0%**	**-1.30 [-2.03, -0.56]**
Heterogeneity: Tau² = 0.00; Chi² = 4.84, df = 15 (P = 0.99); I² = 0%								
Test for overall effect: Z = 3.47 (P = 0.0005)								
**3.3.2 2-6months**								
Batrakoulis(2018)	-1.4	4.62	14	0.8	6.5	21	2.3%	-2.20 [-5.89, 1.49]
Brooker(2023)a	0.06	1.99	42	-0.86	2.33	13	12.1%	0.92 [-0.48, 2.32]
Brooker(2023)b	-0.93	2.37	46	-0.86	2.33	13	11.6%	-0.07 [-1.51, 1.37]
Cantarero-Villanueva(2016)	0.8	4.74	15	2.5	4.79	16	2.7%	-1.70 [-5.06, 1.66]
Furzer(2016)	-0.61	9.77	18	0.07	11.12	19	0.7%	-0.68 [-7.42, 6.06]
Herring(2017)	-0.7	1.5	12	0.6	1.7	12	13.7%	-1.30 [-2.58, -0.02]
Lee(2021)	-1.36	5.64	15	-0.17	7.3	12	1.3%	-1.19 [-6.21, 3.83]
Lo(2011)a	1	2.89	10	1.9	3.23	5	2.7%	-0.90 [-4.25, 2.45]
Lo(2011)b	1.7	3.4	10	1.9	3.23	5	2.5%	-0.20 [-3.73, 3.33]
Nemoto(2024)	0.1	9.41	29	0.5	9.95	27	1.2%	-0.40 [-5.48, 4.68]
Rosado(2022)a	-0.3	4.8	16	-0.4	6.8	8	1.1%	0.10 [-5.17, 5.37]
Rosado(2022)b	-0.1	6.2	16	-0.4	6.8	8	1.0%	0.30 [-5.31, 5.91]
Schranz(2014)	-2.5	5.75	22	-5	8.05	21	1.8%	2.50 [-1.70, 6.70]
Theodorou(2016)b	-0.35	2.31	41	-0.4	5.33	15	3.8%	0.05 [-2.74, 2.84]
Wang(2022)	-0.16	0.79	15	0.14	0.87	15	30.2%	-0.30 [-0.89, 0.29]
Zhao(2024)	-2.23	1.98	22	0.39	2.16	12	11.2%	-2.62 [-4.10, -1.14]
**Subtotal (95% CI)**			**343**			**222**	**100.0%**	**-0.56 [-1.13, 0.01]**
Heterogeneity: Tau² = 0.19; Chi² = 17.84, df = 15 (P = 0.27); I² = 16%								
Test for overall effect: Z = 1.92 (P = 0.05)								
**3.3.3 > 6months**								
Opdenacker(2011)	-0.2	8.09	49	0.1	8.12	46	2.8%	-0.30 [-3.56, 2.96]
Vainshelboim(2016)	-0.4	3	14	0.6	3	14	6.0%	-1.00 [-3.22, 1.22]
Zhang(2017)a	1.42	1.98	69	1.22	2.04	37	45.8%	0.20 [-0.61, 1.01]
Zhang(2017)b	0.89	1.97	66	1.22	2.04	37	45.3%	-0.33 [-1.14, 0.48]
**Subtotal (95% CI)**			**198**			**134**	**100.0%**	**-0.13 [-0.67, 0.42]**
Heterogeneity: Tau² = 0.00; Chi² = 1.48, df = 3 (P = 0.69); I² = 0%								
Test for overall effect: Z = 0.45 (P = 0.65)								

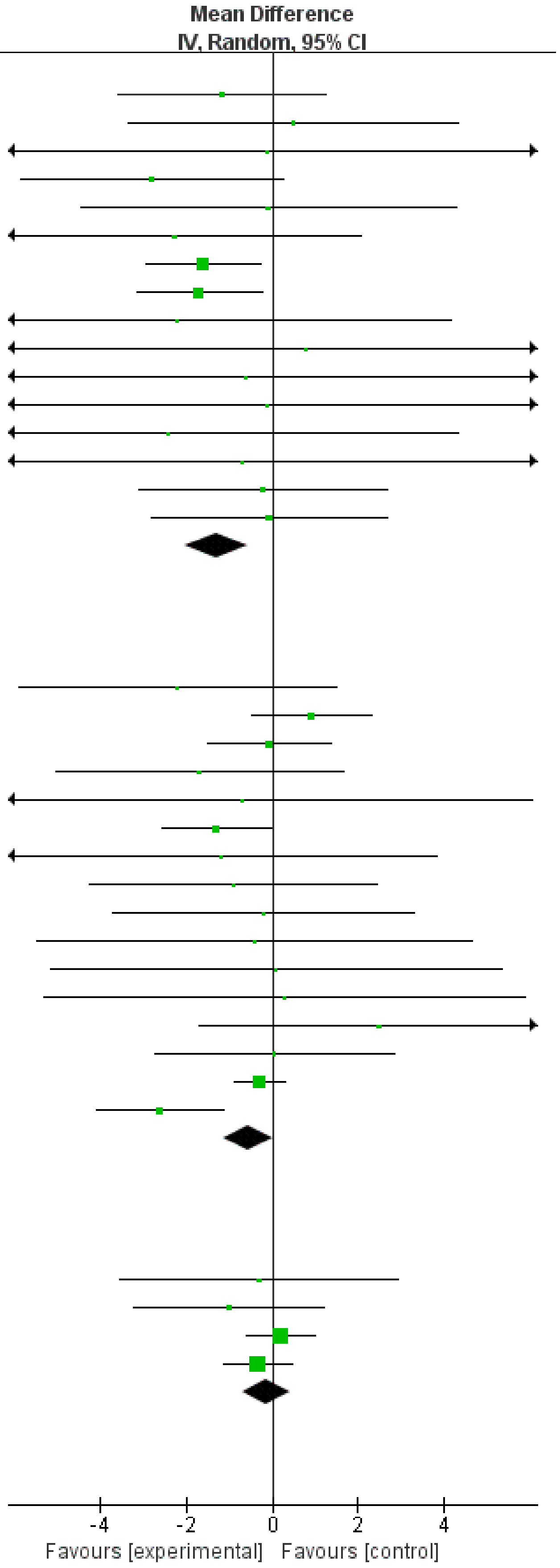

Test for subgroup differences: Chi² = 6.29, df = 2 (P = 0.04), I² = 68.2%

Supplementary Figure 31 Forest plot of subanalyses of Fat mass

Study or Subgroup	exercise Mean	exercise SD	exercise Total	Control Mean	Control SD	Control Total	Weight	Mean Difference IV, Random, 95% CI
**3.4.1 ≤ 2months**								
Chen(2018)	-0.47	7.54	17	0.33	6.25	16	2.7%	-0.80 [-5.51, 3.91]
Kranen(2023)	0.1	3.15	10	-0.2	4.56	9	4.8%	0.30 [-3.26, 3.86]
Moghadasi(2012)	-2.75	3.48	8	0.05	9.55	8	1.2%	-2.80 [-9.84, 4.24]
Rosety-Rodriguez(2014)a	-1.8	1.01	11	0.1	1.15	9	66.1%	-1.90 [-2.86, -0.94]
Shokri(2023)	-0.6	2.21	15	0.1	2.45	15	21.8%	-0.70 [-2.37, 0.97]
Sosa(2014)	-0.9	3.83	10	0.4	4.89	7	3.2%	-1.30 [-5.63, 3.03]
**Subtotal (95% CI)**			**71**			**64**	**100.0%**	**-1.49 [-2.27, -0.71]**
Heterogeneity: Tau² = 0.00; Chi² = 2.75, df = 5 (P = 0.74); I² = 0%								
Test for overall effect: Z = 3.75 (P = 0.0002)								
**3.4.2 2-6months**								
Batrakoulis(2018)	-1.7	6.74	14	1.2	6.5	21	1.5%	-2.90 [-7.39, 1.59]
Bjerre(2019)	-0.3	2.39	97	0.3	2.39	90	34.2%	-0.60 [-1.29, 0.09]
Brooker(2023)a	-0.38	2.6	42	-1.21	3.87	13	5.7%	0.83 [-1.42, 3.08]
Brooker(2023)b	-1.54	3.01	46	-1.4	3.6	13	6.3%	-0.14 [-2.28, 2.00]
Herring(2017)	-1.9	4.1	12	2.1	2.8	12	3.8%	-4.00 [-6.81, -1.19]
Kindred(2019)a	-0.07	2.5	20	-0.49	4.54	26	6.7%	0.42 [-1.64, 2.48]
Knols(2011)	-0.8	6.71	51	1.3	7.95	54	3.8%	-2.10 [-4.91, 0.71]
Lo(2011)a	1	2.42	10	1.9	3.13	5	3.1%	-0.90 [-4.03, 2.23]
Lo(2011)b	1.2	2.87	10	1.9	3.13	5	2.8%	-0.70 [-3.97, 2.57]
Rosety-Rodriguez(2014)b	-0.8	1.15	22	0.05	1.19	18	32.0%	-0.85 [-1.58, -0.12]
**Subtotal (95% CI)**			**324**			**257**	**100.0%**	**-0.73 [-1.30, -0.17]**
Heterogeneity: Tau² = 0.12; Chi² = 10.61, df = 9 (P = 0.30); I² = 15%								
Test for overall effect: Z = 2.56 (P = 0.01)								
**3.4.3 > 6months**								
Bertz (2012)	-2.5	5.9	15	-1.8	6.2	13	13.7%	-0.70 [-5.20, 3.80]
Haganes(2025)	-3.7	11.27	12	-1.7	9.52	10	3.7%	-2.00 [-10.69, 6.69]
Kindred(2019)b	0.84	2.68	20	-0.36	4.49	26	63.6%	1.20 [-0.89, 3.29]
Waller(2017)	-0.3	8.7	40	-0.4	8.26	36	19.1%	0.10 [-3.71, 3.91]
**Subtotal (95% CI)**			**87**			**85**	**100.0%**	**0.61 [-1.05, 2.28]**
Heterogeneity: Tau² = 0.00; Chi² = 1.05, df = 3 (P = 0.79); I² = 0%								
Test for overall effect: Z = 0.72 (P = 0.47)								

Test for subgroup differences: Chi² = 5.73, df = 2 (P = 0.06), I² = 65.1%

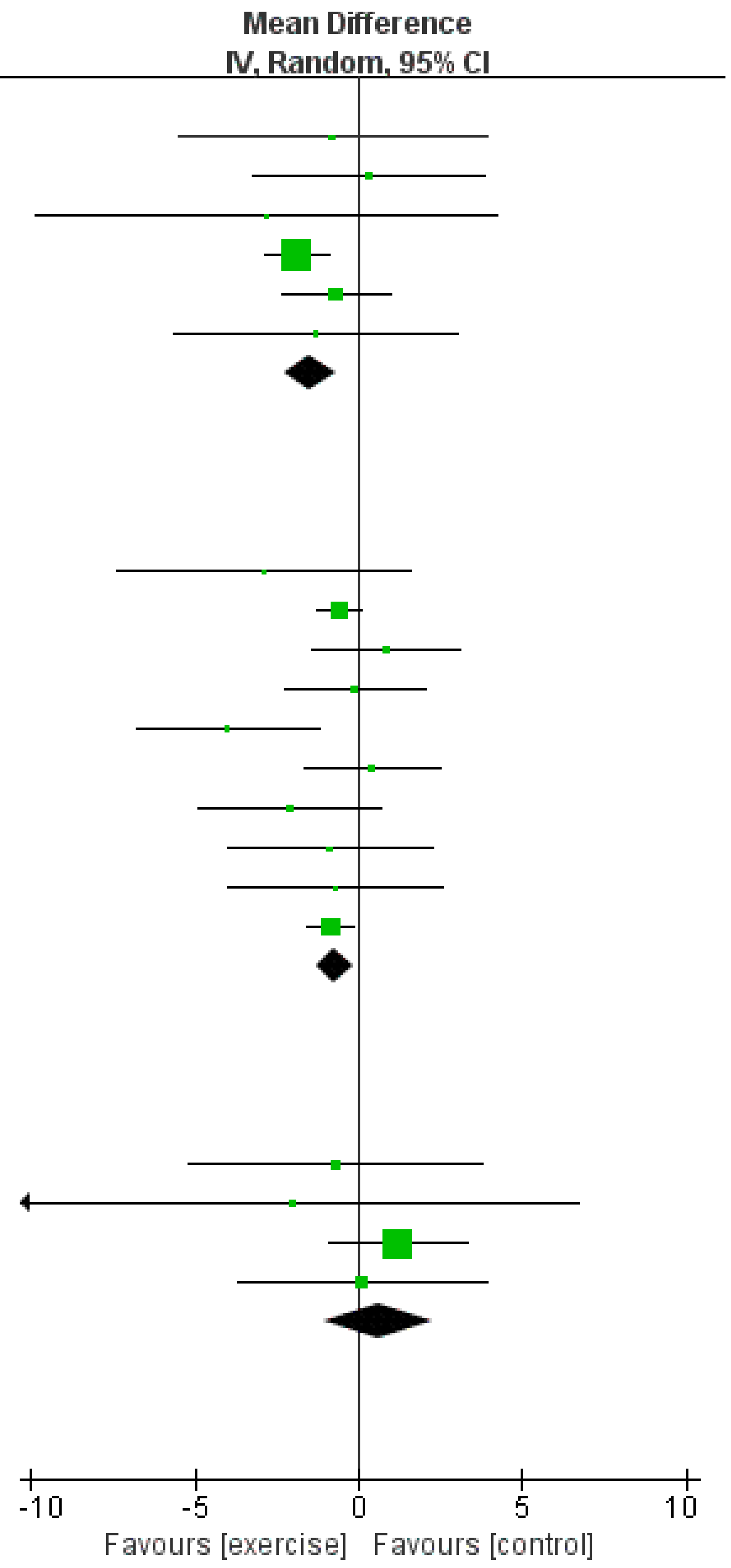

Supplementary Figure 32 Forest plot of subanalyses of Muscle mass

Study or Subgroup	exercise Mean	exercise SD	exercise Total	Control Mean	Control SD	Control Total	Weight	Mean Difference IV, Random, 95% CI
**3.5.1 ≤ 2months**								
Teixeira(2023)	0.08	8.45	18	-0.39	8.6	17	4.3%	0.47 [-5.18, 6.12]
**Subtotal (95% CI)**			**18**			**17**	**4.3%**	**0.47 [-5.18, 6.12]**
Heterogeneity: Not applicable								
Test for overall effect: Z = 0.16 (P = 0.87)								
**3.5.2 2-6months**								
Cantarero-Villanueva(2016)	0.9	1.9	15	0	2.63	16	31.3%	0.90 [-0.71, 2.51]
**Subtotal (95% CI)**			**15**			**16**	**31.3%**	**0.90 [-0.71, 2.51]**
Heterogeneity: Not applicable								
Test for overall effect: Z = 1.10 (P = 0.27)								
**3.5.3 > 6months**								
Bertz (2012)	0.2	1.4	15	1.1	1.5	13	44.8%	-0.90 [-1.98, 0.18]
Haganes(2025)	-1.5	2.45	12	0.3	3.15	11	19.6%	-1.80 [-4.12, 0.52]
**Subtotal (95% CI)**			**27**			**24**	**64.4%**	**-1.06 [-2.04, -0.08]**
Heterogeneity: Tau² = 0.00; Chi² = 0.47, df = 1 (P = 0.49); I² = 0%								
Test for overall effect: Z = 2.12 (P = 0.03)								
**Total (95% CI)**			**60**			**57**	**100.0%**	**-0.45 [-1.67, 0.76]**
Heterogeneity: Tau² = 0.55; Chi² = 4.76, df = 3 (P = 0.19); I² = 37%								
Test for overall effect: Z = 0.73 (P = 0.46)								

Test for subgroup differences: Chi² = 4.28, df = 2 (P = 0.12), I² = 53.3%

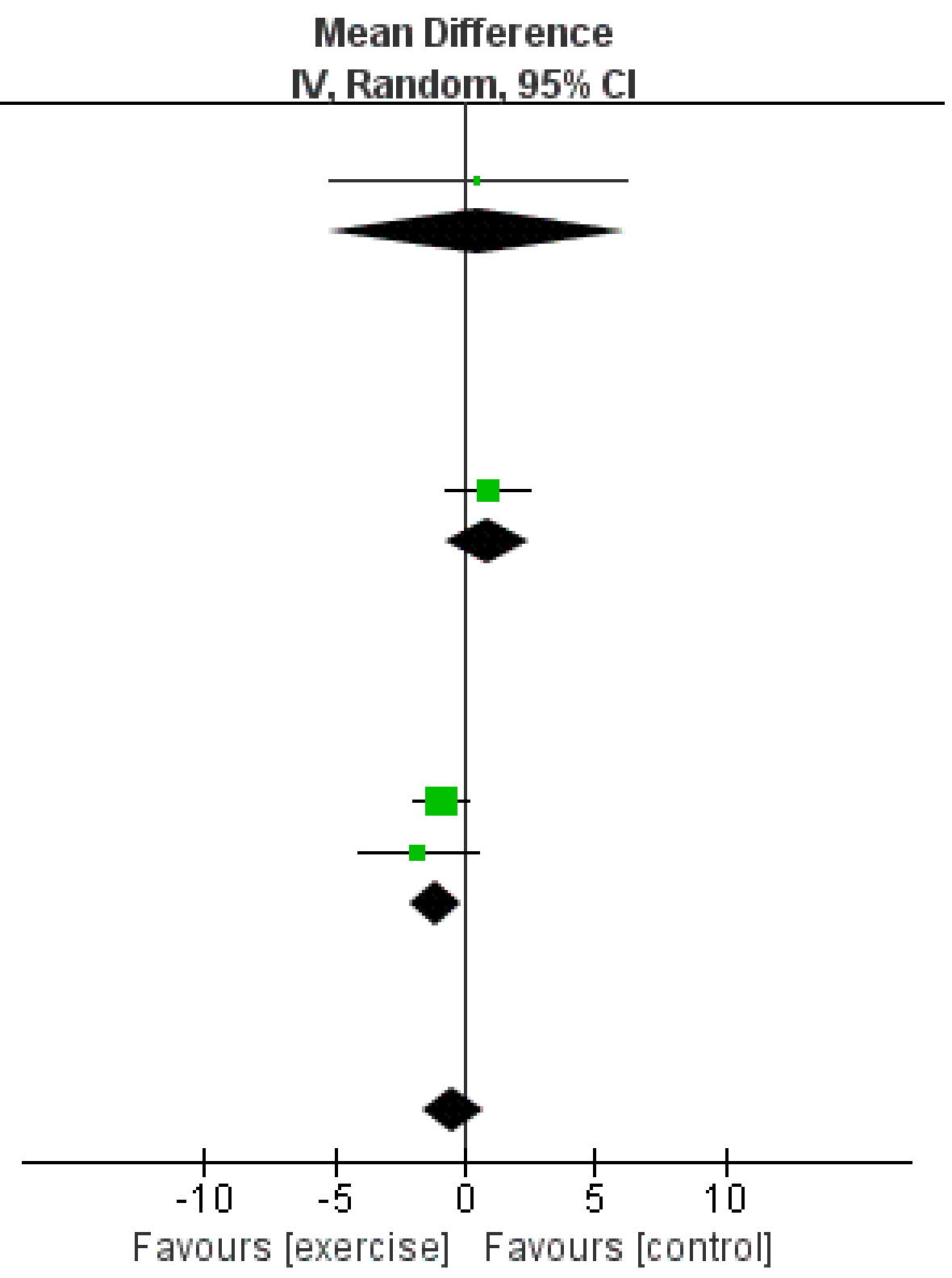

Supplementary Figure 33 Forest plot of subanalyses of Waist circumference

Study or Subgroup	exercise Mean	exercise SD	exercise Total	Control Mean	Control SD	Control Total	Weight	Mean Difference IV, Random, 95% CI
**3.8.1 ≤ 2months**								
Broderick(2013)	-2.2	3.95	20	-0.4	3.52	18	34.0%	-1.80 [-4.18, 0.58]
Chee Huei(2017)	-0.99	8.08	34	0.32	8.38	85	18.2%	-1.31 [-4.56, 1.94]
Nikseresht(2016)a	-5.2	5.99	12	-1	4.55	6	7.7%	-4.20 [-9.17, 0.77]
Nikseresht(2016)b	-4.3	5.01	10	-1	4.55	6	8.4%	-3.30 [-8.09, 1.49]
Rosety-Rodriguez(2014)a	-2.6	3.2	11	-0.1	3.15	9	24.5%	-2.50 [-5.29, 0.29]
Shokri(2023)	-0.81	6.96	15	0.65	7.45	15	7.2%	-1.46 [-6.62, 3.70]
**Subtotal (95% CI)**			**102**			**139**	**100.0%**	**-2.17 [-3.55, -0.79]**
Heterogeneity: Tau² = 0.00; Chi² = 1.34, df = 5 (P = 0.93); I² = 0%								
Test for overall effect: Z = 3.07 (P = 0.002)								
**3.8.2 2-6months**								
Batrakoulis(2018)	-2.4	9.44	14	1.5	4.96	21	2.9%	-3.90 [-9.28, 1.48]
Cantarero-Villanueva(2016)	0.4	3.34	15	2.9	3.75	16	9.7%	-2.50 [-5.00, -0.00]
Hansen(2024)	-7.97	26.3	8	0.08	22.41	6	0.1%	-8.05 [-33.62, 17.52]
Herring(2017)	-3.9	9.1	12	0.5	3	12	2.9%	-4.40 [-9.82, 1.02]
Lo(2011)a	1.1	2.45	10	0.56	3.81	5	5.6%	0.54 [-3.13, 4.21]
Lo(2011)b	0.5	3.33	10	0.56	3.81	5	5.0%	-0.06 [-3.99, 3.87]
Martins(2010)a	-3.9	9.24	18	-5	10.83	7	1.1%	1.10 [-7.99, 10.19]
Martins(2010)b	-0.8	10.95	14	-5	10.83	7	0.9%	4.20 [-5.66, 14.06]
Rosety-Rodriguez(2014)b	-1.7	3.35	22	0.05	3.1	18	12.5%	-1.75 [-3.75, 0.25]
Stener-Victorin(2012)	-0.27	2.66	30	0.89	2.07	15	17.1%	-1.16 [-2.58, 0.26]
Wang(2022)	-0.08	0.99	15	0.04	0.31	15	25.0%	-0.12 [-0.64, 0.40]
Zhao(2024)	-4.05	3.68	22	-0.42	3.97	12	8.6%	-3.63 [-6.35, -0.91]
Zheng(2019)	-2.29	9.23	85	0.72	8.9	85	8.6%	-3.01 [-5.74, -0.28]
**Subtotal (95% CI)**			**275**			**224**	**100.0%**	**-1.43 [-2.40, -0.46]**
Heterogeneity: Tau² = 0.91; Chi² = 20.21, df = 12 (P = 0.06); I² = 41%								
Test for overall effect: Z = 2.90 (P = 0.004)								
**3.8.3 >6months**								
Brekke(2014)	-6	9.9	15	-3.9	5.6	13	2.1%	-2.10 [-7.96, 3.76]
Brobakken(2024)	1	21.14	14	-1	13.09	14	0.4%	2.00 [-11.02, 15.02]
Gram(2010)a	-1	10.15	21	-1	9.17	11	1.5%	0.00 [-6.94, 6.94]
Gram(2010)b	-2	9.9	24	-1	9.17	11	1.6%	-1.00 [-7.71, 5.71]
Kim(2011)a	-1.1	10.11	59	-0.4	10.02	61	5.6%	-0.70 [-4.30, 2.90]
Nogueira(2017)	-2	9.36	155	0	9.28	85	12.0%	-2.00 [-4.46, 0.46]
Opdenacker(2011)	-0.6	11.18	49	-1.1	11.37	46	3.5%	0.50 [-4.04, 5.04]
Vainshelboim(2016)	-4	5	14	-3	13	14	1.4%	-1.00 [-8.30, 6.30]
Zhang(2017)a	-2.02	3.56	69	-0.25	3.56	37	36.1%	-1.77 [-3.19, -0.35]
Zhang(2017)b	-2.85	3.54	66	-0.25	3.56	37	35.7%	-2.60 [-4.03, -1.17]
**Subtotal (95% CI)**			**486**			**329**	**100.0%**	**-1.89 [-2.75, -1.04]**
Heterogeneity: Tau² = 0.00; Chi² = 3.22, df = 9 (P = 0.95); I² = 0%								
Test for overall effect: Z = 4.35 (P < 0.0001)								

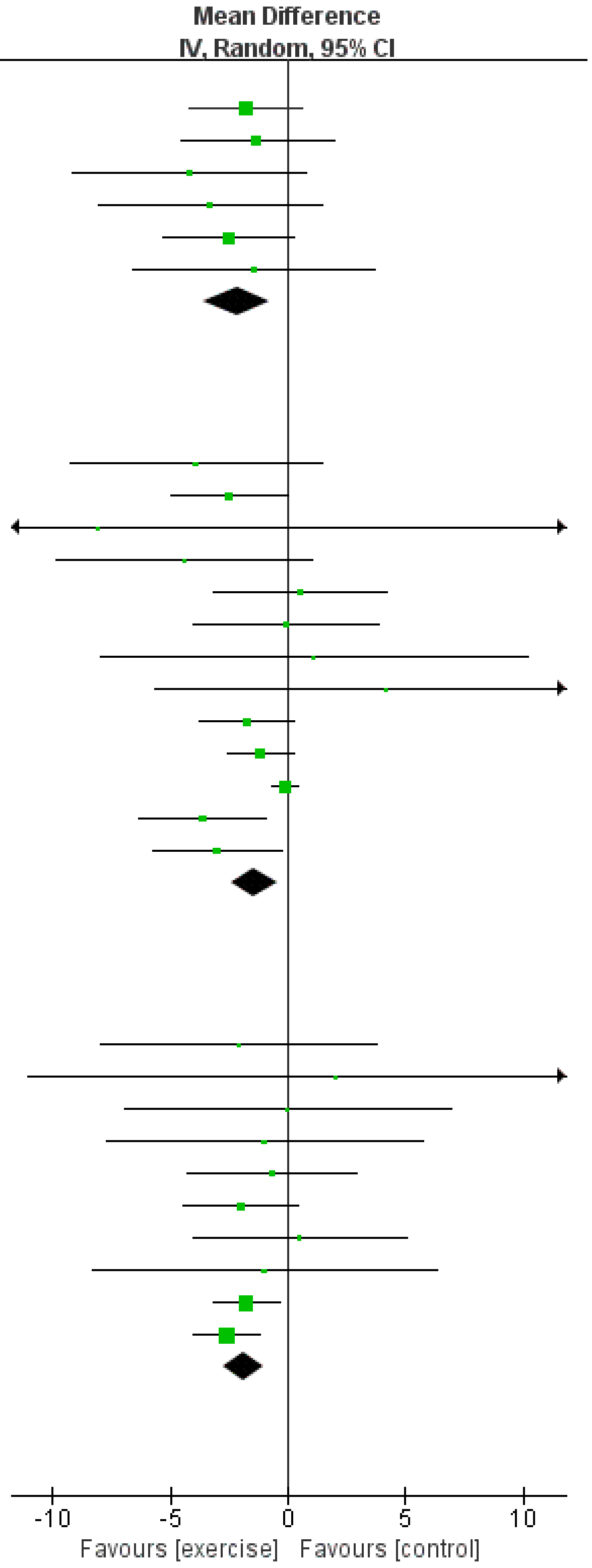

Test for subgroup differences: Chi² = 0.86, df = 2 (P = 0.65), I² = 0%

Supplementary Figure 34 Forest plot of subanalyses of Waist-to-Hip Ratio

Study or Subgroup	exercise Mean	exercise SD	exercise Total	Control Mean	Control SD	Control Total	Weight	Mean Difference IV, Random, 95% CI
**3.9.1 ≤ 2months**								
Elliott(2002)	0	0.09	8	0	0.04	7	0.3%	0.00 [-0.07, 0.07]
Moghadasi(2012)	-0.01	0.03	8	0	0.08	8	0.3%	-0.01 [-0.07, 0.05]
Nikseresht(2016)a	-0.04	0.04	10	0.01	0.05	6	0.6%	-0.05 [-0.10, -0.00]
Nikseresht(2016)b	0	0.04	12	0.01	0.05	6	0.6%	-0.01 [-0.06, 0.04]
**Subtotal (95% CI)**			**38**			**27**	**1.7%**	**-0.02 [-0.05, 0.01]**
Heterogeneity: Tau² = 0.00; Chi² = 2.17, df = 3 (P = 0.54); I² = 0%								
Test for overall effect: Z = 1.57 (P = 0.12)								
**3.9.3 2-6months**								
Batrakoulis(2018)	-0.02	0.07	14	0	0.04	21	0.7%	-0.02 [-0.06, 0.02]
Carter(2013)	0	0.07	12	0.02	0.09	12	0.3%	-0.02 [-0.08, 0.04]
Fatouros(2005)a	0.01	0.08	14	0.01	0.07	3	0.2%	0.00 [-0.09, 0.09]
Fatouros(2005)b	0	0.05	12	0.01	0.07	3	0.2%	-0.01 [-0.09, 0.07]
Fatouros(2005)c	0	0.06	14	0.01	0.07	3	0.2%	-0.01 [-0.10, 0.08]
Lo(2011)a	-0.03	0.09	10	0.01	0.03	5	0.3%	-0.04 [-0.10, 0.02]
Lo(2011)b	0.01	0.03	10	0.01	0.03	5	1.2%	0.00 [-0.03, 0.03]
Wang(2022)	-0.001	0.006	15	0.0004	0.004	15	91.5%	-0.00 [-0.01, 0.00]
Zheng(2019)	-0.01	0.06	85	0.01	0.06	85	3.7%	-0.02 [-0.04, -0.00]
**Subtotal (95% CI)**			**186**			**152**	**98.3%**	**-0.00 [-0.01, 0.00]**
Heterogeneity: Tau² = 0.00; Chi² = 6.47, df = 8 (P = 0.59); I² = 0%								
Test for overall effect: Z = 1.36 (P = 0.17)								
**Total (95% CI)**			**224**			**179**	**100.0%**	**-0.00 [-0.01, 0.00]**
Heterogeneity: Tau² = 0.00; Chi² = 10.53, df = 12 (P = 0.57); I² = 0%								
Test for overall effect: Z = 1.55 (P = 0.12)								
Test for subgroup differences: Chi² = 1.89, df = 1 (P = 0.17), I² = 47.2%								

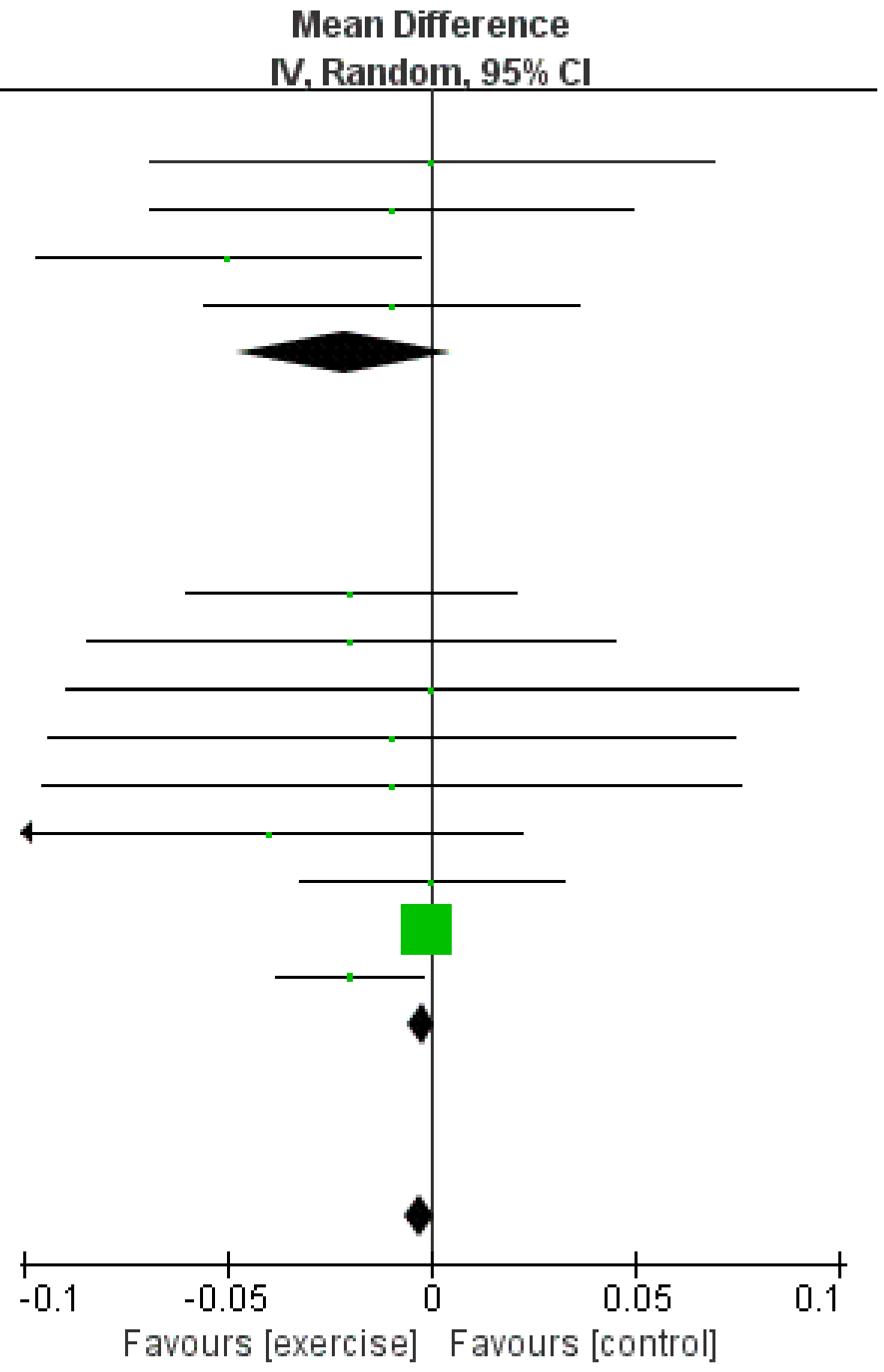

Supplementary Figure 35 Forest plot of subanalyses of Fat-free mass

Study or Subgroup	exercise Mean	exercise SD	exercise Total	Control Mean	Control SD	Control Total	Weight	Mean Difference IV, Random, 95% CI
**3.6.1 ≤ 2months**								
Kranen(2023)	0.7	6.56	10	0.9	7.66	9	17.3%	-0.20 [-6.65, 6.25]
Sloan(2018)	2.18	11.55	40	-0.88	12.36	58	31.4%	3.06 [-1.73, 7.85]
Sosa(2012)	0.7	9.06	10	0	7.47	8	12.3%	0.70 [-6.94, 8.34]
Sosa(2014)	0.9	3.73	10	-0.4	4.89	7	39.0%	1.30 [-3.00, 5.60]
**Subtotal (95% CI)**			**70**			**82**	**100.0%**	**1.52 [-1.16, 4.20]**
Heterogeneity: Tau² = 0.00; Chi² = 0.73, df = 3 (P = 0.87); I² = 0%								
Test for overall effect: Z = 1.11 (P = 0.27)								
**3.6.2 2-6months**								
Batrakoulis(2018)	0.2	3.42	14	-0.5	6.92	21	2.4%	0.70 [-2.76, 4.16]
Brooker(2023)a	-0.99	1.76	42	0.11	1.53	13	29.0%	-1.10 [-2.09, -0.11]
Brooker(2023)b	-0.45	1.97	46	0.11	1.53	13	27.9%	-0.56 [-1.57, 0.45]
Herring(2017)	-0.8	1.7	12	0.8	1.8	12	14.4%	-1.60 [-3.00, -0.20]
Nordén(2024)	0	12.91	28	0.1	11.45	29	0.7%	-0.10 [-6.44, 6.24]
Rydwik(2007)	-0.9	1.97	19	0.2	1.91	14	15.9%	-1.10 [-2.44, 0.24]
Zhao(2024)	-0.13	1.3	22	-0.49	2.86	12	9.7%	0.36 [-1.35, 2.07]
**Subtotal (95% CI)**			**183**			**114**	**100.0%**	**-0.83 [-1.36, -0.30]**
Heterogeneity: Tau² = 0.00; Chi² = 4.55, df = 6 (P = 0.60); I² = 0%								
Test for overall effect: Z = 3.06 (P = 0.002)								

Test for subgroup differences: Chi² = 2.83, df = 1 (P = 0.09), I² = 64.7%

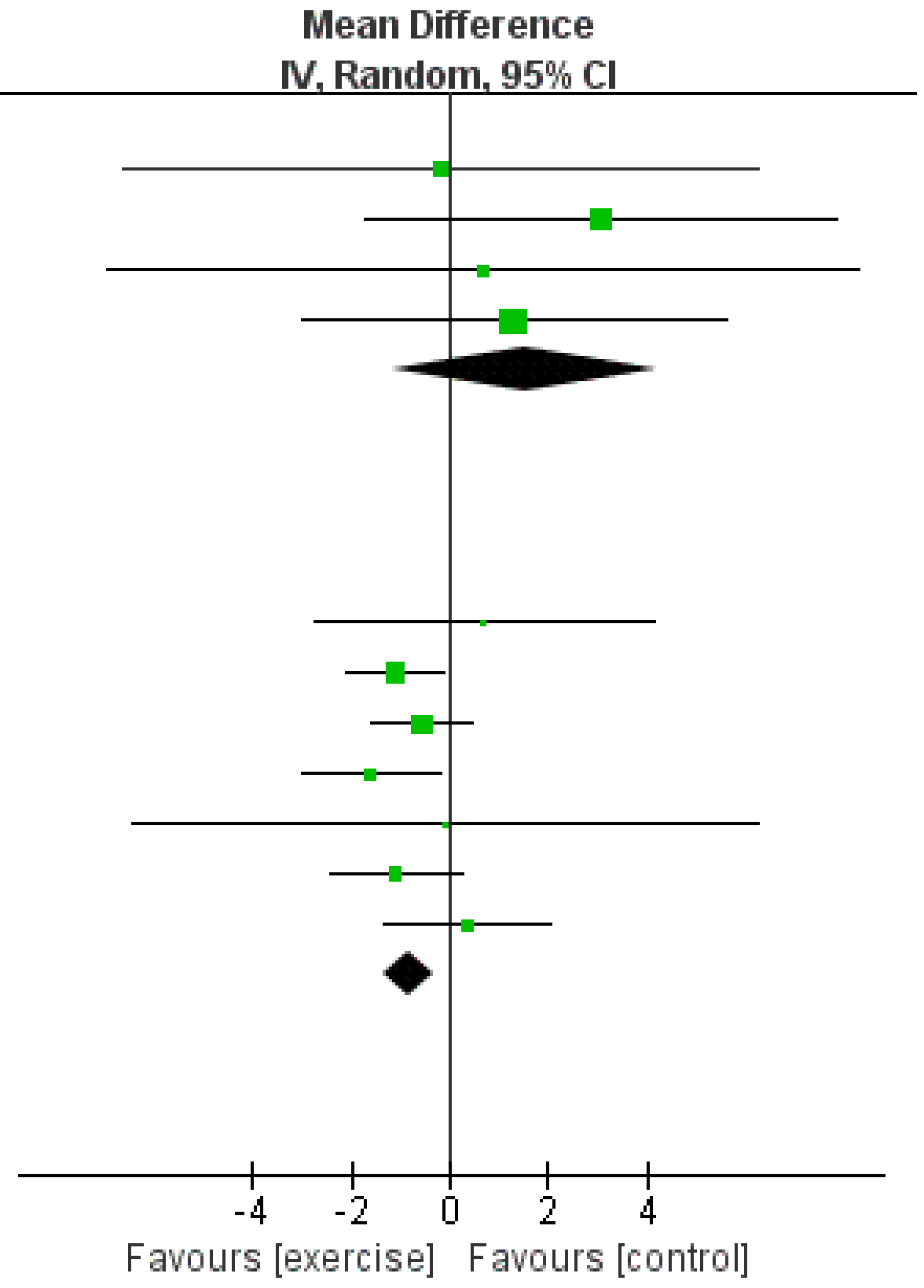

Supplementary Figure 36 Forest plot of subanalyses of Lean body mass

Study or Subgroup	exercise Mean	exercise SD	exercise Total	Control Mean	Control SD	Control Total	Weight	Mean Difference IV, Random, 95% CI
**3.7.1 ≤ 2months**								
Lovell(2011)	2.1	9.09	12	0.2	5.2	12	0.4%	1.90 [-4.03, 7.83]
Moghadasi(2012)	0.59	6.91	8	0.05	4.3	8	0.5%	0.54 [-5.10, 6.18]
**Subtotal (95% CI)**			**20**			**20**	**0.9%**	**1.19 [-2.90, 5.27]**
Heterogeneity: Tau² = 0.00; Chi² = 0.11, df = 1 (P = 0.74); I² = 0%								
Test for overall effect: Z = 0.57 (P = 0.57)								
**3.7.2 2-6months**								
Bjerre(2019)	-0.3	1.49	97	-0.1	1.67	90	75.3%	-0.20 [-0.65, 0.25]
Furzer(2016)	0.95	10.64	18	1.61	9.75	19	0.4%	-0.66 [-7.25, 5.93]
Knols(2011)	-0.3	11.05	51	-1.1	10.87	54	0.9%	0.80 [-3.40, 5.00]
Lo(2011)a	-0.36	1.16	10	-0.14	1.29	5	8.7%	-0.22 [-1.56, 1.12]
Lo(2011)b	-0.08	1.33	10	-0.14	1.29	5	8.0%	0.06 [-1.34, 1.46]
Rosado(2022)a	0.4	7.2	16	0.1	7.45	8	0.4%	0.30 [-5.95, 6.55]
Rosado(2022)b	0.1	6.06	16	0.1	7.45	8	0.4%	0.00 [-5.96, 5.96]
Schranz(2014)	2.4	7.69	21	4.7	10.58	22	0.5%	-2.30 [-7.81, 3.21]
**Subtotal (95% CI)**			**239**			**211**	**94.6%**	**-0.18 [-0.59, 0.23]**
Heterogeneity: Tau² = 0.00; Chi² = 0.95, df = 7 (P = 1.00); I² = 0%								
Test for overall effect: Z = 0.87 (P = 0.38)								
**3.7.3 >6months**								
Waller(2017)	-0.2	3.95	40	-0.2	4.3	36	4.5%	0.00 [-1.86, 1.86]
**Subtotal (95% CI)**			**40**			**36**	**4.5%**	**0.00 [-1.86, 1.86]**
Heterogeneity: Not applicable								
Test for overall effect: Z = 0.00 (P = 1.00)								
**Total (95% CI)**			**299**			**267**	**100.0%**	**-0.16 [-0.55, 0.24]**
Heterogeneity: Tau² = 0.00; Chi² = 1.51, df = 10 (P = 1.00); I² = 0%								
Test for overall effect: Z = 0.79 (P = 0.43)								
Test for subgroup differences: Chi² = 0.46, df = 2 (P = 0.80), I² = 0%								

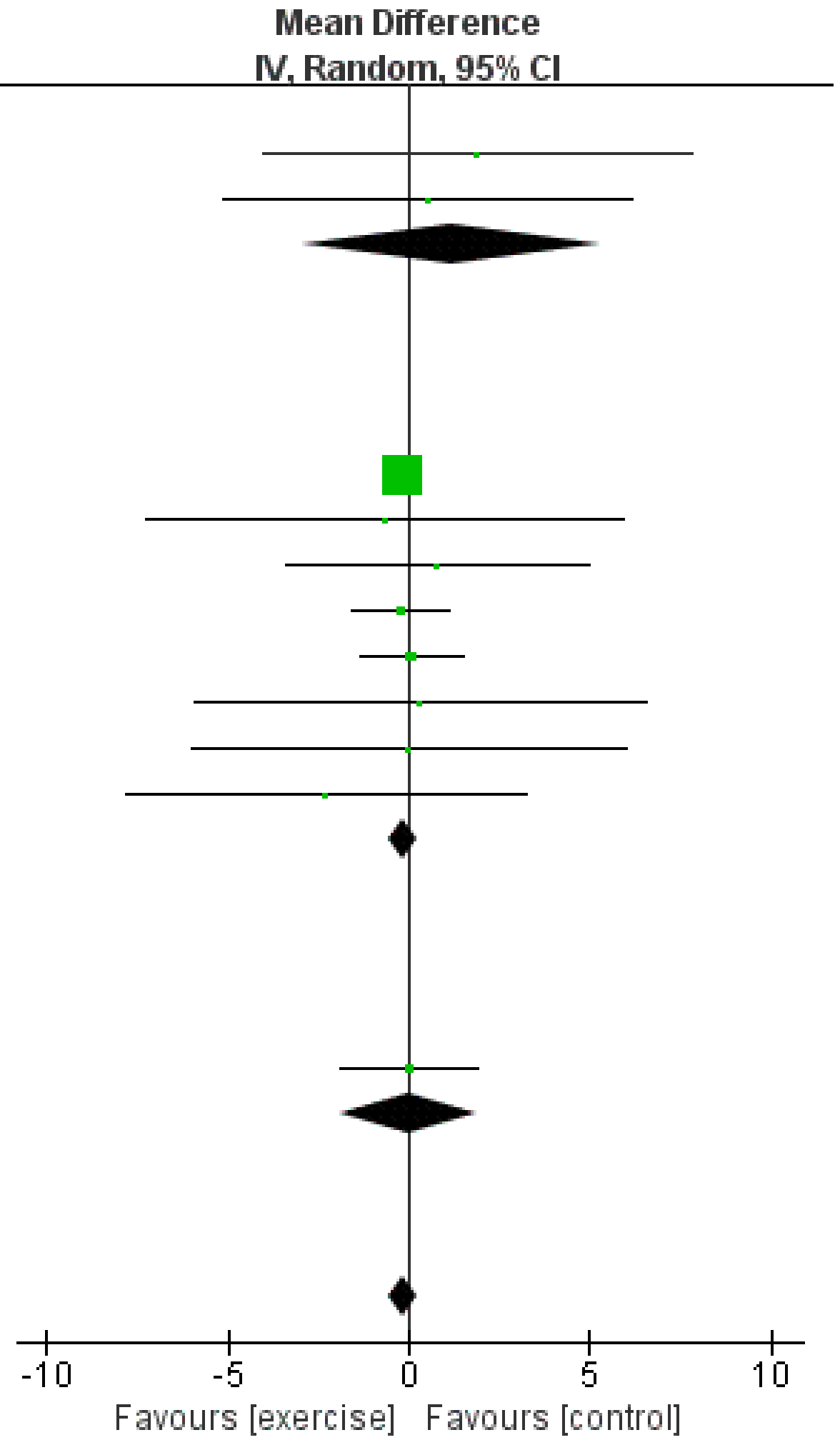

Supplementary Figure 37 Forest plot of subanalyses of Skinfold

Study or Subgroup	exercise Mean	exercise SD	exercise Total	Control Mean	Control SD	Control Total	Weight	Mean Difference IV, Random, 95% CI
Fatouros(2004)	-1.7	13.46	6	0.7	13.72	5	14.1%	-2.40 [-18.54, 13.74]
Fatouros(2005)a	-0.2	11.6	14	0.8	8.91	3	26.5%	-1.00 [-12.77, 10.77]
Fatouros(2005)b	-0.4	7.15	12	0.8	8.91	3	31.1%	-1.20 [-12.06, 9.66]
Fatouros(2005)c	-2	11.49	14	0.8	8.91	3	26.6%	-2.80 [-14.54, 8.94]
Schranz(2014)	-16.8	76.75	21	-15.5	75.03	22	1.8%	-1.30 [-46.69, 44.09]
**Total (95% CI)**			**67**			**36**	**100.0%**	**-1.74 [-7.80, 4.31]**
Heterogeneity: Tau² = 0.00; Chi² = 0.06, df = 4 (P = 1.00); I² = 0%								
Test for overall effect: Z = 0.56 (P = 0.57)								

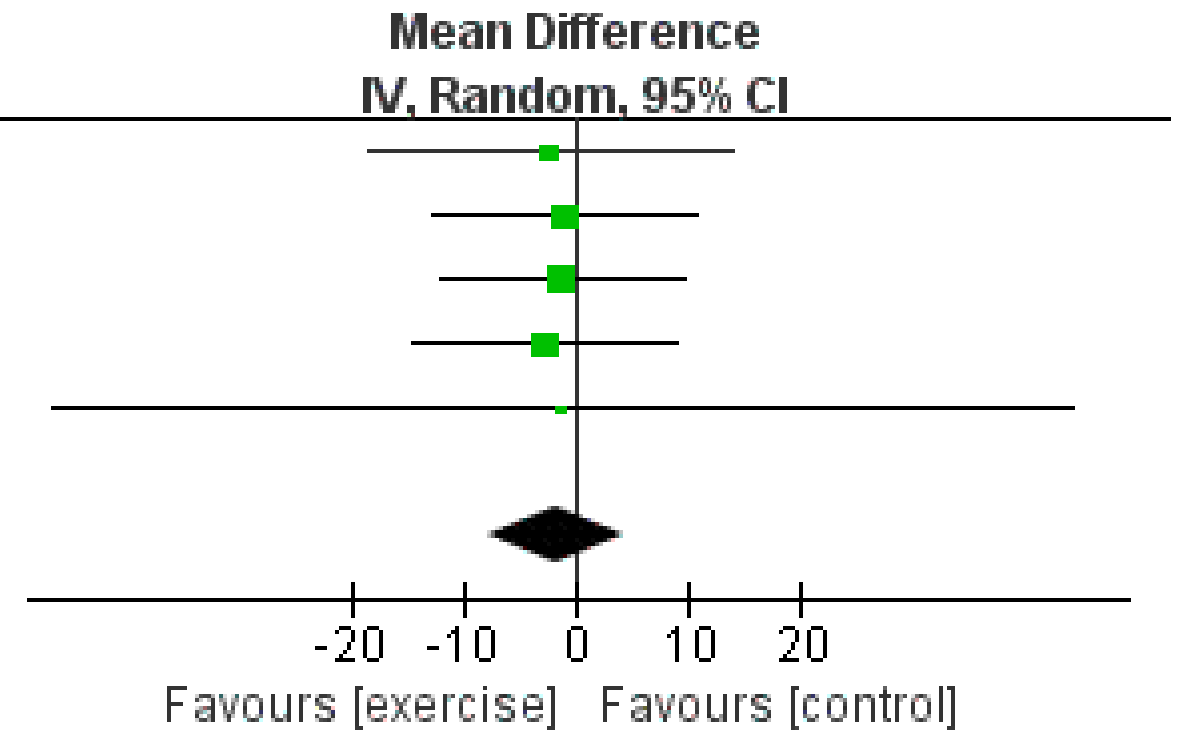

Supplementary Figure 38 Forest plot of subanalyses of Systolic blood pressure

Study or Subgroup	exercise Mean	exercise SD	exercise Total	Control Mean	Control SD	Control Total	Weight	Mean Difference IV, Random, 95% CI
**3.11.1 ≤ 2months**								
Bisconti(2020)a	2	7.55	13	1	5.57	6	11.6%	1.00 [-5.06, 7.06]
Bisconti(2020)b	-1	4.58	14	1	5.57	6	16.6%	-2.00 [-7.06, 3.06]
Chee Huei(2017)	-1.54	14.7	34	-0.68	12.74	85	13.4%	-0.86 [-6.49, 4.77]
Elliott(2002)	-4	17.78	8	1	18.03	7	1.3%	-5.00 [-23.17, 13.17]
Okamoto(2007)a	-4.7	9.13	7	-1.5	8.15	4	3.9%	-3.20 [-13.67, 7.27]
Okamoto(2007)b	-2.8	10.67	7	-1.5	8.15	4	3.4%	-1.30 [-12.54, 9.94]
Oliveira(2021)	-1.6	9.66	10	-0.8	10.65	9	5.0%	-0.80 [-9.98, 8.38]
Prasertsri(2019)	-5.62	8.57	18	-1.5	12.94	21	9.2%	-4.12 [-10.92, 2.68]
Shorki(2021)	-7.5	8.37	15	1.4	10.66	15	9.0%	-8.90 [-15.76, -2.04]
Sloan(2021)	-5.5	15.3	40	-1.34	14.34	58	11.8%	-4.16 [-10.17, 1.85]
Teixeira(2023)	-5	13.11	19	-4	12.12	18	6.4%	-1.00 [-9.13, 7.13]
Thompson(2010)	-2	11.14	21	-2	12.12	20	8.4%	0.00 [-7.14, 7.14]
**Subtotal (95% CI)**			**206**			**253**	**100.0%**	**-2.34 [-4.41, -0.28]**
Heterogeneity: Tau² = 0.00; Chi² = 6.35, df = 11 (P = 0.85); I² = 0%								
Test for overall effect: Z = 2.23 (P = 0.03)								
**3.11.2 2-6months**								
Hansen(2024)	1.39	54.11	8	-6.77	42.08	6	0.4%	8.16 [-42.23, 58.55]
Herring(2017)	-6.9	9.2	12	0.4	6.7	12	10.7%	-7.30 [-13.74, -0.86]
Kagioglou(2021)	2	14.93	12	0	12.24	10	5.4%	2.00 [-9.35, 13.35]
Krawcyk(2022)a	-7	19.31	31	-9	18.52	32	7.1%	2.00 [-7.35, 11.35]
Li(2015)	-7.59	10.35	101	-6.71	11.6	105	16.9%	-0.88 [-3.88, 2.12]
Miyachi(2004)	0	11.23	14	2	9.9	14	8.8%	-2.00 [-9.84, 5.84]
Nemoto(2024)	2.7	15.52	29	0.2	12.6	27	9.4%	2.50 [-4.88, 9.88]
Nordén(2024)	2	12.53	28	-2	13.53	29	10.2%	4.00 [-2.77, 10.77]
Stener-Victorin(2012)	-2.9	5.6	30	-2.3	7.1	15	14.8%	-0.60 [-4.71, 3.51]
Wang(2022)	-2.87	19.56	15	1.07	18.39	15	4.1%	-3.94 [-17.53, 9.65]
Zheng(2019)	-10.2	17.21	85	1.7	19.44	85	12.2%	-11.90 [-17.42, -6.38]
**Subtotal (95% CI)**			**365**			**350**	**100.0%**	**-1.89 [-4.98, 1.20]**
Heterogeneity: Tau² = 12.39; Chi² = 21.79, df = 10 (P = 0.02); I² = 54%								
Test for overall effect: Z = 1.20 (P = 0.23)								
**3.11.3 >6months**								
Brekke(2014)	-3	11	15	2.4	16	13	3.9%	-5.40 [-15.73, 4.93]
Brobakken(2024)	1	11.05	14	6	14.81	14	4.4%	-5.00 [-14.68, 4.68]
Gjellesvik(2020)	-2.56	15.26	28	-4.89	15.73	27	6.1%	2.33 [-5.86, 10.52]
Gram(2010)a	-5	23.01	21	-7	16.38	11	2.2%	2.00 [-11.80, 15.80]
Gram(2010)b	-10	17.53	24	-7	16.38	11	2.9%	-3.00 [-14.95, 8.95]
Haganes(2025)	-6	9.95	11	1.4	10.19	10	5.5%	-7.40 [-16.03, 1.23]
Krawcyk(2022)b	-6	19	29	-9	20.07	32	4.3%	3.00 [-6.81, 12.81]
O'Connor(2017)a	-2.2	14.98	12	-1.7	11.65	10	3.3%	-0.50 [-11.63, 10.63]
O'Connor(2017)b	1	13.25	10	-1.7	11.65	10	3.4%	2.70 [-8.24, 13.64]
Opdenacker(2011)	0.1	18.49	49	4.5	19.16	46	7.2%	-4.40 [-11.98, 3.18]
Zhang(2017)a	-4.9	9.37	69	-2.8	9.64	37	28.4%	-2.10 [-5.91, 1.71]
Zhang(2017)b	-7.2	9.15	66	-2.8	9.64	37	28.4%	-4.40 [-8.21, -0.59]
**Subtotal (95% CI)**			**348**			**258**	**100.0%**	**-2.69 [-4.73, -0.66]**
Heterogeneity: Tau² = 0.00; Chi² = 6.95, df = 11 (P = 0.80); I² = 0%								
Test for overall effect: Z = 2.60 (P = 0.009)								

Test for subgroup differences: Chi² = 0.19, df = 2 (P = 0.91), I² = 0%

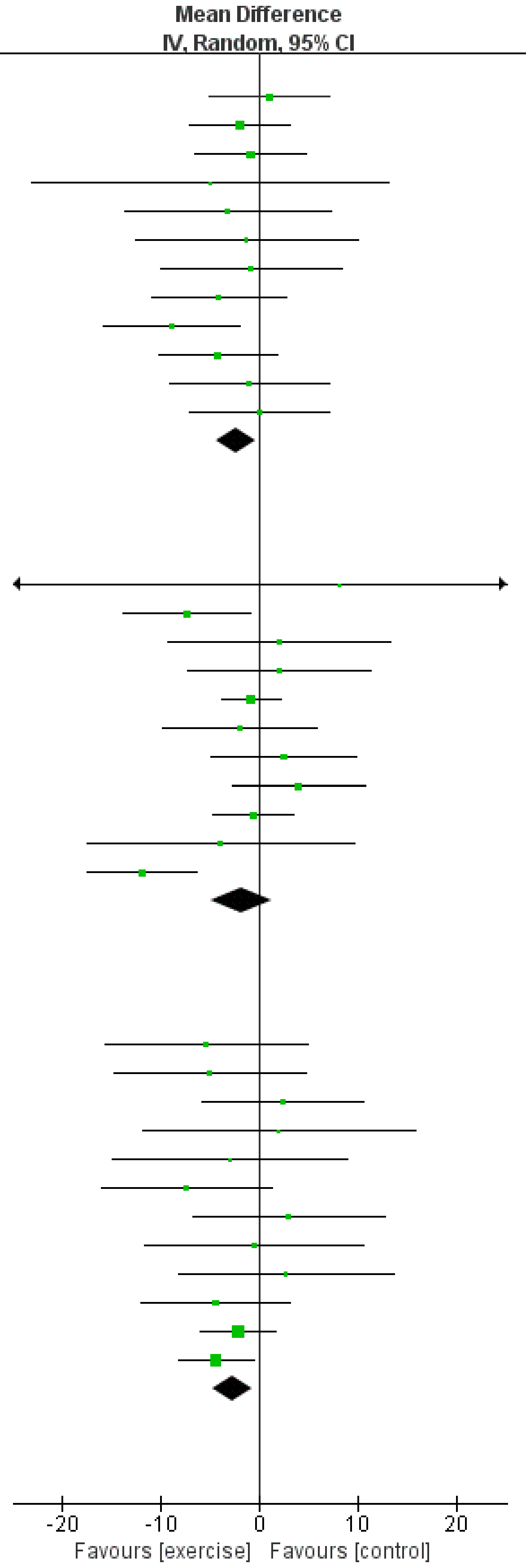

Supplementary Figure 39 Forest plot of subanalyses of Diastolic blood pressure

Study or Subgroup	exercise Mean	exercise SD	exercise Total	Control Mean	Control SD	Control Total	Weight	Mean Difference IV, Random, 95% CI
**3.12.1 ≤ 2months**								
Bisconti(2020)a	-3	10.54	13	-2	8	6	3.1%	-1.00 [-9.59, 7.59]
Bisconti(2020)b	-9	8	14	-2	8	6	3.8%	-7.00 [-14.65, 0.65]
Chee Huei(2017)	-1.21	9.73	34	-0.51	7.01	85	12.3%	-0.70 [-4.29, 2.89]
Elliott(2002)	-5	11	8	2	17.35	7	1.1%	-7.00 [-21.94, 7.94]
Okamoto(2007)a	-2.7	5.69	7	-0.2	4.56	4	5.5%	-2.50 [-8.64, 3.64]
Okamoto(2007)b	-4.2	6.75	7	-0.2	4.56	4	4.8%	-4.00 [-10.71, 2.71]
Oliveira(2021)	1.7	7.73	10	-4.2	8.05	9	4.3%	5.90 [-1.21, 13.01]
Prasertsri(2019)	-1.32	6.63	18	0.04	7.7	21	9.0%	-1.36 [-5.86, 3.14]
Shorki(2021)	-1.04	0.97	15	-0.9	1.26	15	30.9%	-0.14 [-0.94, 0.66]
Sloan(2021)	-6.94	8.79	40	-1.99	8.59	58	12.6%	-4.95 [-8.46, -1.44]
Teixeira(2023)	-3	8.19	19	-1.7	8.72	18	6.7%	-1.30 [-6.76, 4.16]
Thompson(2010)	-2	9	21	1	10.15	20	5.9%	-3.00 [-8.88, 2.88]
**Subtotal (95% CI)**			**206**			**253**	**100.0%**	**-1.59 [-3.17, -0.01]**
Heterogeneity: Tau² = 1.93; Chi² = 15.93, df = 11 (P = 0.14); I² = 31%								
Test for overall effect: Z = 1.97 (P = 0.05)								
**3.12.2 2-6months**								
Hansen(2024)	0.37	33.51	8	-5.61	28.65	6	0.5%	5.98 [-26.65, 38.61]
Herring(2017)	-5.2	5.6	12	2.7	4.3	12	12.0%	-7.90 [-11.89, -3.91]
Kagioglou(2021)	1.2	10.78	12	1.4	16	10	3.5%	-0.20 [-11.84, 11.44]
Krawcyk(2022)a	-4	9	31	-6	11.53	32	9.9%	2.00 [-3.10, 7.10]
Li(2015)	-3.95	8.53	101	-4.8	9.12	105	15.1%	0.85 [-1.56, 3.26]
Miyachi(2004)	1	6.48	14	4	6.47	14	10.5%	-3.00 [-7.80, 1.80]
Nemoto(2024)	0.1	10.14	29	-1	9.56	27	9.8%	1.10 [-4.06, 6.26]
Nordén(2024)	3	10	28	-2	8.54	29	10.4%	5.00 [0.16, 9.84]
Stener-Victorin(2012)	-2.9	5.1	30	-2.1	6.4	15	12.5%	-0.80 [-4.52, 2.92]
Wang(2022)	3.53	15.52	15	0.93	26.37	15	2.2%	2.60 [-12.88, 18.08]
Zheng(2019)	-3.05	10.05	85	0.93	11.02	85	13.6%	-3.98 [-7.15, -0.81]
**Subtotal (95% CI)**			**365**			**350**	**100.0%**	**-0.87 [-3.29, 1.56]**
Heterogeneity: Tau² = 8.61; Chi² = 26.08, df = 10 (P = 0.004); I² = 62%								
Test for overall effect: Z = 0.70 (P = 0.48)								
**3.12.3 >6months**								
Brekke(2014)	0	9.6	15	-2.1	10	13	6.2%	2.10 [-5.19, 9.39]
Brobakken(2024)	2	8.65	14	5	8.97	14	7.2%	-3.00 [-9.53, 3.53]
Gjellesvik(2020)	-2.56	15.26	28	-4.89	15.73	27	5.3%	2.33 [-5.86, 10.52]
Gram(2010)a	-4	10.35	21	-1	9.85	11	6.2%	-3.00 [-10.31, 4.31]
Gram(2010)b	-2	10.41	24	-1	9.85	11	6.4%	-1.00 [-8.16, 6.16]
Haganes(2025)	-0.8	7.14	11	2.4	8.94	10	6.6%	-3.20 [-10.16, 3.76]
Krawcyk(2022)b	0	8.19	29	-6	11.53	32	9.9%	6.00 [1.02, 10.98]
O'Connor(2017)a	-1	12.54	12	5.5	8.4	10	4.7%	-6.50 [-15.30, 2.30]
O'Connor(2017)b	-7.5	11.67	10	5.5	8.4	10	4.7%	-13.00 [-21.91, -4.09]
Opdenacker(2011)	-7.6	10.07	49	-5.4	10.39	46	11.8%	-2.20 [-6.32, 1.92]
Zhang(2017)a	-4.9	6.66	69	-1.1	6.86	37	15.4%	-3.80 [-6.51, -1.09]
Zhang(2017)b	-3.3	6.51	66	-1.1	6.86	37	15.5%	-2.20 [-4.91, 0.51]
**Subtotal (95% CI)**			**348**			**258**	**100.0%**	**-1.93 [-4.12, 0.26]**
Heterogeneity: Tau² = 6.17; Chi² = 21.08, df = 11 (P = 0.03); I² = 48%								
Test for overall effect: Z = 1.73 (P = 0.08)								

Test for subgroup differences: Chi² = 0.42, df = 2 (P = 0.81), I² = 0%

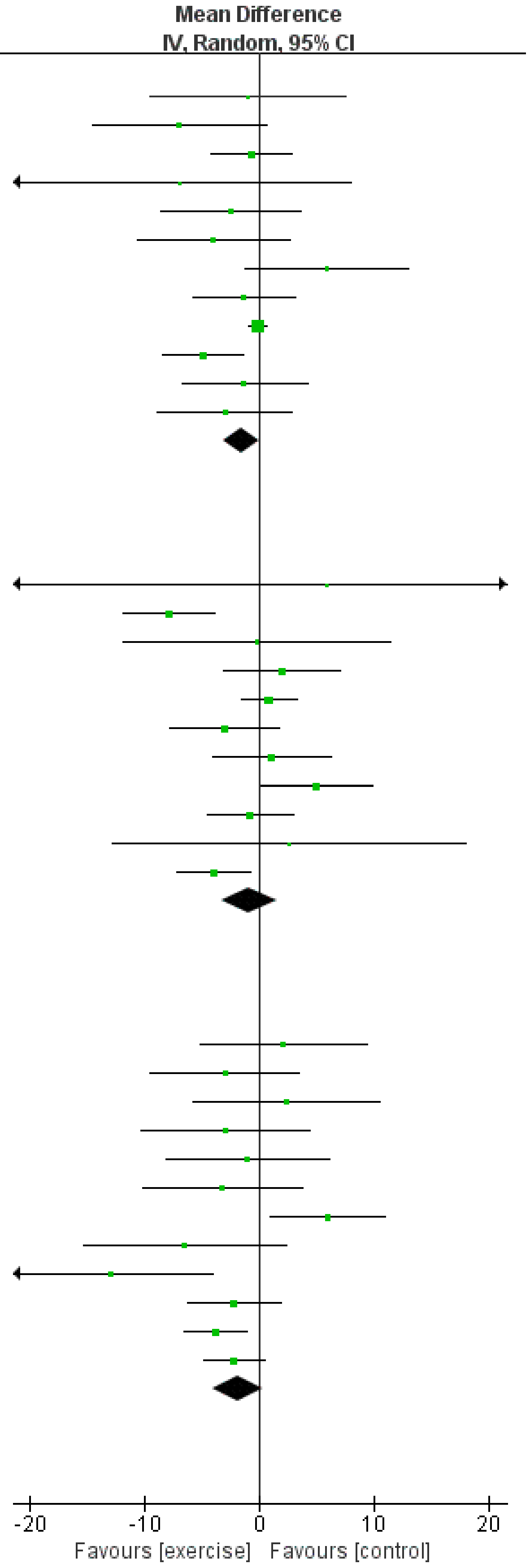

Supplementary Figure 40 Forest plot of subanalyses of Mean arterial pressure

Study or Subgroup	exercise Mean	exercise SD	exercise Total	Control Mean	Control SD	Control Total	Weight	Mean Difference IV, Random, 95% CI
**3.13.1 ≤ 2months**								
Bisconti(2020)a	-1	7	13	0	7.55	6	11.9%	-1.00 [-8.14, 6.14]
Bisconti(2020)b	-7	6.56	14	0	7.55	6	12.6%	-7.00 [-13.95, -0.05]
Elliott(2002)	-4	12.12	8	2	10.54	7	4.6%	-6.00 [-17.47, 5.47]
Okamoto(2007)a	-1.9	7.67	7	2.4	4.04	4	12.7%	-4.30 [-11.23, 2.63]
Okamoto(2007)b	-2.2	7.04	7	2.4	4.04	4	14.2%	-4.60 [-11.15, 1.95]
Prasertsri(2019)	-3.19	6.34	18	0.23	8.9	21	26.4%	-3.42 [-8.22, 1.38]
Teixeira(2023)	-3	9.54	19	-3.8	8.66	18	17.7%	0.80 [-5.07, 6.67]
**Subtotal (95% CI)**			**86**			**66**	**100.0%**	**-3.23 [-5.70, -0.77]**
Heterogeneity: Tau² = 0.00; Chi² = 3.81, df = 6 (P = 0.70); I² = 0%								
Test for overall effect: Z = 2.57 (P = 0.01)								
**Total (95% CI)**			**86**			**66**	**100.0%**	**-3.23 [-5.70, -0.77]**
Heterogeneity: Tau² = 0.00; Chi² = 3.81, df = 6 (P = 0.70); I² = 0%								
Test for overall effect: Z = 2.57 (P = 0.01)								
Test for subgroup differences: Not applicable								

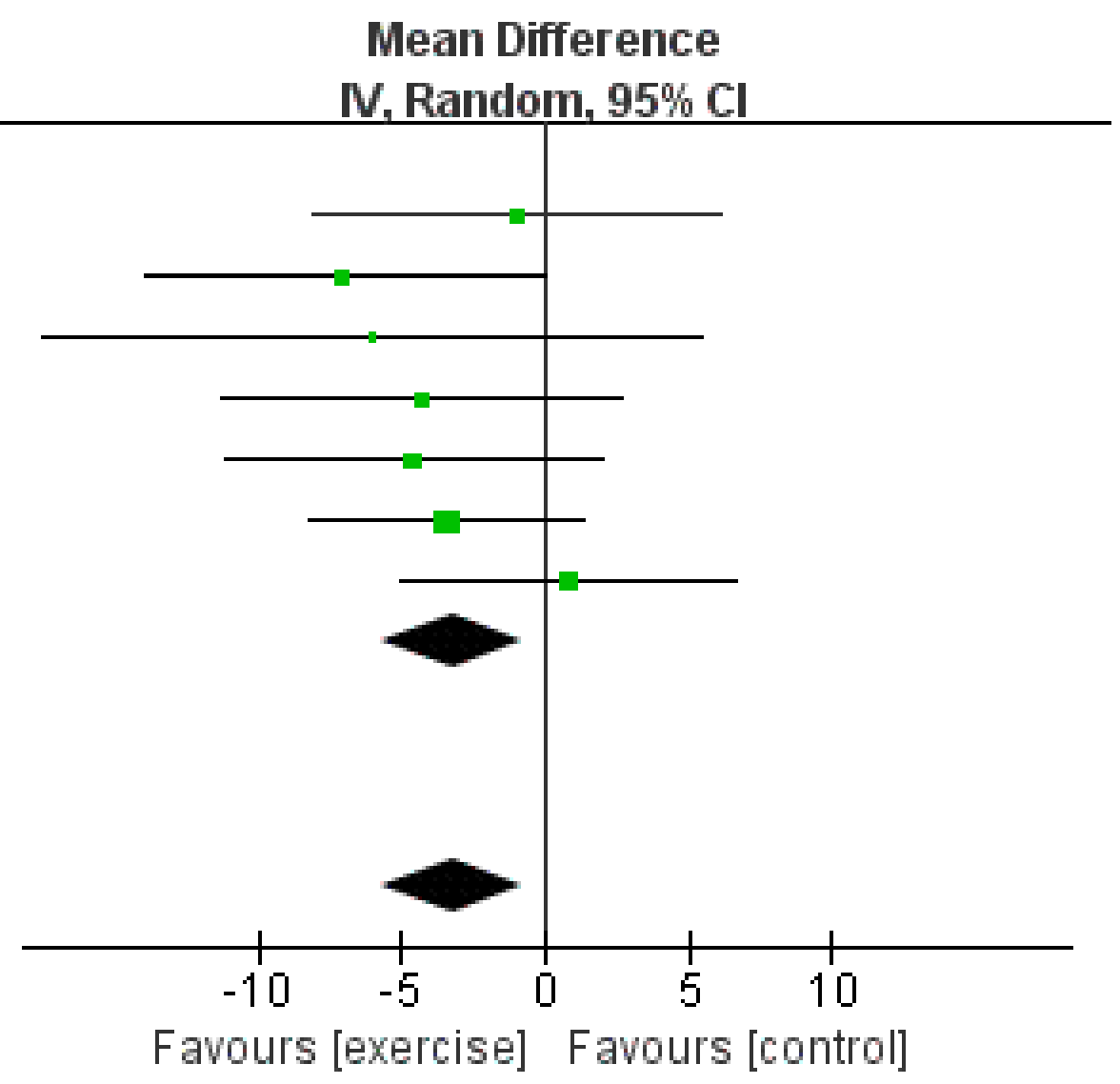

Supplementary Figure 41 Forest plot of subanalyses of Resting heart rate

Study or Subgroup	exercise Mean	exercise SD	exercise Total	Control Mean	Control SD	Control Total	Weight	Mean Difference IV, Random, 95% CI
**3.14.1 ≤ 2months**								
Bisconti(2020)a	-10	10.15	13	-7	8	6	3.4%	-3.00 [-11.45, 5.45]
Bisconti(2020)b	-9	8	14	-7	8	6	4.1%	-2.00 [-9.65, 5.65]
Niño(2017)a	1	15.72	10	1	8.54	3	1.3%	0.00 [-13.72, 13.72]
Niño(2017)b	-4	17.78	11	1	8.54	3	1.2%	-5.00 [-19.28, 9.28]
Niño(2017)c	-7	11.14	9	1	8.54	3	1.6%	-8.00 [-20.10, 4.10]
Okamoto(2007)a	-1.5	9.04	7	-0.4	9.24	4	1.9%	-1.10 [-12.36, 10.16]
Okamoto(2007)b	0	5.8	7	-0.4	9.24	4	2.4%	0.40 [-9.62, 10.42]
Oliveira(2021)	-1	7.55	10	-3	6.56	9	6.0%	2.00 [-4.35, 8.35]
Prasertsri(2019)	-3.43	10.68	18	-1.45	7.47	21	6.9%	-1.98 [-7.86, 3.90]
Shorki(2021)	-1.08	8.39	15	-1.76	6.98	15	7.9%	0.68 [-4.84, 6.20]
Sloan(2021)	-0.86	8.51	40	-1.88	8.55	58	20.3%	1.02 [-2.41, 4.45]
Stebbings(2013)a	0.76	3.87	10	-1.01	3.67	12	23.8%	1.77 [-1.40, 4.94]
Stebbings(2013)b	2.08	5.56	14	-1.74	3.48	6	14.8%	3.82 [-0.21, 7.85]
Teixeira(2023)	-4	13.45	19	-1	9.17	18	4.4%	-3.00 [-10.38, 4.38]
**Subtotal (95% CI)**			**197**			**168**	**100.0%**	**0.71 [-0.84, 2.26]**
Heterogeneity: Tau² = 0.00; Chi² = 8.62, df = 13 (P = 0.80); I² = 0%								
Test for overall effect: Z = 0.90 (P = 0.37)								
**3.14.2 2-6months**								
Hamdorf(1993)	-2	10.29	27	-2	9.28	31	8.8%	0.00 [-5.07, 5.07]
Herring(2017)	-5	8.8	12	-3.4	9.3	12	4.3%	-1.60 [-8.84, 5.64]
Kagioglou(2021)	-3.6	8.57	12	-0.7	6.64	10	5.6%	-2.90 [-9.26, 3.46]
Li(2015)	-0.93	18.52	101	4.51	13.86	105	11.3%	-5.44 [-9.92, -0.96]
Malandish(2020)	-2.36	9.56	13	1	10.07	13	4.0%	-3.36 [-10.91, 4.19]
Miyachi(2004)	-2	7.03	14	0	7.38	14	7.9%	-2.00 [-7.34, 3.34]
Nemoto(2024)	2.4	8.96	29	4.8	14	27	5.9%	-2.40 [-8.61, 3.81]
Nordén(2024)	-1	8.54	27	-5	10.58	29	9.0%	4.00 [-1.02, 9.02]
Stener-Victorin(2012)	-1.7	8	30	-1.2	6.9	15	11.1%	-0.50 [-5.02, 4.02]
Zheng(2019)	-6.2	8.8	85	-4.7	8.8	85	32.3%	-1.50 [-4.15, 1.15]
**Subtotal (95% CI)**			**350**			**341**	**100.0%**	**-1.46 [-2.96, 0.05]**
Heterogeneity: Tau² = 0.00; Chi² = 8.64, df = 9 (P = 0.47); I² = 0%								
Test for overall effect: Z = 1.90 (P = 0.06)								
**3.14.3 >6months**								
Haganes(2025)	-0.5	9.17	12	-4.2	7.88	9	47.0%	3.70 [-3.61, 11.01]
Nogueira(2017)	-6	5.57	155	5	7.21	85	53.0%	-11.00 [-12.77, -9.23]
**Subtotal (95% CI)**			**167**			**94**	**100.0%**	**-4.10 [-18.47, 10.28]**
Heterogeneity: Tau² = 100.69; Chi² = 14.68, df = 1 (P = 0.0001); I² = 93%								
Test for overall effect: Z = 0.56 (P = 0.58)								

Mean Difference IV, Random, 95% CI

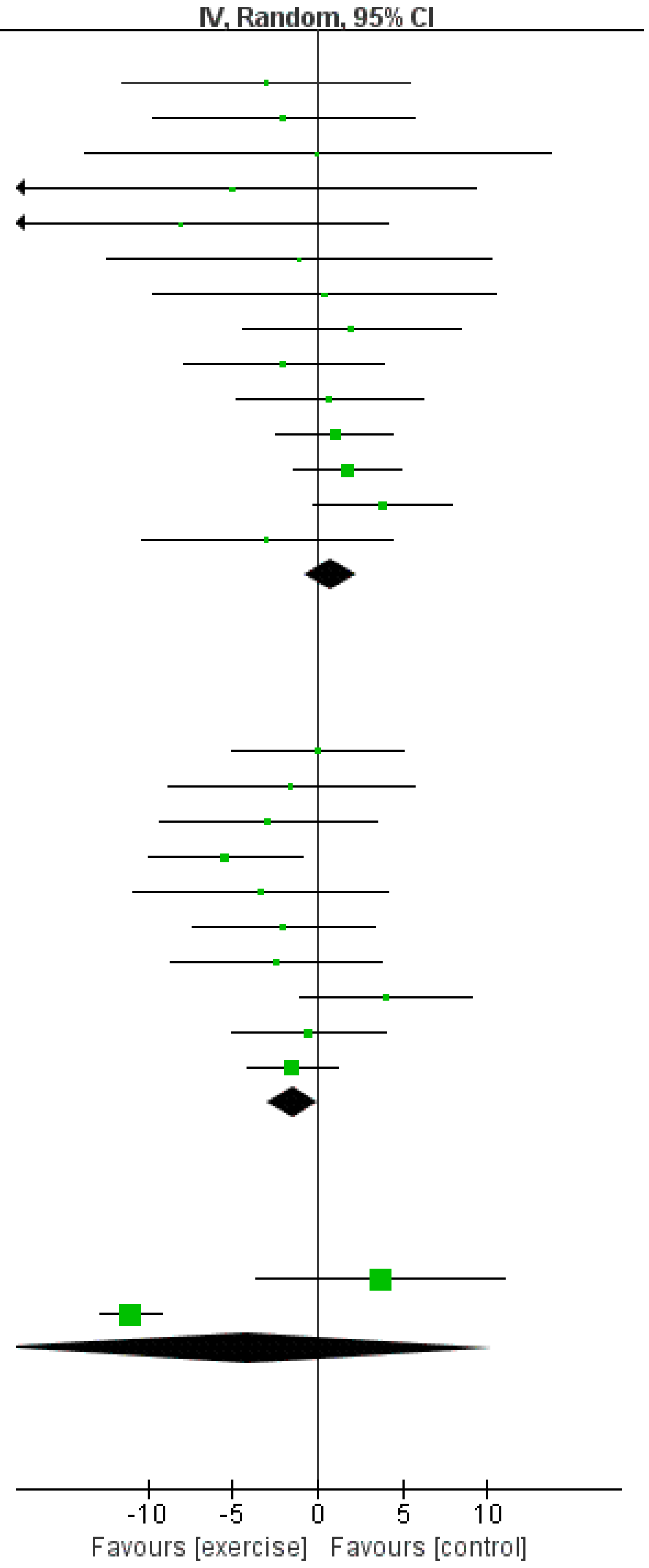

Test for subgroup differences: Chi² = 4.13, df = 2 (P = 0.13), I² = 51.6%

Supplementary Figure 42 Forest plot of subanalyses of VO_{2peak}

	exercise			Control				Std. Mean Difference
**Study or Subgroup**	**Mean**	**SD**	**Total**	**Mean**	**SD**	**Total**	**Weight**	**IV, Random, 95% CI**
**3.15.1 ≤ 2months**								
Islam(2020)a	0	6	27	0	6	8	12.5%	0.00 [-0.79, 0.79]
Islam(2020)b	3	7.55	26	0	6	8	12.4%	0.40 [-0.39, 1.20]
Nikseresht(2016)a	4.1	1.55	10	-0.3	4.8	6	7.6%	1.33 [0.19, 2.47]
Nikseresht(2016)b	1.1	3.65	12	-0.3	4.8	6	9.4%	0.33 [-0.66, 1.32]
Nuri(2016)	6.3	9.84	15	-0.78	14.27	15	13.7%	0.56 [-0.17, 1.29]
Shorki(2021)	0.98	5.57	15	1.05	6.44	15	14.0%	-0.01 [-0.73, 0.70]
Sosa(2012)	0.5	5.97	10	-2.9	7.09	8	9.9%	0.50 [-0.45, 1.45]
Sosa(2014)	5.4	3.42	10	-4.1	4.89	7	6.4%	2.21 [0.93, 3.50]
Teixeira(2023)	-3	4.26	17	-4.3	5.07	14	14.1%	0.27 [-0.44, 0.98]
**Subtotal (95% CI)**			**142**			**87**	**100.0%**	**0.49 [0.12, 0.85]**
Heterogeneity: Tau² = 0.11; Chi² = 12.76, df = 8 (P = 0.12); I² = 37%								
Test for overall effect: Z = 2.62 (P = 0.009)								
**3.15.2 2-6months**								
Brooker(2023)a	3.03	4.77	42	0.09	3.29	13	18.3%	0.65 [0.01, 1.28]
Brooker(2023)b	2.41	4.98	46	0.09	3.29	13	19.0%	0.49 [-0.13, 1.11]
Fatouros(2004)	0.9	1.9	6	-1	2.29	5	4.6%	0.83 [-0.43, 2.10]
Kagioglou(2021)	0.3	2.2	12	-0.1	2.15	10	10.4%	0.18 [-0.66, 1.02]
Kindred(2019)a	4.75	7.03	20	1.3	8.97	26	21.2%	0.41 [-0.18, 1.00]
Nordén(2024)	0.1	2	27	-0.04	3	28	26.4%	0.05 [-0.47, 0.58]
**Subtotal (95% CI)**			**153**			**95**	**100.0%**	**0.37 [0.10, 0.64]**
Heterogeneity: Tau² = 0.00; Chi² = 2.99, df = 5 (P = 0.70); I² = 0%								
Test for overall effect: Z = 2.68 (P = 0.007)								
**3.15.3 >6months**								
Anandavadivelan(2024)a	0.58	9.13	93	-0.15	8.7	36	18.1%	0.08 [-0.30, 0.47]
Anandavadivelan(2024)b	2.1	8.95	90	-0.15	8.7	36	17.8%	0.25 [-0.14, 0.64]
Frost(2020)a	-1.43	3.17	34	-0.99	3.85	16	7.6%	-0.13 [-0.72, 0.47]
Frost(2020)b	0.78	3.61	33	-0.99	3.85	16	7.3%	0.47 [-0.13, 1.08]
Gjellesvik(2020)	1.21	10.73	28	-2.44	8.47	28	9.6%	0.37 [-0.16, 0.90]
Haganes(2025)	0.6	5.28	12	-0.3	4.94	9	3.6%	0.17 [-0.70, 1.03]
Kindred(2019)b	2.67	7.36	20	0.85	9.15	26	7.8%	0.21 [-0.37, 0.80]
O'Connor(2017)a	3.9	5.77	12	4.1	4.52	10	3.8%	-0.04 [-0.88, 0.80]
O'Connor(2017)b	-0.7	3.95	10	4.1	4.52	10	3.0%	-1.08 [-2.04, -0.13]
Opdenacker(2011)	3.5	5.77	49	3.1	5.58	46	16.5%	0.07 [-0.33, 0.47]
Vainshelboim(2016)	0.1	2	14	-0.4	3	14	4.9%	0.19 [-0.55, 0.93]
**Subtotal (95% CI)**			**395**			**247**	**100.0%**	**0.13 [-0.03, 0.29]**
Heterogeneity: Tau² = 0.00; Chi² = 9.77, df = 10 (P = 0.46); I² = 0%								
Test for overall effect: Z = 1.56 (P = 0.12)								

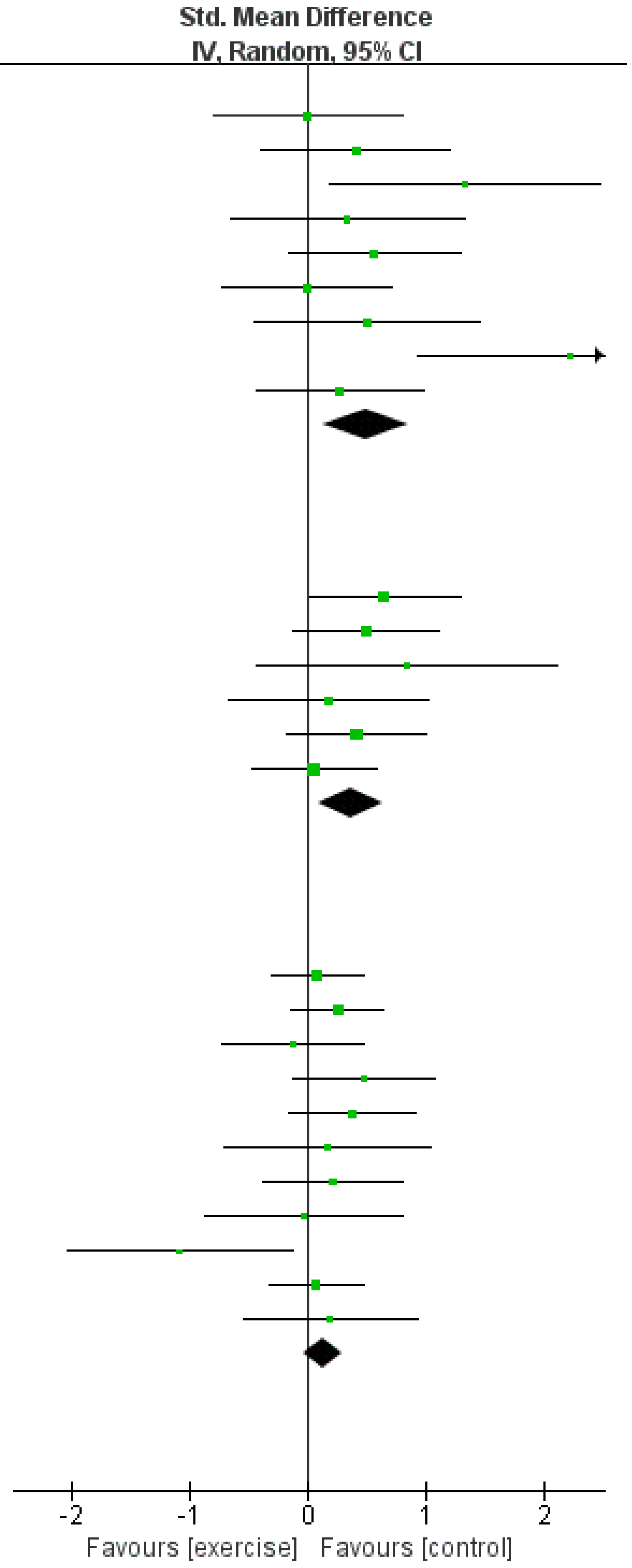

Test for subgroup differences: Chi² = 4.36, df = 2 (P = 0.11), I² = 54.1%

Supplementary Figure 43 Forest plot of subanalyses of VO_{2max}

Study or Subgroup	exercise Mean	exercise SD	exercise Total	Control Mean	Control SD	Control Total	Weight	Std. Mean Difference IV, Random, 95% CI
**3.16.1 ≤ 2months**								
Broderick(2013)	2.9	3.53	20	1.2	3.92	18	13.9%	0.45 [-0.20, 1.09]
Liu(2023)	3.3	3.21	11	0	4.4	9	6.7%	0.83 [-0.09, 1.76]
Lovell(2011)	0.2	3.46	12	0.3	3.99	12	9.0%	-0.03 [-0.83, 0.77]
Moghadasi(2012)	3.9	4.7	8	0.09	5.7	8	5.6%	0.69 [-0.33, 1.71]
Niño(2017)a	6.2	9.41	10	-1.8	7.39	3	3.2%	0.82 [-0.52, 2.16]
Niño(2017)b	3	5.89	11	-1.8	7.39	3	3.3%	0.73 [-0.59, 2.04]
Niño(2017)c	6.1	7.13	9	-1.8	7.39	3	3.0%	1.02 [-0.38, 2.41]
Rosety-Rodriguez(2014)a	2.8	6.18	11	0.3	5.55	9	7.3%	0.41 [-0.49, 1.30]
Sloan(2018)	2.89	7.7	40	-1.62	6.45	58	33.9%	0.64 [0.23, 1.05]
Thompson(2010)	1.8	4.91	21	-2.2	4.6	20	14.1%	0.82 [0.18, 1.46]
**Subtotal (95% CI)**			**153**			**143**	**100.0%**	**0.60 [0.36, 0.84]**
Heterogeneity: Tau² = 0.00; Chi² = 4.01, df = 9 (P = 0.91); I² = 0%								
Test for overall effect: Z = 4.87 (P < 0.00001)								
**3.16.2 2-6months**								
Batrakoulis(2018)	2.1	3.25	14	-0.5	3.15	21	31.1%	0.80 [0.09, 1.50]
Fatouros(2005)a	0.4	2.88	14	-0.4	2.7	3	9.9%	0.27 [-0.99, 1.52]
Fatouros(2005)b	0.3	2.6	12	-0.4	2.7	3	9.6%	0.25 [-1.02, 1.52]
Fatouros(2005)c	0.7	2.52	14	-0.4	2.7	3	9.8%	0.41 [-0.85, 1.67]
Rosety-Rodriguez(2014)b	1.1	6.14	22	-0.1	5.43	18	39.6%	0.20 [-0.42, 0.83]
**Subtotal (95% CI)**			**76**			**48**	**100.0%**	**0.42 [0.03, 0.81]**
Heterogeneity: Tau² = 0.00; Chi² = 1.69, df = 4 (P = 0.79); I² = 0%								
Test for overall effect: Z = 2.09 (P = 0.04)								
**3.16.3 >6months**								
Nogueira(2017)	5.7	7.35	155	3.9	7.05	85	66.5%	0.25 [-0.02, 0.51]
Øiestad(2023)a	1.4	7.91	45	-2	6.6	21	17.1%	0.45 [-0.08, 0.97]
Øiestad(2023)b	-0.5	4.55	39	-2	6.6	21	16.5%	0.28 [-0.26, 0.81]
**Subtotal (95% CI)**			**239**			**127**	**100.0%**	**0.29 [0.07, 0.50]**
Heterogeneity: Tau² = 0.00; Chi² = 0.44, df = 2 (P = 0.80); I² = 0%								
Test for overall effect: Z = 2.59 (P = 0.009)								

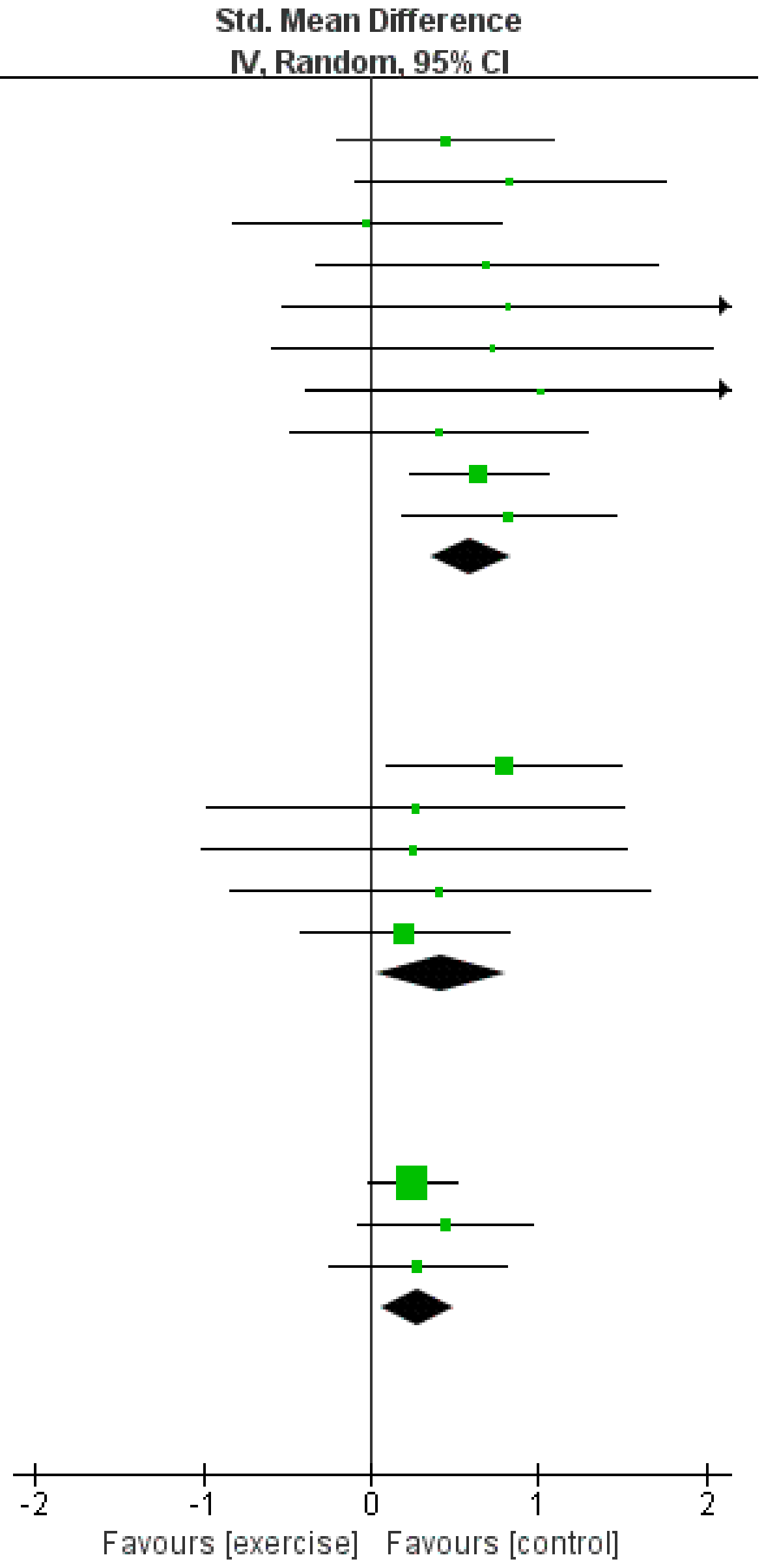

Test for subgroup differences: Chi² = 3.56, df = 2 (P = 0.17), I² = 43.9%

Supplementary Figure 44 Forest plot of subanalyses of Total cholesterol

Study or Subgroup	exercise Mean	exercise SD	exercise Total	Control Mean	Control SD	Control Total	Weight	Std. Mean Difference IV, Random, 95% CI
**3.17.1 ≤ 2months**								
Elliott(2002)	-1	1.46	8	-0.08	1.14	7	8.5%	-0.65 [-1.71, 0.40]
Kranen(2023)	-0.36	0.53	10	-0.36	0.48	9	9.5%	0.00 [-0.90, 0.90]
Nikseresht(2016)a	-0.5	0.82	10	-0.44	1.16	6	8.7%	-0.06 [-1.07, 0.95]
Nikseresht(2016)b	-0.14	0.57	12	-0.44	1.16	6	8.9%	0.36 [-0.63, 1.34]
Prasertsri(2019)	0.12	0.94	18	-0.08	1.11	21	11.3%	0.19 [-0.44, 0.82]
Shorki(2021)	-0.63	0.14	15	-0.06	0.18	15	7.7%	-3.44 [-4.62, -2.26]
Stergioulas(2006)	0.14	1.49	21	0.04	1.64	17	11.3%	0.06 [-0.58, 0.70]
Teixeira(2023)	-0.04	0.94	19	-0.19	1.13	17	11.2%	0.14 [-0.51, 0.80]
Theodorou(2016)a	-0.41	2.78	41	-0.11	2.43	15	11.6%	-0.11 [-0.70, 0.48]
Thompson(2010)	-0.13	1.06	21	-0.26	0.78	20	11.5%	0.14 [-0.48, 0.75]
**Subtotal (95% CI)**			**175**			**133**	**100.0%**	**-0.25 [-0.73, 0.24]**
Heterogeneity: Tau² = 0.44; Chi² = 35.05, df = 9 (P < 0.0001); I² = 74%								
Test for overall effect: Z = 0.99 (P = 0.32)								
**3.17.2 2-6months**								
Hansen(2024)	0.25	1.52	8	-0.13	1.82	6	3.8%	0.22 [-0.85, 1.28]
Martins(2010)a	-0.25	0.85	18	-0.07	0.99	7	5.6%	-0.20 [-1.07, 0.68]
Martins(2010)b	-0.18	0.83	14	-0.07	0.99	7	5.2%	-0.12 [-1.03, 0.79]
Nemoto(2024)	0.12	0.91	29	0.19	0.92	27	15.7%	-0.08 [-0.60, 0.45]
Nordén(2024)	-0.7	1.28	28	-0.5	1.1	30	16.2%	-0.17 [-0.68, 0.35]
Theodorou(2016)b	-0.29	2.77	41	-0.07	2.75	15	12.3%	-0.08 [-0.67, 0.51]
Zheng(2019)	0.27	0.79	79	0.46	1.06	69	41.1%	-0.20 [-0.53, 0.12]
**Subtotal (95% CI)**			**217**			**161**	**100.0%**	**-0.14 [-0.35, 0.07]**
Heterogeneity: Tau² = 0.00; Chi² = 0.71, df = 6 (P = 0.99); I² = 0%								
Test for overall effect: Z = 1.33 (P = 0.18)								
**3.17.3 >6months**								
Brekke(2014)	-0.71	0.59	15	-0.75	0.6	13	15.1%	0.07 [-0.68, 0.81]
Brobakken(2024)	0.29	1.25	14	0.31	1.15	14	15.2%	-0.02 [-0.76, 0.72]
Gjellesvik(2020)	-0.06	1.03	23	0.13	1.14	26	26.3%	-0.17 [-0.73, 0.39]
Gram(2010)a	-0.33	0.81	21	-0.19	1.3	11	15.6%	-0.14 [-0.87, 0.59]
Gram(2010)b	0.21	0.89	24	-0.19	1.3	11	16.1%	0.38 [-0.34, 1.10]
Haganes(2025)	0.1	0.7	12	0	1.1	10	11.8%	0.11 [-0.73, 0.95]
**Subtotal (95% CI)**			**109**			**85**	**100.0%**	**0.01 [-0.27, 0.30]**
Heterogeneity: Tau² = 0.00; Chi² = 1.64, df = 5 (P = 0.90); I² = 0%								
Test for overall effect: Z = 0.10 (P = 0.92)								

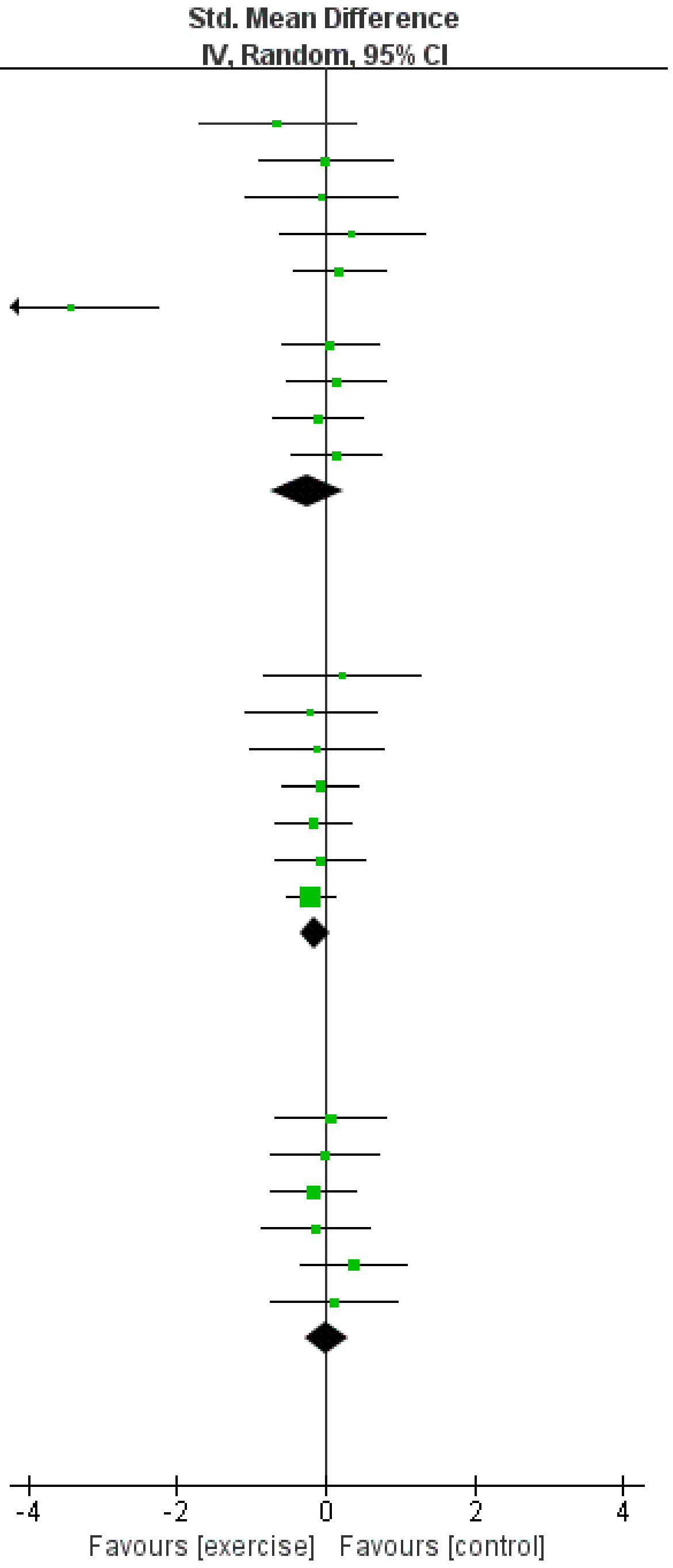

Test for subgroup differences: Chi² = 1.10, df = 2 (P = 0.58), I² = 0%

Supplementary Figure 45 Forest plot of subanalyses of HDL-C

Study or Subgroup	exercise Mean	exercise SD	exercise Total	Control Mean	Control SD	Control Total	Weight	Std. Mean Difference IV, Random, 95% CI
**3.18.1 ≤ 2months**								
Chee Huei(2017)	0.02	0.16	34	0.02	0.15	82	15.1%	0.00 [-0.40, 0.40]
Elliott(2002)	0.05	0.39	8	-0.26	0.44	7	6.9%	0.71 [-0.35, 1.76]
Kranen(2023)	-0.06	0.3	10	-0.15	0.33	9	8.3%	0.27 [-0.63, 1.18]
Nikseresht(2016)a	0.15	0.2	10	-0.01	0.25	6	7.0%	0.69 [-0.36, 1.74]
Nikseresht(2016)b	0.07	0.2	12	-0.01	0.25	6	7.5%	0.35 [-0.64, 1.34]
Prasertsri(2019)	0.13	0.3	18	-0.02	0.13	21	11.4%	0.65 [0.01, 1.30]
Shorki(2021)	0.21	0.08	15	0.04	0.09	15	8.5%	1.94 [1.05, 2.83]
Teixeira(2023)	-0.06	0.14	19	-0.03	0.25	17	11.3%	-0.15 [-0.80, 0.51]
Theodorou(2016)a	-0.02	0.88	41	0.07	1.08	15	12.2%	-0.09 [-0.69, 0.50]
Thompson(2010)	0.04	0.22	21	-0.03	0.22	20	11.8%	0.31 [-0.30, 0.93]
**Subtotal (95% CI)**			**188**			**198**	**100.0%**	**0.39 [0.04, 0.74]**
Heterogeneity: Tau² = 0.17; Chi² = 21.01, df = 9 (P = 0.01); I² = 57%								
Test for overall effect: Z = 2.21 (P = 0.03)								
**3.18.2 2-6months**								
Hansen(2024)	0.05	0.64	8	-0.04	0.34	6	3.8%	0.16 [-0.90, 1.22]
Martins(2010)a	-0.03	0.27	18	-0.04	0.25	7	5.7%	0.04 [-0.84, 0.91]
Martins(2010)b	-0.02	0.24	14	-0.04	0.25	7	5.2%	0.08 [-0.83, 0.99]
Nemoto(2024)	0.06	0.51	29	0.05	0.37	27	15.7%	0.02 [-0.50, 0.55]
Nordén(2024)	0.1	0.56	28	0	0.56	30	16.2%	0.18 [-0.34, 0.69]
Theodorou(2016)b	0.06	1.04	41	0.02	3.52	15	12.3%	0.02 [-0.57, 0.61]
Zheng(2019)	-0.8	0.83	79	-1.08	1.09	69	41.0%	0.29 [-0.03, 0.61]
**Subtotal (95% CI)**			**217**			**161**	**100.0%**	**0.17 [-0.04, 0.37]**
Heterogeneity: Tau² = 0.00; Chi² = 1.21, df = 6 (P = 0.98); I² = 0%								
Test for overall effect: Z = 1.56 (P = 0.12)								
**3.18.3 >6months**								
Brekke(2014)	-0.19	0.19	15	-0.26	0.3	13	15.5%	0.28 [-0.47, 1.02]
Brobakken(2024)	-0.2	0.31	14	-0.01	0.33	14	15.1%	-0.58 [-1.33, 0.18]
Gjellesvik(2020)	-0.04	0.42	23	0	0.45	26	24.7%	-0.09 [-0.65, 0.47]
Gram(2010)a	1.15	0.26	21	1.3	0.32	11	15.7%	-0.52 [-1.26, 0.22]
Gram(2010)b	1.54	0.85	24	1.3	0.32	11	16.6%	0.32 [-0.40, 1.04]
Haganes(2025)	0.2	0.3	12	0.1	0.3	10	12.5%	0.32 [-0.53, 1.17]
**Subtotal (95% CI)**			**109**			**85**	**100.0%**	**-0.05 [-0.37, 0.26]**
Heterogeneity: Tau² = 0.02; Chi² = 5.90, df = 5 (P = 0.32); I² = 15%								
Test for overall effect: Z = 0.34 (P = 0.73)								

Test for subgroup differences: Chi² = 3.49, df = 2 (P = 0.18), I² = 42.6%

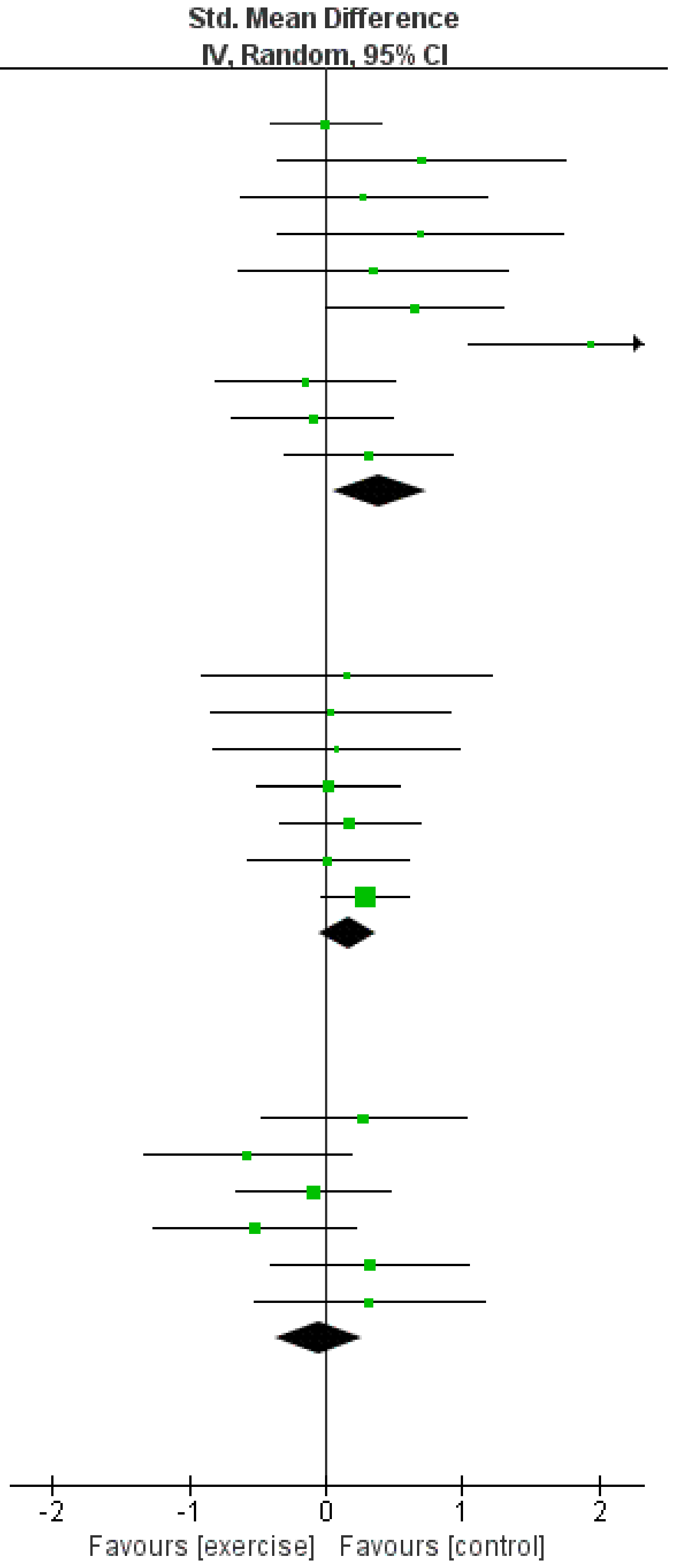

Supplementary Figure 46 Forest plot of subanalyses of LDL-C

	exercise			Control				Std. Mean Difference
**Study or Subgroup**	**Mean**	**SD**	**Total**	**Mean**	**SD**	**Total**	**Weight**	**IV, Random, 95% CI**
**3.19.1 ≤ 2months**								
Elliott(2002)	-0.94	1.46	8	-0.37	1.07	7	11.6%	-0.41 [-1.44, 0.61]
Kranen(2023)	-0.28	0.44	10	-0.25	0.63	9	12.4%	-0.05 [-0.95, 0.85]
Nikseresht(2016)a	-0.61	0.59	10	-0.33	0.6	6	11.6%	-0.45 [-1.47, 0.58]
Nikseresht(2016)b	-0.53	0.61	12	-0.33	0.6	6	11.9%	-0.31 [-1.30, 0.67]
Prasertsri(2019)	-0.06	0.896	18	-0.05	1.01	21	14.1%	-0.01 [-0.64, 0.62]
Shorki(2021)	-0.62	0.13	15	-0.04	0.16	15	10.1%	-3.87 [-5.14, -2.60]
Teixeira(2023)	0.11	0.88	19	-0.11	1.02	17	14.0%	0.23 [-0.43, 0.88]
Theodorou(2016)a	-0.21	3.58	41	-0.02	0.91	15	14.4%	-0.06 [-0.65, 0.53]
**Subtotal (95% CI)**			**133**			**96**	**100.0%**	**-0.51 [-1.17, 0.14]**
Heterogeneity: Tau² = 0.68; Chi² = 34.57, df = 7 (P < 0.0001); I² = 80%								
Test for overall effect: Z = 1.53 (P = 0.12)								
**3.19.2 2-6months**								
Hansen(2024)	0.25	1.26	8	-0.08	1.34	6	3.8%	0.24 [-0.82, 1.30]
Martins(2010)a	-0.63	0.72	18	-0.74	0.74	7	5.7%	0.15 [-0.73, 1.02]
Martins(2010)b	-0.25	0.74	14	-0.74	0.74	7	5.0%	0.64 [-0.30, 1.57]
Nemoto(2024)	0.09	0.8	29	0.15	0.78	27	15.7%	-0.07 [-0.60, 0.45]
Nordén(2024)	-0.7	1.15	28	-0.5	1.05	30	16.2%	-0.18 [-0.70, 0.34]
Theodorou(2016)b	-0.09	3.62	41	0.02	3.52	15	12.3%	-0.03 [-0.62, 0.56]
Zheng(2019)	0.06	0.28	79	0.09	0.03	69	41.3%	-0.15 [-0.47, 0.18]
**Subtotal (95% CI)**			**217**			**161**	**100.0%**	**-0.06 [-0.26, 0.15]**
Heterogeneity: Tau² = 0.00; Chi² = 3.14, df = 6 (P = 0.79); I² = 0%								
Test for overall effect: Z = 0.52 (P = 0.60)								
**3.19.3 >6months**								
Brekke(2014)	-0.47	0.47	15	-0.46	0.68	13	15.1%	-0.02 [-0.76, 0.73]
Brobakken(2024)	0.72	1.21	14	0.39	0.97	14	15.0%	0.29 [-0.45, 1.04]
Gjellesvik(2020)	-0.1	0.93	23	0.05	0.97	26	26.4%	-0.16 [-0.72, 0.41]
Gram(2010)a	-0.76	0.77	21	-0.07	3.12	11	15.4%	-0.35 [-1.09, 0.38]
Gram(2010)b	0.03	1.73	24	-0.07	3.12	11	16.3%	0.04 [-0.67, 0.76]
Haganes(2025)	0	0.75	12	0	1.05	10	11.8%	0.00 [-0.84, 0.84]
**Subtotal (95% CI)**			**109**			**85**	**100.0%**	**-0.05 [-0.34, 0.24]**
Heterogeneity: Tau² = 0.00; Chi² = 1.68, df = 5 (P = 0.89); I² = 0%								
Test for overall effect: Z = 0.32 (P = 0.75)								

Test for subgroup differences: Chi² = 1.78, df = 2 (P = 0.41), I² = 0%

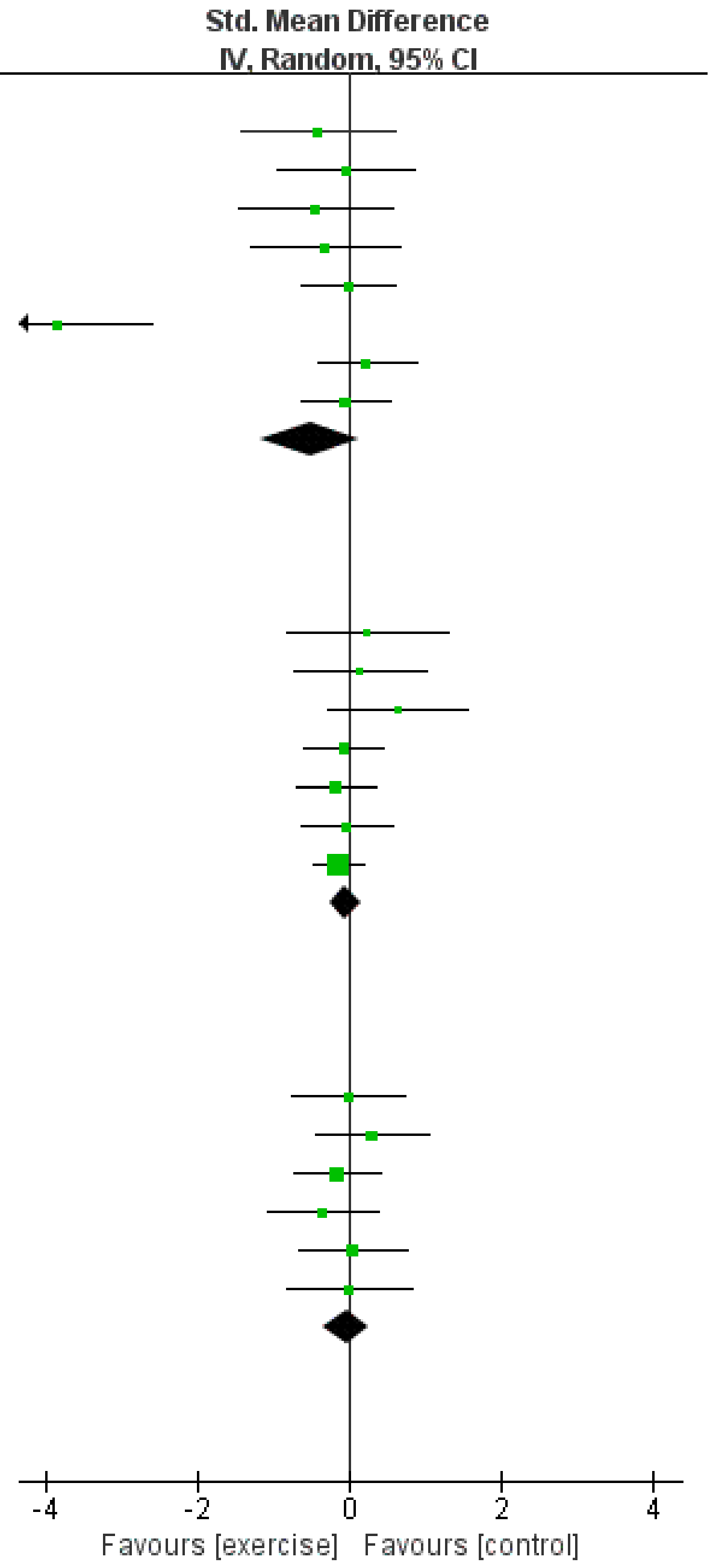

Supplementary Figure 47 Forest plot of subanalyses of Triglycerides

Study or Subgroup	Experimental Mean	Experimental SD	Experimental Total	Control Mean	Control SD	Control Total	Weight	Std. Mean Difference IV, Random, 95% CI
**3.20.1 ≤ 2months**								
Chee Huei(2017)	-0.2	0.78	34	-0.09	1.05	85	11.0%	-0.11 [-0.51, 0.29]
Elliott(2002)	-0.44	1.37	8	0.34	1.11	7	2.1%	-0.58 [-1.63, 0.46]
Kranen(2023)	-0.06	0.29	10	0.18	0.3	9	2.5%	-0.78 [-1.72, 0.16]
Nikseresht(2016)a	-0.29	0.484	10	-0.32	0.91	6	2.2%	0.04 [-0.97, 1.05]
Nikseresht(2016)b	-0.23	0.981	12	-0.32	0.91	6	2.3%	0.09 [-0.89, 1.07]
Prasertsri(2019)	-0.12	0.476	18	-0.01	0.82	21	5.2%	-0.16 [-0.79, 0.47]
Shorki(2021)	-0.07	0.05	15	0.01	0.06	15	3.3%	-1.41 [-2.22, -0.60]
Stergioulas(2006)	-0.53	1.01	21	-0.09	1.11	17	5.0%	-0.41 [-1.05, 0.24]
Teixeira(2023)	-0.47	1.64	19	-0.23	1.23	17	4.8%	-0.16 [-0.82, 0.49]
Theodorou(2016)a	-0.09	0.91	41	0.25	1.26	15	5.7%	-0.33 [-0.93, 0.26]
Thompson(2010)	-0.15	0.7	21	-0.29	0.71	20	5.4%	0.19 [-0.42, 0.81]
**Subtotal (95% CI)**			**209**			**218**	**49.5%**	**-0.28 [-0.52, -0.04]**
Heterogeneity: Tau² = 0.04; Chi² = 13.15, df = 10 (P = 0.22); I² = 24%								
Test for overall effect: Z = 2.26 (P = 0.02)								
**3.20.2 2-6months**								
Hansen(2024)	-0.08	1.46	8	0.06	0.72	6	2.0%	-0.11 [-1.17, 0.95]
Martins(2010)a	-0.13	0.51	18	-0.15	0.34	7	2.9%	0.04 [-0.83, 0.91]
Martins(2010)b	0.16	0.93	14	-0.15	0.34	7	2.6%	0.38 [-0.54, 1.29]
Nordén(2024)	0	0.87	28	0	0.8	30	7.3%	0.00 [-0.52, 0.52]
Theodorou(2016)b	-0.1	0.99	15	0.03	1.29	15	4.1%	-0.11 [-0.83, 0.61]
Zheng(2019)	0.05	0.97	79	-0.1	1.21	69	14.7%	0.14 [-0.19, 0.46]
**Subtotal (95% CI)**			**162**			**134**	**33.7%**	**0.08 [-0.15, 0.31]**
Heterogeneity: Tau² = 0.00; Chi² = 1.01, df = 5 (P = 0.96); I² = 0%								
Test for overall effect: Z = 0.68 (P = 0.50)								
**3.20.3 >6months**								
Brekke(2014)	0.06	0.25	15	0.15	0.52	13	3.8%	-0.22 [-0.96, 0.53]
Brobakken(2024)	0.55	1.52	14	0.32	1.16	14	3.9%	0.17 [-0.58, 0.91]
Gjellesvik(2020)	0.17	0.7	22	-0.21	1.27	26	6.1%	0.36 [-0.22, 0.93]
Haganes(2025)	0.1	0.61	12	-0.1	0.53	10	3.0%	0.33 [-0.51, 1.18]
**Subtotal (95% CI)**			**63**			**63**	**16.9%**	**0.18 [-0.17, 0.53]**
Heterogeneity: Tau² = 0.00; Chi² = 1.60, df = 3 (P = 0.66); I² = 0%								
Test for overall effect: Z = 1.01 (P = 0.31)								
**Total (95% CI)**			**434**			**415**	**100.0%**	**-0.08 [-0.23, 0.08]**
Heterogeneity: Tau² = 0.01; Chi² = 22.58, df = 20 (P = 0.31); I² = 11%								
Test for overall effect: Z = 0.99 (P = 0.32)								
Test for subgroup differences: Chi² = 6.30, df = 2 (P = 0.04), I² = 68.3%								

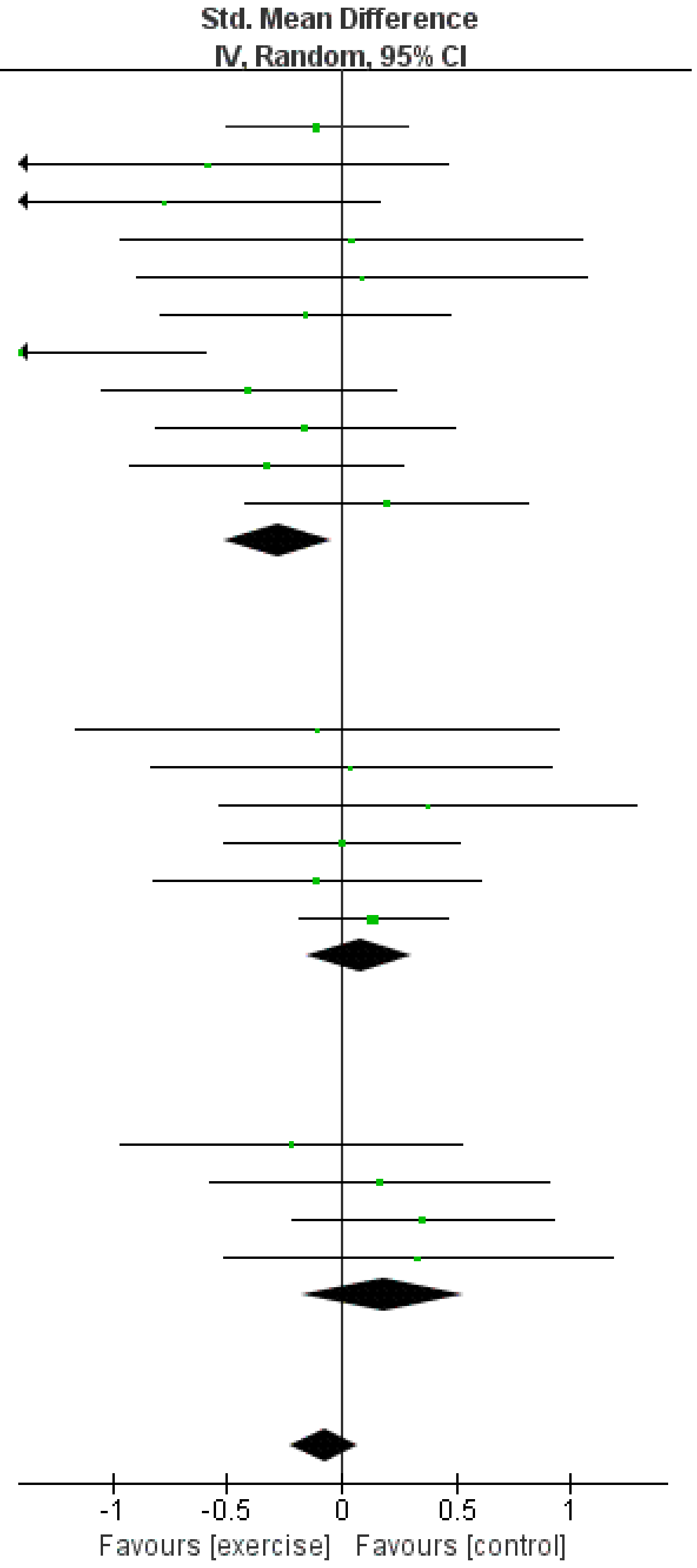

Supplementary Figure 48 Forest plot of subanalyses of HOMA-IR

Study or Subgroup	Experimental Mean	Experimental SD	Experimental Total	Control Mean	Control SD	Control Total	Weight	Std. Mean Difference IV, Random, 95% CI
**3.23.1 ≤ 2months**								
Donyaei(2024)	-0.92	0.6	17	0.07	1.22	17	18.9%	-1.01 [-1.72, -0.29]
Moghadasi(2012)	-1.36	0.56	8	0.12	0.69	8	9.7%	-2.23 [-3.55, -0.90]
Nuri(2016)	-0.17	0.35	15	-0.06	0.4	15	18.9%	-0.28 [-1.00, 0.44]
Thompson(2010)	0.3	4.21	21	1.3	5.89	20	21.2%	-0.19 [-0.81, 0.42]
**Subtotal (95% CI)**			**61**			**60**	**68.8%**	**-0.77 [-1.47, -0.07]**
Heterogeneity: Tau² = 0.34; Chi² = 9.46, df = 3 (P = 0.02); I² = 68%								
Test for overall effect: Z = 2.14 (P = 0.03)								
**3.23.2 2-6months**								
Fatouros(2005)a	0.37	2.61	14	0.13	2.93	3	10.5%	0.09 [-1.16, 1.33]
Fatouros(2005)b	0.18	2.92	12	0.13	2.93	3	10.3%	0.02 [-1.25, 1.28]
Fatouros(2005)c	-0.17	2.03	14	0.13	2.93	3	10.5%	-0.13 [-1.38, 1.12]
**Subtotal (95% CI)**			**40**			**9**	**31.2%**	**-0.01 [-0.73, 0.71]**
Heterogeneity: Tau² = 0.00; Chi² = 0.06, df = 2 (P = 0.97); I² = 0%								
Test for overall effect: Z = 0.03 (P = 0.98)								
**Total (95% CI)**			**101**			**69**	**100.0%**	**-0.50 [-1.00, -0.01]**
Heterogeneity: Tau² = 0.20; Chi² = 11.56, df = 6 (P = 0.07); I² = 48%								
Test for overall effect: Z = 2.00 (P = 0.05)								
Test for subgroup differences: Chi² = 2.17, df = 1 (P = 0.14), I² = 53.9%								

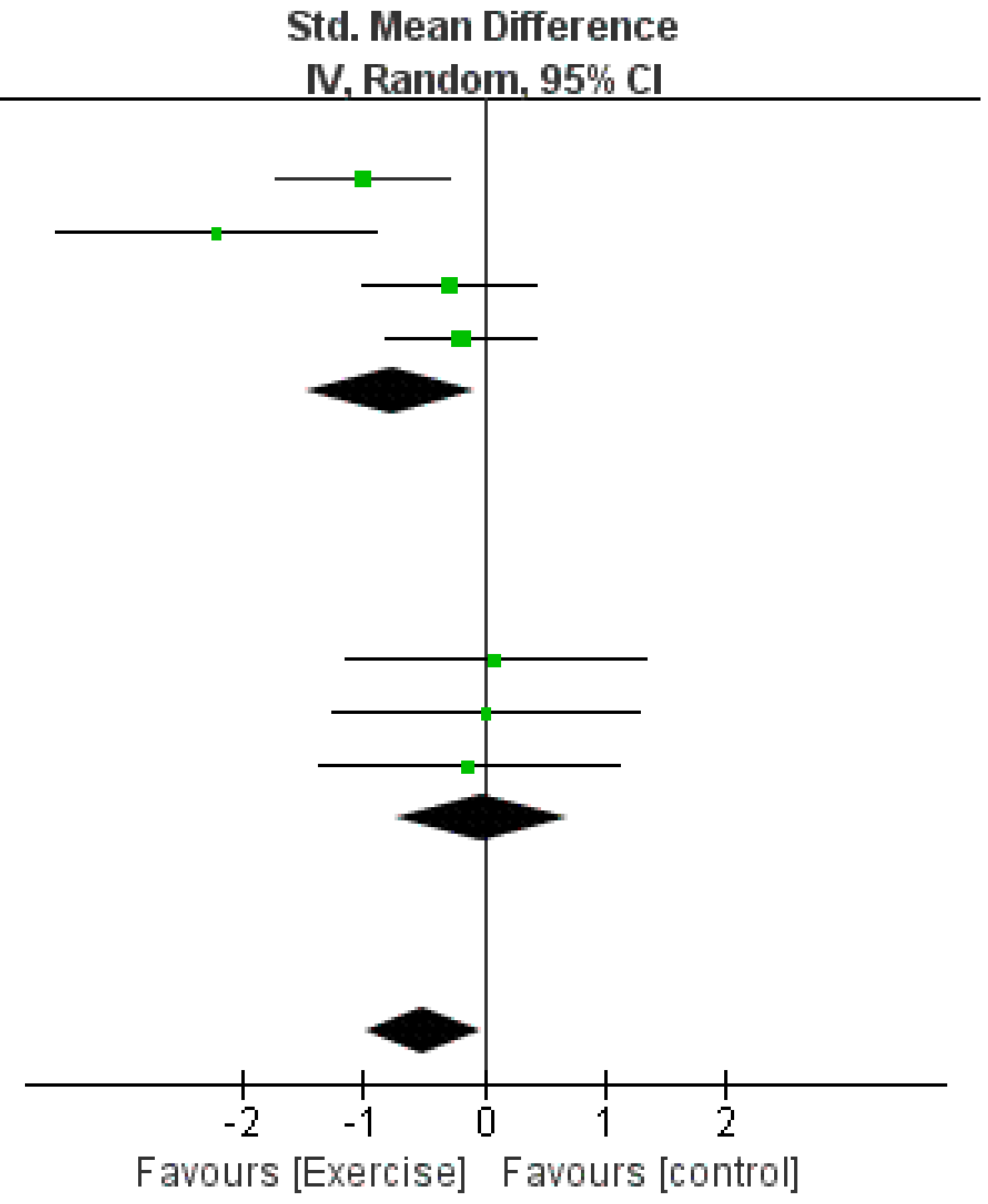

Supplementary Figure 49 Forest plot of subanalyses of Fasting blood glucose

Study or Subgroup	exercise Mean	exercise SD	exercise Total	Control Mean	Control SD	Control Total	Weight	Std. Mean Difference IV, Random, 95% CI
**3.21.1 ≤ 2months**								
Chee Huei(2017)	-0.15	0.89	34	-0.04	0.75	85	9.6%	-0.14 [-0.54, 0.26]
Donyaei(2024)	-0.63	0.58	17	-0.02	0.77	17	6.5%	-0.87 [-1.58, -0.17]
Kranen(2023)	-0.05	0.61	10	0.34	0.39	9	4.7%	-0.72 [-1.66, 0.22]
Moghadasi(2012)	-0.9	0.34	8	0.1	3.97	8	4.4%	-0.34 [-1.32, 0.65]
Nikseresht(2016)a	-1.2	0.76	10	-0.03	0.78	6	3.5%	-1.44 [-2.61, -0.28]
Nikseresht(2016)b	-2.13	0.5	12	-0.03	0.78	6	2.2%	-3.32 [-4.90, -1.75]
Nuri(2016)	-0.12	0.35	15	-0.12	0.38	15	6.4%	0.00 [-0.72, 0.72]
Prasertsri(2019)	-0.15	0.71	18	0.16	0.64	21	7.1%	-0.45 [-1.09, 0.19]
Teixeira(2023)	-0.17	0.39	19	-0.2	0.76	17	6.9%	0.05 [-0.61, 0.70]
Thompson(2010)	0.08	0.6	21	0.09	0.41	20	7.4%	-0.02 [-0.63, 0.59]
**Subtotal (95% CI)**			**164**			**204**	**58.9%**	**-0.50 [-0.89, -0.11]**
Heterogeneity: Tau² = 0.23; Chi² = 24.49, df = 9 (P = 0.004); I² = 63%								
Test for overall effect: Z = 2.54 (P = 0.01)								
**3.21.2 2-6months**								
Fatouros(2005)a	0.8	11.23	14	-0.4	13.65	3	3.2%	0.10 [-1.15, 1.35]
Fatouros(2005)b	-0.1	1.53	12	-0.4	13.65	3	3.1%	0.05 [-1.21, 1.32]
Fatouros(2005)c	0	9.23	14	-0.4	13.65	3	3.2%	0.04 [-1.21, 1.29]
Hansen(2024)	-0.28	1.26	8	-0.18	0.6	6	4.0%	-0.09 [-1.15, 0.97]
Zheng(2019)	-0.04	0.97	79	-0.02	1.71	69	10.5%	-0.01 [-0.34, 0.31]
**Subtotal (95% CI)**			**127**			**84**	**24.0%**	**-0.01 [-0.29, 0.28]**
Heterogeneity: Tau² = 0.00; Chi² = 0.07, df = 4 (P = 1.00); I² = 0%								
Test for overall effect: Z = 0.06 (P = 0.96)								
**3.21.3 >6months**								
Brekke(2014)	0.11	0.33	15	0.22	0.55	13	6.1%	-0.24 [-0.99, 0.51]
Brobakken(2024)	1.43	2.38	14	0.48	0.82	14	6.1%	0.52 [-0.24, 1.27]
Haganes(2025)	0	0.35	12	0.4	0.36	10	4.9%	-1.09 [-2.00, -0.17]
**Subtotal (95% CI)**			**41**			**37**	**17.1%**	**-0.24 [-1.11, 0.63]**
Heterogeneity: Tau² = 0.43; Chi² = 7.10, df = 2 (P = 0.03); I² = 72%								
Test for overall effect: Z = 0.54 (P = 0.59)								
**Total (95% CI)**			**332**			**325**	**100.0%**	**-0.31 [-0.57, -0.05]**
Heterogeneity: Tau² = 0.14; Chi² = 35.07, df = 17 (P = 0.006); I² = 52%								
Test for overall effect: Z = 2.34 (P = 0.02)								
Test for subgroup differences: Chi² = 4.07, df = 2 (P = 0.13), I² = 50.9%								

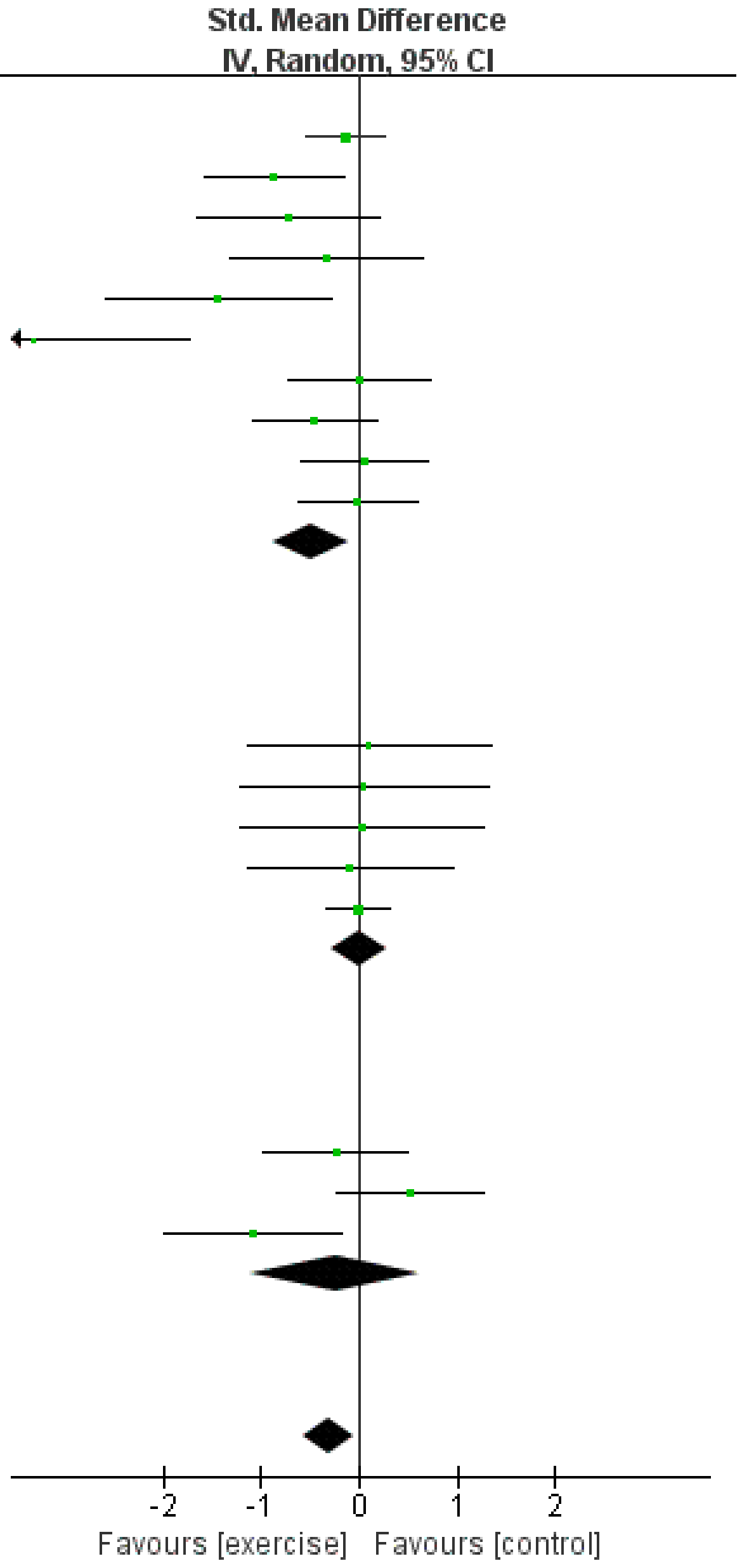

Supplementary Figure 50 Forest plot of subanalyses of Fasting insulin

Study or Subgroup	Exercise Mean	Exercise SD	Exercise Total	Control Mean	Control SD	Control Total	Weight	Std. Mean Difference IV, Random, 95% CI
**3.25.1 ≤2months**								
Donyaei(2024)	-0.86	1.11	17	-0.13	1.33	17	18.4%	-0.58 [-1.27, 0.11]
Kranen(2023)	1.9	4.03	10	3.28	4.22	9	11.3%	-0.32 [-1.23, 0.59]
Moghadasi(2012)	-4.12	1.74	8	0.26	2.62	8	6.5%	-1.86 [-3.09, -0.63]
Nuri(2016)	-0.6	1.08	15	-0.1	1.5	15	16.9%	-0.37 [-1.09, 0.35]
Thompson(2010)	0.6	18.47	21	4.5	24.97	20	22.2%	-0.17 [-0.79, 0.44]
**Subtotal (95% CI)**			**71**			**69**	**75.4%**	**-0.51 [-0.95, -0.08]**
Heterogeneity: Tau² = 0.08; Chi² = 6.09, df = 4 (P = 0.19); I² = 34%								
Test for overall effect: Z = 2.32 (P = 0.02)								
**3.25.2 2-6 months**								
Hansen(2024)	-5.46	19.88	8	-2	35.17	6	8.5%	-0.12 [-1.18, 0.94]
**Subtotal (95% CI)**			**8**			**6**	**8.5%**	**-0.12 [-1.18, 0.94]**
Heterogeneity: Not applicable								
Test for overall effect: Z = 0.22 (P = 0.83)								
**3.25.3 > 6months**								
Brekke(2014)	1.94	1.13	15	2.45	3.8	13	16.1%	-0.18 [-0.93, 0.56]
**Subtotal (95% CI)**			**15**			**13**	**16.1%**	**-0.18 [-0.93, 0.56]**
Heterogeneity: Not applicable								
Test for overall effect: Z = 0.48 (P = 0.63)								
**Total (95% CI)**			**94**			**88**	**100.0%**	**-0.41 [-0.73, -0.08]**
Heterogeneity: Tau² = 0.02; Chi² = 6.85, df = 6 (P = 0.34); I² = 12%								
Test for overall effect: Z = 2.47 (P = 0.01)								
Test for subgroup differences: Chi² = 0.86, df = 2 (P = 0.65), I² = 0%								

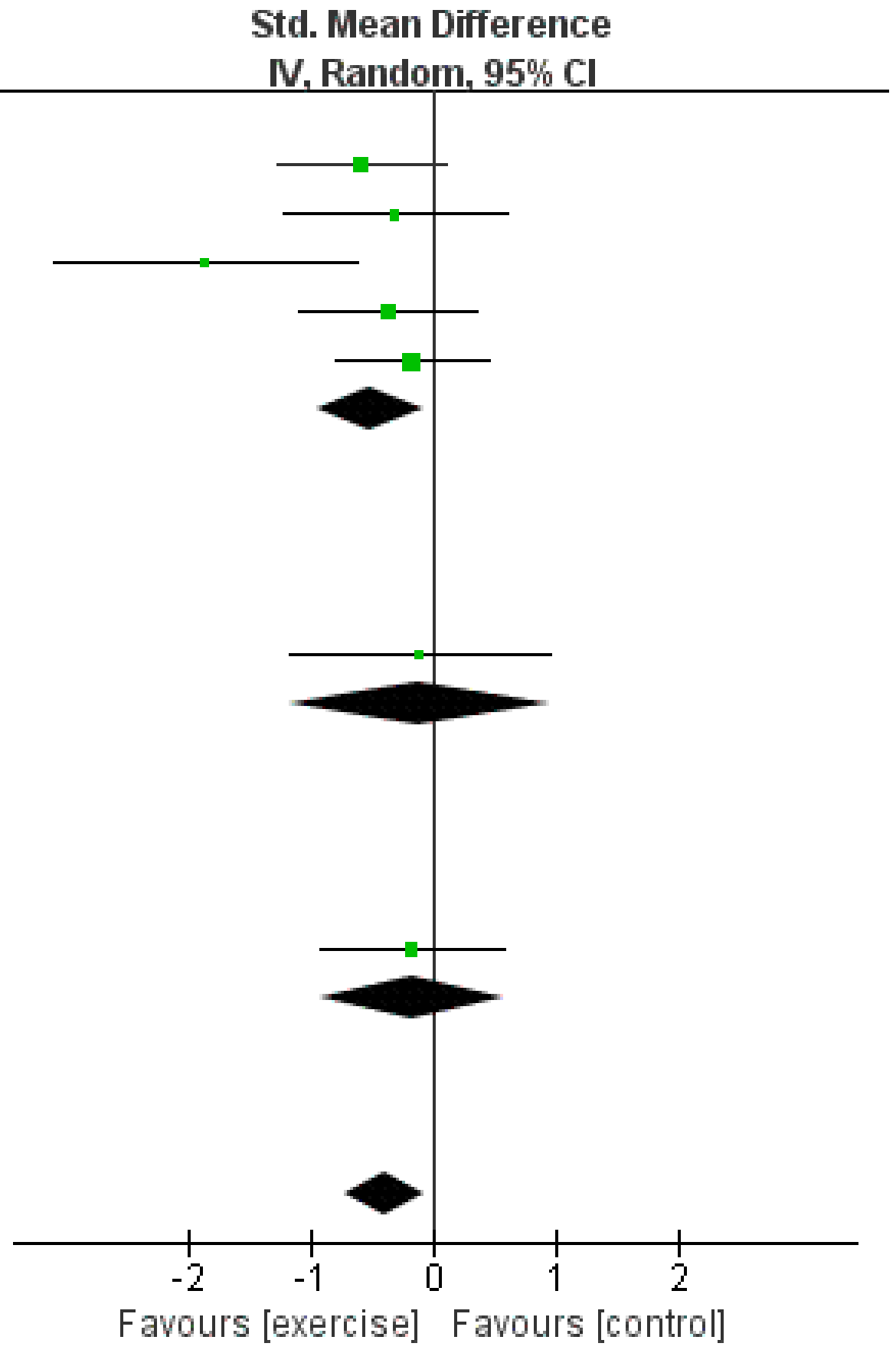

Supplementary Figure 51 Funnel plot of study bias on Body mass

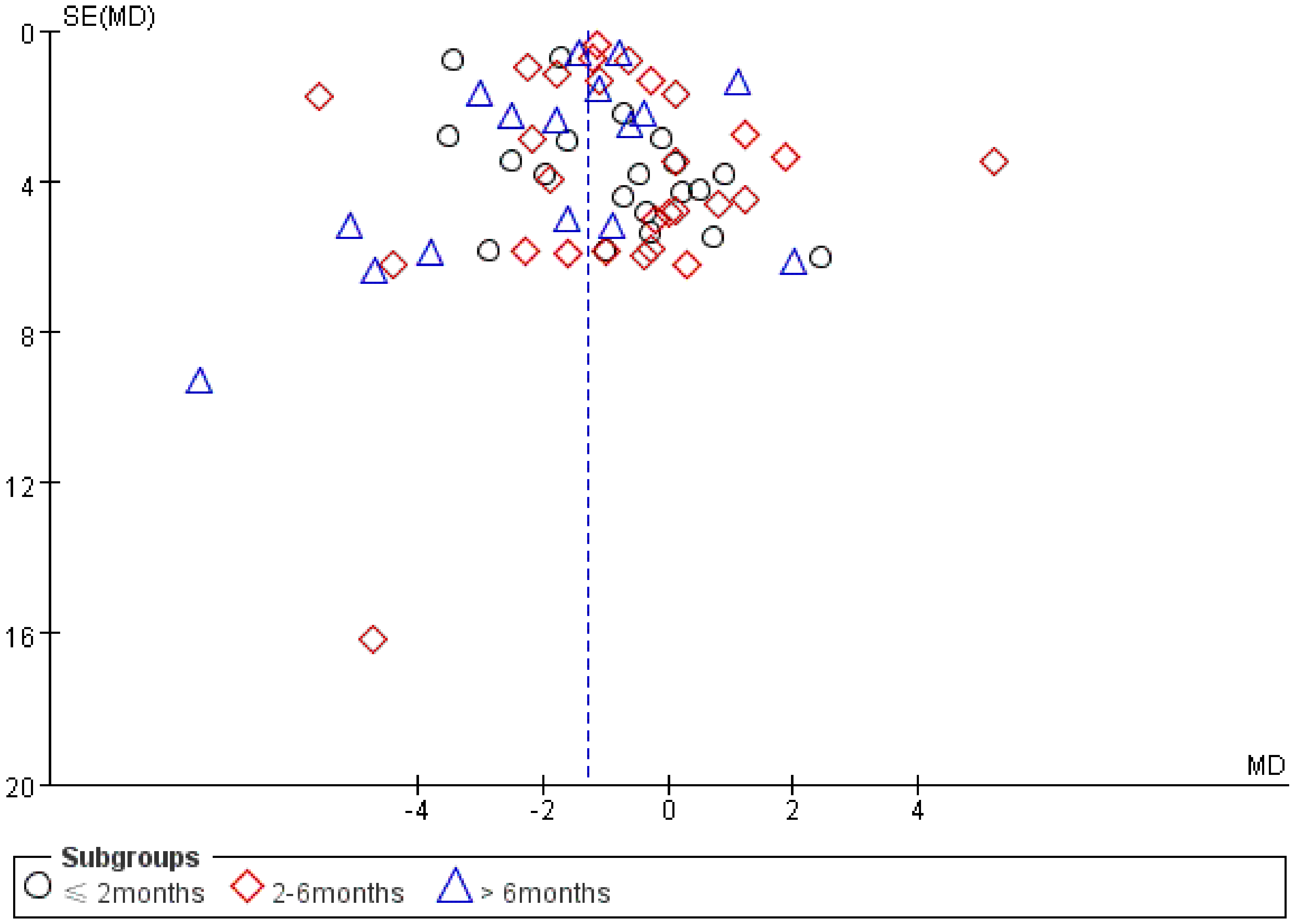

Supplementary Figure 52 Funnel plot of study bias on BMI

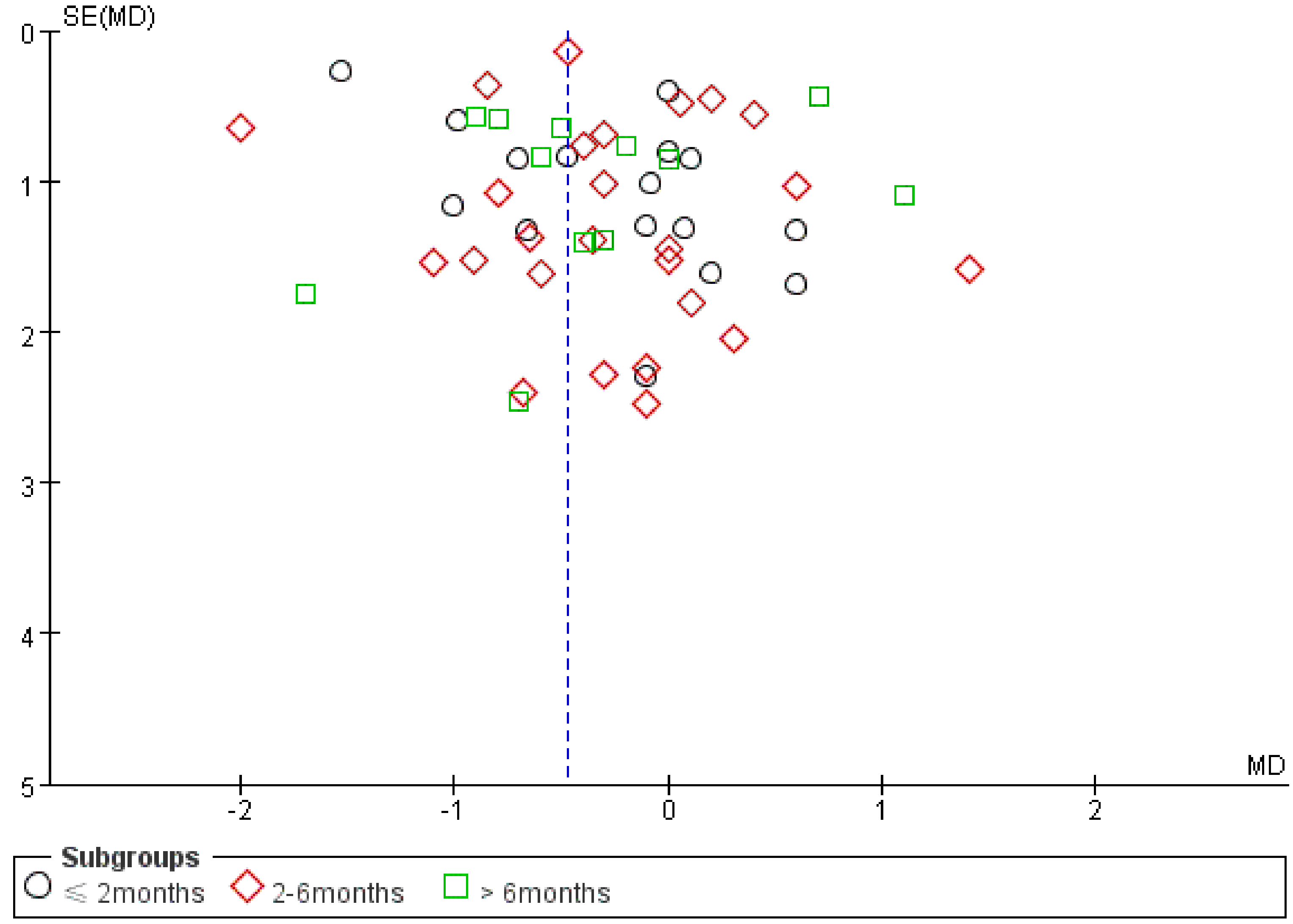

Supplementary Figure 53 Funnel plot of study bias on Body fat percentage

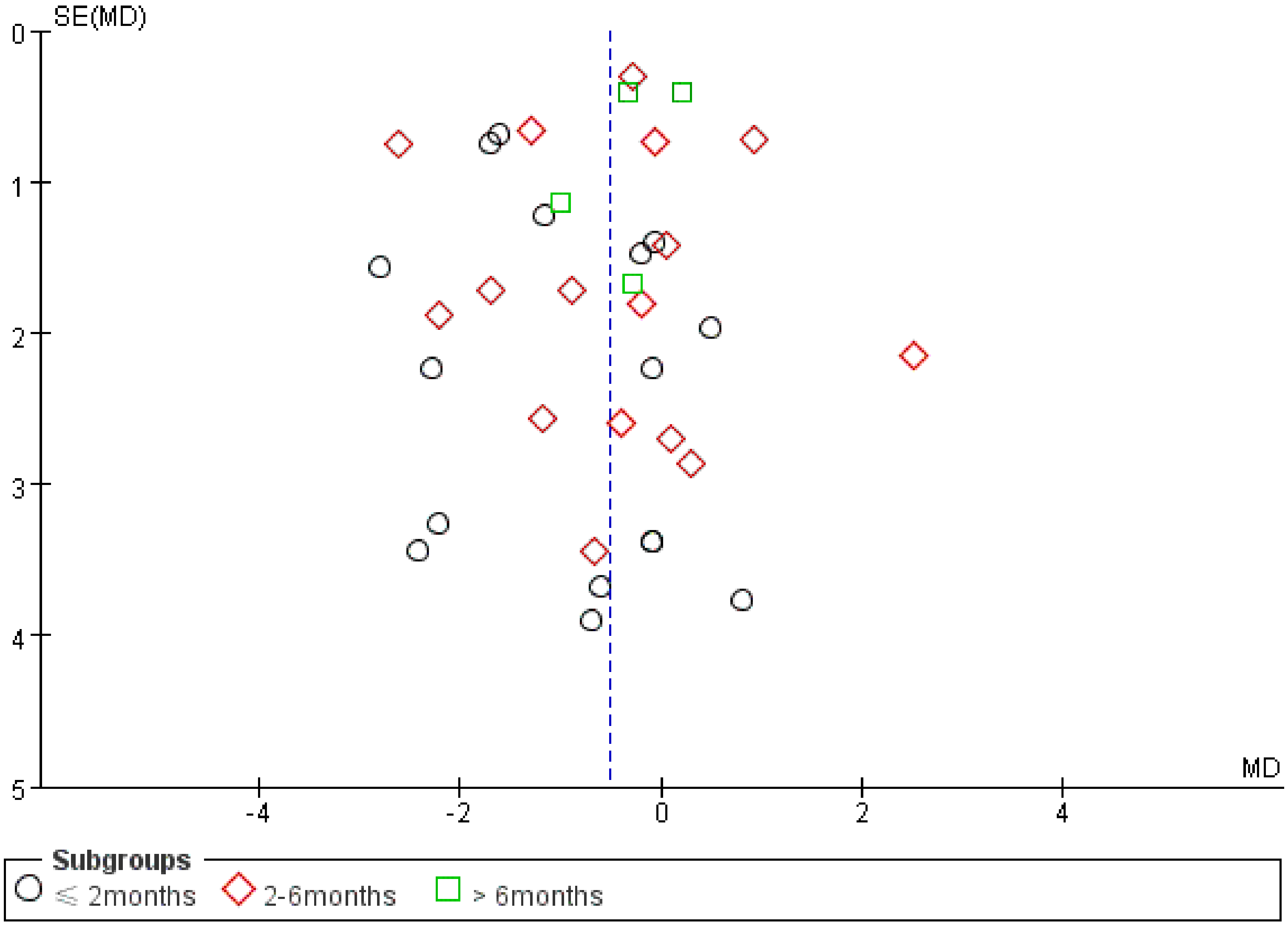

Supplementary Figure 54 Funnel plot of study bias on Fat mass

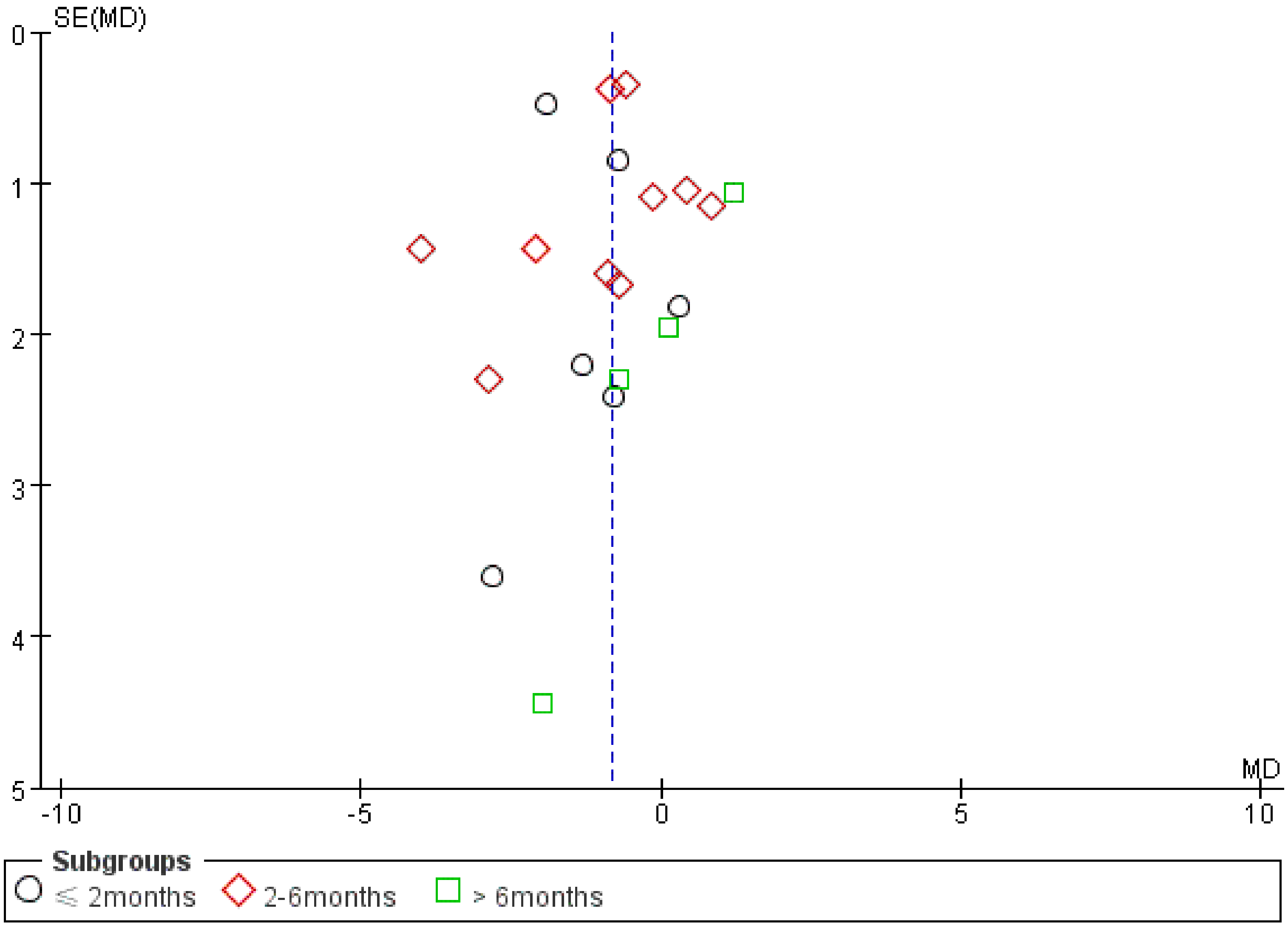

Supplementary Figure 55 Funnel plot of study bias on Muscle mass

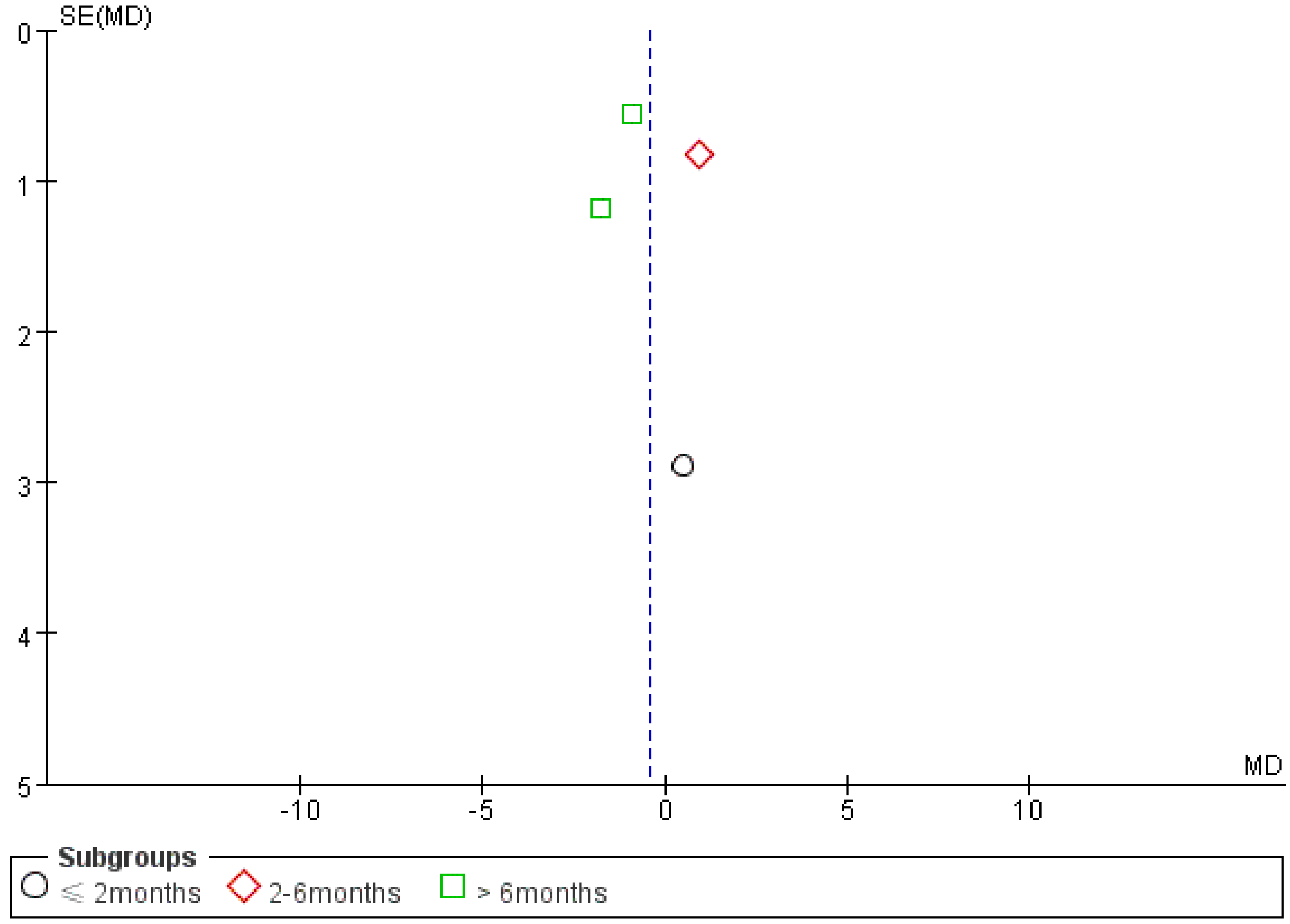

Supplementary Figure 56 Funnel plot of study bias on Waist circumference

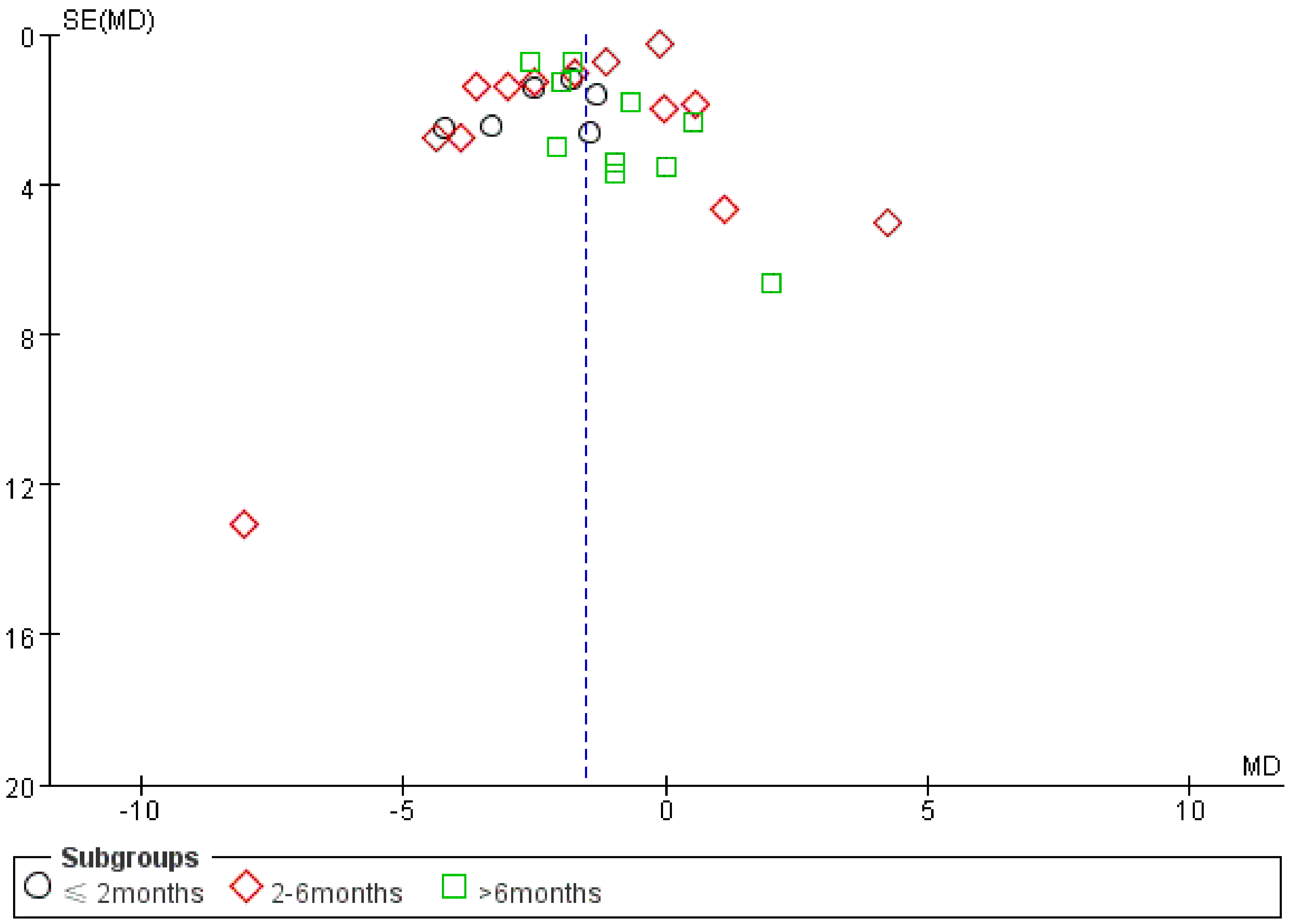

Supplementary Figure 57 Funnel plot of study bias on Waist-to-Hip Ratio

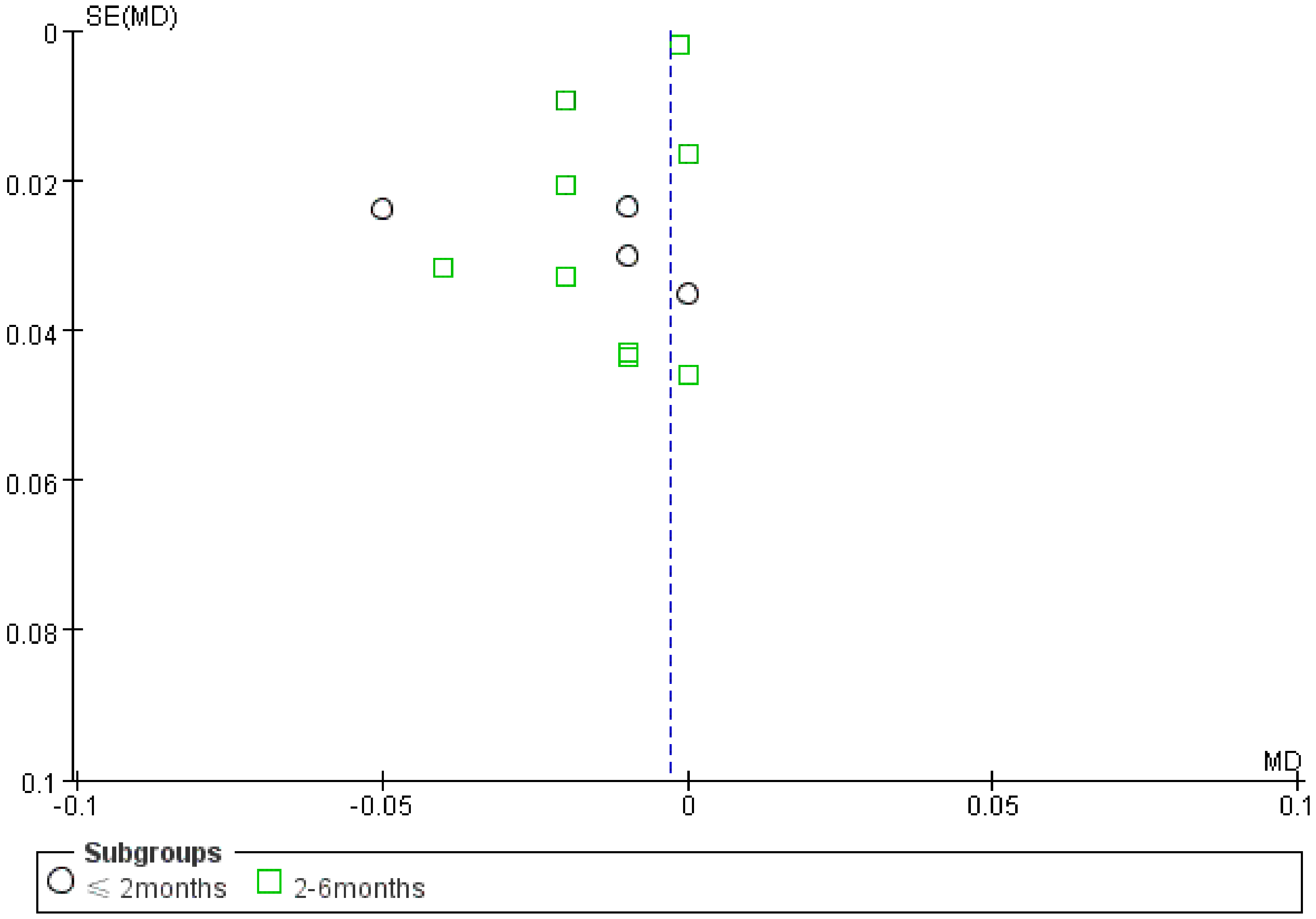

Supplementary Figure 58 Funnel plot of study bias on Fat-free mass

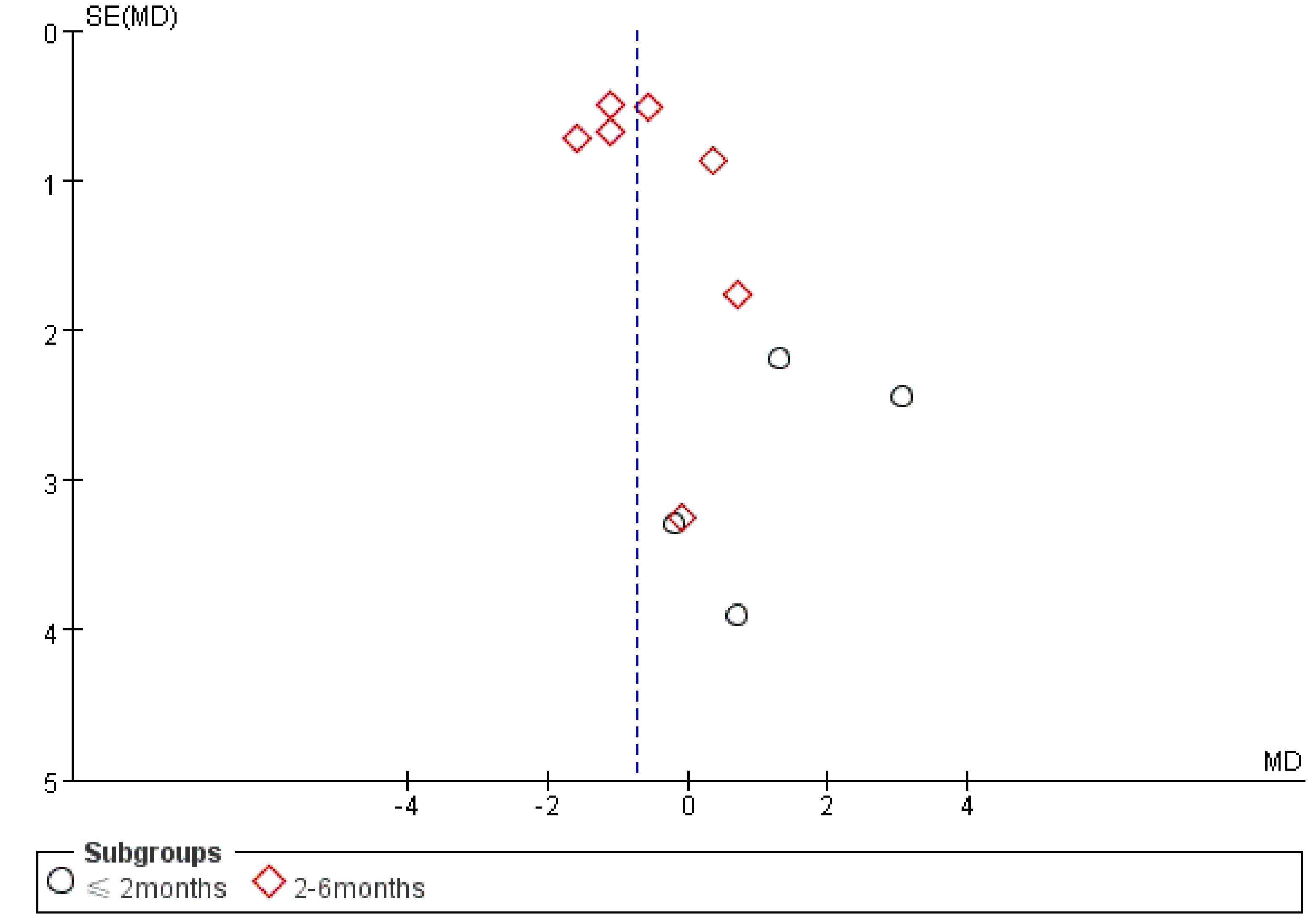

Supplementary Figure 59 Funnel plot of study bias on Lean body mass

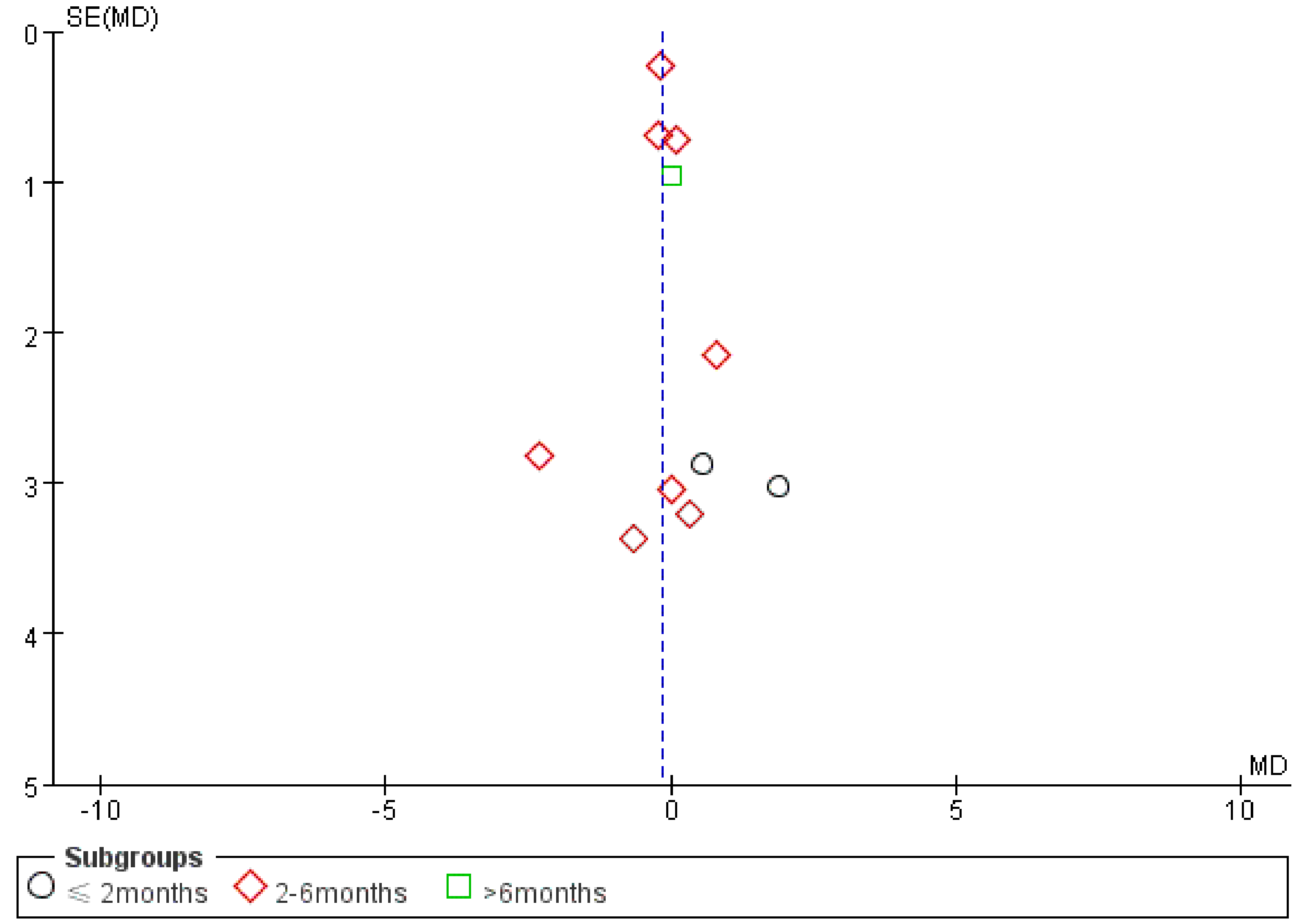

Supplementary Figure 60 Funnel plot of study bias on Skinfold

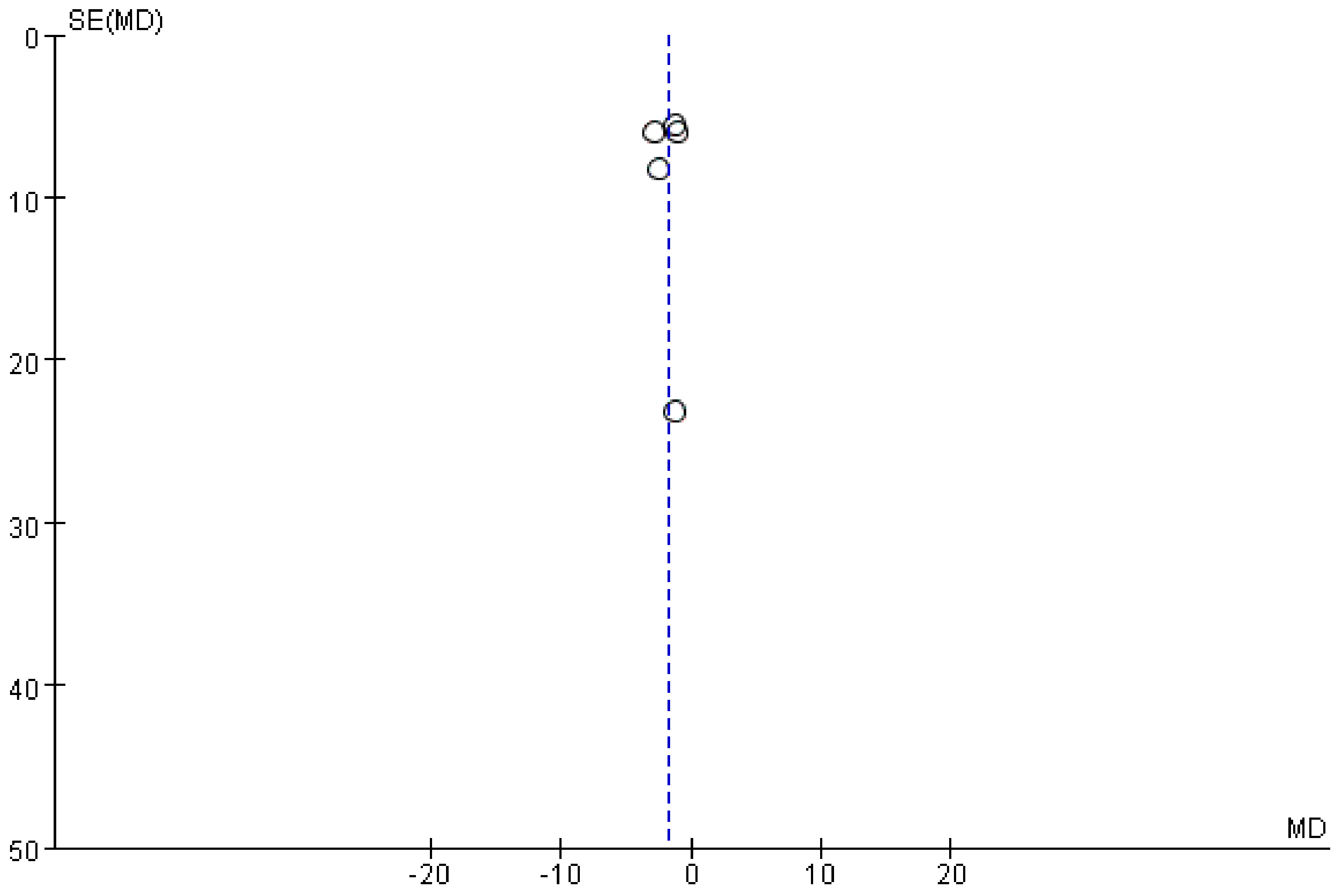

Supplementary Figure 61 Funnel plot of study bias on Systolic blood pressure

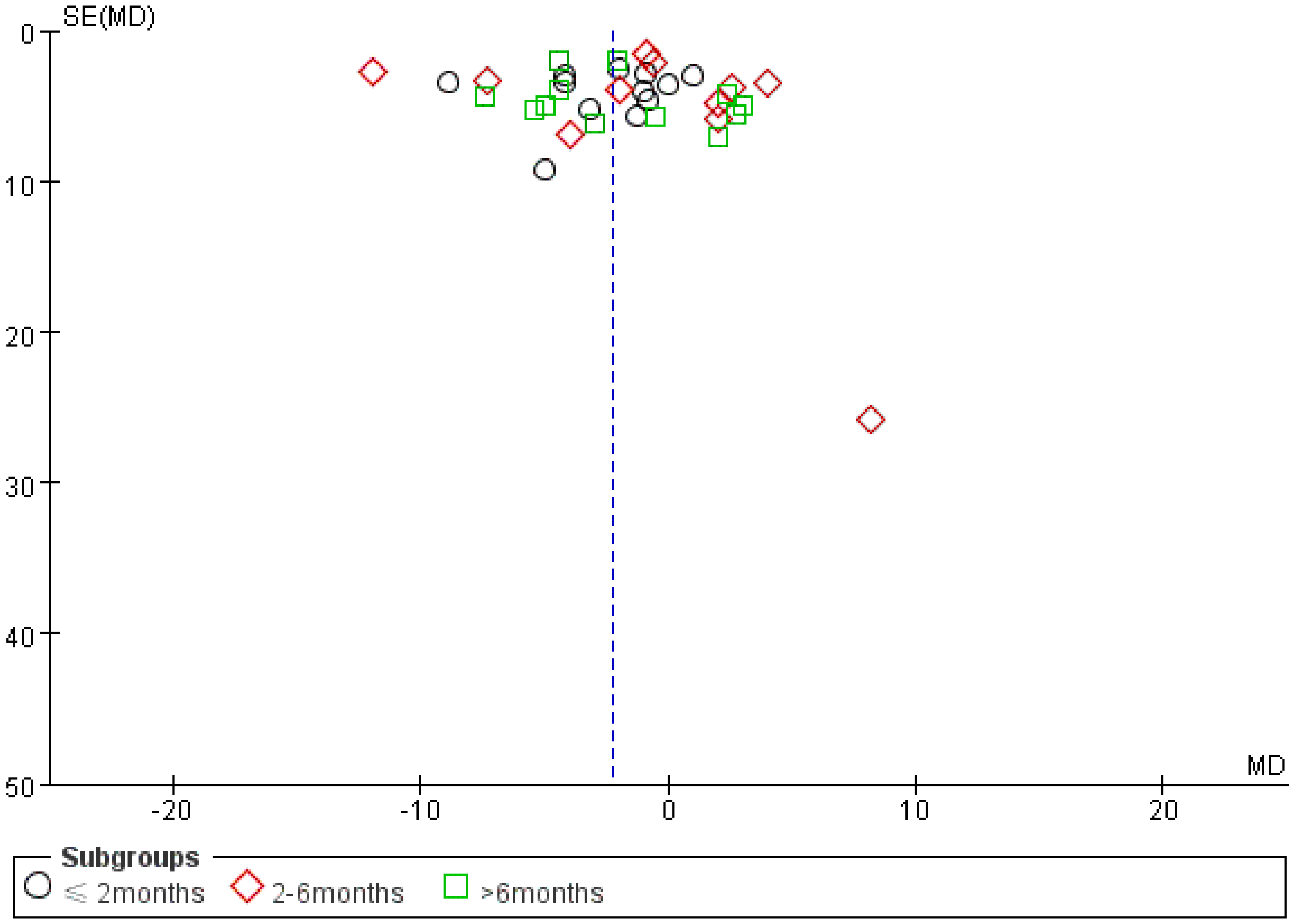

Supplementary Figure 62 Funnel plot of study bias on Diastolic blood pressure

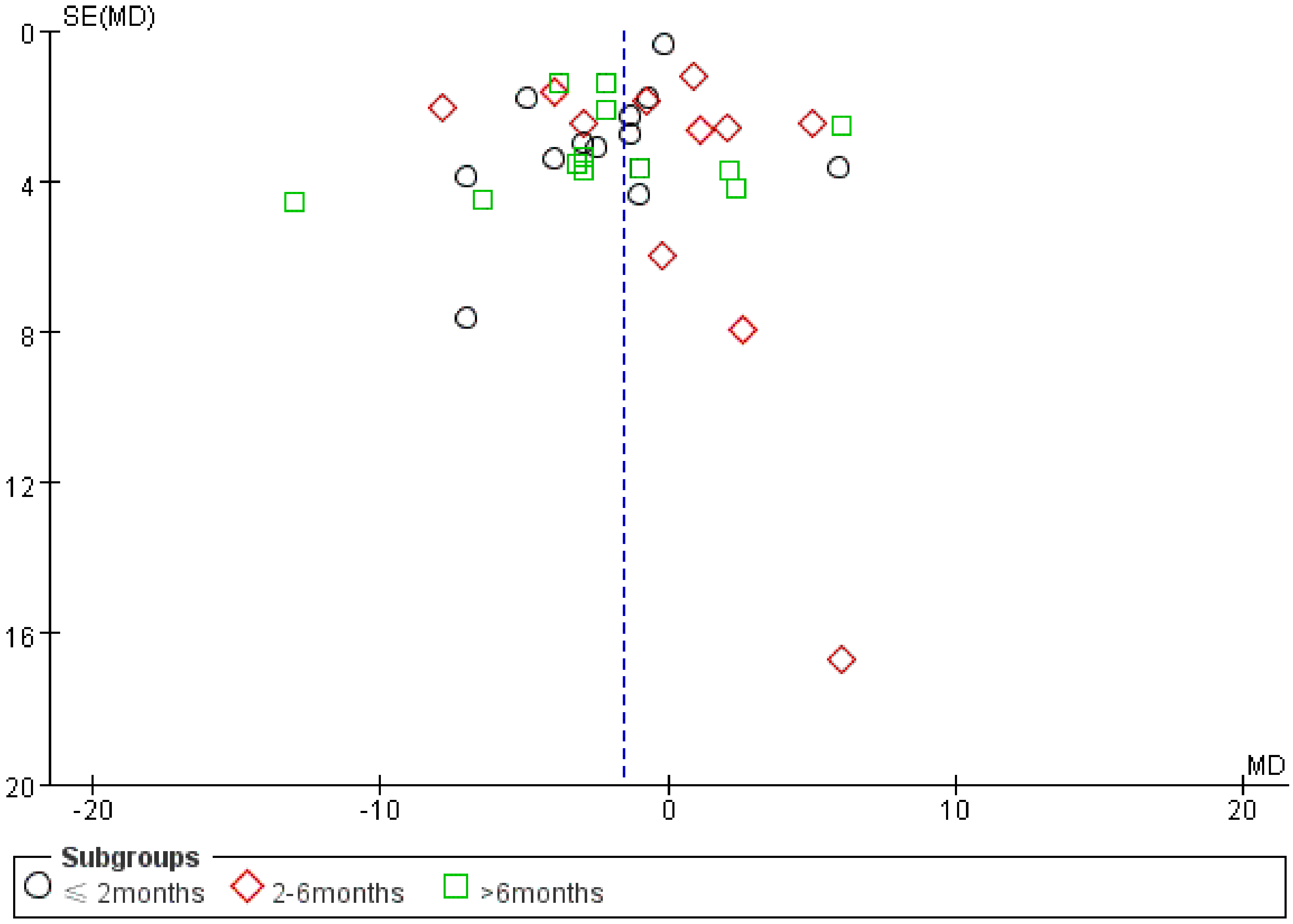

Supplementary Figure 63 Funnel plot of study bias on Mean arterial pressure

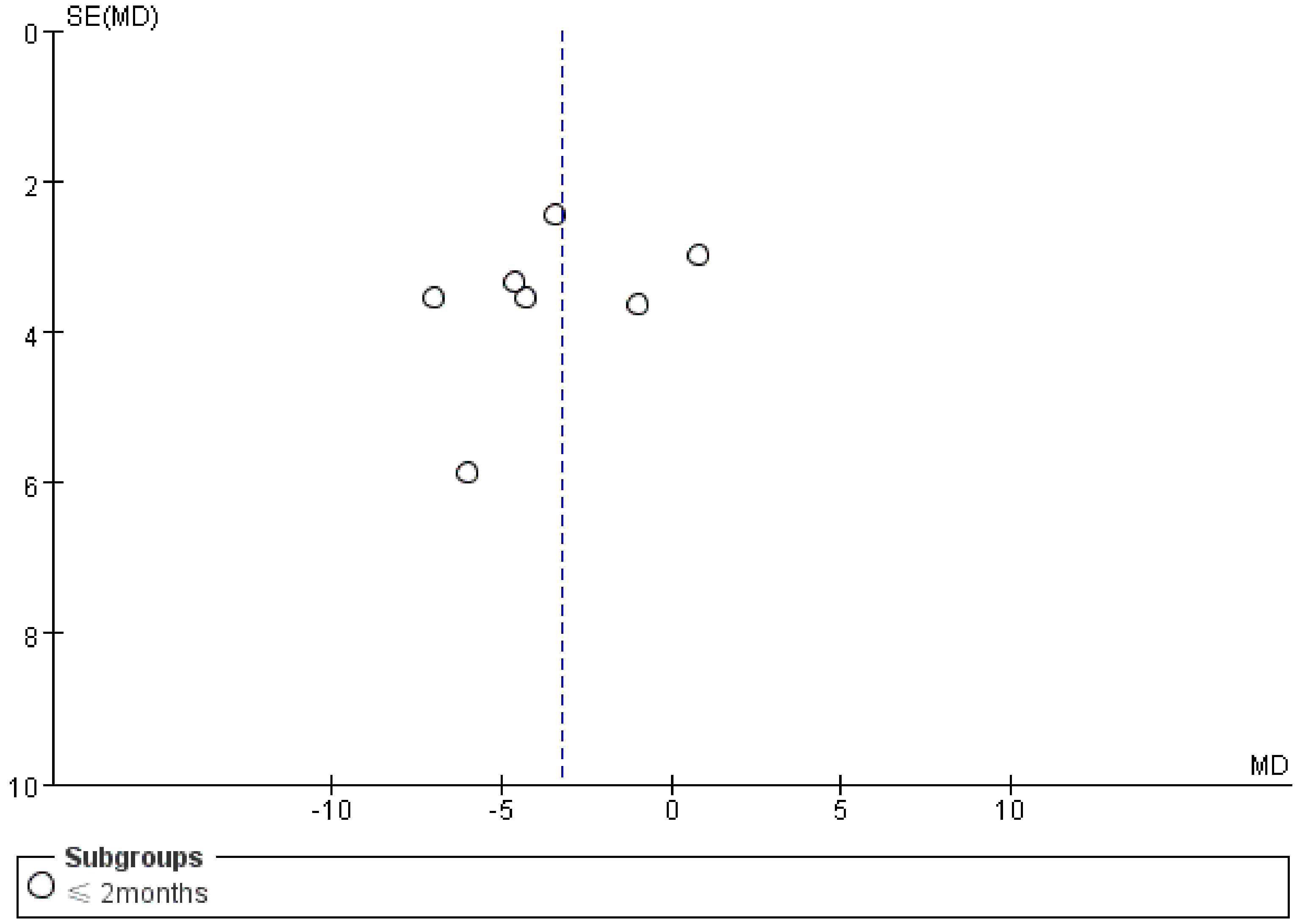

Supplementary Figure 64 Funnel plot of study bias on Resting heart rate

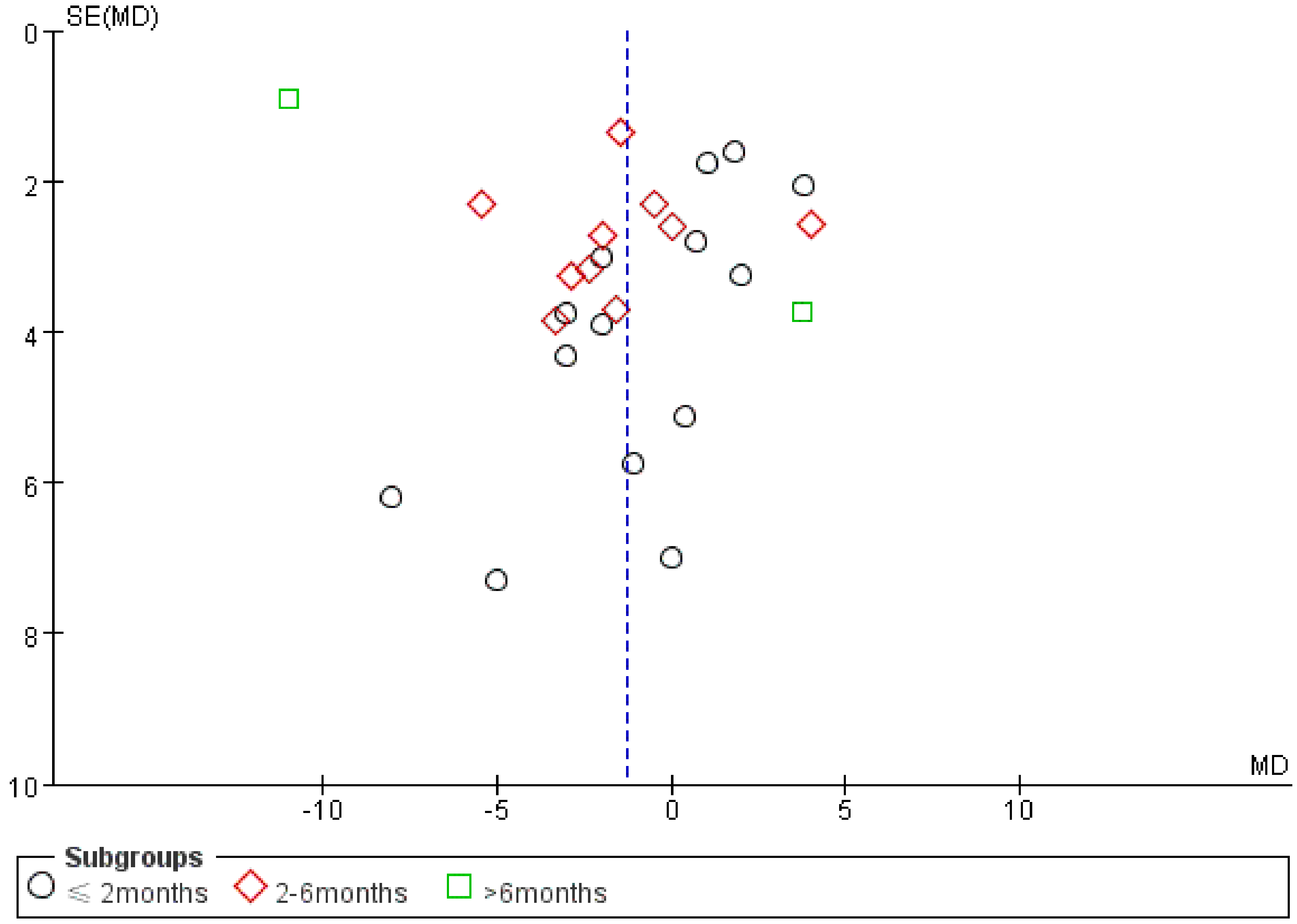

Supplementary Figure 65 Funnel plot of study bias on VO_{2peak}

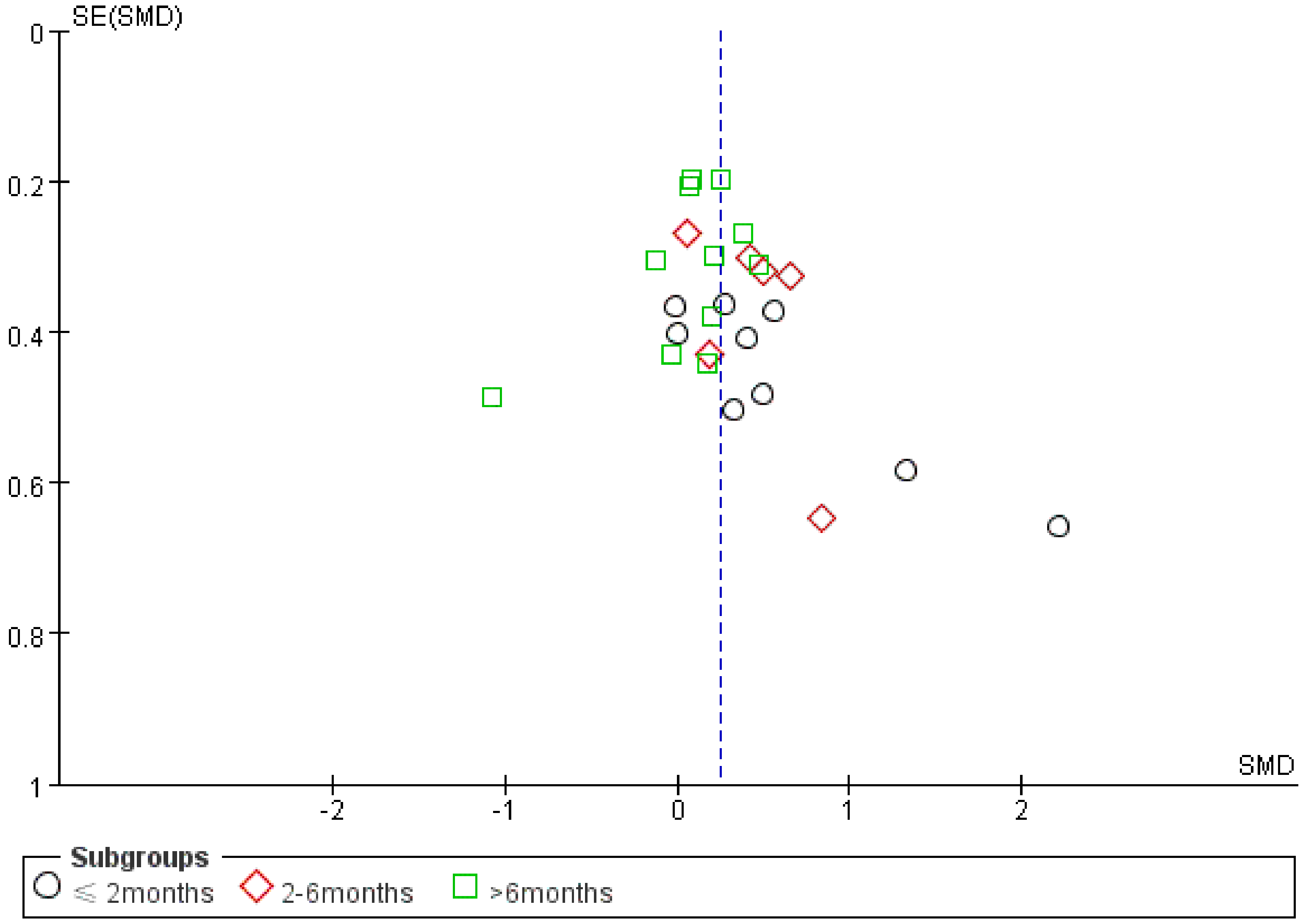

Supplementary Figure 66 Funnel plot of study bias on VO_{2max}

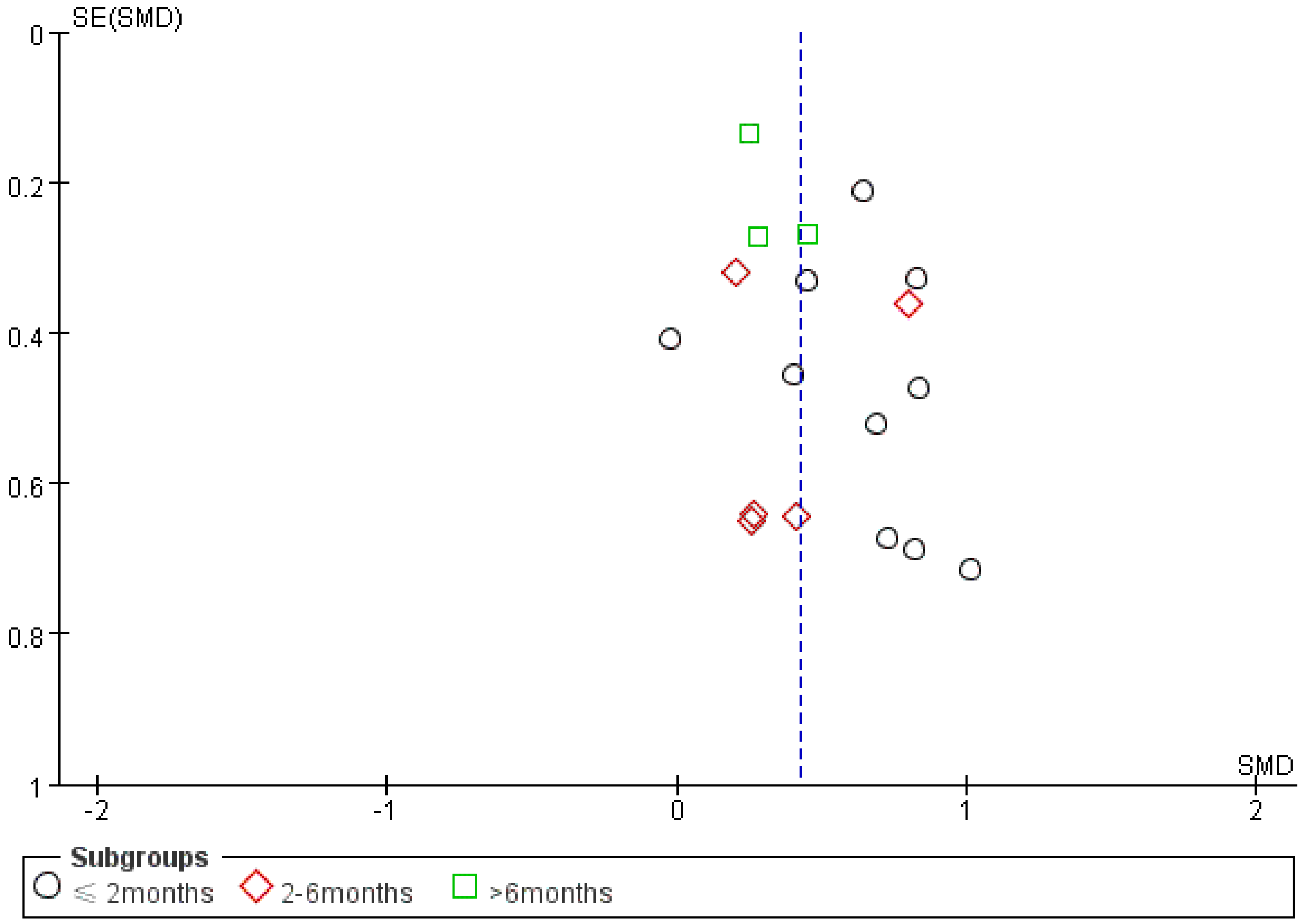

Supplementary Figure 67 Funnel plot of study bias on Total cholesterol

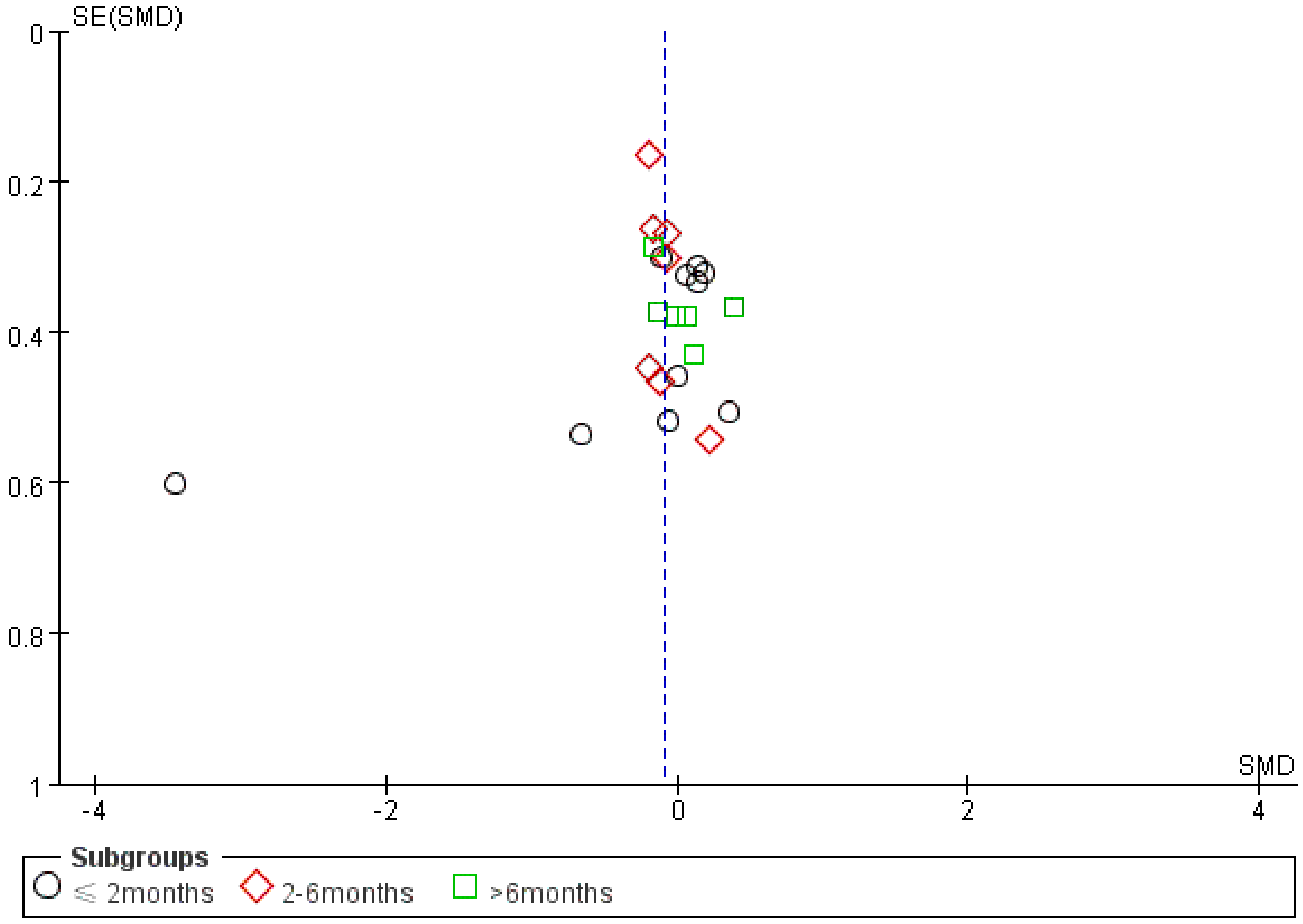

Supplementary Figure 68 Funnel plot of study bias on HDL-C

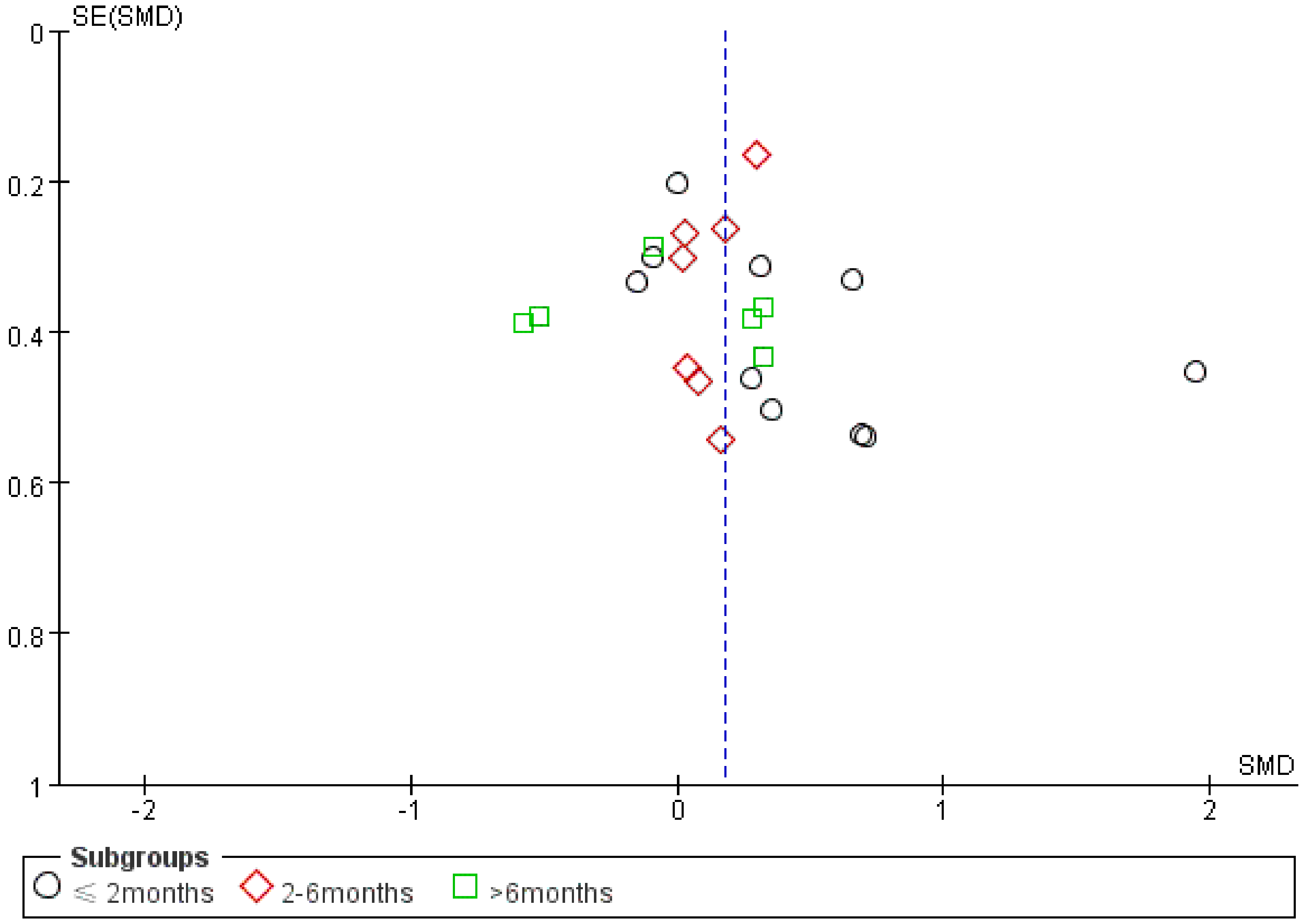

Supplementary Figure 69 Funnel plot of study bias on LDL-C

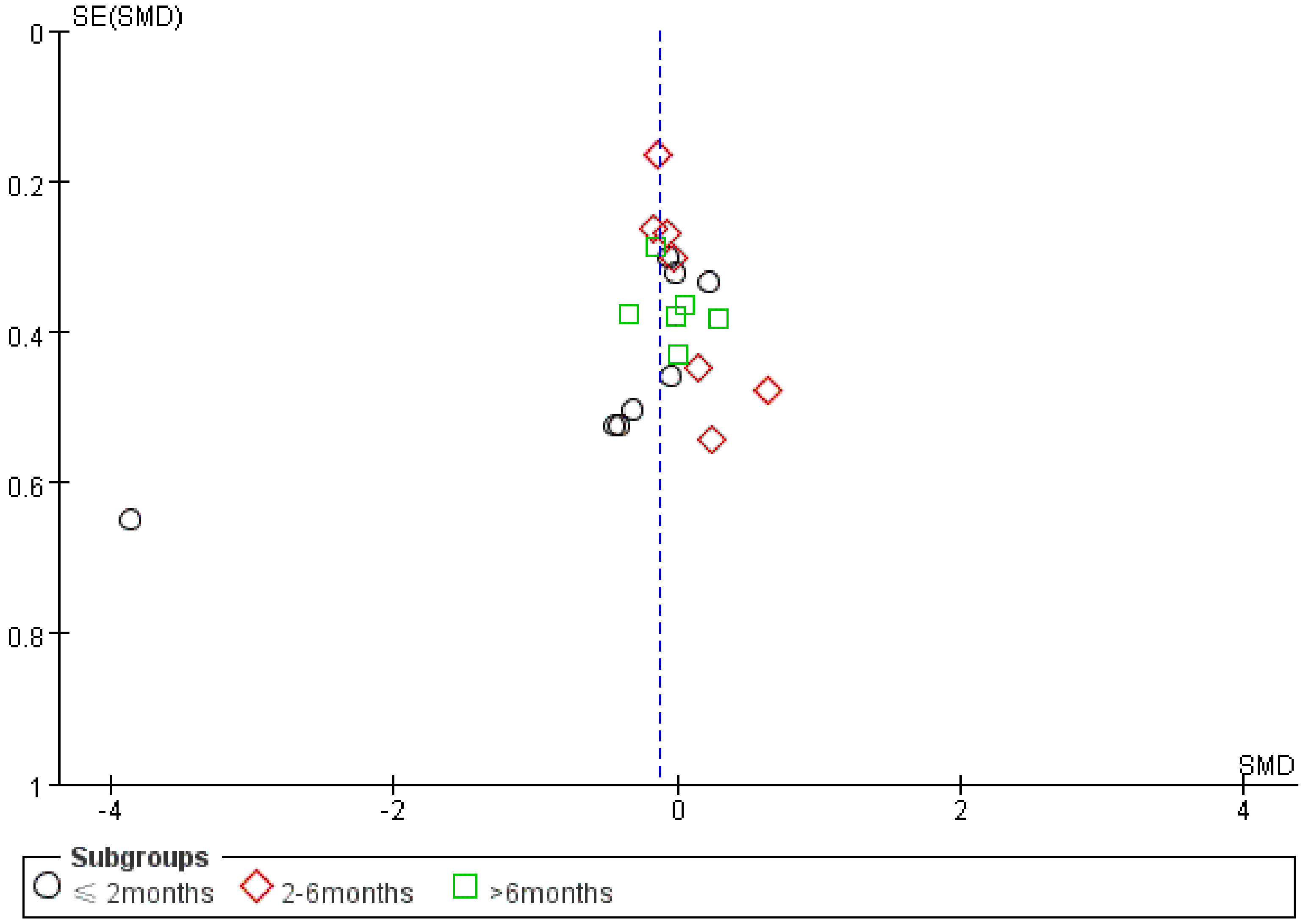

Supplementary Figure 70 Funnel plot of study bias on Triglycerides

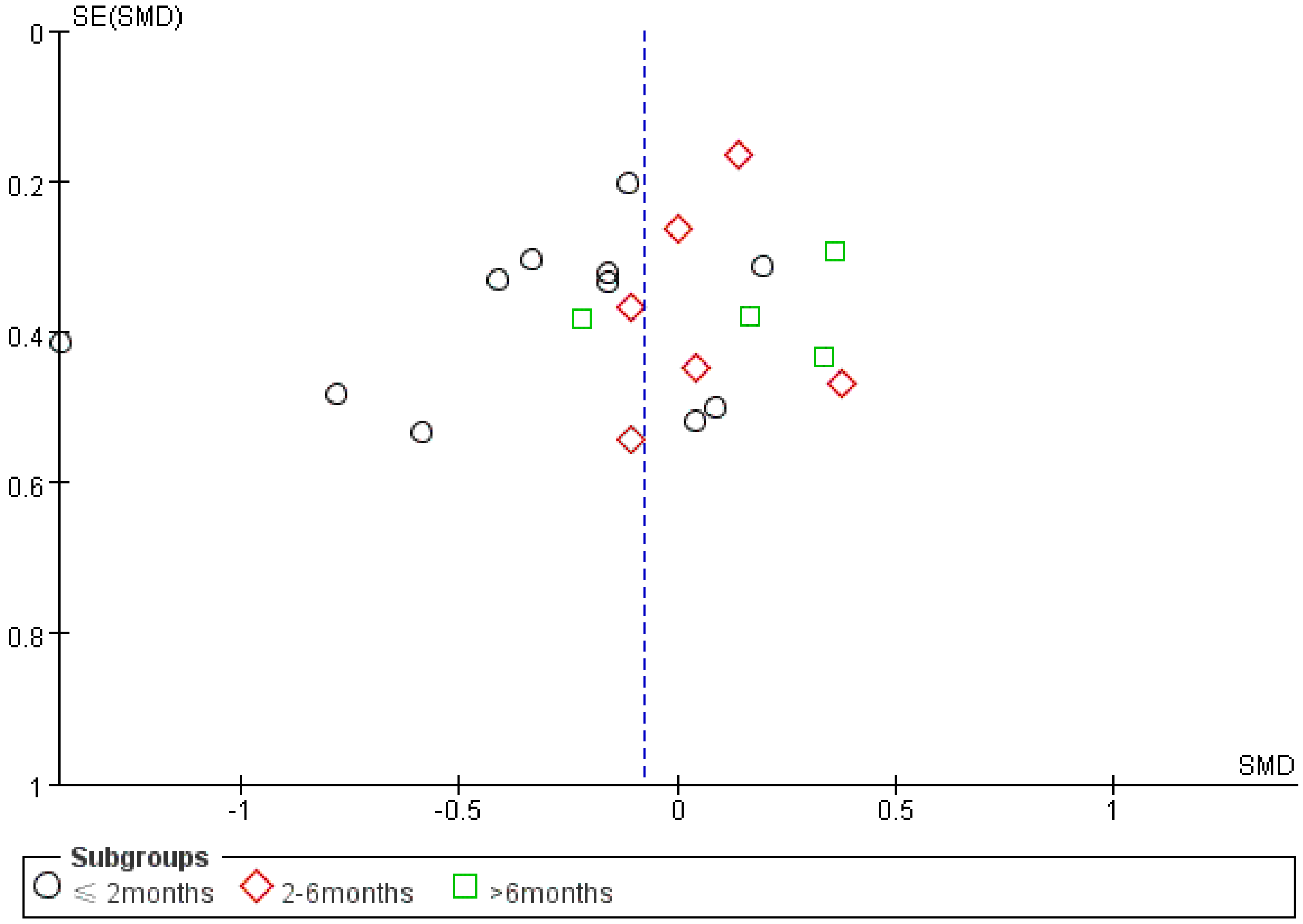

Supplementary Figure 71 Funnel plot of study bias on HOMA-IR

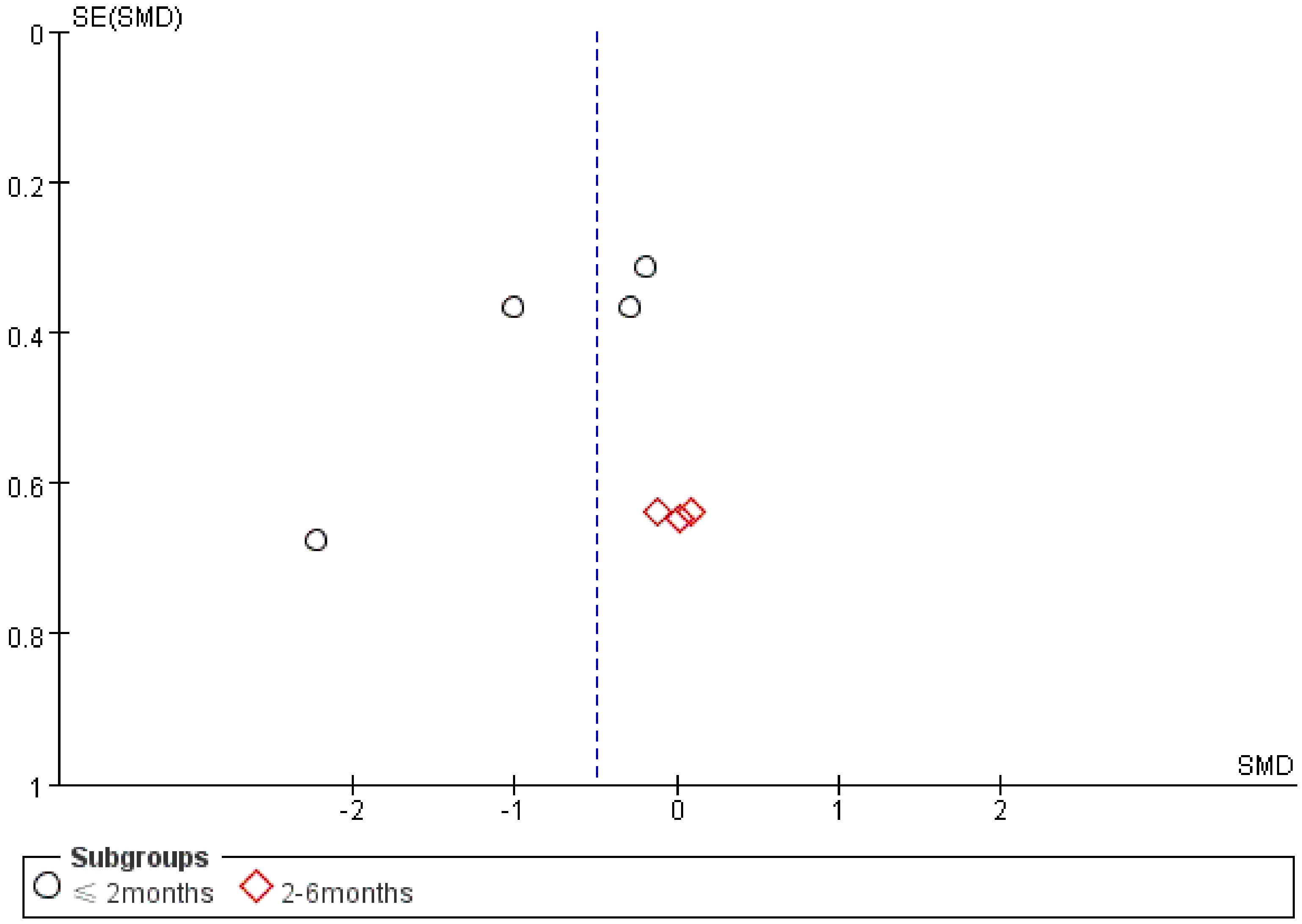

Supplementary Figure 72 Funnel plot of study bias on Fasting blood glucose

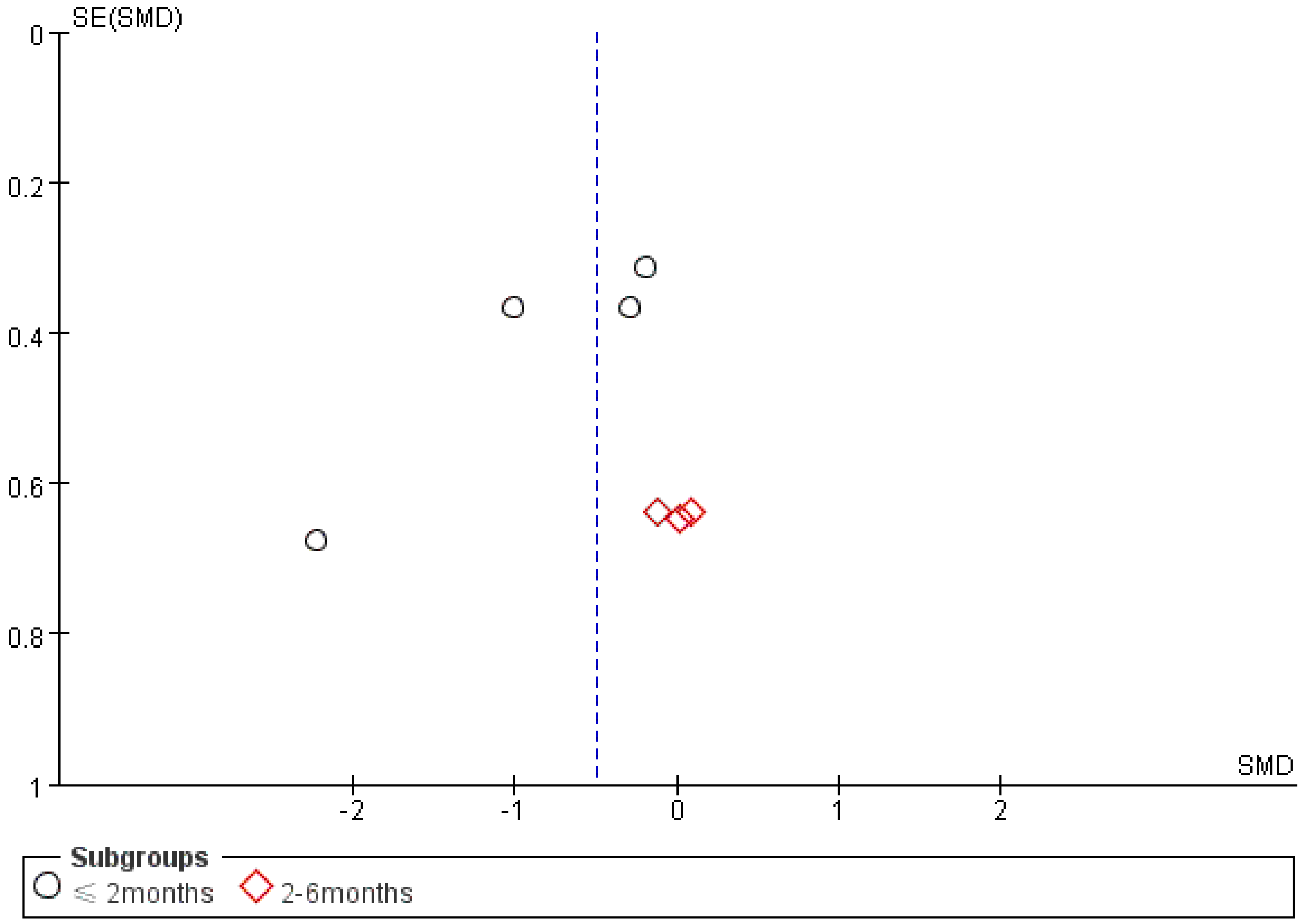

Supplementary Figure 73 Funnel plot of study bias on Fasting insulin

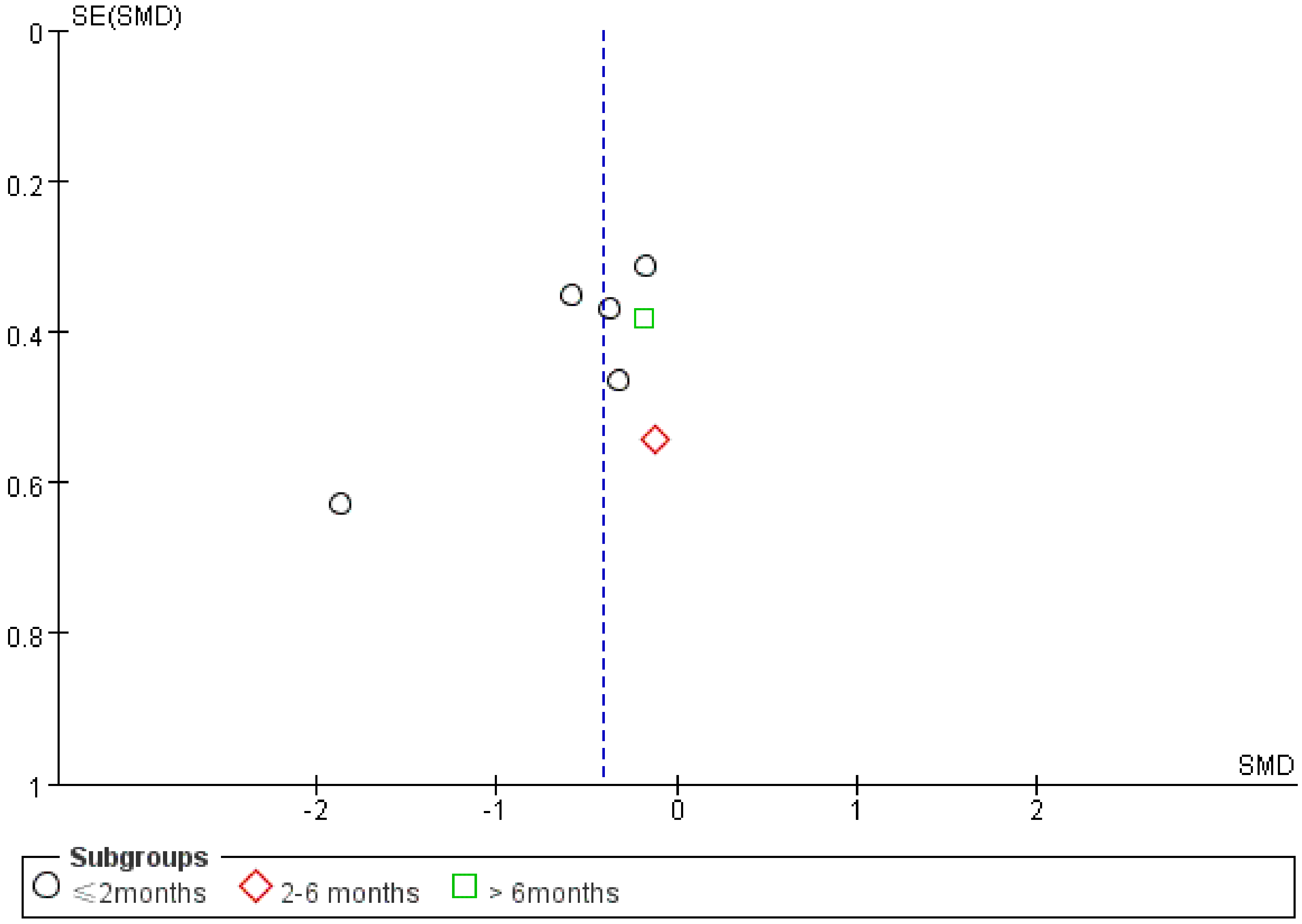

Supplementary Figure 74 Trim-and-fill funnel plot for Body mass in short-term subgroup

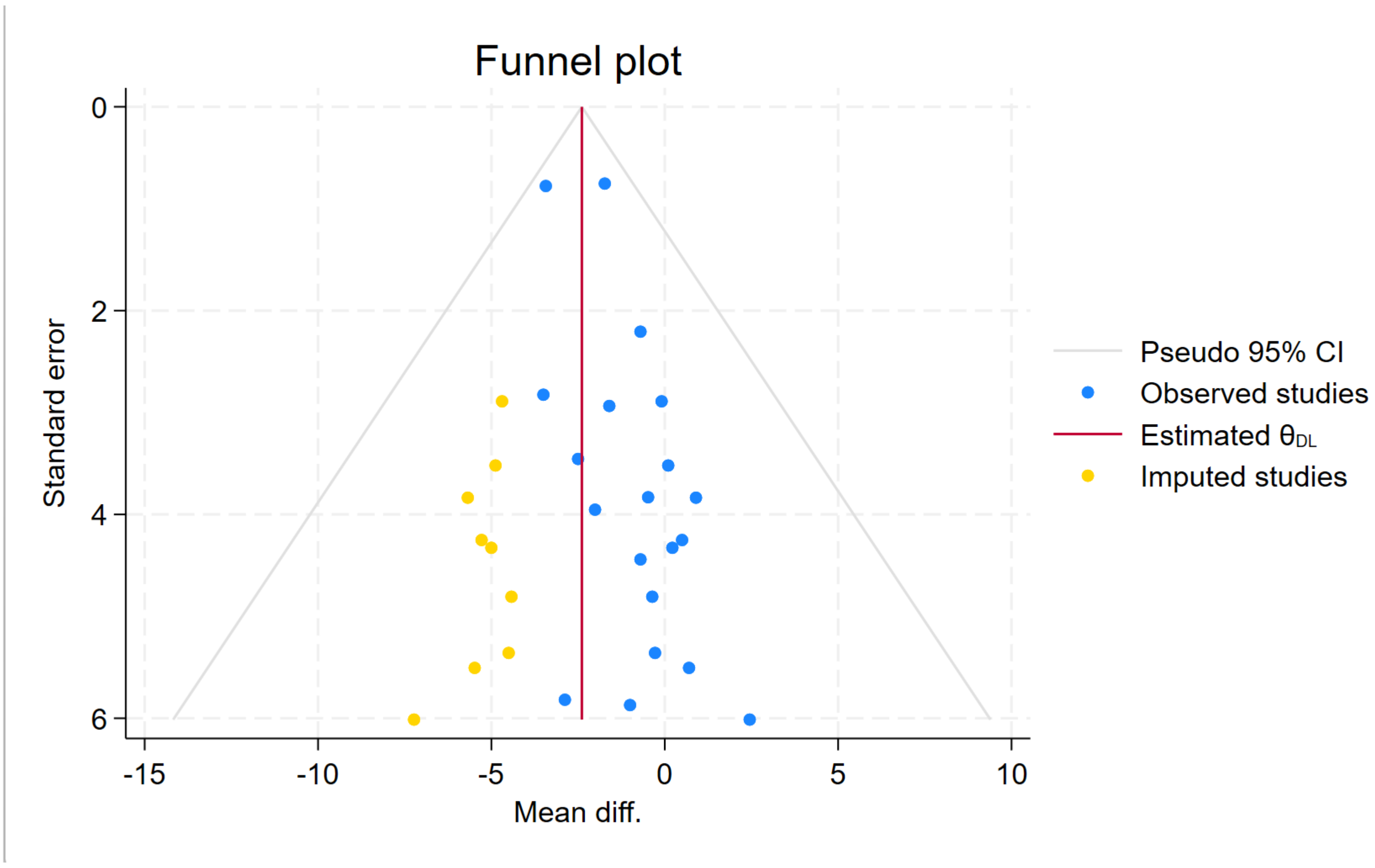

Supplementary Figure 75 Trim-and-fill funnel plot for BMI in short-term subgroup

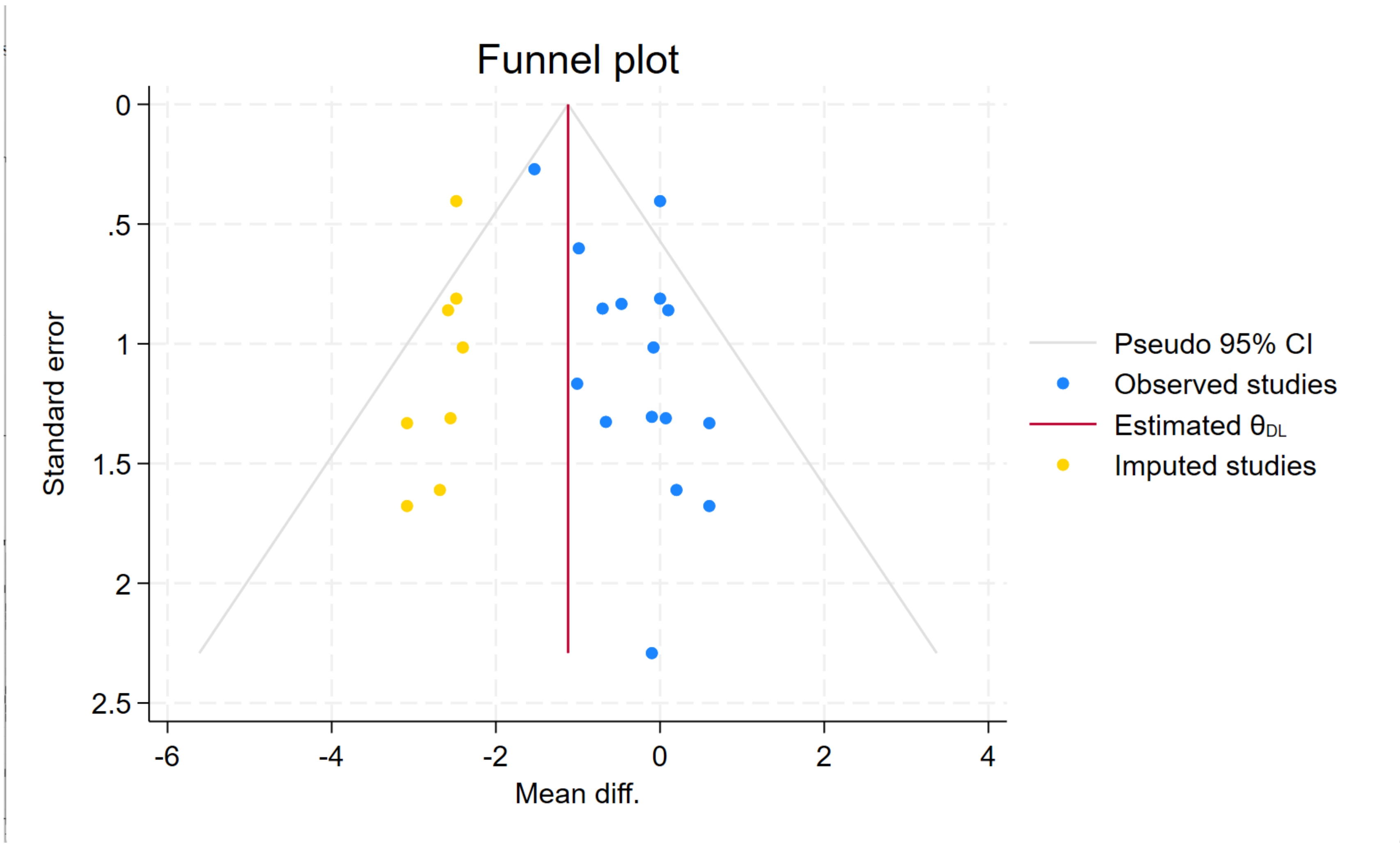

Supplementary Figure 76 Trim-and-fill funnel plot for Waist circumference in long-term subgroup

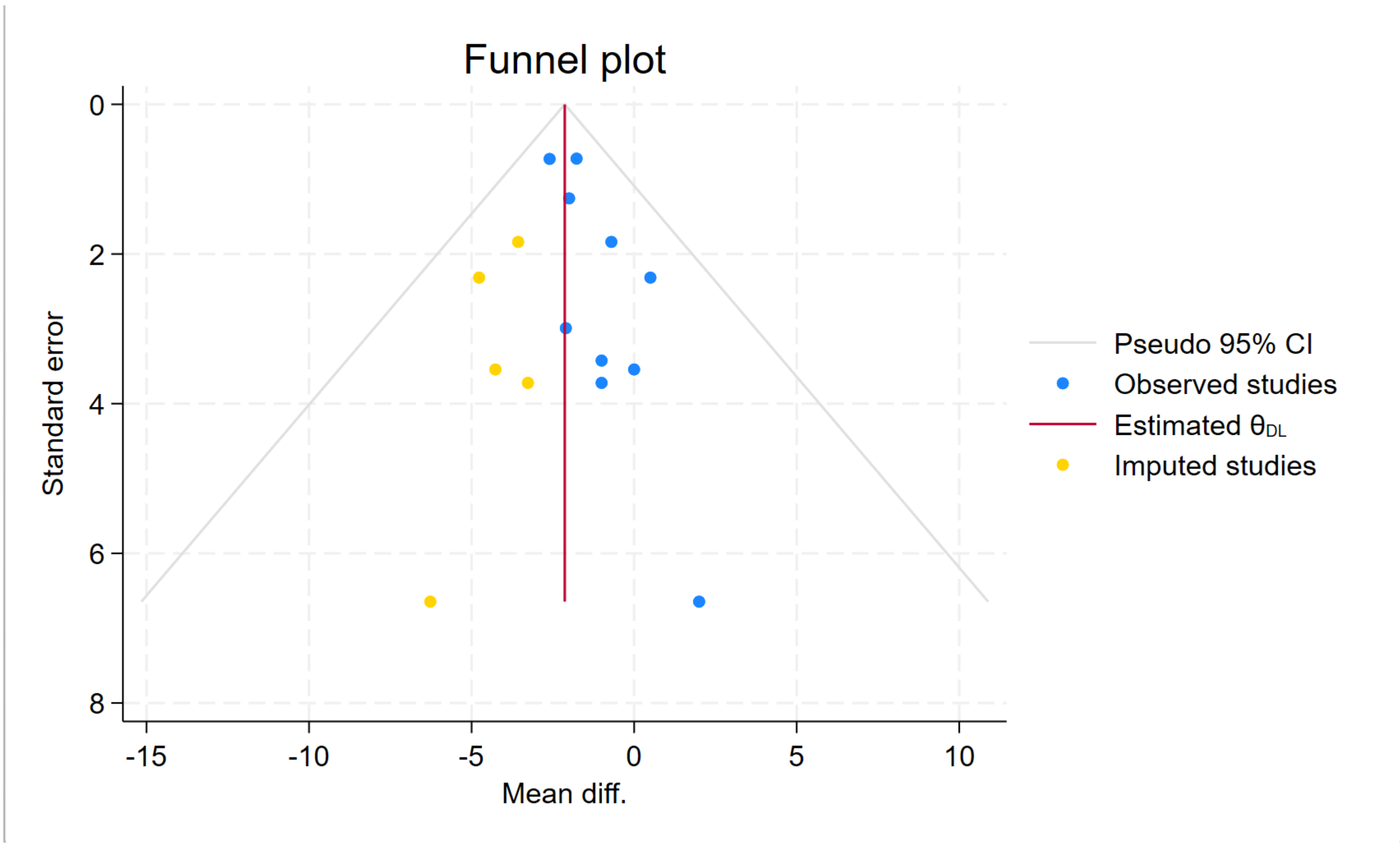

Supplementary Figure 77 Trim-and-fill funnel plot for Resting heart rate in short-term subgroup

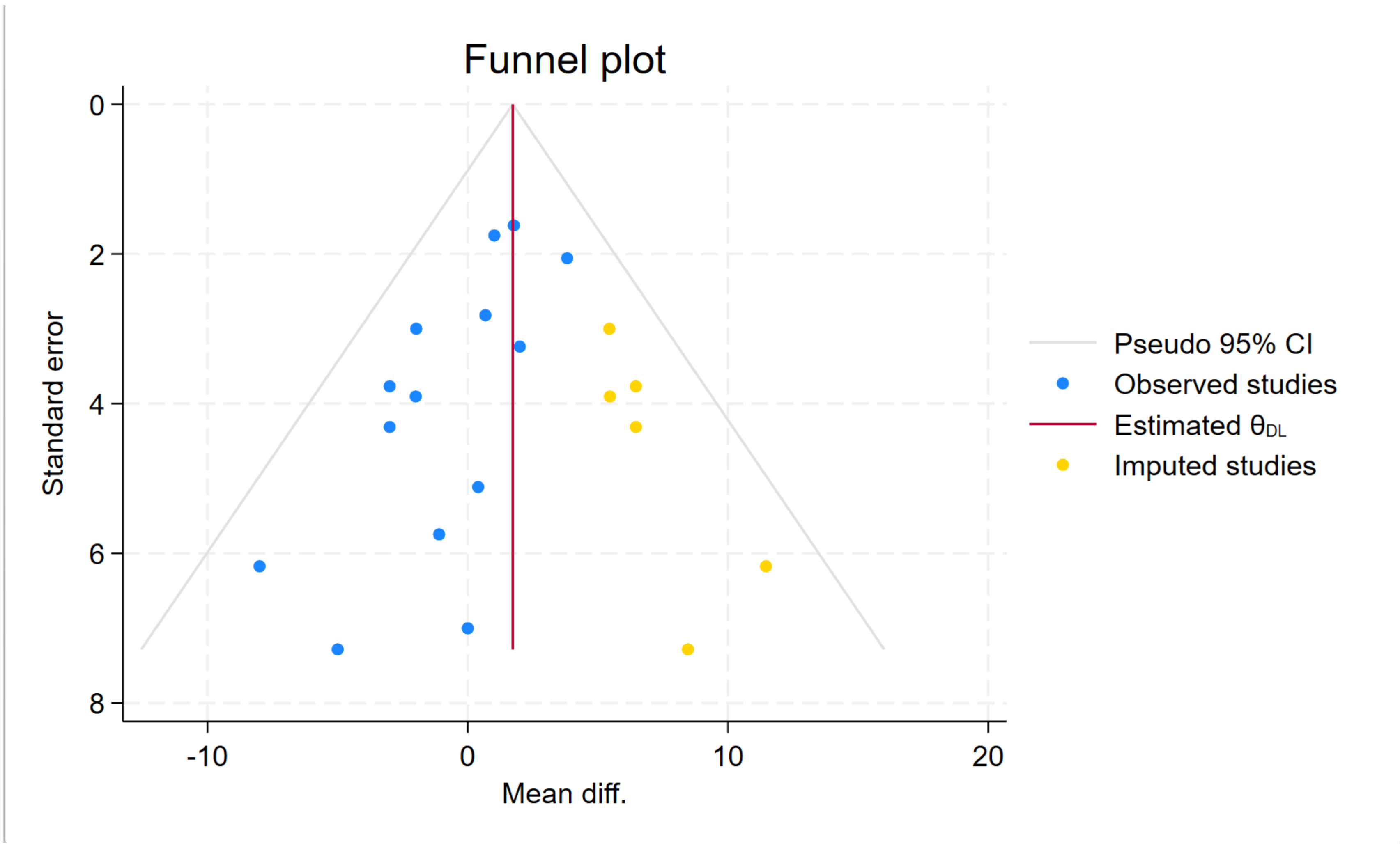

Supplement: Supplementary file 2 [file DataSheet2.pdf]
